# Conservation systematics of the shield-backed trapdoor spiders of the *nigrum*-group (Mygalomorphae, Idiopidae, *Idiosoma*): integrative taxonomy reveals a diverse and threatened fauna from south-western Australia

**DOI:** 10.3897/zookeys.756.24397

**Published:** 2018-05-09

**Authors:** Michael G. Rix, Joel A. Huey, Steven J.B. Cooper, Andrew D. Austin, Mark S. Harvey

**Affiliations:** 1 Biodiversity and Geosciences Program, Queensland Museum, South Brisbane, Queensland 4101, Australia; 2 Australian Centre for Evolutionary Biology and Biodiversity, and Department of Ecology and Evolutionary Biology, School of Biological Sciences, The University of Adelaide, Adelaide, South Australia 5005, Australia; 3 Department of Terrestrial Zoology, Western Australian Museum, Welshpool, Western Australia 6106, Australia; 4 Adjunct, School of Biological Sciences, The University of Western Australia, Crawley, Western Australia 6009, Australia; 5 Adjunct, School of Natural Sciences, Edith Cowan University, Joondalup, Western Australia 6027, Australia; 6 Evolutionary Biology Unit, South Australian Museum, Adelaide, South Australia 5000, Australia

**Keywords:** biodiversity hotspot, conservation biology, illustrated key, subfamily Arbanitinae, taxonomy, tribe Aganippini

## Abstract

The aganippine shield-backed trapdoor spiders of the monophyletic *nigrum*-group of *Idiosoma* Ausserer *s. l.* are revised, and 15 new species are described from Western Australia and the Eyre Peninsula of South Australia: *I. arenaceum* Rix & Harvey, **sp. n.**, *I. corrugatum* Rix & Harvey, **sp. n.**, *I. clypeatum* Rix & Harvey, **sp. n.**, *I. dandaragan* Rix & Harvey, **sp. n.**, *I. formosum* Rix & Harvey, **sp. n.**, *I. gardneri* Rix & Harvey, **sp. n.**, *I. gutharuka* Rix & Harvey, **sp. n.**, *I. incomptum* Rix & Harvey, **sp. n.**, *I. intermedium* Rix & Harvey, **sp. n.**, *I. jarrah* Rix & Harvey, **sp. n.**, *I. kopejtkaorum* Rix & Harvey, **sp. n.**, *I. kwongan* Rix & Harvey, **sp. n.**, *I. mcclementsorum* Rix & Harvey, **sp. n.**, *I. mcnamarai* Rix & Harvey, **sp. n.**, and *I. schoknechtorum* Rix & Harvey, **sp. n.** Two previously described species from south-western Western Australia, *I. nigrum* Main, 1952 and *I. sigillatum* (O. P.-Cambridge, 1870), are re-illustrated and re-diagnosed, and complementary molecular data for 14 species and seven genes are analysed with Bayesian methods. Members of the *nigrum*-group are of long-standing conservation significance, and *I. nigrum* is the only spider in Australia to be afforded threatened species status under both State and Commonwealth legislation. Two other species, *I. formosum* Rix & Harvey, **sp. n.** and *I. kopejtkaorum* Rix & Harvey, **sp. n.**, are also formally listed as Endangered under Western Australian State legislation. Here we significantly relimit *I. nigrum* to include only those populations from the central and central-western Wheatbelt bioregion, and further document the known diversity and conservation status of all known species.

## Introduction

**Figures 1–12.**
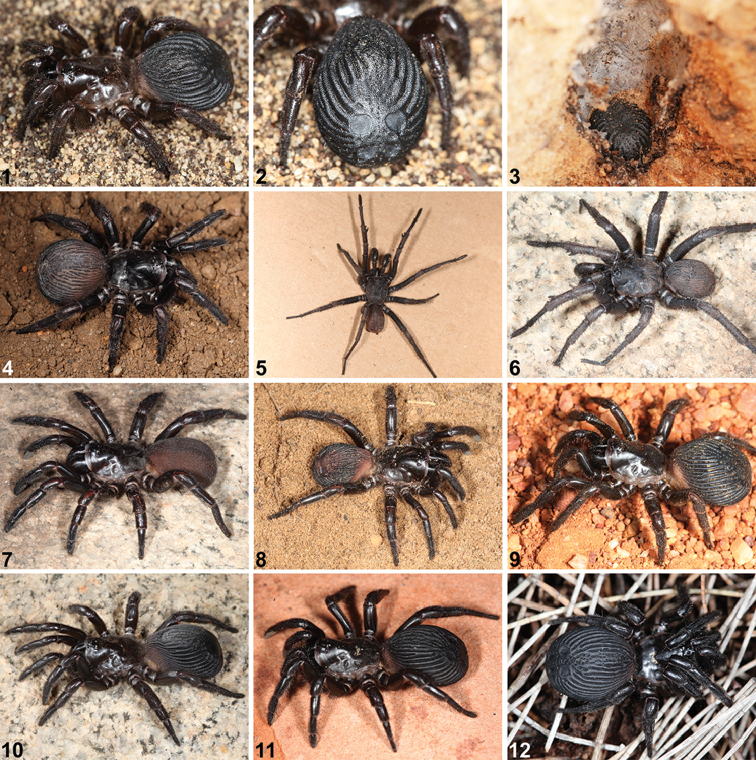
Live habitus images of shield-backed trapdoor spiders of the *Idiosoma nigrum*-group. **1–3** Female *I. nigrum* Main, 1952 (WAM T132737) from Minnivale Nature Reserve (Western Australia) **4** Female *I. sigillatum* (O. P.-Cambridge, 1870) (WAM T129191) from Karnup, Perth (Western Australia) **5** Male *I. sigillatum* (WAM T132564) from Crawley, Perth (Western Australia) **6** Male *I. sigillatum* (WAM T136936) from Wangara (Western Australia) **7** Female *I. jarrah* (WAM T136937) from Darlington (Western Australia) **8** Female *I. mcclementsorum* sp. n. (WAM T139469) from Julimar State Forest (Western Australia) **9** Female *I. kopejtkaorum* sp. n. from Charles Darwin Nature Reserve (Western Australia) **10** Female *I. schoknechtorum* sp. n. (WAM T140765) from SW. of York (Western Australia) **11–12** Female *I. arenaceum* sp. n. (WAM T141118) from Kalbarri National Park (Western Australia). Images 1–3, 6, 7, 9–12 by M. Harvey; 4 by Greg Anderson, used with permission; 5, 8 by M. Rix.

**Figures 13–24.**
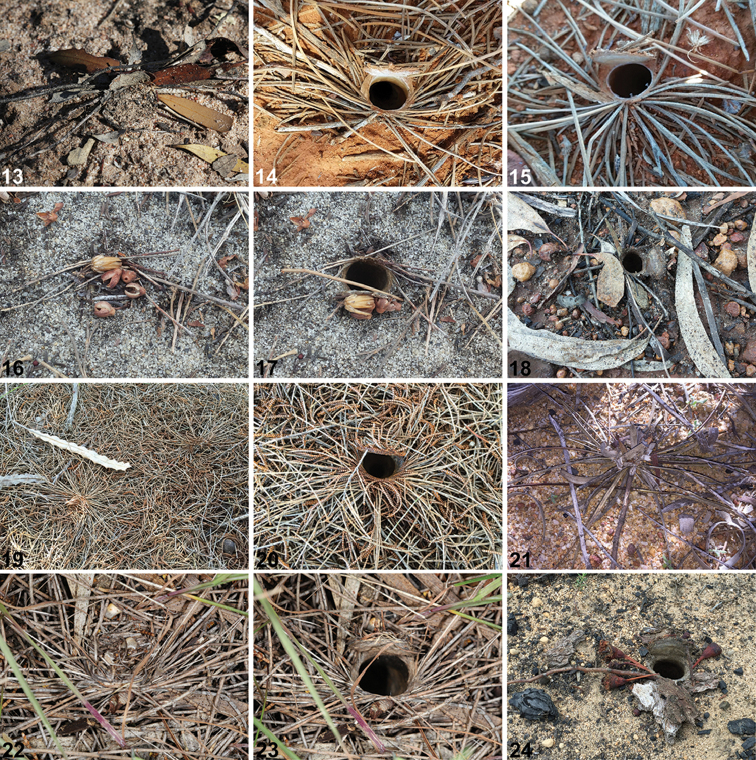
Burrows of shield-backed trapdoor spiders of the *Idiosoma nigrum*-group. **13** *I. nigrum* from Minnivale Nature Reserve (Western Australia) **14–15** *I. clypeatum* sp. n. from Weld Range (Western Australia) **16–17** *I. sigillatum* (O. P.-Cambridge, 1870) from Shenton Park, Perth (Western Australia) **18** *I. schoknechtorum* sp. n. from Meenaar Nature Reserve (Western Australia) **19–20** *I. arenaceum* sp. n. from Kalbarri National Park (Western Australia) **21** *I. kopejtkaorum* sp. n. from Charles Darwin Nature Reserve (Western Australia) **22–23** *I. jarrah* sp. n. from N. of Arthur River (Western Australia) **24** *I. mcclementsorum* sp. n. from Julimar State Forest (Western Australia). Note the ‘moustache-like’ arrangement of twig-lines characteristic of the *nigrum*-group. Images 13, 19–23, 24 by M. Harvey; 14–15 by Astrid Heidrich, used with permission; 16–18 by M. Rix.

The shield-backed trapdoor spiders of the genus *Idiosoma* Ausserer, 1871 (Figs 1–12), otherwise known as the *nigrum*-group (*sensu* Rix et al. 2017b, d), are an iconic and threatened component of the Australian mygalomorph spider fauna. Renowned for their unusual morphology and biology (Main 2003), they are of long-standing conservation significance, and *I. nigrum* Main, 1952 (Figs 1–3, 13) remains the only spider in Australia to be afforded threatened species status under the Commonwealth’s *Environmental Protection and Biodiversity Conservation Act 1999* (EPBC Act). In Western Australia, *I. nigrum* is also listed as Endangered under the *Western Australian Wildlife Conservation Act 1950* (WAWC Act), along with two other species newly described in this paper. Indeed, species of *Idiosoma* are fast becoming the face of terrestrial invertebrate conservation in Western Australia; the recognition of their conservation significance goes well beyond esoteric listing, and has directly impacted resource development proposals and the process of environmental impact assessment (EIA) for more than 15 years. Their prominence in popular science articles and books (e.g., Main 1964, 1967, 1976; Rix 2017) has further influenced a broader appeal within the public consciousness.

Shield-backed trapdoor spiders are atypical idiopids for a number of reasons. Among species of *Idiosoma*, they are unique in possessing an enlarged and sclerotised third pair of abdominal sigilla, an adaptation to phragmotic defence, the latter of which has also driven the evolution of corrugate, highly sclerotised abdominal ‘shields’ in a number of species (Figs 1–3, 9–12). Analogous to convergent morphologies in *I. galeosomoides* Rix, Main, Raven & Harvey, 2017 from south-western Australia, species of *Galeosoma* Purcell, 1903 (Idiopidae) from southern Africa, and species of *Cyclocosmia* Ausserer, 1871 (Ctenizidae) from North America and East Asia, these modified abdomens are able to be wedged midway down the burrow shaft upon disturbance (Fig. 3). This phragmotic behaviour presents the reinforced, sigillate abdominal ‘shield’ uppermost, presumably protecting the inhabitant from predatory attack (e.g., by wasps, ants and centipedes). Species of *Idiosoma* in the *nigrum*-group are also unusual in building a ‘moustache-like’ arrangement of twig-lines at the burrow entrance (Figs 13–24), a feature which aids enormously in field recognition. While numerous other species of *Idiosoma*, along with species of *Gaius* Rainbow, 1914 and some *Blakistonia* Hogg, 1902, also adorn their burrows with twig-lines (see Rix et al. 2017d, figs 65, 109, 254, 255), most of these are characterised by radial arrangements. All species in the *nigrum*-group are further endemic to the biodiversity hotspot of south-western Western Australia (see Rix et al. 2015), except for a single enigmatic species from the Eyre Peninsula of South Australia, tentatively included *incertae sedis* within the *nigrum*-group.

Main (1952) was the first to highlight the relationship between *I. sigillatum* (O. P-Cambridge, 1870) and the then newly described *I. nigrum*, at a time when relatively few specimens were available for study. These two species were the sole members of the genus *Idiosoma* for the next 65 years, until Rix et al. (2017b, d) demonstrated the paraphyly of the large genus *Aganippe* O. P.-Cambridge, 1877 relative to the *nigrum*-group (i.e., *Idiosoma* *s. s.*), and Rix et al. (2017d) formally synonymised the two genera, for which *Idiosoma* was the older name. In the years subsequent to its description, *I. nigrum* in particular became something of a spider icon in Western Australia, thanks to its Schedule 3 State listing as a threatened species in 1998, and the publication of numerous papers, books and popular science articles, mostly by B.Y. Main and colleagues (e.g., Main 1957a, b, 1962, 1964, 1967, 1976, 1985, 1986, 1990, 1996, 1999, 2003; Ellis 2015; Rix 2017; Rix et al. 2017b–d). However, in the last few decades it also became apparent that the *nigrum*-group included more than just two species of *Idiosoma*, as collections from the Perth Hills and other more remote areas of Western Australia revealed in increasing detail. Unfortunately, the identifications of some of these species were for a long time conflated, and *I. nigrum* was formally assessed under the EPBC Act in 2013 as what is now known to be five separate species. Revised assessment in 2017 under the WAWC Act clarified these taxonomic issues for the purposes of conservation legislation, with the result that *I. nigrum* was upgraded from Vulnerable to Endangered (W. A. Government Gazette 2018). However, the broader taxonomic problem has long persisted, with land managers, environmental consultants, ecologists, resources staff and the general public in need of an updated, publicly accessible taxonomy to reflect the actual diversity, distribution, and morphological complexity of this charismatic group of spiders.

The aims of this study are therefore three-fold. Firstly, using a multi-locus molecular dataset including 14 of the 17 recognised species, the monophyly and phylogeny of the *nigrum*-group of *Idiosoma* and the species therein are tested. Secondly, using all available collections (including both male and female specimens) and by reconciling molecular data with morphology, we present a revised taxonomy of the shield-backed trapdoor spiders of Australia, updating key distributional information and clarifying diagnostic autapomorphies. Finally, by applying International Union for the Conservation of Nature (IUCN) criteria, we assess the conservation status of all known species, and summarise the known biology and phenology of taxa where known. In total, 15 new species are described, increasing the total number of species in the *nigrum*-group to 17.

## Materials and methods

*Morphological methods*. Morphological methods, including the format of species descriptions and imaging techniques, follow Rix et al. (2017a, d), with measurements in millimetres and species presented in alphabetical order (following *I. nigrum*). All species are distinguished and diagnosed according to a generalised species concept, whereby morphological and molecular (for 14 of 17 species) data are combined to provide the operational criteria for distinguishing “separately evolving metapopulation lineages” (de Queiroz 2007: 880). Most available male specimens were illustrated for this study, either within the primary numbered plates or, for additional (non-holotype) specimens, as an ‘Atlas’ series of more rapidly assembled single-shot images in five standard views (see Suppl. material 1). The latter are included for ease of comparison to the type specimens, to directly illustrate the morphological differences within and between species, and to provide a comprehensive digital compendium of the material available in collections. For records with multiple specimens per vial, usually only a single exemplar specimen was imaged. For *I. sigillatum* (for which > 110 records were available), *I. clypeatum* sp. n. (19 records) and *I. jarrah* (21 records), a geographically and morphologically representative selection of 10 male specimens was imaged for each species (see Suppl. material 1). Maps were generated using the online Atlas of Living Australia (http://www.ala.org.au/ [accessed March 2018]), and are reproduced under a Creative Commons Attribution 3.0 Australia license.

Specimens are lodged at the Western Australian Museum, Perth (**WAM**), the Australian Museum, Sydney (**AMS**), the South Australian Museum, Adelaide (**SAM**) and the Oxford University Museum, Oxford (**OUM**), and the following abbreviations are used throughout the text:

**ALE** anterior lateral eye/s;

**AME** anterior median eye/s;

***COI*** cytochrome *c* oxidase subunit I;

***CYB*** cytochrome b;

**IBRA** Interim Biogeographic Regionalisation of Australia Version 7 (see https://www.environment.gov.au/land/nrs/science/ibra [accessed March 2018]; and abbreviations therein);

**ITS1–2** internal transcribed spacer 1–2;

***MRPL45*** 39S ribosomal protein L45 mitochondrial;

**PLE** posterior lateral eye/s;

**PME** posterior median eye/s;

**SP** sigilla pair (1–5);

***RPF2*** ribosome production factor 2 homolog;

**RTA** retrolateral tibial apophysis (of male pedipalp);

***XPNPEP3*** probable Xaa-Pro aminopeptidase 3.

For readability and ease of diagnosis, ‘sp. n.’ epithets are removed from the main text after the illustrated key to species.

*Molecular methods*. Nucleotide sequences for seven genes (*COI*, *CYB*, ITS1–2, *MRPL45*, *RPF2*, *XPNPEP3*) were generated for 39 specimens in the *Idiosoma nigrum*-group, using a next-generation parallel tagged amplicon sequencing (TAS) approach, described in detail by Rix et al. (2017b). For 22 additional specimens (codes: NCB_001–022), sequences were generated using standard bi-directional sequencing of polymerase chain reaction (PCR) amplicons, as per Castalanelli et al. (2017), using the same primers as Rix et al. (2017b). In total, sequences were available for 61 specimens of shield-backed trapdoor spiders, for all but three recognised species (see Suppl. material 2). For each specimen sequenced, project-specific DNA voucher codes are provided next to repository registration numbers in the material examined section for each species (below), in the form: [Registration^DNA_Voucher_Code^].

**Figure 25.**
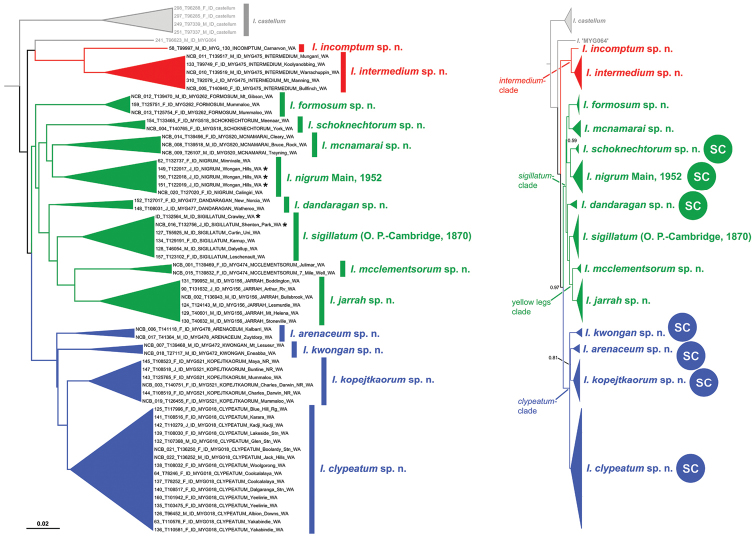
Tamura-Nei neighbour-joining tree of the COI dataset (at left) and Bayesian 50% majority-rule consensus tree resulting from a partitioned phylogenetic analysis of the 7 gene FULL dataset (at right) (both datasets with 82 taxa; most outgroups removed for ease of presentation). Shield-backed trapdoor spiders of the *Idiosoma nigrum*-group are coloured according to clade designations (with closely related outgroups in pale grey), and specimens of *I. nigrum* and *I. sigillatum* from their respective type localities are highlighted (*). Seven species with highly sigillate (sclerotised) abdomens in the polyphyletic ‘sigillate complex’ are denoted by circular ‘SC’ icons on the FULL dataset tree. Congruent species lineages that are concordant with morphological species concepts are collapsed on both trees, and posterior probabilities < 0.99 are shown adjacent to nodes on the FULL dataset tree (all other nodes have a posterior probability of 0.99–1.0). Note the erroneous position of 241_T99623 in the COI dataset tree; this species (‘MYG064’) is a close sister-lineage to the *nigrum*-group (as per the FULL dataset tree), and does not possess the morphological synapomorphies characteristic of other *nigrum*-group taxa. Note also that sequences were not available for *I. corrugatum* sp. n. (*incertae sedis*), *I. gardneri* sp. n. (*sigillatum*-clade) or *I. gutharuka* sp. n. (*intermedium*-clade).

Most outgroup sequences were obtained from data previously published by Rix et al. (2017b). The ultimate outgroup for the molecular analyses was the diplurid spider *Cethegus fugax* (Simon, 1908), and an undescribed species of *Prothemenops* Schwendinger, 1991 was also included in all analyses. Other outgroups included one species per genus for the aganippine genera *Bungulla* Rix, Main, Raven & Harvey, 2017, *Eucyrtops* Pocock, 1897, *Eucanippe* Rix, Main, Raven & Harvey, 2017 and *Gaius*, along with 15 additional specimens of *Idiosoma* belonging to the ‘south-western Australian clade’ as identified by Rix et al. (2017b). The latter included four specimens of *I. castellum* (Main, 1986) and one specimen of *Idiosoma* ‘MYG064’, both of which are closely related sister lineages to the *nigrum*-group (Fig. 25). In total, 21 outgroup taxa were included in the analyses (see Suppl. material 2), and these provided a broad and phylogenetically relevant selection of taxa to test the monophyly of the *nigrum*-group.

Gene alignments were conducted in Geneious R6 (Biomatters Ltd.; http://www.geneious.com/ [accessed March 2018]) using the MAFFT v7.017 plugin with default parameters (Katoh and Standley 2013). Individual alignments for the seven genes were concatenated to generate the FULL 82 taxon dataset (Suppl. material 4) which included all available data for all taxa. PartitionFinder Version 1.1.1 (Lanfear et al. 2012) was used to choose an optimal partitioning scheme, favouring a 14-partition model for the FULL dataset. The COI dataset (Suppl. materials 3, 5) was visualised with a Tamura-Nei neighbour-joining tree generated using Geneious R6 (Fig. 25), and uncorrected pairwise distances were calculated using MEGA Version 6.06 (Tamura et al. 2013). The FULL dataset was analysed in MrBayes Version 3.2.6 (Huelsenbeck and Ronquist 2001; Ronquist and Huelsenbeck 2003) via the CIPRES Science Gateway (Miller et al. 2010), with substitution model parameters estimated independently for each partition ([Unlink tratio = (all) pinvar = (all) shape = (all) statefreq = (all) revmat = (all)]) and rates allowed to vary across partitions ([Prset applyto = (all) ratepr = variable]). Four Markov Chain Monte Carlo (MCMC) chains were run for 40 million generations, sampling every 1000 generations, with the first 10% of sampled trees discarded as ‘burnin’ ([burnin = 4000]). Summary statistics of estimated parameters, including ESS values, were assessed using Tracer Version 1.6 (Rambaut et al. 2014), and FigTree Version 1.4.2 (http://tree.bio.ed.ac.uk/software/figtree/ [accessed March 2018]) was used to visualise the 50% majority-rule consensus tree (Fig. 25).

*Conservation assessments.* The conservation status of each species of *Idiosoma* was assessed using a standard International Union for the Conservation of Nature (IUCN) approach, similar to that applied by Harvey et al. (2015) for migid trapdoor spiders of the genus *Bertmainius* Harvey, Main, Rix & Cooper, 2015, and by Rix et al. (2017a) for the idiopid genus *Cataxia* Rainbow, 1914 in south-western Australia. As long term data on population reductions (Criterion A), population sizes or declines (Criterion C) or the number of mature individuals in any one population (Criterion D) were not available for most taxa, we assessed species using information on their geographic range (Criterion B) (as per Rix et al. 2017a), the latter calculated using area polygons in Google Earth Pro (Google Inc.). These assessments therefore focused on the extent of occurrence (EOO) of each species, the area of occupancy (AOO) within that range, and the health or otherwise of occupied habitats. Individual assessments are listed for each species under their relevant entry in the Systematics section (below), with precise EOO values reported within square brackets.

## Results and discussion

*Molecular analyses.* Fourteen of the 17 species of shield-backed trapdoor spiders were sampled for the molecular analyses; those not included were *I. corrugatum* (known from only a handful of specimens collected in 1959), *I. gardneri* (known from only a single specimen collected in 1989), and *I. gutharuka* (known from only a single specimen collected in 1996). The molecular and morphological data were unambiguously congruent, with monophyletic species lineages in the mitochondrial and multi-locus (nuclear plus mitochondrial) trees fully concordant with both each other and with the morphological hypotheses presented below (Fig. 25). Three well-supported deeper clades were resolved within the monophyletic and similarly well-supported *nigrum*-group, corresponding to the *intermedium*-clade, the *sigillatum*-clade and the *clypeatum*-clade (see Remarks under Systematics section, below) (Fig. 25). An undescribed species from south-western Western Australia, *Idiosoma* ‘MYG064’, was recovered as the immediate sister-species to the *nigrum*-group, closely followed by *I. castellum* (Fig. 25). Both the ‘tree-stem trapdoor spider’ (*I. castellum*) and *I.* ‘MYG064’ are also known to build a ‘moustache-like’ arrangement of twig-lines at their burrow entrances (e.g., see Main 1986, figs 2, 4i; Rix et al. 2017d, fig. 114; MSH, pers. obs.), raising the possibility that ‘moustache’ twig-lining behaviour is a key synapomorphy of this broader clade of *Idiosoma*.

Within the *nigrum*-group, extremely well known and well-sampled species like *I. sigillatum* and *I. jarrah* provided important contextual information regarding expected levels of intra-specific mitochondrial divergence, and corresponding divergence levels in the multi-locus phylogeny with nuclear data. Key sister-species relationships within the *sigillatum*-clade, also strongly supported by morphology (e.g., *I. formosum* + *I. mcnamarai*; *I. nigrum* + *I. schoknechtorum*; *I. jarrah* + *I. mcclementsorum*), revealed average interspecific mitochondrial pairwise distances of between 10.0–10.3% (Suppl. material 5). In the highly derived *clypeatum*-clade of four species, average interspecific mitochondrial p-distances were lower, varying from 7.0–9.0% (mean = 8.1%), but correspondingly higher than the intraspecific p-distances in the broadly distributed species *I. clypeatum* (the latter of which averaged just 5.4%) (Suppl. material 5).

While strong mitochondrial phylogeographic structure is evident among most populations of most species (e.g., a mean of 8.9% intraspecific pairwise divergence for *I. mcnamarai*; Fig. 25, Suppl. material 5), the deeper (multi-locus) phylogeny of the *nigrum*-group is generally characterised by very shallow branch lengths (Fig. 25), consistent with a rapid pulse of late Miocene speciation (as inferred by Rix et al. 2017b) during a period of intense aridification (see Byrne et al. 2008). The existence of a polyphyletic ‘sigillate complex’ of seven species (Fig. 25) is also evidence that highly phragmotic morphologies have evolved at least twice, a result which, upon close inspection, is also supported by the morphology of the SP4 sclerites on females. Increasingly effective abdominal ‘plugs’ likely offered a selective advantage to these spiders, and the transition from largely unmodified (symplesiomorphic) abdominal sigilla (e.g., in *I. incomptum* and *I. gutharuka*) to increasingly apomorphic ‘shield-like’ sigilla can be tracked across the phylogeny. Northern taxa in the *clypeatum*-clade further support the hypothesis of ‘derived xeric adaptation’ (see Rix et al. 2015), which posits that arid zone taxa are phylogenetically derived relative to their temperate congeners. Given that shield-backed trapdoor spiders belong to the restricted ‘south-western Australian clade’ of *Idiosoma* (*sensu* Rix et al. 2017b, fig. 7), *I. clypeatum* in particular is an example of a lineage that has ‘broken out’ of the temperate zone relatively recently, and is now distributed throughout most of the arid Murchison bioregion.

*Conservation assessments.* The conservation status of each species of *Idiosoma* was assessed using standard IUCN criteria, therefore providing an initial conservation ‘snapshot’ for those 12 species which have not already been formally assessed and/or listed under State or Commonwealth legislation. Three species (i.e., *I. nigrum*, *I. formosum* and *I. kopejtkaorum*), assessed in 2017 as Endangered under the *Western Australian Wildlife Conservation Act 1950* (approved 16 January 2018; see W. A. Government Gazette 2018), are restricted to the Avon Wheatbelt bioregion, which has suffered from high levels of historical land clearing, and continues to be degraded by dryland salinity, introduced pests and fungal dieback disease, among other threatening processes (see Keighery 2004; Environmental Protection Authority 2007; Laurance et al. 2011; Rix et al. 2017c). Four additional species from the Wheatbelt or northern Jarrah Forest bioregions (i.e., *I. dandaragan*, *I. mcclementsorum*, *I. mcnamarai*, and *I. schoknechtorum*) are also hereby assessed as Endangered, with one other (*I. gutharuka*) tentatively assigned as Critically Endangered. *Idiosoma sigillatum*, from the heavily cleared Swan Coastal Plain bioregion, was assessed by Rix et al. (2017c) as Vulnerable. Two species (i.e., *I. clypeatum* and *I. intermedium*) were assessed in 2017 as non-threatened ‘priority 3’ fauna, and two other species (i.e., *I. arenaceum* and *I. jarrah*) are also not currently considered to be of conservation concern. Unfortunately, four species (i.e., *I. corrugatum*, *I. gardneri*, *I. incomptum* and *I. kwongan*) are considered data deficient for the purposes of conservation assessment, although it is highly likely that one of these (*I. corrugatum*) is critically endangered or even extinct (see entry under this species, below).

Conservation considerations are necessarily central to our understanding of the *nigrum*-group of *Idiosoma*, for which the greatest threatening syndrome seems to be ongoing population decline followed by local extinction in already severely fragmented landscapes (e.g., the Avon Wheatbelt and Swan Coastal Plain). Main (1987, 2003) documented this process for populations of *I. nigrum* at East Yorkrakine Nature Reserve from 1989–1999, and on a private property (‘Fairfields’) near North Bungulla Nature Reserve from 1952–1980. Main (1990) also elegantly showed how populations of *I. sigillatum* in the Perth region were impacted by the city’s expansion during the 20^th^ Century, and more recently, Rix et al. (2017c) presented evidence for widespread declines among species of Idiopidae across southern Australia more generally, in the period since 1950. Maps presented in the current paper (and by extension, extent of occurrence calculations therein) make no distinction between extant or now extinct populations, many of which are represented only by older collection records from prior to 1960. Thus, for threatened taxa, ascertaining which populations are actually still viable is an urgent necessity, should we wish to prevent future extinctions. For a suite of other taxa, mining and resources development projects are having additional impacts, especially throughout the Murchison bioregion (*I. clypeatum*), the western Coolgardie bioregion (*I. intermedium*) and in the north-eastern Wheatbelt near Lake Moore (*I. kopejtkaorum* and *I. formosum*).

With morphological, distributional and molecular taxonomic resources now available for the shield-backed trapdoor spiders, we recommend that future conservation management strategies should focus on conducting rigorous surveys for burrow presence or absence, and on developing non-lethal and non-destructive methods for obtaining genetic material from wild animals (for the purposes of species designation and individual genotyping). These data will provide the quantitative foundations required for critical ongoing management, and for future assessment under IUCN Criteria A–D.

## Systematics

### Genus Idiosoma

Taxon classificationAnimaliaAraneaeIdiopidae

Ausserer, 1871

Idiosoma Ausserer, 1871: 150.
Aganippe O. P.-Cambridge, 1877: 28. Type species by subsequent designation (of Simon, 1892: 160) *Aganippe subtristis* O. P.-Cambridge, 1877 (synonymised by Rix et al. 2017d: 590).
Anidiops Pocock, 1897: 114. Type species by monotypy *Anidiops manstridgei* Pocock, 1897 (synonymised by Rix et al. 2017d: 590).

#### Type species.

*Idiops sigillatus* O. P.-Cambridge, 1870, by monotypy.

#### Diagnosis.

Species of *Idiosoma* can be distinguished from all other Arbanitinae by the presence of a median retrolateral digital process on the male pedipalp, distal to a burr-like RTA (this digital process secondarily reduced to a nubbin in a very few species) (Rix et al. 2017d). Males and females of most (but not all) *Idiosoma* species also possess at least one prominent pair of sclerotised sigilla on the dorsal abdomen, at position SP2 (also present in *Eucanippe*).

#### Distribution.

The genus *Idiosoma* has a broad distribution across mainland Australia in all States and Territories, mostly south of the Tropic of Capricorn and west of the Great Dividing Range (Rix et al. 2017d).

#### Composition and remarks.

*Idiosoma* was found to be the sister-genus to *Eucanippe* by Rix et al. (2017b), and includes 29 species, 15 of which are newly described in this study. A very large number of undescribed species are also known from museum collections (M. Rix, unpubl. data).

##### Shield-backed trapdoor spiders (the *Idiosoma nigrum*-group)

**Diagnosis and remarks.** The monophyletic *nigrum*-group comprises 17 species of *Idiosoma*, all but one of which are endemic to Western Australia. They can be distinguished from other congeners by the presence of a phragmotic abdominal morphology in which SP3 is uniquely sclerotised and always larger than SP2 (e.g., Figs 146, 151). Many (but not all) species possess prominent abdominal corrugations (e.g., Figs 2, 12), and the seven species in the polyphyletic ‘sigillate complex’ also have a heavily sclerotised, thickened and leathery cuticle which is somewhat truncate posteriorly (Figs 1–3, 9–12), giving the abdomen a reinforced, sigillate ‘shield-like’ morphology (hence the common name). Three clades are recognised: the *clypeatum*-clade (four species), the *intermedium*-clade (three species) and the *sigillatum*-clade (nine species) (Fig. 25). The sole species found outside of south-western Australia, *I. corrugatum*, is of uncertain affinity, due to a slightly unusual morphology and lack of sequence data.

Shield-backed trapdoor spiders are usually dark brown to black in life (Figs 1–12), and burrows are characterised by the presence of a ‘moustache-like’ arrangement of twig-lines and thin flap-type or wafer-type doors which are adorned with leaf litter debris (Figs 13–24). Most species have fairly restricted short-range endemic distributions (see Harvey 2002) which are sometimes overlapping or closely parapatric (Figs 374–377), although direct syntopy of multiple *nigrum*-group taxa in the field is rare.

##### Illustrated key to Australian shield-backed trapdoor spiders of the *Idiosoma nigrum*-group

NB. While females of *I. gardneri* sp. n., *I. gutharuka* sp. n., *I. incomptum* sp. n., and *I. kwongan* sp. n. are unknown, their likely position within the key is indicated (based on male morphology and phylogeny). See Suppl. material 1 for additional images of relevant character states for males.

**Table:** 

1	Males (17 of 17 known species)	**2**
–	Females (13 of 17 known species)	**17**
**MALES**		
2	Prolateral tibia I (Ti1) with clasping spurs (CS) oriented dorso-ventrally (2.1)	***I. corrugatum* sp. n.**
–	Prolateral tibia I with clasping spurs oriented longitudinally or nearly so (2.2, 2.3)	**3**
	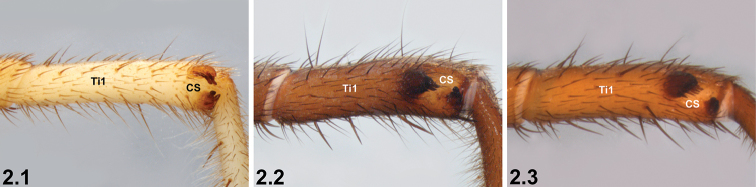	
3	SP3 sclerites only marginally larger than SP2 sclerites (3.1); SP4 unsclerotised or only barely sclerotised (3.1)	**4**
–	SP3 sclerites significantly larger than SP2 sclerites (3.2, 3.3); SP4 sclerites enlarged and sclerotised (3.2, 3.3)	**5**
	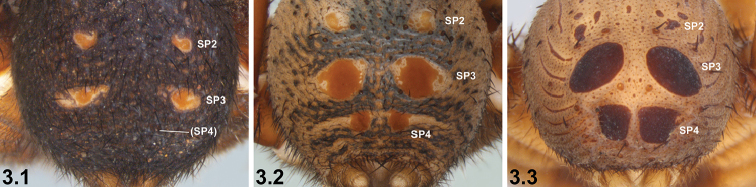	
4	Embolus (E) sharply tapering distally, with prominent longitudinal flange (F) (4.1)	***I. gutharuka* sp. n.**
–	Embolus without prominent flange, gradually tapering distally (4.2)	***I. incomptum* sp. n.**
	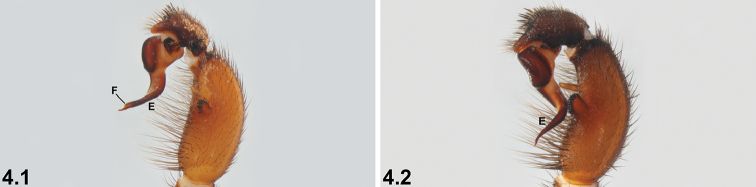	
5	Legs bi-coloured, with strongly contrasting bright yellow or orange-yellow femora (Fe) (5.1, 5.2)	**6**
–	Legs without strongly contrasting yellow femora (5.3) (NB. femora usually still relatively pale compared to other leg segments)	**7**
	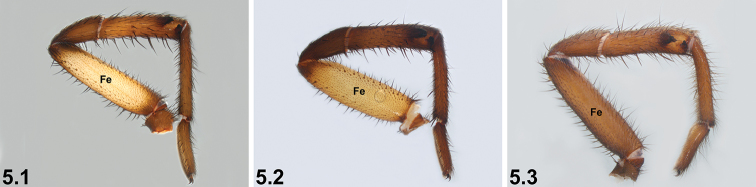	
6	SP3 sclerites relatively small; SP4 with weakly sclerotised spots (6.1)	***I. jarrah* sp. n.**
–	SP3 sclerites larger; SP4 with two relatively large sclerites (6.2)	***I. mcclementsorum* sp. n.**
	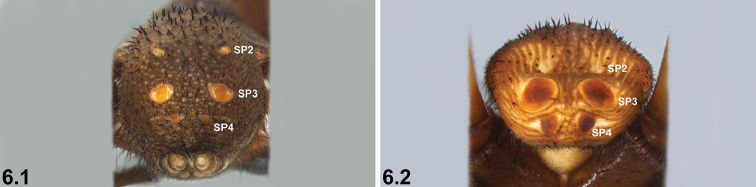	
7	SP4 with two long, elongate-oval (longitudinal) sclerites (7.1)	***I. arenaceum* sp. n.**
–	SP4 shorter, with two circular, broadly oval or subquadrate sclerites (7.2, 7.3)	**8**
	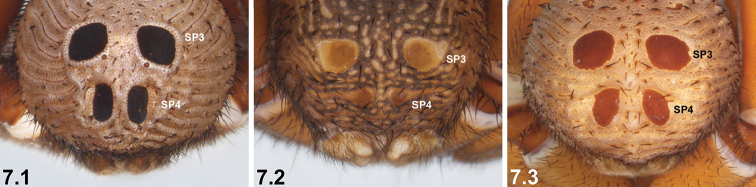	
8	Abdomen with distinctive SP2, each sclerite usually surrounded by contrasting pad of unsclerotised cuticle (P) (8.1, 8.2); SP4 each with long, transverse pad of unsclerotised cuticle (TP) extending laterally in chevron-like fashion (8.1, 8.2); abdomen without well-defined lateral sclerotic strips (8.1, 8.2)	**9**
–	SP2 poorly demarcated relative to surrounding abdomen (8.3); SP4 relatively large and well sclerotised, without long chevron-like pad of unsclerotised cuticle laterally (8.3); abdomen with well-defined lateral sclerotic strips (SS) (8.3)	**13**
	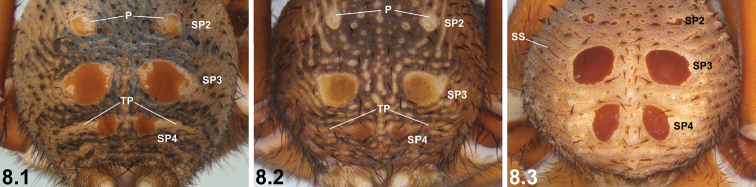	
9	Abdomen with well-defined dorso-lateral corrugations (LC) and striations (9.1)	**10**
–	Abdomen without such well-defined dorso-lateral corrugations or striations (9.2)	**11**
	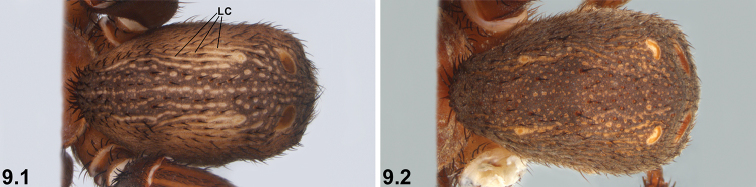	
10	SP3 sclerites relatively small, usually separated by approximately equal to, or more than, their own diameter (10.1); SP4 sclerites relatively small (10.1)	***I. sigillatum* (O. P.-Cambridge, 1870)**
–	SP3 sclerites larger, separated by less than their own diameter (10.2); SP4 sclerites larger (10.2)	***I. gardneri* sp. n.**
	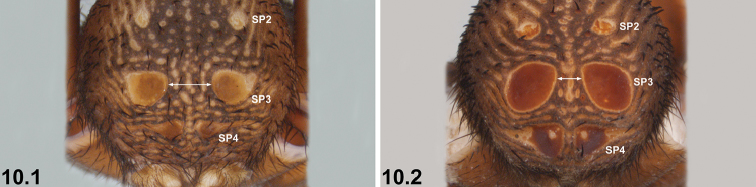	
11	Leg I tibia (Ti1) short and stout, with prolateral clasping spurs (CS) occupying most of distal half of segment (11.1); abdomen with ornate, bi-coloured pattern dorsally and postero-dorsally [NB. depending upon state of preservation] (11.2)	***I. formosum* sp. n.**
–	Leg I tibia longer, with prolateral clasping spurs occupying distal third of segment (11.3); abdomen more uniformly coloured dorsally (11.4)	**12**
	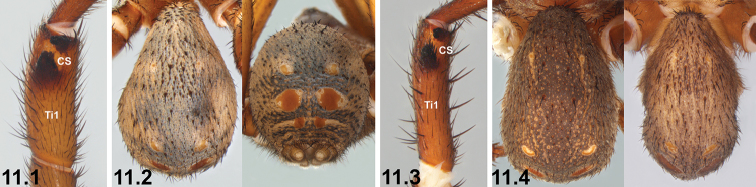	
12	SP3 each with unsclerotised, antero-laterally directed triangular ‘corner’ (TC) laterally (12.1, 12.2); SP4 sclerites relatively large (12.1, 12.2)	***I. mcnamarai* sp. n.**
–	Triangular ‘corners’ of SP3, if present, laterally or postero-laterally directed (12.3); SP4 sclerites relatively small (12.3)	***I. intermedium* sp. n.**
	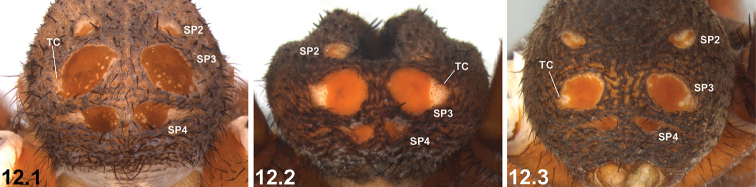	
13	SP4 each with distinctive, semi-circular lateral indentation (LI) adjacent to sclerite (13.1)	***I. kwongan* sp. n.**
–	SP4 without semi-circular lateral indentations (13.2, 13.3)	**14**
	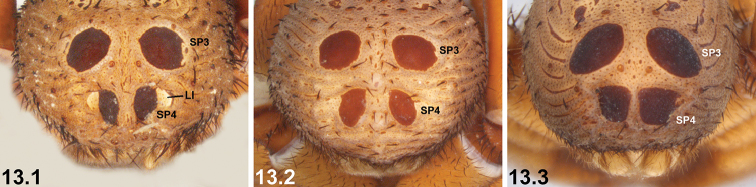	
14	Embolus (E) with prominent sub-distal embolic apophysis (EA) (14.1)	**15**
–	Embolic apophysis absent or nearly so (14.2, 14.3)	**16**
	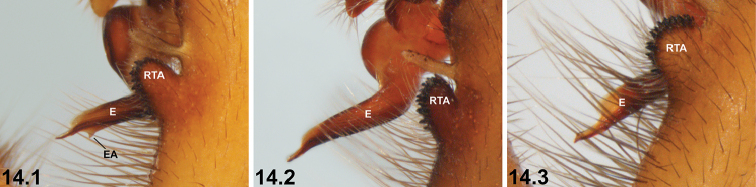	
15	Dorsal abdomen largely asetose (15.1); SP4 with two large subquadrate sclerites (15.2)	***I. nigrum* Main, 1952**
–	Dorsal abdomen with longitudinal rows of stiff, porrect black setae (15.3); SP4 with two circular or oval sclerites (15.4)	***I. dandaragan* sp. n.** and ***I. schoknechtorum* sp. n.*^A^***
	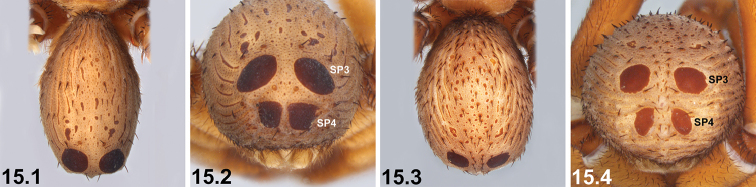	
16	Metatarsus I (Me1) heavily setose (16.1)	***I. clypeatum* sp. n.**
–	Metatarsus I setose, but not as heavily so (16.2)	***I. kopejtkaorum* sp. n.**
	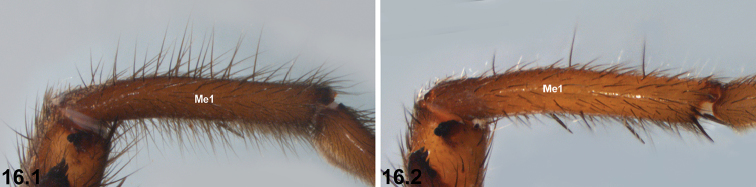	
**FEMALES**		
17	Abdomen with strongly corrugate morphology, bearing reinforced, sclerotised ridges dorsally and laterally (17.1, 17.2)	**18**
–	Abdominal cuticle largely unmodified, without reinforced, sclerotised ridges (but with minor corrugations normally still present) (17.3)	**24**
	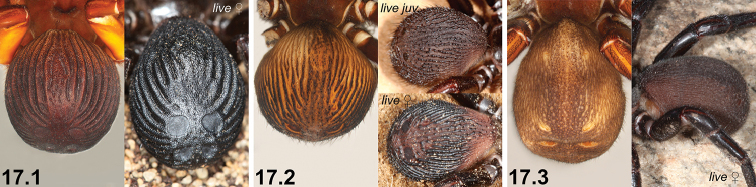	
18	Eye group compact, PLE–PLE/ALE–ALE ratio ~1.5 (18.1)	***I. corrugatum* sp. n.**
–	Eye group broadly trapezoidal, PLE–PLE/ALE–ALE ratio ≥ 2.0 (18.2, 18.3)	**19**
	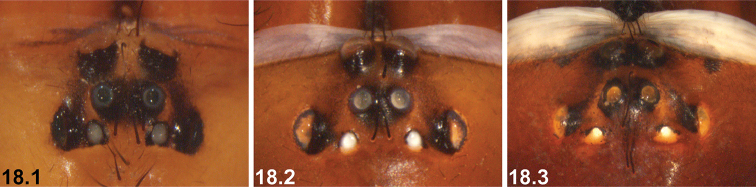	
19	Reinforced, sclerotised ridges on abdomen separated by longitudinal rows of less sclerotised cuticle (19.1, 19.2); SP2 sclerites defined and visible (19.1, 19.2)	**20 *^B^***
–	Entire posterior abdomen heavily sclerotised and strongly sigillate, with thickened, leathery (and usually moderately truncate) ‘shield-like’ morphology (19.3); SP2 sclerites obscured (19.3)	**21**
	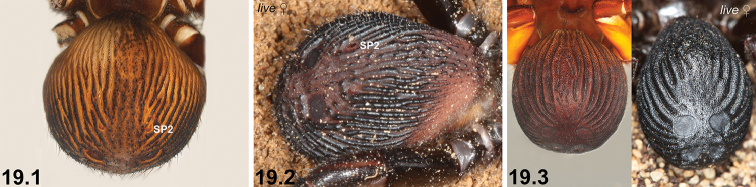	
20	SP3 sclerites relatively large (20.1); sclerotised ridges on abdomen well developed (20.1)	***I. mcclementsorum* sp. n.**
–	SP3 sclerites smaller (20.2); sclerotised ridges on abdomen less well developed, but only marginally so (20.2)	***I. sigillatum* (O. P.-Cambridge, 1870)**
	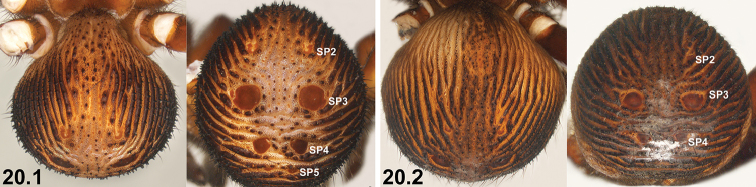	
21	SP4 with two long, elongate-oval (longitudinal) sclerites (21.1) [NB. sigilla sclerites artificially highlighted]	***I. arenaceum* sp. n.**
–	SP4 shorter, with two circular, oval or subquadrate sclerites (21.2, 21.3)	**22**
	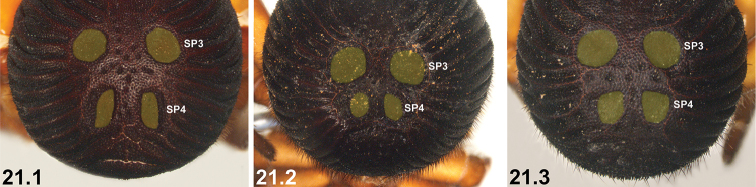	
22	SP4 sclerites approximately half the size of SP3 sclerites***^C^*** (22.1, 22.2) [NB. sigilla sclerites artificially highlighted]	***I. clypeatum* sp. n.** and ***I. kopejtkaorum* sp. n.*^D^***
–	SP4 sclerites larger, > 0.5 × the size of SP3 sclerites (22.3)	**23**
	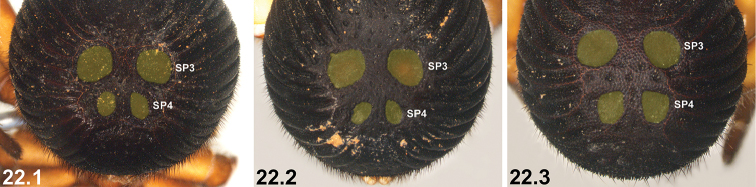	
23	SP4 with two large subquadrate sclerites (23.1) [NB. sigilla sclerites artificially highlighted]; posterior abdomen (anterior to spinnerets) heavily sclerotised, obscuring SP5 (23.1)	***I. nigrum* Main, 1952**
–	SP4 with two circular or broadly oval sclerites (23.2, 23.3); posterior abdomen (anterior to spinnerets) less sclerotised, with defined SP5 sclerites visible (23.2, 23.3)	***I. dandaragan* sp. n.** and ***I. schoknechtorum* sp. n.*^A^***
	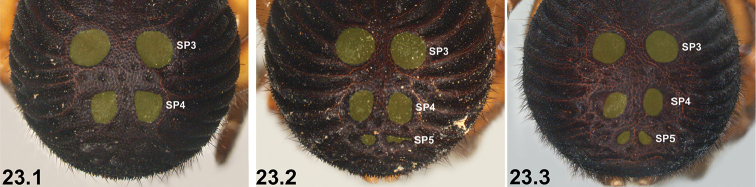	
24	Abdominal sigilla relatively small, SP4 sclerites not significantly larger than SP2 sclerites (24.1, 24.2)	**25 *^E^***
–	Abdominal sigilla larger, SP4 sclerites significantly larger than SP2 sclerites (24.3)	**26**
	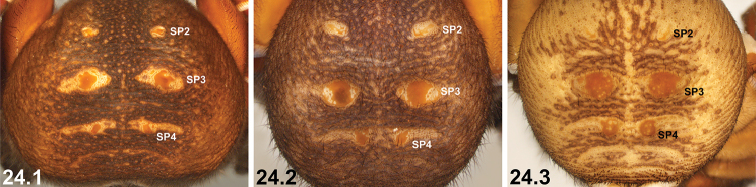	
25	SP3 and SP4 sclerites relatively small (25.1)	***I. jarrah* sp. n.**
–	SP3 and SP4 sclerites larger, but only marginally so (25.2)	***I. intermedium* sp. n.**
	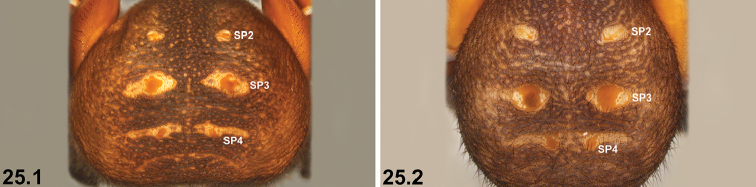	
26	Abdomen with ornate, bi-coloured pattern dorsally and postero-dorsally [NB. depending upon state of preservation] (26.1)	***I. formosum* sp. n.**
–	Abdomen more uniformly coloured dorsally (26.2); SP4 sclerites larger, but only marginally so	***I. mcnamarai* sp. n.**
	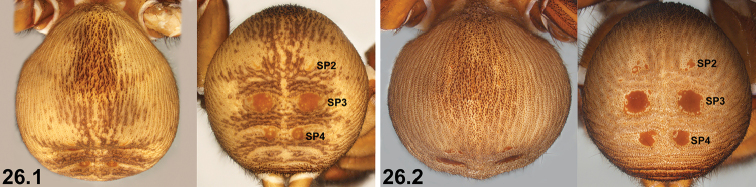	

***^A^*** By our assessment, males and females *I. dandaragan* sp. n. and *I. schoknechtorum* sp. n. appear to be indistinguishable morphologically; molecular data (Fig. 25) or geographic distribution (Fig. 374) are required for accurate identification.

***^B^*** The unknown female of *I. gardneri* sp. n. would likely key out to this couplet.

***^C^*** The unknown female of *I. kwongan* sp. n. would likely key out to this position.

***^D^*** By our assessment, females of *I. clypeatum* sp. n. and *I. kopejtkaorum* sp. n. appear to be indistinguishable morphologically; males, molecular data (Fig. 25) or geographic distribution (Fig. 374) are required for accurate identification.

***^E^*** The unknown females of *I. gutharuka* sp. n. and *I. incomptum* sp. n. would likely key out to this couplet.

### Idiosoma nigrum

Taxon classificationAnimaliaAraneaeIdiopidae

Main, 1952

Figs 1–3 , 13 , 25 , 26–35 , 36–38 , 39–47 , 48–56 , 374

Idiosoma nigrum Main, 1952: 133, pl. 1, figs 2–5, fig. 2C (in part; cited specimens from Wongan Hills, Bolgart and Goomalling). Main 1957b: 439, figs 2G–H, 12D, 14B, 24B (in part; cited specimens from Wongan Hills, Bindi Bindi, Bolgart, Botherling, N. of Bungulla, Calcarra, Goomalling, Koorda, Minnivale, W. of Nittymarra Hill, E. of Walebing and S. of Wyalkatchem). Main 1985: 13, figs 23, 27, 215. Rix et al. 2017d: 601, fig. 108.

#### Type material.

Holotype female. Wongan Hills (IBRA_AVW), Western Australia, Australia, 30°54'S, 116°43'E, 19 June 1952, B.Y. Main (WAM T3960; examined).

Paratype. 1 ♀, same data as holotype except 20 June 1952 (AMS KS6392).

#### Other material examined.

**AUSTRALIA: *Western Australia***: 1 ♀, Wongan Hills, Mount Matilda (IBRA_AVW), 30°49'14"S, 116°38'13"E, 5 November 2011, B.Y. Main (WAM T122017^DNA_Voucher_149^); 1 ♀, same data except 30°49'15"S, 116°38'12"E (WAM T122018^DNA_Voucher_150^); 1 juvenile, same data except 1 km NW. of Wongan Hills, 30°52'35"S, 116°42'46"E (WAM T122019^DNA_Voucher_151^); 1 ♀, Wongan Hills, water catchment area NW. of the town (IBRA_AVW), 30°53'S, 116°43'E, 5 November 2011, B.Y. Main, Ecologia staff (WAM T143998); 1 ♀, NW. of Wongan Hills, W. side of Wongan Hills about half way along range (IBRA_AVW), 30°52'S, 116°42'E, 19 June 1952, B.Y. Main (WAM T144765); 1 juvenile, same data (WAM T144766); 1 ♀, same data (WAM T144767); 1 juvenile, same data (WAM T144768); 1 ♀, same data (WAM T144769); 1 ♀, same data (WAM T144806); 1 ♀, same data (WAM T144807); 1 juvenile, same data (WAM T144808); 1 ♀, same data except 20 June 1952 (WAM T144770); 1 ♀, about 1 mile N. of Bindi Bindi (IBRA_AVW), 30°33'S, 116°21'E, 4 August 1955, B.Y. Main (WAM T144793); 1 ♀, same data (WAM T144794); 1 juvenile, same data except 20 May 1954 (WAM T144815); 1 ♀, 1 mile S. of Bolgart on main road (IBRA_AVW), 31°17'S, 116°31'E, 18 June 1952, B.Y. Main (WAM T144805); 1 juvenile, 7 miles W. of Bolgart (IBRA_AVW), 31°16'S, 116°28'E, 4 June 1953, B.Y. Main (WAM T144811); 1 ♀, Botherling (IBRA_AVW), 31°07'S, 116°48'E, 30 September 1952, B.Y. Main (WAM T144773); 1 ♀, first creek W. of Calcarra (IBRA_AVW), 31°08'S, 116°27'E, 4 May 1953, B.Y. Main (WAM T144810); 1 ♂, Durokoppin Nature Reserve, NW. tip, Transect E (IBRA_AVW), 31°24'S, 117°45'E, pitfall trap, 3 May–25 June 1988, B.Y. Main (WAM T139511); 1 ♀, East Yorkrakine Nature Reserve (IBRA_AVW), 31°23'S, 117°40'E, 8 December 2001, B.Y. Main (WAM T144622); 1 ♀, ‘Fairfields’, 9 miles N. of Bungulla (IBRA_AVW), 31°30'S, 117°35'E, 18 May 1956, B.Y. Main (WAM T144624); 1 juvenile, same data (WAM T144837); 1 ♀, same data except 10 March 1957 (WAM T144627); 1 ♀, same data (WAM T144838); 1 ♀, same data (WAM T144839); 3 ♀, same data (WAM T144840); 1 juvenile, same data (WAM T144842); 1 juvenile, same data except 1 September 1955 (WAM T144790); 1 juvenile, same data (WAM T144791); 1 juvenile, same data (WAM T144792); 1 ♀, Goomalling Reserve, 1 mile N. of town, near trotting course (IBRA_AVW), 31°17'S, 116°50'E, 18 June 1952, B.Y. Main (WAM T144764); 1 ♀, about half way between Kalguddering and Calingiri (W. of Nittymarra Hill) (IBRA_AVW), 31°02'S, 116°38'E, 4 May 1953, B.Y. Main (WAM T144779); 1 ♀, Minnivale, scrub on S. side of townsite (IBRA_AVW), 31°08'S, 117°11'E, 20 July 1954, B.Y. Main (WAM T144787); 1 ♀, Minnivale Nature Reserve, 19 km WNW. of Wyalkatchem (IBRA_AVW), 31°07'55"S, 117°11'39"E, hand collected from open mallee woodland, 21 April 2014, M.G. Rix, M.S. Harvey (WAM T132737^DNA_Voucher_62^); 1 juvenile, Minnivale Fauna Reserve (IBRA_AVW), 31°08'S, 117°11'E, 4 April 1984, B.Y. Main (WAM T144843); 1 ♀, same data except 18 May 1985 (WAM T144844); 1 ♀, 37 km N. of Minnivale (IBRA_AVW), 30°48'S, 117°13'E, 20 October 1984, B.Y. Main (WAM T144626); 1 ♀, 20.9 km E. of New Norcia (IBRA_AVW), 31°00'49"S, 116°25'37"E, dug from burrow, 15 September 2012, T. Sachse (WAM T127020^DNA_Voucher_NCB_020^); 1 ♂, North Bungulla, Lind Road, W. of Reserve (IBRA_AVW), 31°31'S, 117°35'E, pitfall trap, 23 June–4 August 1987, B.Y. Main (WAM T139514); 1 ♀, 5 miles E. of Walebing on Great Northern Highway (IBRA_AVW), 30°39'S, 116°17'E, 24 April 1955, B.Y. Main (WAM T144799); 1 juvenile, about 7 miles E. of Walebing (IBRA_AVW), 30°42'S, 116°20'E, 5 June 1953, B.Y. Main (WAM T144812); 1 juvenile, same data (WAM T144813); 1 juvenile, same data (WAM T144814); 1 ♂ ‘allotype’ [NB. not a paratype as subsequently designated by Main, 1957b: 439], Walk Walkin, via Koorda (IBRA_AVW), Western Australia, Australia, 30°50'S, 117°29'E, hand collected, 13 May 1940, G.F. Best (WAM T3301); 1 ♂, Walk Walkin Nature Reserve, site WH12 (IBRA_AVW), 30°48'08"S , 117°19'19"E, wet pitfall trap, 15 September 1998–25 October 1999, B. Durrant, CALM Survey (WAM T139510); 1 ♂, Wroth Road Nature Reserve, site JB6 (IBRA_AVW), 31°19'16"S, 116°33'38"E, wet pitfall trap, 15 September 1998–4 November 1999, N. Guthrie, CALM Survey (WAM T139515); 1 ♀, 1 mile S. of Wyalkatchem (IBRA_AVW), 31°12'S, 117°24'E, 20 July 1954, B.Y. Main (WAM T144785); 1 ♀, same data (WAM T144786).

#### Diagnosis.

*Idiosoma nigrum* is one of seven highly autapomorphic species in the polyphyletic ‘sigillate complex’ (Fig. 25); members of this complex can be distinguished from all other species in the *nigrum*-group from south-western Australia (i.e., *I. formosum*, *I. gardneri*, *I. gutharuka*, *I. incomptum*, *I. intermedium*, *I. jarrah*, *I. mcclementsorum*, *I. mcnamarai* and *I. sigillatum*) by the presence of well-defined lateral sclerotic strips on the male abdomen (e.g., Figs 32, 63, 256), and by the very heavily sclerotised, leathery, ‘shield-like’ morphology of the female abdomen (e.g., Figs 1–3, 9–12, 52, 74, 96). Males of *I. nigrum* can be further distinguished from those of *I. arenaceum* by the shape of the SP4 sclerites, which are not elongate-oval (Fig. 32; cf. Fig. 63); from *I. kwongan* by the absence of semi-circular lateral indentations adjacent to the SP4 sclerites (Fig. 32; cf. Fig. 278, Key pane 13.1); from *I. clypeatum* and *I. kopejtkaorum* by the presence of a prominent sub-distal embolic apophysis (Key pane 14.1; cf. Key panes 14.2, 14.3); and from *I. dandaragan* and *I. schoknechtorum* by the largely asetose dorsal abdomen (Fig. 27; cf. Figs 124, 330), and by the shape of the SP4 sclerites, which are large and subquadrate (Fig. 32; cf. Figs 129, 335).

Females can be distinguished from those of *I. arenaceum* by the shape of the SP4 sclerites, which are not elongate-oval (Figs 43, 52, Key pane 21.3; cf. Fig. 74, Key pane 21.1); from *I. clypeatum* and *I. kopejtkaorum* by the size of the SP4 sclerites, which are greater than half the size of the SP3 sclerites (Figs 43, 52, Key pane 22.3; cf. Figs 96, 267, Key panes 22.1, 22.2); and from *I. dandaragan* and *I. schoknechtorum* by the shape of the SP4 sclerites, which are subquadrate (Figs 43, 52, Key pane 21.3; cf. Figs 140, 346, Key panes 23.2, 23.3), and by the absence of well-defined SP5 sclerites (Figs 43, 52, Key pane 21.3; cf. Figs 140, 346, Key panes 23.2, 23.3) [NB. females of *I. kwongan* are unknown].

This species can also be distinguished from *I. corrugatum* (from the Eyre Peninsula of South Australia) by the shape of the prolateral clasping spurs on the male tibia I, which are oriented longitudinally (Fig. 34; cf. Fig. 109), and by the shape of the female eye group, which is broadly trapezoidal (Figs 42, 51; cf. Fig. 117).

#### Description (male WAM T3301).

**Figures 26–35.**
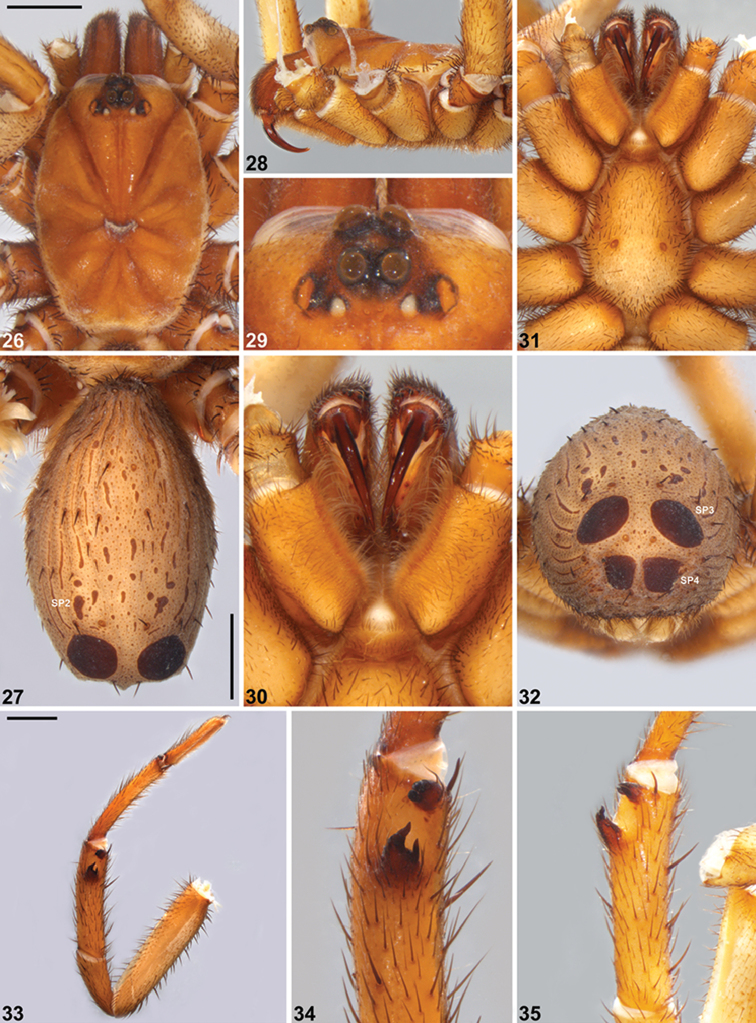
*Idiosoma nigrum* Main, 1952, male (WAM T3301) from Walk Walkin, via Koorda (Western Australia; AVW), somatic morphology. **26–27** Carapace and abdomen, dorsal view **28** Carapace, lateral view **29** Eyes, dorsal view **30** Mouthparts, ventral view **31** Sternum, ventral view **32** Abdomen, posterior view **33** Leg I, prolateral view **34** Leg I tibia, clasping spurs, prolateral view (note loss of clasping spur macroseta) **35** Leg I tibia, proventral view. Scale bars 2.0. SP2–4 = sigilla pairs 2–4.

**Figures 36–38.**
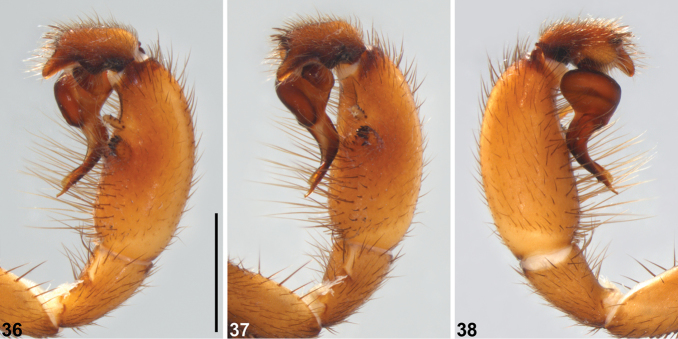
*Idiosoma nigrum* Main, 1952, male (WAM T3301) from Walk Walkin, via Koorda (Western Australia; AVW), pedipalp. **36** Retrolateral view **37** Retroventral view **38** Prolateral view. Scale bar 2.0.

Total length 16.1. Carapace 7.0 long, 4.8 wide. Abdomen 7.3 long, 4.4 wide. Carapace (Fig. 26) tan, with darker ocular region; lateral margins with uniformly-spaced fringe of porrect black setae; fovea procurved. Eye group (Fig. 29) trapezoidal (anterior eye row strongly procurved), 0.7 × as long as wide, PLE–PLE/ALE–ALE ratio 2.0; ALE almost contiguous; AME separated by less than their own diameter; PME separated by 3.4 × their own diameter; PME and PLE separated by slightly more than diameter of PME, PME positioned in line with level of PLE. Maxillae and labium without cuspules. Abdomen (Figs 27, 32) oval, beige-brown in dorsal view with lateral sclerotic strips, dorso-lateral corrugations and scattered dorsal sclerotic spots. Dorsal surface of abdomen (Fig. 27) largely asetose, with only a sparse assortment of stiff, porrect black setae, each with slightly raised, dark brown sclerotic base. Posterior abdomen strongly sigillate (Figs 27, 32); SP2 sclerites irregular, comma-shaped spots; SP3 sclerites very large and circular; SP4 sclerites subquadrate; SP5 obscured. Legs (Figs 33–35) variable shades of tan, with light scopulae on tarsi I–II; distal tibia I with pair of large prolateral clasping spurs oriented longitudinally; proximal-most clasping spur with missing macroseta. Leg I: femur 6.0; patella 3.0; tibia 4.4; metatarsus 4.6; tarsus 2.8; total 20.7. Leg I femur–tarsus/carapace length ratio 3.0. Pedipalpal tibia (Figs 36–38) 2.3 × longer than wide; RTA burr-like, with conical basal protuberance and field of retroventral spinules; digital process porrect, unmodified. Cymbium (Figs 36–38) setose, with field of spinules disto-dorsally. Embolus (Figs 36–38) broadly twisted and sharply tapering distally, with prominent longitudinal flange and triangular (sub-distal) embolic apophysis.

#### Description (female WAM T132737).

**Figures 48–56.**
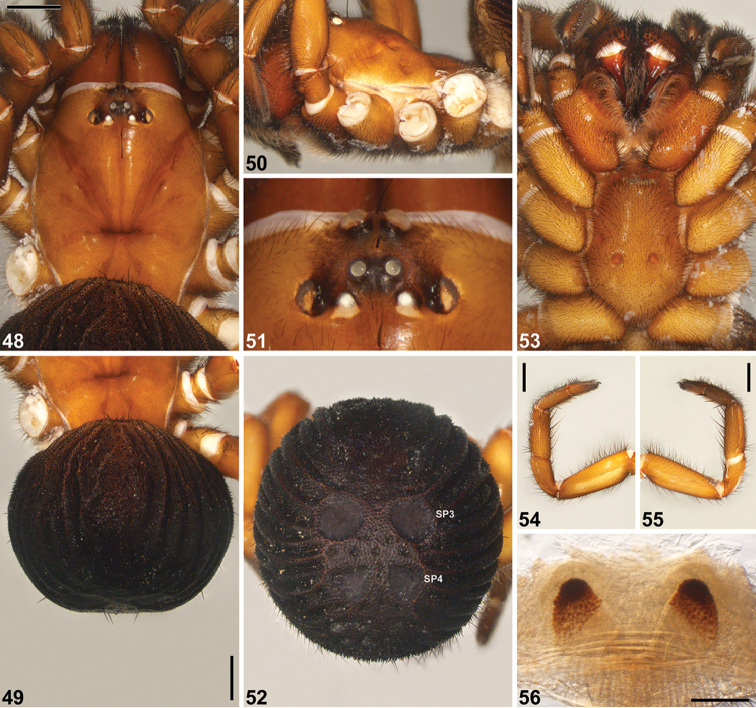
*Idiosoma nigrum* Main, 1952, female (WAM T132737) from Minnivale Nature Reserve (Western Australia; AVW). **48–49** Carapace and abdomen, dorsal view **50** Carapace, lateral view **51** Eyes, dorsal view **52** Abdomen, posterior view **53** Sternum, ventral view **54** Leg I, prolateral view **55** Leg I, retrolateral view **56** Spermathecae, dorsal view. Scale bars 2.0 (**48–49, 54–55**), 0.5 (**56**). SP3–4 = sigilla pairs 3–4.

Total length 19.6. Carapace 8.3 long, 6.6 wide. Abdomen 8.8 long, 10.2 wide. Carapace (Fig. 48) dark tan (dark brown-black in life; Fig. 1), with darker ocular region; fovea slightly procurved. Eye group (Fig. 51) trapezoidal (anterior eye row strongly procurved), 0.6 × as long as wide, PLE–PLE/ALE–ALE ratio 2.4; ALE almost contiguous; AME separated by approximately their own diameter; PME separated by 2.4 × their own diameter; PME and PLE separated by more than diameter of PME, PME positioned in line with level of PLE. Maxillae with field of cuspules confined to inner corner (Fig. 53); labium without cuspules. Abdomen (Figs 49, 52) dark brown-black (shiny black in life; Figs 1–3), corrugate and highly sclerotised, with leathery appearance typical of those species in the ‘sigillate complex’ (see Fig. 25). Posterior face of abdomen (Fig. 52, Key pane 23.1) with truncate ‘shield-like’ morphology; SP3 sclerites very large and circular; SP4 sclerites subquadrate; SP5 obscured by thickened cuticle. Legs (Figs 54–55) variable shades of dark tan (darker brown-black in life; Fig. 1); scopulae present on tarsi and metatarsi I–II; tibia I with two stout pro-distal macrosetae and row of five longer retroventral macrosetae; metatarsus I with eight stout macrosetae; tarsus I with distal cluster of short macrosetae. Leg I: femur 5.2; patella 3.4; tibia 3.3; metatarsus 2.6; tarsus 2.1; total 16.6. Leg I femur–tarsus/carapace length ratio 2.0. Pedipalp dark tan, spinose on tibia and tarsus, with thick tarsal scopula. Genitalia (Fig. 56) with pair of short, obliquely angled spermathecae, each bearing dense field of glandular vesicles distally, and more sparsely distributed glandular field sub-distally.

#### Distribution and remarks.

*Idiosoma nigrum* (Figs 1–3), a ‘sigillate complex’ member of the diverse *sigillatum*-clade (Fig. 25), has a restricted distribution in the central and central-western Wheatbelt bioregion of south-western Australia (Fig. 374). Its range roughly corresponds to the polygon demarcated by Bolgart, New Norcia, Walebing, and Bindi Bindi along its western margin, east to Koorda along its northern margin, south to Durokoppin and Kellerberrin along its eastern margin, and from Kellerberrin to Bolgart along its southern margin. While additional populations may occur slightly outside of this area, this range appears to roughly correspond to the area south and west of the 300 mm annual rainfall isohyet, bounded to the south by the Mortlock River and Avon River catchments, and to the west by the Lake Ninan and Lake Hinds catchments along the western edge of the Avon Wheatbelt bioregion. The distribution of *I. nigrum* closely abuts those of three other species in the ‘sigillate complex’ (Figs 25, 374): at its northern extent it nears the southern limit of the range of the distantly related *I. kopejtkaorum*; along its southern margin (near Northam and Meckering) it approaches the range of the closely related sister species *I. schoknechtorum*; and at its north-western extent (near New Norcia) it abuts the range of the closely related *I. dandaragan*. Unsurprisingly, all three species have in the past been confused with *I. nigrum* (e.g., Main 1957b), although morphology and/or molecular data can now be used to convincingly separate these species. Burrows are adorned with a ‘moustache-like’ arrangement of twig-lines (Fig. 13), and male specimens have been collected wandering in search of females in late autumn and winter.

#### Conservation assessment.

*Idiosoma nigrum* is the only spider in Australia to be afforded threatened species status under both State and Commonwealth legislation. In Western Australia in 2017, it was elevated from Vulnerable to Endangered (B1ab[iii] + B2ab[iii]) under the *Western Australian Wildlife Conservation Act 1950* (approved 16 January 2018; see W. A. Government Gazette 2018); this assessment incorporated the latest taxonomic, geographic and genetic data summarised in the current study (with a number of additional records also identified subsequently). At a Commonwealth level, under the *Environmental Protection and Biodiversity Act 1999* (EPBC), *I. nigrum* is listed as Vulnerable, although this EPBC assessment will need to be revised in light of current knowledge. The threats to the species are manifold, and its long-term persistence in the severely fragmented central Wheatbelt is tenuous (Main 2003). Its extent of occurrence is nearly 8,500 km^2^ [8,410 km^2^], and its area of occupancy within that range is < 500 km^2^. The current strongholds of *I. nigrum* are the larger nature reserves (NR) within its range, namely Durokoppin NR, East Yorkrakine NR, Minnivale NR, North Bungulla NR and Wongan Hills NR. However, multiple lines of evidence suggest populations are suffering severe contemporary declines (Main 2003; Rix et al. 2017c). Indeed, at Yorkrakine Rock NR it is now very difficult to find any idiopid trapdoor spiders, despite the seemingly healthy populations that once occurred at this site in the 1950s (see Main 1967; cf. Rix et al. 2017c). Similarly, at Durokoppin NR, only a single male was collected in pitfall traps set in 1988, and no males were collected during a long-term pitfall trapping survey run at four sites in the reserve from 1997–1998 (see Keighery 2004). Main (2003) further documented the decline of the East Yorkrakine Nature Reserve population during a long-term demographic survey run from 1989–1999, and Main (1987) highlighted a similar decline on a private property (‘Fairfields’) near North Bungulla Nature Reserve between 1952 and 1980. If remaining *I. nigrum* populations continue to decline at the current rate throughout the species’ range, a Critically Endangered assessment may need to be considered in the future.

### Idiosoma arenaceum

Taxon classificationAnimaliaAraneaeIdiopidae

Rix & Harvey sp. n.

http://zoobank.org/2D4DE886-0589-4BFF-9BA4-D59BB03AFD74

Figs 11 , 12 , 19 , 20 , 25 , 57–66 , 67–69 , 70–78 , 374

Idiosoma ‘*nigrum*’ Main, 1957b: 440 (in part; cited specimens from Canna, N. of Galena and Woolaga Creek).

#### Type material.

Holotype male. Zuytdorp, site ZU1 (IBRA_GES), Western Australia, Australia, 27°15'42"S, 114°01'30"E, wet pitfall trap, 19 May–17 August 1995, N. Hall, WAM-CALM Carnarvon Survey (WAM T139527).

Paratype. 1 ♂, same data as holotype (WAM T41787).

#### Other material examined.

**AUSTRALIA: *Western Australia***: 2 ♂, Zuytdorp, site ZU3 (IBRA_GES), 27°15'34"S, 114°04'03"E, wet pitfall trap, 18 May–16 August 1995, N. Hall, WAM-CALM Carnarvon Survey (WAM T41364^DNA_Voucher_NCB_017^); 1 ♂, Zuytdorp Nature Reserve, site ZU2 (IBRA_GES), 27°15'41"S, 114°01'48"E, wet pitfall trap, 18 May–17 August 1995, N. Hall, WAM-CALM Carnarvon Survey (WAM T41788); 1 ♀, creek just N. of Canna (Irwin River system) (IBRA_AVW), 28°54'S, 115°52'E, 5 October 1953, B.Y. Main (WAM T144780); 1 ♀, 2.3 miles S. of Ebano Creek in Woolaga Creek (IBRA_AVW), 29°12'S, 115°39'E, 29 May 1954, B.Y. Main (WAM T144783); 1 juvenile, same data (WAM T144784); 1 juvenile, Galena, 2 miles N. of Murchison River on North-west Coastal Highway (IBRA_GES), 27°48'S, 114°42'E, 9 July 1954, B.Y. Main (WAM T144788); 1 ♂, Geraldton (IBRA_GES), 28°47'S, 114°37'E, 1943, E.G. Osborne (AMS KS17388); 1 ♀, same data (AMS KS6393); 1 ♀, same locality data except 28°46'S, 114°37'E, 16 February 1983, A. Mollan (WAM T27120); 1 ♂, Geraldton, Minnenooka (IBRA_GES), 28°49'S, 114°54'E, 19 August 1971, J. Bryo (WAM T27119); 1 ♀, Kalbarri National Park, George Grey Drive, 18 km (by road) S. of Kalbarri (IBRA_GES), 27°51'22"S, 114°09'02"E, dug from burrow, 17 August 2016, M.S. Harvey, M.E. Blosfelds (WAM T141118^DNA_Voucher_NCB_006^); 1 ♀, 27 km S. of Kalbarri (IBRA_GES), 27°57'S, 114°10'E, 23 September 1989, D. Mead-Hunter (WAM T21810); 1 ♀, 18 miles S. of Mullewa on Mingenew Road (IBRA_GES), 28°48'S, 115°31'E, 15 July 1955, B.Y. Main (WAM T144835); 1 ♀, same data (WAM T144836); 1 ♀, N. side of Murchison River (IBRA_GES), 27°50'S, 114°41'E, 9 July 1954, B.Y. Main (WAM T144817); 1 ♀, same data (WAM T144789); 1 ♂, Northampton (IBRA_GES), 28°21'S, 114°38'E, hand collected under sheet of iron, 1 January 1982, Dr Allan (WAM T27123); 1 ♀, Sunset Beach (IBRA_GES), 28°43'S, 114°37'E, 9 September 1997, T. Burrows (WAM T44355).

#### Etymology.

The specific epithet is derived from the Latin *arenaceus* (adjective: ‘of sand’, ‘sandy’; see Brown 1956), in reference to the near-coastal sandy habitats occupied by this species in the Geraldton Sandplains bioregion.

#### Diagnosis.

*Idiosoma arenaceum* is one of seven highly autapomorphic species in the polyphyletic ‘sigillate complex’ (Fig. 25); members of this complex can be distinguished from all other species in the *nigrum*-group from south-western Australia (i.e., *I. formosum*, *I. gardneri*, *I. gutharuka*, *I. incomptum*, *I. intermedium*, *I. jarrah*, *I. mcclementsorum*, *I. mcnamarai* and *I. sigillatum*) by the presence of well-defined lateral sclerotic strips on the male abdomen (e.g., Figs 32, 63, 256), and by the very heavily sclerotised, leathery, ‘shield-like’ morphology of the female abdomen (e.g., Figs 1–3, 9–12, 52, 74, 96). Males and females of *I. arenaceum* can be further distinguished from those of all other known ‘sigillate complex’ species (i.e., *I. clypeatum*, *I. dandaragan*, *I. kopejtkaorum*, *I. kwongan*, *I. nigrum* and *I. schoknechtorum*) by the shape of the SP4 sclerites, which are longitudinally elongate-oval (Figs 63, 74, Key panes 7.1, 21.1) [NB. females of *I. kwongan* are unknown].

This species can also be distinguished from *I. corrugatum* (from the Eyre Peninsula of South Australia) by the shape of the prolateral clasping spurs on the male tibia I, which are oriented longitudinally (Fig. 65; cf. Fig. 109), and by the shape of the female eye group, which is broadly trapezoidal (Fig. 73; cf. Fig. 117).

#### Description (male holotype).

**Figures 57–66.**
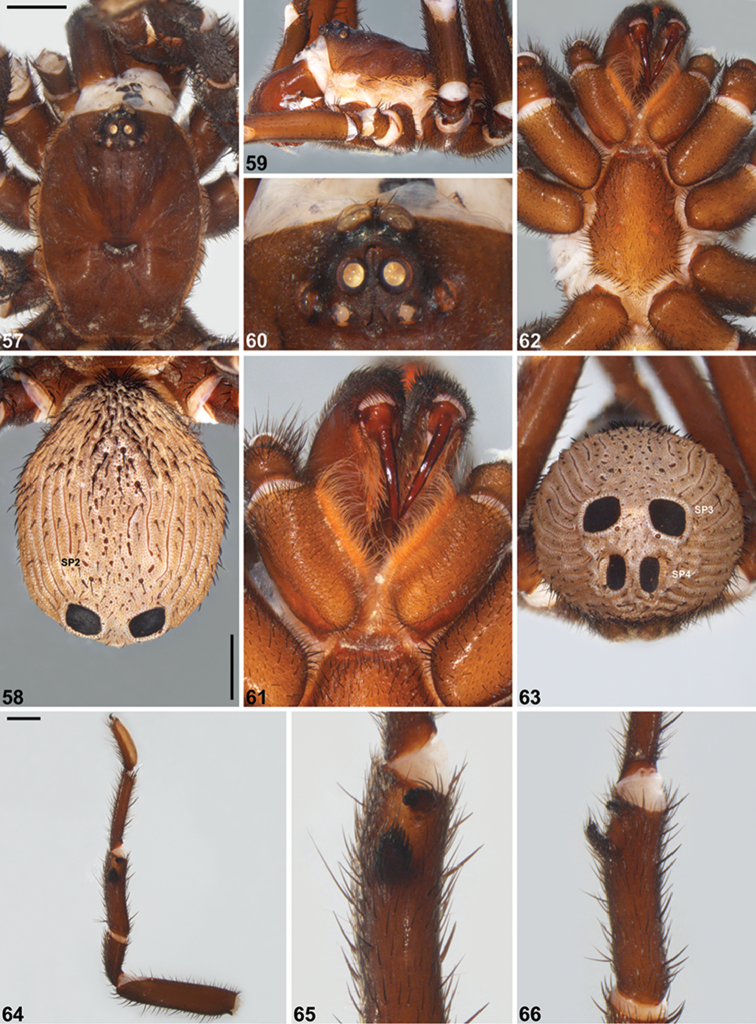
*Idiosoma arenaceum* sp. n., male holotype (WAM T139527) from Zuytdorp (Western Australia; GES), somatic morphology. **57–58** Carapace and abdomen, dorsal view **59** Carapace, lateral view **60** Eyes, dorsal view **61** Mouthparts, ventral view **62** Sternum, ventral view **63** Abdomen, posterior view **64** Leg I, prolateral view **65** Leg I tibia, clasping spurs, prolateral view **66** Leg I tibia, proventral view. Scale bars 2.0. SP2–4 = sigilla pairs 2–4.

**Figures 67–69.**
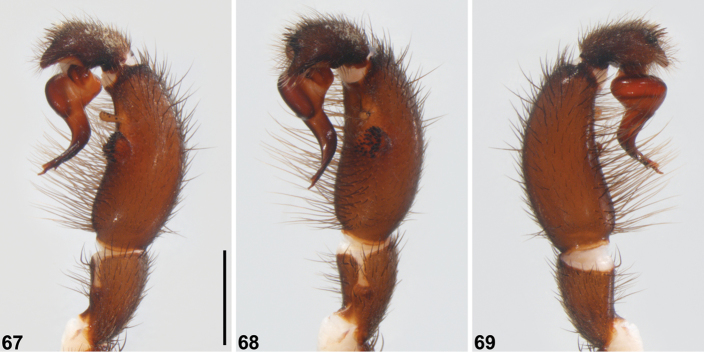
*Idiosoma arenaceum* sp. n., male holotype (WAM T139527) from Zuytdorp (Western Australia; GES), pedipalp. **67** Retrolateral view **68** Retroventral view **69** Prolateral view. Scale bar 2.0.

Total length 19.7. Carapace 8.0 long, 5.6 wide. Abdomen 8.4 long, 6.4 wide. Carapace (Fig. 57) dark chocolate-brown, with darker ocular region; lateral margins with uniformly-spaced fringe of porrect black setae; fovea procurved. Eye group (Fig. 60) trapezoidal (anterior eye row strongly procurved), 0.8 × as long as wide, PLE–PLE/ALE–ALE ratio 2.0; ALE almost contiguous; AME separated by less than their own diameter; PME separated by 2.8 × their own diameter; PME and PLE separated by slightly more than diameter of PME, PME positioned slightly posterior to level of PLE. Maxillae with field of small cuspules confined to inner corner; labium without cuspules. Abdomen (Figs 58, 63) broadly oval, light beige-brown in dorsal view with lateral sclerotic strips, dorso-lateral corrugations and scattered dorsal sclerotic spots. Dorsal surface of abdomen (Fig. 58) more heavily setose anteriorly, with assortment of stiff, porrect black setae, each with slightly raised, dark brown sclerotic base. Posterior abdomen strongly sigillate (Figs 58, 63); SP2 sclerites small oval spots; SP3 sclerites very large and circular; SP4 sclerites longitudinally elongate-oval; SP5 obscured. Legs (Figs 64–66) variable shades of dark brown, with light scopulae on tarsi I–II; distal tibia I with pair of large prolateral clasping spurs oriented longitudinally. Leg I: femur 7.2; patella 3.5; tibia 4.7; metatarsus 5.0; tarsus 3.4; total 23.8. Leg I femur–tarsus/carapace length ratio 3.0. Pedipalpal tibia (Figs 67–69) 2.5 × longer than wide; RTA burr-like, with conical basal protuberance and field of retroventral spinules; digital process porrect, unmodified. Cymbium (Figs 67–69) setose, with field of spinules disto-dorsally. Embolus (Figs 67–69) broadly twisted and sharply tapering distally, with prominent longitudinal flange and triangular (sub-distal) embolic apophysis.

#### Description (female AMS KS6393).

**Figures 70–78.**
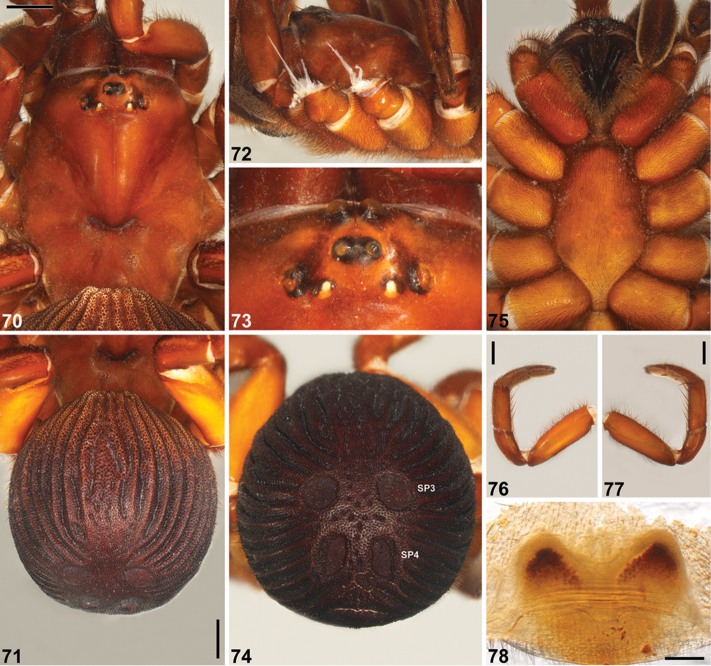
*Idiosoma arenaceum* sp. n., female (AMS KS6393) from Geraldton (Western Australia; GES). **70–71** Carapace and abdomen, dorsal view **72** Carapace, lateral view **73** Eyes, dorsal view **74** Abdomen, posterior view **75** Sternum, ventral view **76** Leg I, prolateral view **77** Leg I, retrolateral view **78** Spermathecae, dorsal view. Scale bars 2.0 (**70–71, 76–77**), 0.5 (**78**). SP3–4 = sigilla pairs 3–4.

Total length 24.8. Carapace 10.9 long, 8.1 wide. Abdomen 11.6 long, 10.8 wide. Carapace (Fig. 70) dark tan and chocolate-brown, with darker ocular region; fovea procurved. Eye group (Fig. 73) trapezoidal (anterior eye row strongly procurved), 0.6 × as long as wide, PLE–PLE/ALE–ALE ratio 2.6; ALE almost contiguous; AME separated by approximately their own diameter; PME separated by 5.0 × their own diameter; PME and PLE separated by more than diameter of PME, PME positioned in line with level of PLE. Maxillae with field of cuspules confined to inner corner (Fig. 75); labium without cuspules. Abdomen (Figs 71, 74) dark maroon-brown, corrugate and highly sclerotised, with leathery appearance typical of those species in the ‘sigillate complex’ (see Fig. 25). Posterior face of abdomen (Fig. 74, Key pane 21.1) with truncate ‘shield-like’ morphology; SP3 sclerites very large and circular; SP4 sclerites longitudinally elongate-oval; SP5 obscured by thickened cuticle. Legs (Figs 76–77) variable shades of dark tan; scopulae present on tarsi and metatarsi I–II; tibia I with one stout pro-distal macroseta and row of five longer retroventral macrosetae; metatarsus I with six stout macrosetae (at least one broken off at base); tarsus I with distal cluster of short macrosetae. Leg I: femur 6.8; patella 4.3; tibia 3.9; metatarsus 3.3; tarsus 2.3; total 20.7. Leg I femur–tarsus/carapace length ratio 1.9. Pedipalp dark tan, spinose on tibia and tarsus, with thick tarsal scopula. Genitalia (Fig. 78) with pair of short, subtriangular spermathecae, each bearing dense field of glandular vesicles distally and more sparsely distributed glandular field sub-distally.

#### Distribution and remarks.

*Idiosoma arenaceum* (formerly known by WAM identification code ‘MYG478’) (Figs 11–12) has a moderately widespread distribution in the Geraldton Sandplains and far northern Wheatbelt bioregions of south-western Western Australia, from near Yandanooka, Canna, and Geraldton north to Zuytdorp (Fig. 374). It is closely related to the three other ‘sigillate complex’ species in the northern *clypeatum*-clade: *I. clypeatum*, *I. kopejtkaorum*, and *I. kwongan* (Fig. 25). Burrows are adorned with a ‘moustache-like’ arrangement of twig-lines, sometimes under *Casuarina* (Figs 19, 20), and male specimens have been collected wandering in search of females in late autumn and winter, with an outlying record from January.

#### Conservation assessment.

*Idiosoma arenaceum* has a known extent of occurrence (EOO) of nearly 12,000 km^2^ [11,939 km^2^; with coastline as western margin], although this value is possibly an underestimate due to fairly limited survey effort. The area of occupancy within that range is similarly difficult to estimate, although is likely to be small as a proportion of EOO due to the scale of land clearing throughout most of its range. We do not currently consider this species to be of conservation concern, although further close assessment under both Criteria A and B is warranted in the future.

### Idiosoma clypeatum

Taxon classificationAnimaliaAraneaeIdiopidae

Rix & Harvey sp. n.

http://zoobank.org/86DFA140-6AB2-4FFE-B24E-D7F5B7AEE3D6

Figs 14 , 15 , 25 , 79–88 , 89–91 , 92–100 , 374

Idiosoma ‘*nigrum*’ Main, 1957b: 440 (in part; cited specimens from E. of Ederga, N. of Gullewa, S. of Mullewa and E./SE. of Paynes Find). Ellis 2015: 242, 244, figs 1, 3, 4.

#### Type material.

Holotype male. Albion Downs, 70.1 km NNW. of Leinster (IBRA_MUR), Western Australia, Australia, 27°21'30"S, 120°21'33"E, dry pitfall trap, 28 August–3 September 2008, Z. Hamilton, R. Teale (WAM T96452^DNA_Voucher_126^).

Paratype. 1 ♂, same data as holotype (WAM T96467).

#### Other material examined.

**AUSTRALIA: *Western Australia***: 1 ♂, Albion Downs, 62.6 km NNW. of Leinster (IBRA_MUR), 27°25'03"S, 120°23'45"E, dry pitfall trap, 28 August–3 September 2008, Z. Hamilton, R. Teale (WAM T96463); 1 ♂, same data (WAM T96510); 1 ♀, Blue Hill Range [not Blue Hills] (IBRA_YAL), 29°08'39"S, 116°53'04"E, burrow excavation, 14 October 2011, P.R. Langlands (WAM T117996^DNA_Voucher_125^); 1 juvenile, same data (WAM T117995); 1 juvenile, same data except 29°08'07"S, 116°52'49"E (WAM T117997); 1 juvenile, same locality data except 29°08'27"S, 116°52'39"E, 25 June 2010, L. Quinn, S. White (WAM T107333); 1 ♀, same locality data except 29°07'15"S, 116°45'18"E, 26–30 October 2012, S. White, F. Bokhari (WAM T127535); 1 ♀, same data except 29°07'29"S, 116°47'41"E (WAM T127536); 1 ♀, same data except 29°07'08"S, 116°46'56"E (WAM T127534); 1 ♀, Boolardy Station (IBRA_MUR), 27°03'46"S, 116°41'43"E, burrow excavation, 8 December 2014, A. Leung (WAM T136250^DNA_Voucher_NCB_021^); 1 ♂, Browns Soak, 20 miles N. of Lake Barlee on Youanmi-Pidgeon Rock Road (IBRA_MUR), ca. 29°00'S, 118°47'E, July–August 1957, W.H. Butler (WAM T139501); 1 ♀, ca. 16 km NE. of Coolcalalaya Homestead, Dampier- Bunbury Natural Gas Pipeline, site 6.07 (IBRA_YAL), 27°25'57"S, 115°11'02"E, in pipeline trench, 4 June 2006, P. Cullen (WAM T78246^DNA_Voucher_64^); 1 ♀, same data except ca. 21 km NE. of Coolcalalaya Homestead, 27°22'23"S, 115°11'07"E, 1 June 2006 (WAM T78252^DNA_Voucher_137^); 1 ♀, Dalgaranga Station (IBRA_MUR), 27°50'40"S, 117°08'14"E, 13 January 2010, M. Davis (WAM T108517^DNA_Voucher_140^); 1 ♀, 4 miles E. of Ederga (IBRA_YAL), 28°31'S, 116°28'E, 14 July 1955, B.Y. Main (WAM T144831); 1 ♀, same data (WAM T144802); 1 juvenile, same data (WAM T144832); 1 juvenile, same data (WAM T144833); 1 ♀, same data (WAM T144834); 1 ♂, Glen Station (IBRA_MUR), 26°34'23"S, 117°37'30"E, excavation, 10 May 2009, Ecologia (WAM T107368^DNA_Voucher_132^); 2 ♂, Glen Station, off Kalli Road (IBRA_MUR), 26°34'23"S, 117°37'30"E, wet pitfall trap, 5 May–28 June 2009, N. Dight, L. Quinn (WAM T98142); 1 ♀, 46 miles NE. of Gutha, 8 miles N. of Mullewa (IBRA_YAL), 28°35'S, 116°24'E, 18 August 1953, B.Y. Main (WAM T144781); 1 ♂, Jack Hills, ca. 1 km SW. of Mount Hale (IBRA_MUR), 26°02'53"S, 117°15'13"E, burrow excavation, 24 May 2012, P. Langlands (WAM T136251); 1 ♂, same data (WAM T136252^DNA_Voucher_NCB_022^); 1 juvenile, same data except 18 May 2012 (WAM T136247); 1 juvenile, same data (WAM T136248); 1 juvenile, same data (WAM T136249); 1 ♂, Jack Hills, site 17 (IBRA_MUR), 26°07'S, 117°09'E, 24 August–24 November 2006, Ecologia (WAM T139504); 1 ♀, Jack Hills, Creek Road, site 91, 18 October 2007, Ecologia staff (WAM T144762); 1 ♀, same data except site 79, 22 October 2007 (WAM T144761); 1 juvenile, Kadji Kadji Nature Reserve, 35 km NE. of Morawa (IBRA_ AVW), 29°15'36"S, 116°22'51"E, mammal pitfall, 6 August 2010, M. Bamford (WAM T110279^DNA_Voucher_142^); 1 ♀, Karara (IBRA_YAL), 29°10'42"S, 116°41'33"E, 12 January 2010, B. Durrant (WAM T108516^DNA_Voucher_141^); 1 ♀, Karara Station, ca. 21 km NE. of homestead (IBRA_YAL), 29°09'03"S, 116°54'24"E, mixed shrubland, *Acacia*/mallee, 10 October 2006, M. Bamford et al. (WAM T132582); 1 ♀, Mt Karara, quadrat 6.4 (IBRA_YAL), 29°10'50"S, 116°46'44"E, 29 June 2008, M. Bamford (WAM T91625); 1 ♀, Kirkalocka MMS Gold Mine (IBRA_MUR), 28°41'36"S, 117°46'56"E, 2–6 September 2011, S. White, M.C. Leng (WAM T116660); 1 juvenile, same data except 28°40'53"S, 117°45'04"E (WAM T116661); 1 ♀, Lakeside Station (IBRA_MUR), 27°37'11"S, 117°25'55"E, 12 January 2010, M. Davis (WAM T108030^DNA_Voucher_139^); 1 juvenile, 2 miles E. of Paynes Find (IBRA_MUR), 29°15'S, 117°48'E, 4 October 1955, B.Y. Main (WAM T144818); 1 ♀, 1 juvenile, same data (WAM T144819); 1 juvenile, same data (WAM T144820); 1 juvenile, same data (WAM T144821); 1 ♀, 10 juveniles, same data (WAM T144822); 1 ♀, same data (WAM T144823); 1 juvenile, same data (WAM T144824); 1 juvenile, same data (WAM T144825); 1 ♀, 24 miles SW. of Paynes Find on Great Northern Highway (IBRA_YAL), 29°21'S, 117°31'E, 4 November 1955, B.Y. Main (WAM T144798); 1 ♂, Urawa Nature Reserve, north, site ML7 (IBRA_YAL), 28°24'02"S, 115°34'34"E, wet pitfall traps, 15 September 1998–18 October 1999, B. Durrant, CALM Survey (WAM T139505); 1 ♂, Weld Range [no specific details] (IBRA_MUR), Ecologia (WAM T139581); 1 ♀, same locality data, 2 October 2007, B.Y. Main (WAM T144759); 1 ♀, same data (WAM T144760); 1 ♀, same locality data except 26°53'17"S, 117°43'05"E, 30 May 2010, J.D. Clark (WAM T136484); 2 ♂, Weld Range North, site WN1 (IBRA_MUR), 26°56'S, 117°38'E, 20–23 August 2007, Ecologia (WAM T139508); 1 ♂, same data except site WN2 (WAM T139509); 3 ♂, same data except site WN5 (WAM T139500); 1 ♂, same data except site WN6 (WAM T139507); 1 ♂, same data except site WN8 (WAM T139506); 1 juvenile, same data except site WN10, 26°47'47"S, 117°53'19"E, 3 September 2007 (WAM T144758); 1 ♂, same data except site WN11 (WAM T139503); 1 ♂, same data except site WN12 (WAM T139502); 1 ♀, Woolgorong (IBRA_YAL), 27°36'29"S, 116°42'42"E, 15 January 2010, M. Davis (WAM T108032^DNA_Voucher_138^); 1 ♀, Yakabindie (IBRA_MUR), 27°24'02"S, 120°41'29"E, targeted search, 21 January 2011, P. Bolton (WAM T110576^DNA_Voucher_63^); 1 ♀, same data except 27°17'27"S, 120°36'35"E (WAM T110581^DNA_Voucher_136^); 1 ♀, same data except 27°21'41"S, 120°40'59"E, 18 January 2011 (WAM T110579); 1 ♀, same data (WAM T110582); 1 ♀, same data (WAM T110575); 1 ♀, same data except 27°26'16"S, 120°36'35"E, 14 January 2011 (WAM T110580); 1 ♀, same data (WAM T110578); 1 ♀, 12 juveniles, same data (WAM T110577); 1 ♀, Yeelirrie, ca. 66.70 km SW. of Wiluna Airport (IBRA_MUR), 27°02'06"S, 119°42'55"E, hand collected, 16 March 2015, M. Bamford (WAM T135961); 1 ♀, same data except 27°02'07"S, 119°43'00"E (WAM T135962); 1 ♀, Yeelirrie Station (IBRA_MUR), 27°19'02"S, 120°12'18"E, excavation, 28 January–4 February 2010, Ecologia (WAM T101942^DNA_Voucher_160^); 1 ♀, same data except 27°08'15"S, 119°53'20"E, 11 September 2010, Ecologia Staff (WAM T103475^DNA_Voucher_135^).

#### Etymology.

The specific epithet is derived from the Latin *clypeatus* (adjective: ‘shielded’, ‘armed with a shield’), in reference to the highly sclerotised, phragmotic abdominal morphology of this species.

#### Diagnosis.

*Idiosoma clypeatum* is one of seven highly autapomorphic species in the polyphyletic ‘sigillate complex’ (Fig. 25); members of this complex can be distinguished from all other species in the *nigrum*-group from south-western Australia (i.e., *I. formosum*, *I. gardneri*, *I. gutharuka*, *I. incomptum*, *I. intermedium*, *I. jarrah*, *I. mcclementsorum*, *I. mcnamarai* and *I. sigillatum*) by the presence of well-defined lateral sclerotic strips on the male abdomen (e.g., Figs 32, 63, 256), and by the very heavily sclerotised, leathery, ‘shield-like’ morphology of the female abdomen (e.g., Figs 1–3, 9–12, 52, 74, 96). Males of *I. clypeatum* can be further distinguished from those of *I. arenaceum* by the shape of the SP4 sclerites, which are not elongate-oval (Fig. 85; cf. Fig. 63); from *I. kwongan* by the absence of semi-circular lateral indentations adjacent to the SP4 sclerites (Fig. 85; cf. Fig. 278, Key pane 13.1); from *I. dandaragan*, *I. nigrum* and *I. schoknechtorum* by the absence of a prominent sub-distal embolic apophysis (Key pane 14.3; cf. Key pane 14.1); and from *I. kopejtkaorum* by the more heavily setose morphology of metatarsus I (Fig. 86, Key pane 16.1; cf. Fig. 257, Key pane 16.2).

Females can be distinguished from those of *I. arenaceum* by the shape of the SP4 sclerites, which are not elongate-oval (Fig. 96, Key pane 22.2; cf. Fig. 74, Key pane 21.1); and from *I. dandaragan*, *I. nigrum* and *I. schoknechtorum* by the size of the SP4 sclerites, which are approximately half the size of the SP3 sclerites (Fig. 96, Key pane 22.2; cf. Figs 43, 52, 140, 346, Key panes 23.1–23.3) [NB. females of *I. kwongan* are unknown]. By our assessment, females of *I. clypeatum* are morphologically indistinguishable from those of *I. kopejtkaorum*; males, molecular data (Fig. 25) or geographic distribution (Fig. 374) are required for accurate identification.

This species can also be distinguished from *I. corrugatum* (from the Eyre Peninsula of South Australia) by the shape of the prolateral clasping spurs on the male tibia I, which are oriented longitudinally (Fig. 86; cf. Fig. 109), and by the shape of the female eye group, which is broadly trapezoidal (Fig. 95; cf. Fig. 117).

#### Description (male holotype).

**Figures 79–88.**
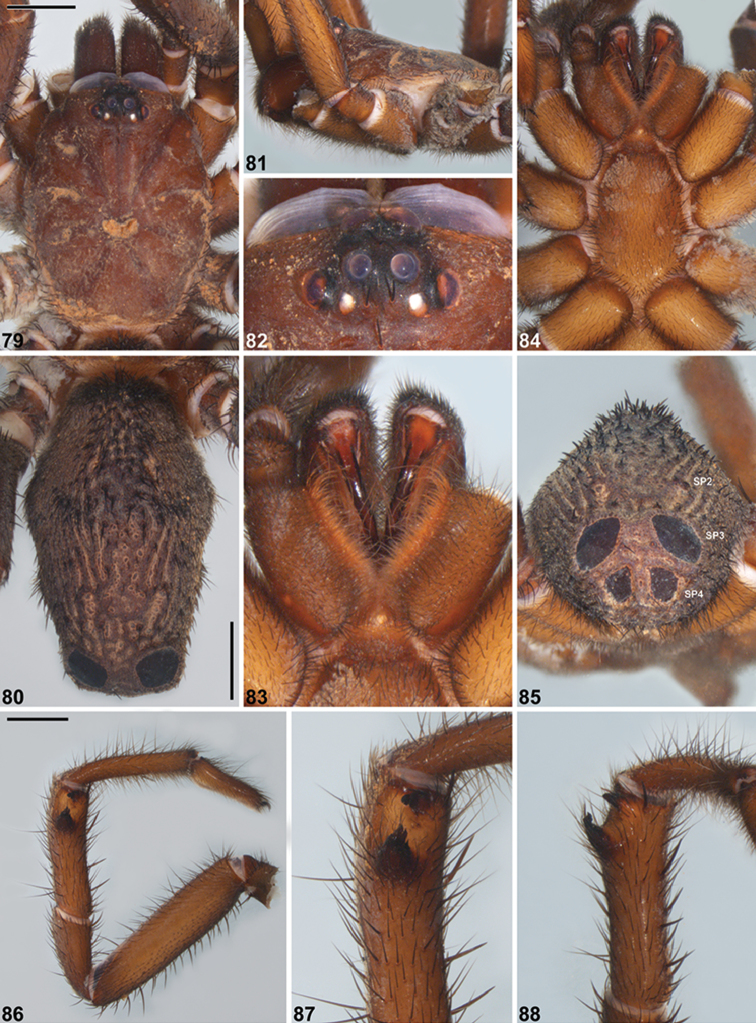
*Idiosoma clypeatum* sp. n., male holotype (WAM T96452) from Albion Downs (Western Australia; MUR), somatic morphology. **79–80** Carapace and abdomen, dorsal view **81** Carapace, lateral view **82** Eyes, dorsal view **83** Mouthparts, ventral view **84** Sternum, ventral view **85** Abdomen, posterior view **86** Leg I, prolateral view **87** Leg I tibia, clasping spurs, prolateral view **88** Leg I tibia, proventral view. Scale bars 2.0. SP2–4 = sigilla pairs 2–4.

**Figures 89–91.**
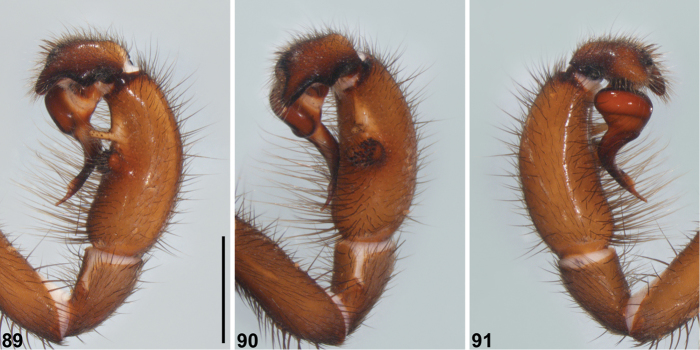
*Idiosoma clypeatum* sp. n., male holotype (WAM T96452) from Albion Downs (Western Australia; MUR), pedipalp. **89** Retrolateral view **90** Retroventral view **91** Prolateral view. Scale bar 2.0.

Total length 17.3. Carapace 7.4 long, 5.3 wide. Abdomen 8.2 long, 4.9 wide. Carapace (Fig. 79) dark chocolate-brown, with darker ocular region; lateral margins with uniformly-spaced fringe of porrect black setae; fovea procurved. Eye group (Fig. 82) trapezoidal (anterior eye row strongly procurved), 0.6 × as long as wide, PLE–PLE/ALE–ALE ratio 2.0; ALE almost contiguous; AME separated by less than their own diameter; PME separated by 4.0 × their own diameter; PME and PLE separated by slightly more than diameter of PME, PME positioned slightly posterior to level of PLE. Maxillae and labium without cuspules. Abdomen (Figs 80, 85) irregularly oval, dark brown in dorsal view with lateral sclerotic strips, dorso-lateral corrugations, and scattered dorsal sclerotic spots. Dorsal surface of abdomen (Fig. 80) more heavily setose anteriorly, with assortment of stiff, porrect black setae, each with slightly raised, dark brown sclerotic base. Posterior abdomen strongly sigillate (Figs 80, 85); SP2 sclerites irregular spots; SP3 sclerites very large and circular; SP4 sclerites broadly oval, almost subquadrate; SP5 obscured. Legs (Figs 86–88) variable shades of dark tan, with light scopulae on tarsi I–II; distal tibia I with pair of large prolateral clasping spurs oriented longitudinally. Leg I: femur 6.3; patella 3.3; tibia 4.2; metatarsus 4.7; tarsus 2.5; total 21.1. Leg I femur–tarsus/carapace length ratio 2.9. Pedipalpal tibia (Figs 89–91) 2.4 × longer than wide; RTA burr-like, with conical basal protuberance and field of retroventral spinules; digital process porrect, unmodified. Cymbium (Figs 89–91) setose, with field of spinules disto-dorsally. Embolus (Figs 89–91) broadly twisted and sharply tapering distally, with prominent longitudinal flange; embolic apophysis absent.

#### Description (female WAM T108516).

**Figures 92–100.**
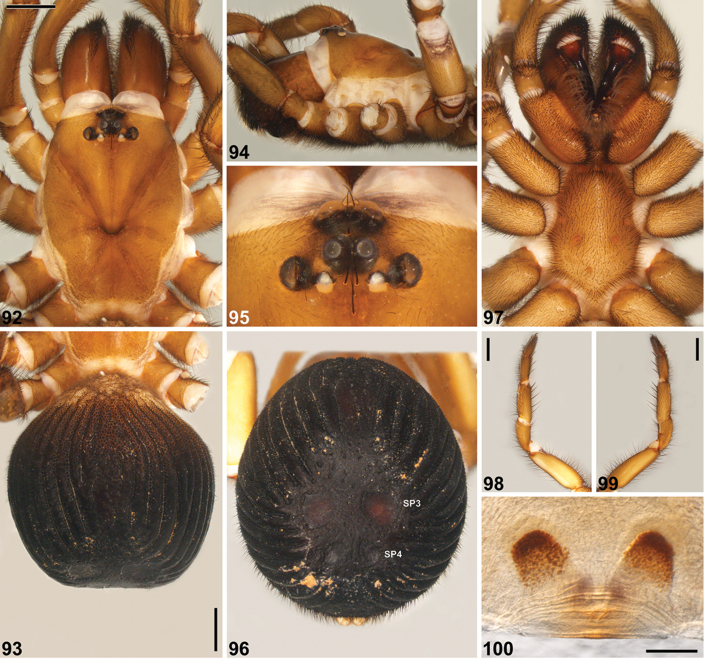
*Idiosoma clypeatum* sp. n., female (WAM T108516) from Karara (Western Australia; YAL). **92–93** Carapace and abdomen, dorsal view **94** Carapace, lateral view **95** Eyes, dorsal view **96** Abdomen, posterior view **97** Sternum, ventral view **98** Leg I, prolateral view **99** Leg I, retrolateral view **100** Spermathecae, dorsal view. Scale bars 2.0 (**92–93, 98–99**), 0.5 (**100**). SP3–4 = sigilla pairs 3–4.

Total length 21.8. Carapace 8.3 long, 5.7 wide. Abdomen 9.9 long, 9.6 wide. Carapace (Fig. 92) tan, with darker ocular region; fovea procurved. Eye group (Fig. 95) trapezoidal (anterior eye row strongly procurved), 0.6 × as long as wide, PLE–PLE/ALE–ALE ratio 2.2; ALE almost contiguous; AME separated by approximately their own diameter; PME separated by 2.1 × their own diameter; PME and PLE separated by more than diameter of PME, PME positioned in line with level of PLE. Maxillae with field of cuspules confined to inner corner (Fig. 97); labium without cuspules. Abdomen (Figs 93, 96) dark brown-black, corrugate and highly sclerotised, with leathery appearance typical of those species in the ‘sigillate complex’ (see Fig. 25). Posterior face of abdomen (Fig. 96, Key pane 22.2) with truncate ‘shield-like’ morphology; SP3 sclerites very large and circular; SP4 sclerites oval; SP5 obscured by thickened cuticle. Legs (Figs 98–99) variable shades of tan; scopulae present on tarsi and metatarsi I–II; tibia I with one stout pro-distal macroseta and row of five longer retroventral macrosetae; metatarsus I with seven stout macrosetae; tarsus I with distal cluster of short macrosetae. Leg I: femur 5.0; patella 3.3; tibia 3.0; metatarsus 2.5; tarsus 2.0; total 15.8. Leg I femur–tarsus/carapace length ratio 1.9. Pedipalp tan, spinose on tibia and tarsus, with thick tarsal scopula. Genitalia (Fig. 100) with pair of short, obliquely angled spermathecae, each bearing dense field of glandular vesicles distally, and more sparsely distributed glandular field sub-distally.

#### Distribution and remarks.

*Idiosoma clypeatum* (formerly known by WAM identification code ‘MYG018’) has a widespread distribution in Western Australia’s inland arid zone, principally throughout the Yalgoo and Murchison bioregions where it is the only known species in the *nigrum*-group (excluding a population of *I. formosum* from the southern Yalgoo) (Fig. 374). It extends from near Paynes Find, the Blue Hill Range, Kadji Kadji Nature Reserve, and Karara in the south, north and north-east to at least Coolcalalaya Homestead, Jack Hills, Albion Downs, Yakabindie, and Yeelirrie. This distribution seems to be strongly correlated with annual rainfall of less than 250 mm. At the southern extent of its range it abuts the northern limit of the closely related species *I. kopejtkaorum*, and on the Geraldton Sandplains is replaced by *I. arenaceum* and *I. kwongan* (all four ‘sigillate complex’ species together forming the northern *clypeatum*-clade; Fig. 25).

*Idiosoma clypeatum* was for a long time misidentified as *I. nigrum*, and indeed the 2013 threatened species assessment of *I. nigrum* prepared under the Commonwealth *Environmental Protection and Biodiversity Conservation Act 1999* conflated the identification of these two species. Burrows are adorned with a ‘moustache-like’ arrangement of twig-lines (Figs 14, 15), and males have been collected wandering in search of females in late autumn, winter and spring, with a peak of activity in winter. Ellis (2015) summarises aspects of the biology of this species based on observations at the Weld Range.

#### Conservation assessment.

In 2017, *Idiosoma clypeatum* was formally assessed as ‘priority 3’ fauna; this assessment incorporated the latest taxonomic, geographic, and genetic data summarised in the current study (with a number of additional records also identified subsequently). It has a known extent of occurrence of over 120,000 km^2^ [120,465 km^2^], and while it therefore cannot be considered threatened under Criterion B, a ‘priority 3’ recommendation was made due to the widespread occurrence of this species in areas prospective for mining and mineral resources. Further close assessment under both Criteria A and B is warranted in the future.

### Idiosoma corrugatum

Taxon classificationAnimaliaAraneaeIdiopidae

Rix & Harvey sp. n.

http://zoobank.org/D697B9D6-152F-427E-870E-9ABD471C9B8D

Figs 101–110 , 111–113 , 114–122 , 377

#### Type material.

Holotype male. Eyre Peninsula, 54 miles SW. of Kimba (IBRA_EYB), South Australia, Australia, ca. 33°42'S, 136°18'E [NB. precise locality unknown], 2 December 1959, B.Y. Main (SAM NN29858).

Paratypes. 1 ♀, same data as holotype (WAM T144751); 1 ♀, same data (WAM T144752); 1 juvenile, same data (WAM T144753); 1 ♀, same data (WAM T144754); 1 ♀, same data (WAM T144755); 1 ♀, same data (WAM T144748); 1 ♀, same data (WAM T144747); 1 ♀, same data (WAM T144749); 1 ♀, same data (WAM T144750); 1 juvenile, same data (SAM NN29859); 1 ♀, same data as holotype except ca. 11 miles NW. of Cleve (NB. precise locality unknown) (WAM T144636); 1 ♀, same data (SAM NN29860); 1 juvenile, same data (WAM T144630); 1 ♀, same data (WAM T144635); 1 ♀, same data (WAM T144629); 1 ♀, same data (WAM T144746); 1 ♀, same data (WAM T144631); 1 ♀, same data (WAM T144632); 1 ♀, same data (WAM T144634).

#### Etymology.

The specific epithet is derived from the Latin *corrugatus* (adjective: ‘ridged’; see Brown 1956), in reference to the corrugate abdominal morphology of this species. This name was first coined informally by Barbara Main, and we reproduce it here in recognition of her discovery of this species.

#### Diagnosis.

Males and females of *Idiosoma corrugatum* can be distinguished from all other species of *Idiosoma* in the *nigrum*-group (i.e., *I. arenaceum*, *I. clypeatum*, *I. dandaragan*, *I. formosum*, *I. gardneri*, *I. gutharuka*, *I. incomptum*, *I. intermedium*, *I. jarrah*, *I. kopejtkaorum*, *I. kwongan*, *I. mcclementsorum*, *I. mcnamarai*, *I. nigrum*, *I. schoknechtorum* and *I. sigillatum*) by the shape of the eye group, which is compact and not broadly trapezoidal (Figs 104, 117). Males can also be distinguished by the shape of the prolateral clasping spurs on tibia I, which are oriented dorso-ventrally (Fig. 109).

#### Description (male holotype).

**Figures 101–110.**
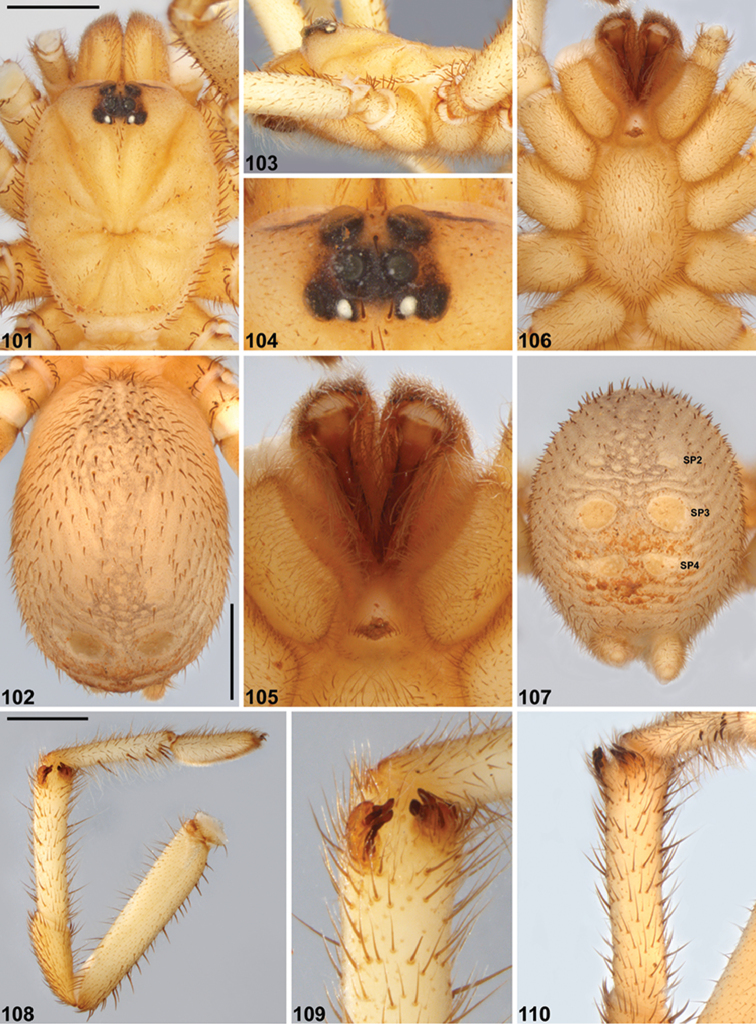
*Idiosoma corrugatum* sp. n., male holotype (SAM NN29858) from SW. of Kimba (South Australia; EYB), somatic morphology. **101–102** Carapace and abdomen, dorsal view **103** Carapace, lateral view **104** Eyes, dorsal view **105** Mouthparts, ventral view **106** Sternum, ventral view **107** Abdomen, posterior view **108** Leg I, prolateral view **109** Leg I tibia, clasping spurs, prolateral view **110** Leg I tibia, proventral view. Scale bars 2.0. SP2–4 = sigilla pairs 2–4.

**Figures 111–113.**
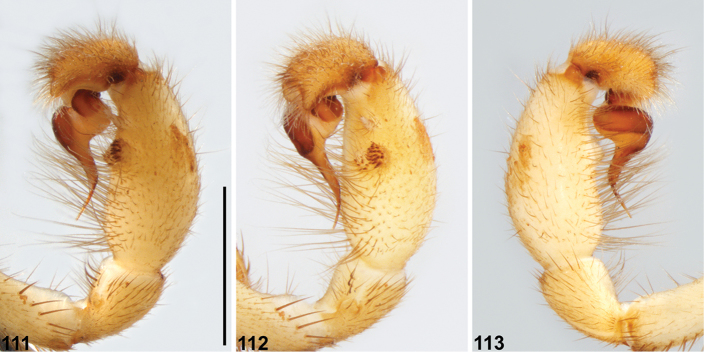
*Idiosoma corrugatum* sp. n., male holotype (SAM NN29858) from SW. of Kimba (South Australia; EYB), pedipalp. **111** Retrolateral view **112** Retroventral view **113** Prolateral view. Scale bar 2.0.

Total length 13.7. Carapace 5.5 long, 4.3 wide. Abdomen 6.7 long, 4.5 wide. Carapace (Fig. 101) pale (faded) tan, with darker ocular region; lateral margins with uniformly-spaced fringe of porrect black setae; fovea procurved. Eye group (Fig. 104) trapezoidal (anterior eye row strongly procurved), 0.8 × as long as wide, PLE–PLE/ALE–ALE ratio 1.3; ALE separated by approximately half their own diameter; AME separated by less than their own diameter; PME separated by 2.9 × their own diameter; PME and PLE separated by approximately diameter of PME, PME positioned in line with level of PLE. Maxillae and labium without cuspules. Abdomen (Figs 102, 107) oval, pale (faded) tan in dorsal view with dorso-lateral corrugations and scattered dorsal sclerotic spots. Dorsal surface of abdomen (Fig. 102) with assortment of stiff, porrect black setae, each with slightly raised sclerotic base. Posterior abdomen sigillate (Figs 102, 107); SP2 sclerites comma-shaped spots; SP3 sclerites large and circular; SP4 sclerites oval, each surrounded by chevron-like pad of unsclerotised cuticle laterally; SP5 obscured. Legs (Figs 108–110) variable shades of pale (faded) tan, with light scopulae on tarsi I–II; distal tibia I with pair of large prolateral clasping spurs oriented dorso-ventrally. Leg I: femur 5.2; patella 2.6; tibia 3.9; metatarsus 3.3; tarsus 2.0; total 17.0. Leg I femur–tarsus/carapace length ratio 3.1. Pedipalpal tibia (Figs 111–113) 2.2 × longer than wide; RTA burr-like, with conical basal protuberance and field of retroventral spinules; digital process porrect, unmodified. Cymbium (Figs 111–113) setose, with field of spinules disto-dorsally. Embolus (Figs 111–113) twisted and gradually tapering distally; embolic apophysis absent.

#### Description (female WAM T144634).

**Figures 114–122.**
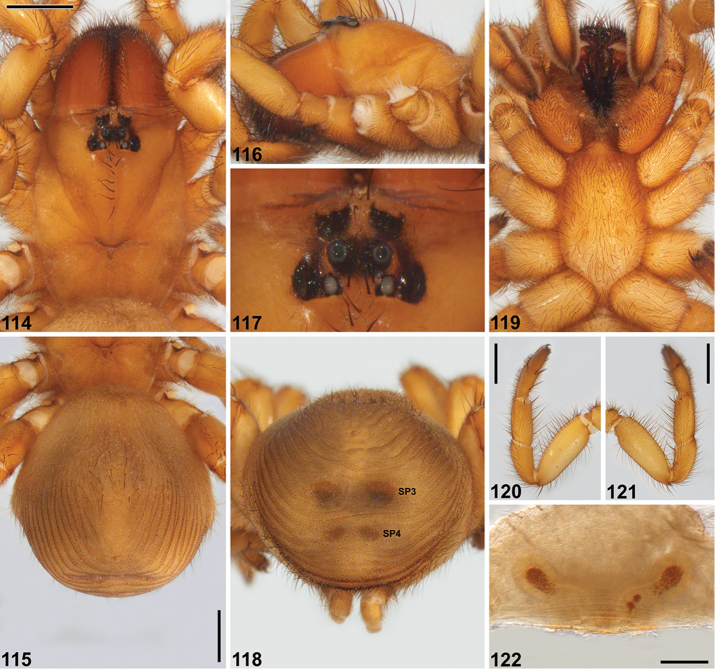
*Idiosoma corrugatum* sp. n., female (WAM T144634) from NW. of Cleve (South Australia; EYB). **114–115** Carapace and abdomen, dorsal view **116** Carapace, lateral view **117** Eyes, dorsal view **118** Abdomen, posterior view **119** Sternum, ventral view **120** Leg I, prolateral view **121** Leg I, retrolateral view **122** Spermathecae, dorsal view. Scale bars 2.0 (**114–115, 120–121**), 0.5 (**122**). SP3–4 = sigilla pairs 3–4.

Total length 17.0. Carapace 6.4 long, 4.8 wide. Abdomen 8.2 long, 7.9 wide. Carapace (Fig. 114) faded tan, with darker ocular region; fovea procurved. Eye group (Fig. 117) trapezoidal (anterior eye row strongly procurved), 0.8 × as long as wide, PLE–PLE/ALE–ALE ratio 1.5; ALE separated by slightly more than half their own diameter; AME separated by more than their own diameter; PME separated by 3.0 × their own diameter; PME and PLE separated by approximately diameter of PME, PME positioned in line with level of PLE. Maxillae with field of cuspules confined to inner corner (Fig. 119); labium without cuspules. Abdomen (Figs 115, 118) faded tan, corrugate and highly sclerotised posteriorly. Posterior face of abdomen (Fig. 118) with truncate ‘shield-like’ morphology; SP3 sclerites large and circular; SP4 sclerites oval; SP5 obscured by thickened cuticle. Legs (Figs 120–121) variable shades of faded tan; scopulae present on tarsi and metatarsi I–II; tibia I with one stout pro-distal macroseta and row of three longer retroventral macrosetae; metatarsus I with six stout macrosetae; tarsus I with distal cluster of short macrosetae. Leg I: femur 3.7; patella 2.6; tibia 2.1; metatarsus 1.7; tarsus 1.3; total 11.4. Leg I femur–tarsus/carapace length ratio 1.8. Pedipalp faded tan, spinose on tibia and tarsus, with thick tarsal scopula. Genitalia (Fig. 122) with pair of widely spaced and obliquely angled spermathecae, each bearing dense field of glandular vesicles distally.

#### Distribution and remarks.

*Idiosoma corrugatum* has a highly restricted distribution in the central-eastern Eyre Peninsula of South Australia, where it is known from only one or possibly two indeterminate sites west of Cleve (both from the late 1950s) (Fig. 377). The type locality (“54 miles SW. of Kimba”) and the other known locality (“11 miles NW. of Cleve”) likely correspond to roughly the same site (or nearly so), although this is difficult to confirm given the collection data available. No other records of the species are known, and dedicated recent searching in the vicinity of Cleve has so far failed to detect any extant populations.

The highly disjunct distribution of *I. corrugatum* relative to other species is unique among shield-backed trapdoor spiders, and raises the question of whether it is actually a member of the *nigrum*-group at all. Convergent evolution of a phragmotic abdominal morphology has already been demonstrated in *I. galeosomoides* (see Rix et al. 2017b, d), and it remains possible that *I. corrugatum* has independently evolved a corrugate abdomen with enlarged SP3 and SP4 sclerites (a possibility further supported by the morphology of the eye group, the female spermathecae and the male leg I clasping spurs). Burrow morphology is undescribed, and it is unclear whether the single known male from December 1959 was collected from within its burrow or wandering in search of females.

#### Conservation assessment.

Due to the known occurrence of this species at only one (or possibly two; see above) sites, we consider it data deficient for the purposes of conservation assessment. However, the absence of recent records (despite extensive collections from the area), the scale of land clearing on the Eyre Peninsula, and the population declines that have occurred among Idiopidae since the 1950s (Rix et al. 2017c), would suggest that this species may be critically endangered or extinct. Assuming extant populations can eventually be detected, further close assessment under both Criteria A and B is warranted in the future.

### Idiosoma dandaragan

Taxon classificationAnimaliaAraneaeIdiopidae

Rix & Harvey sp. n.

http://zoobank.org/E6C8869C-9F37-47D3-B230-9EF5DD690187

Figs 25 , 123–132 , 133–135 , 136–144 , 374

Idiosoma ‘*nigrum*’ Main, 1957b: 440 (in part; cited specimens from S. of Moora and E. of Watheroo).

#### Type material.

Holotype male. 12.2 miles S. of Moora on Mogumber Road (IBRA_JAF), Western Australia, Australia, 30°49'S, 116°02'E, 25 May 1954, B.Y. Main (WAM T139522).

Paratypes. 1 ♂, same data as holotype (WAM T139523); 1 ♂, same data (WAM T139525); 1 ♂, same data (WAM T139526).

#### Other material examined.

**AUSTRALIA: *Western Australia***: 1 ♂, 5.6 miles S. of Moora on Mogumber Road (IBRA_AVW), 30°43'S, 116°01'E, 25 May 1954, B.Y. Main (WAM T139524); 1 ♂, 9.1 miles S. of Moora on Mogumber Road (IBRA_AVW), 30°46'S, 116°01'E, 25 May 1954, B.Y. Main (WAM T139521); 1 ♀, 12.9 km NE. of New Norcia (IBRA_AVW), 30°55'00"S, 116°19'54"E, soil rake, leaf litter, 13 September 2012, T. Sachse (WAM T127016); 1 ♀, same data except 30°54'58"S, 116°19'54"E (WAM T127017^DNA_Voucher_152^); 1 juvenile, Watheroo National Park (IBRA_GES), 30°15'17"S, 116°00'13"E, 7 January 2010, B. Durrant (WAM T108031^DNA_Voucher_148^); 1 ♀, 4 miles E. of Watheroo (IBRA_AVW), 30°18'S, 116°07'E, 11 July 1955, B.Y. Main (WAM T144827); 1 ♀, same data (WAM T144828); 1 ♀, same data (WAM T144829).

#### Etymology.

The specific epithet is a noun in apposition, in reference to the occurrence of this species on the Dandaragan Plateau, north of Perth.

#### Diagnosis.

*Idiosoma dandaragan* is one of seven highly autapomorphic species in the polyphyletic ‘sigillate complex’ (Fig. 25); members of this complex can be distinguished from all other species in the *nigrum*-group from south-western Australia (i.e., *I. formosum*, *I. gardneri*, *I. gutharuka*, *I. incomptum*, *I. intermedium*, *I. jarrah*, *I. mcclementsorum*, *I. mcnamarai* and *I. sigillatum*) by the presence of well-defined lateral sclerotic strips on the male abdomen (e.g., Figs 32, 63, 256), and by the very heavily sclerotised, leathery, ‘shield-like’ morphology of the female abdomen (e.g., Figs 1–3, 9–12, 52, 74, 96). Males of *I. dandaragan* can be further distinguished from those of *I. arenaceum* by the shape of the SP4 sclerites, which are not elongate-oval (Fig. 129; cf. Fig. 63); from *I. kwongan* by the absence of semi-circular lateral indentations adjacent to the SP4 sclerites (Fig. 129; cf. Fig. 278, Key pane 13.1); from *I. clypeatum* and *I. kopejtkaorum* by the presence of a prominent sub-distal embolic apophysis (Key pane 14.1; cf. Key panes 14.2, 14.3); and from *I. nigrum* by the more heavily setose morphology of the dorsal abdomen (Fig. 124; cf. Fig. 27), and by the shape of the SP4 sclerites, which are circular or oval (Fig. 129; cf. Fig. 32). By our assessment, males of *I. dandaragan* are morphologically indistinguishable from those of *I. schoknechtorum*; molecular data (Fig. 25) or geographic distribution (Fig. 374) are required for accurate identification.

Females can be distinguished from those of *I. arenaceum* by the shape of the SP4 sclerites, which are not elongate-oval (Fig. 140, Key pane 23.2; cf. Fig. 74, Key pane 21.1); from *I. clypeatum* and *I. kopejtkaorum* by the size of the SP4 sclerites, which are greater than half the size of the SP3 sclerites (Fig. 140, Key pane 23.2; cf. Figs 96, 267, Key panes 22.1, 22.2); and from *I. nigrum* by the shape of the SP4 sclerites, which are circular or broadly oval (Fig. 140, Key pane 23.2; cf. Figs 43, 52, Key pane 23.1), and by the presence of well-defined SP5 sclerites (Fig. 140, Key pane 23.2; cf. Figs 43, 52, Key pane 23.1) [NB. females of *I. kwongan* are unknown]. By our assessment, females of *I. dandaragan* are morphologically indistinguishable from those of *I. schoknechtorum*; molecular data (Fig. 25) or geographic distribution (Fig. 374) are required for accurate identification.

This species can also be distinguished from *I. corrugatum* (from the Eyre Peninsula of South Australia) by the shape of the prolateral clasping spurs on the male tibia I, which are oriented longitudinally (Fig. 131; cf. Fig. 109), and by the shape of the female eye group, which is broadly trapezoidal (Fig. 139; cf. Fig. 117).

#### Description (male holotype).

**Figures 123–132.**
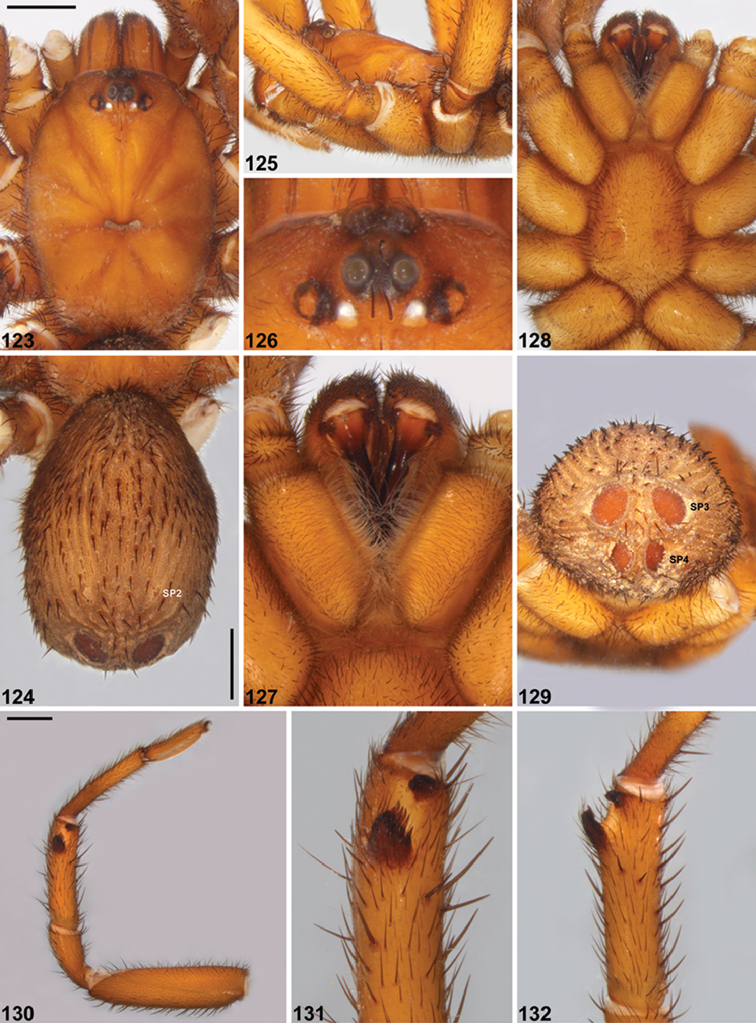
*Idiosoma dandaragan* sp. n., male holotype (WAM T139522) from S. of Moora (Western Australia; JAF), somatic morphology. **123–124** Carapace and abdomen, dorsal view **125** Carapace, lateral view **126** Eyes, dorsal view **127** Mouthparts, ventral view **128** Sternum, ventral view **129** Abdomen, posterior view **130** Leg I, prolateral view **131** Leg I tibia, clasping spurs, prolateral view **132** Leg I tibia, proventral view. Scale bars 2.0. SP2–4 = sigilla pairs 2–4.

**Figures 133–135.**
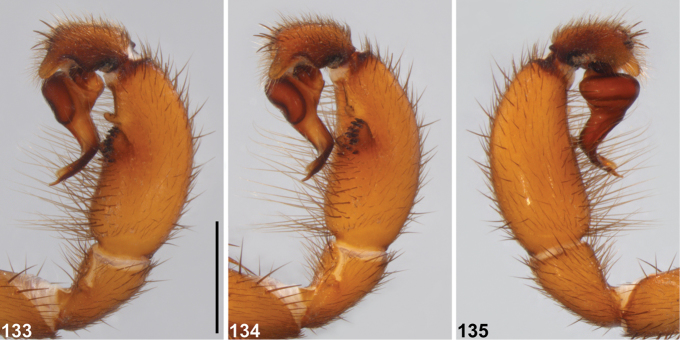
*Idiosoma dandaragan* sp. n., male holotype (WAM T139522) from S. of Moora (Western Australia; JAF), pedipalp. **133** Retrolateral view **134** Retroventral view **135** Prolateral view. Scale bar 2.0.

Total length 17.5. Carapace 8.0 long, 5.7 wide. Abdomen 8.0 long, 5.6 wide. Carapace (Fig. 123) tan, with darker ocular region; lateral margins with uniformly-spaced fringe of porrect black setae; fovea procurved. Eye group (Fig. 126) trapezoidal (anterior eye row strongly procurved), 0.7 × as long as wide, PLE–PLE/ALE–ALE ratio 2.2; ALE almost contiguous; AME separated by less than their own diameter; PME separated by 2.2 × their own diameter; PME and PLE separated by slightly more than diameter of PME, PME positioned in line with level of PLE. Maxillae with field of small cuspules confined to inner corner; labium without cuspules. Abdomen (Figs 124, 129) oval, dark beige-brown in dorsal view with lateral sclerotic strips, dorso-lateral striations, and scattered dorsal sclerotic spots. Dorsal surface of abdomen (Fig. 124) more heavily setose anteriorly, with assortment of stiff, porrect black setae, each with slightly raised, dark brown sclerotic base. Posterior abdomen strongly sigillate (Figs 124, 129); SP2 sclerites irregular, comma-shaped spots; SP3 sclerites very large and circular; SP4 sclerites broadly oval; SP5 obscured. Legs (Figs 130–132) variable shades of tan, with light scopulae on tarsi I–II; distal tibia I with pair of large prolateral clasping spurs oriented longitudinally. Leg I: femur 6.9; patella 3.4; tibia 4.8; metatarsus 4.9; tarsus 2.9; total 22.9. Leg I femur–tarsus/carapace length ratio 2.9. Pedipalpal tibia (Figs 133–135) 2.3 × longer than wide; RTA burr-like, with conical basal protuberance and field of retroventral spinules; digital process porrect, unmodified. Cymbium (Figs 133–135) setose, with field of spinules disto-dorsally. Embolus (Figs 133–135) broadly twisted and sharply tapering distally, with prominent longitudinal flange and triangular (sub-distal) embolic apophysis.

#### Description (female WAM T127017).

**Figures 136–144.**
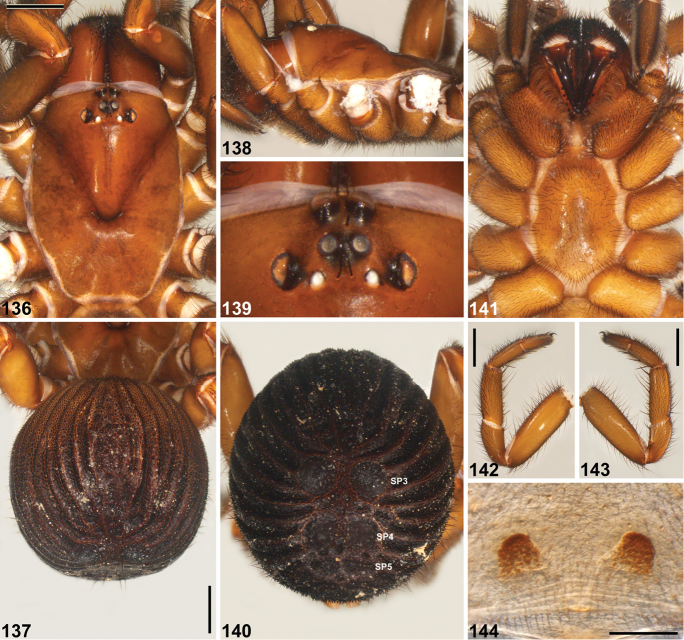
*Idiosoma dandaragan* sp. n., female (WAM T127017) from NE. of New Norcia (Western Australia; AVW). **136–137** Carapace and abdomen, dorsal view **138** Carapace, lateral view **139** Eyes, dorsal view **140** Abdomen, posterior view **141** Sternum, ventral view **142** Leg I, prolateral view **143** Leg I, retrolateral view **144** Spermathecae, dorsal view. Scale bars 2.0 (**136–137, 142–143**), 0.5 (**144**). SP3–5 = sigilla pairs 3–5.

Total length 18.6. Carapace 7.5 long, 5.3 wide. Abdomen 8.6 long, 8.3 wide. Carapace (Fig. 136) dark tan and chocolate-brown, with darker ocular region; fovea procurved. Eye group (Fig. 139) trapezoidal (anterior eye row strongly procurved), 0.7 × as long as wide, PLE–PLE/ALE–ALE ratio 2.3; ALE almost contiguous; AME separated by approximately their own diameter; PME separated by 2.8 × their own diameter; PME and PLE separated by more than diameter of PME, PME positioned in line with level of PLE. Maxillae with field of cuspules confined to inner corner (Fig. 141); labium without cuspules. Abdomen (Figs 137, 140) dark brown-black, corrugate and highly sclerotised, with leathery appearance typical of those species in the ‘sigillate complex’ (see Fig. 25). Posterior face of abdomen (Fig. 140, Key pane 23.2) with truncate ‘shield-like’ morphology; SP3 sclerites very large and circular; SP4 sclerites broadly oval; SP5 sclerites small, broadly oval (left) or irregularly-shaped (right). Legs (Figs 142–143) variable shades of dark tan; scopulae present on tarsi and metatarsi I–II; tibia I with one stout pro-distal macroseta (broken off at base) and row of five longer retroventral macrosetae; metatarsus I with eight stout macrosetae; tarsus I with distal cluster of short macrosetae. Leg I: femur 4.7; patella 2.8; tibia 3.0; metatarsus 2.2; tarsus 1.6; total 14.3. Leg I femur–tarsus/carapace length ratio 1.9. Pedipalp tan, spinose on tibia and tarsus, with thick tarsal scopula. Genitalia (Fig. 144) with pair of short, obliquely angled spermathecae, each bearing dense field of glandular vesicles distally, and more sparsely distributed glandular field sub-distally.

#### Distribution and remarks.

*Idiosoma dandaragan* (formerly known by WAM identification code ‘MYG477’), a ‘sigillate complex’ member of the diverse *sigillatum*-clade (Fig. 25), has a restricted distribution along the eastern margin of the Dandaragan Plateau, from near New Norcia in the south, north to at least the Watheroo National Park (Fig. 374). Its distribution closely abuts the north-western extent of the range of *I. nigrum* near New Norcia and Carani. Both *I. dandaragan* and *I. nigrum* are similar in having strongly sigillate (sclerotised) abdomens, although morphology and molecular data can be used to convincingly separate them. Little is known of the biology of this species, other than that males have been collected (wandering in search of females or waiting in their burrows) in late autumn.

#### Conservation assessment.

*Idiosoma dandaragan* has a known extent of occurrence of nearly 1,500 km^2^ [1,230 km^2^], and an area of occupancy within that range of < 500 km^2^. Given: (i) this geographic range; (ii) the sampling effort that has occurred in surrounding areas as a result of a major biotic survey (see Keighery 2004); (iii) the occurrence of the species at < 10 severely fragmented sites; and (iv) the continuing decline in the area, extent and/or quality of habitat in the western-central Wheatbelt agricultural zone (Laurance et al. 2011), this species is considered Endangered (B1ab[iii] + B2ab[iii]). Further close assessment under both Criteria A and B will be crucial to the continued survival of this species.

### Idiosoma formosum

Taxon classificationAnimaliaAraneaeIdiopidae

Rix & Harvey sp. n.

http://zoobank.org/E8212C7E-27BD-4242-8AD9-C32B89308895

Figs 25 , 145–154 , 155–157 , 158–166 , 375

#### Type material.

Holotype male. Mount Gibson Station, 93 km NE. of Wubin (IBRA_YAL), Western Australia, Australia, 29°41'57"S, 117°24'28"E, pitfall trap, 21–29 August 2001, K. Ottewell, R. Leys (WAM T139470^DNA_Voucher_NCB_012^).

Paratypes. 1 ♀, 25 juveniles, Mummaloo, ca. 75 km NE. of Wubin (IBRA_AVW), Western Australia, Australia, 29°39'33"S, 117°13'52"E, hand collected from under *Eucalyptus* tree, 1 May 2012, M.K. Curran, G.B. Pearson (WAM T125751^DNA_Voucher_159^); 1 ♀, same data except 29°40'16"S, 117°13'41"E, 2 May 2012 (WAM T125754^DNA_Voucher_NCB_013^).

#### Other material examined.

**AUSTRALIA: *Western Australia***: 1 ♂, Dajoing Rock (IBRA_AVW), 30°26'S, 118°04'E, pitfall trap, 28 June–26 July 1985, B.Y. Main (WAM T139495); 1 juvenile, Mummaloo, ca. 75 km NE. of Wubin (IBRA_AVW), 29°39'33"S, 117°13'52"E, 1 May 2012, M.K. Curran, G.B. Pearson (WAM T125750); 1 juvenile, same data (WAM T125752); 1 juvenile, same data except 3 July 2012, M.K. Curran, S.R. Bennett (WAM T125755); 2 ♂, Mungarri Nature Reserve, north, site BE12 (IBRA_AVW), 30°19'51"S, 117°45'12"E, wet pitfall traps, 15 September 1998–25 October 1999, P. Van Heurck, CALM Survey (WAM T139516); 1 ♀, Mt Churchman (IBRA_COO), 29°55'S, 117°54'E, 27 July 1985, B.Y. Main (WAM T144852); 1 ♀, 16 km N. of Rothsay (IBRA_YAL), 29°09'12"S, 116°49'12"E, 6 July 2014, M. Bamford (WAM T136187).

#### Etymology.

The specific epithet is derived from the Latin *formosus* (adjective: ‘beautiful’; see Brown 1956), in reference to the ornate abdominal colouration of this species.

#### Diagnosis.

*Idiosoma formosum* is one of nine south-western Australian species in the *intermedium*- and *sigillatum*-clades which does not belong to the distinctive ‘sigillate complex’ (Fig. 25); these nine species can be distinguished from those ‘sigillate complex’ taxa (i.e., *I. arenaceum*, *I. clypeatum*, *I. dandaragan*, *I. kopejtkaorum*, *I. kwongan*, *I. nigrum* and *I. schoknechtorum*) by the absence of well-defined lateral sclerotic strips on the male abdomen (e.g., Figs 151, 212, 234), and by the significantly less sclerotised morphology of the female abdomen (which may be strongly corrugate but never leathery and ‘shield-like’) (e.g., Figs 4, 7, 8, 159, 220, 242). Males of *I. formosum* can be further distinguished from those of *I. gutharuka* and *I. incomptum* by the presence of enlarged (i.e., clearly visible) SP4 sclerites (Fig. 151; cf. Figs 186, 199); from *I. jarrah* and *I. mcclementsorum* by the colour of the legs, which do not have strongly contrasting bright yellow or orange-yellow femora (Fig. 152; cf. Figs 235, 292); from *I. gardneri* and *I. sigillatum* by the absence of well-defined dorso-lateral abdominal corrugations or striations (Figs 146, 151; cf. Figs 168, 173, 352, 357, Key pane 9.1); and from *I. intermedium* and *I. mcnamarai* by the shape of tibia I, which is short and stout (with the prolateral clasping spurs occupying most of the distal half of the segment) (Fig. 152; cf. Figs 174, 213, 314), and by the colour of the abdomen, which is ornately bi-coloured dorsally and postero-dorsally (Figs 146, 151; cf. Figs 168, 173, 207, 212, 308, 313).

Females can be distinguished from those of *I. mcclementsorum* and *I. sigillatum* by the absence of reinforced, sclerotised ridges on the abdomen (Figs 159, 162; cf. Figs 299, 302, 365, 368); from *I. intermedium* and *I. jarrah* by the size of the SP4 sclerites, which are significantly larger than the SP2 sclerites (Fig. 162; cf. Figs 223, 245); and from *I. mcnamarai* by the colour of the abdomen, which is ornately bi-coloured dorsally and postero-dorsally (Figs 159, 162; cf. Figs 321, 324) [NB. females of *I. gardneri*, *I. gutharuka* and *I. incomptum* are unknown].

This species can also be distinguished from *I. corrugatum* (from the Eyre Peninsula of South Australia) by the shape of the prolateral clasping spurs on the male tibia I, which are oriented longitudinally (Fig. 153; cf. Fig. 109), and by the shape of the female eye group, which is broadly trapezoidal (Fig. 161; cf. Fig. 117).

#### Description (male holotype).

**Figures 145–154.**
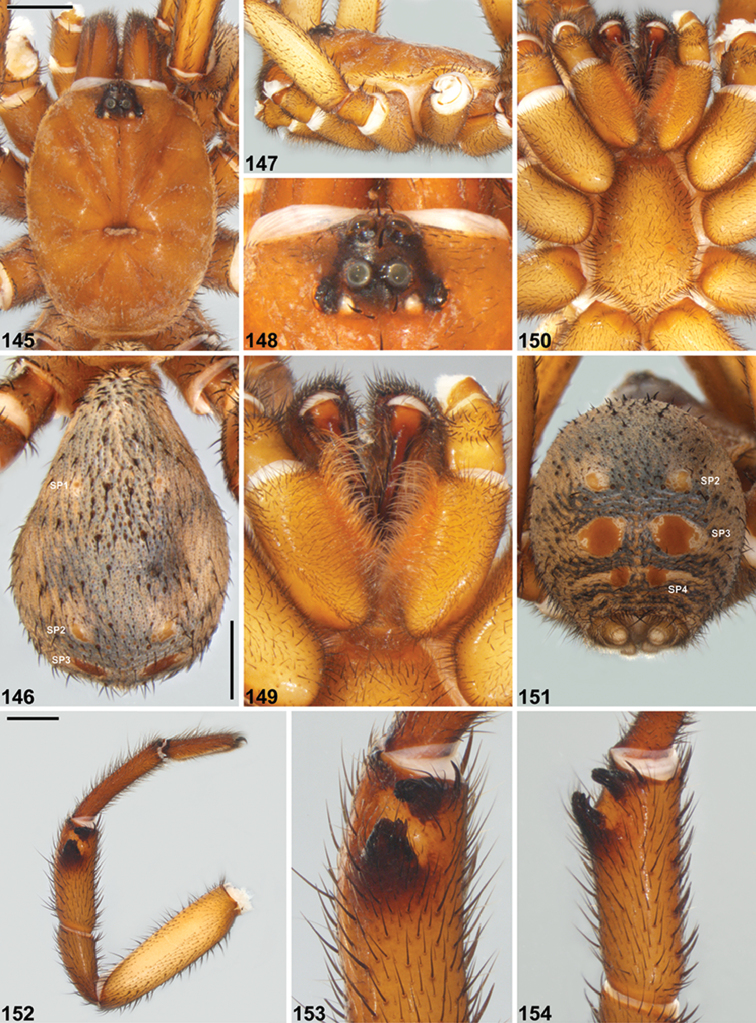
*Idiosoma formosum* sp. n., male holotype (WAM T139470) from Mount Gibson Station (Western Australia; AVW), somatic morphology. **145–146** Carapace and abdomen, dorsal view **147** Carapace, lateral view **148** Eyes, dorsal view **149** Mouthparts, ventral view **150** Sternum, ventral view **151** Abdomen, posterior view **152** Leg I, prolateral view **153** Leg I tibia, clasping spurs, prolateral view **154** Leg I tibia, proventral view. Scale bars 2.0. SP1–4 = sigilla pairs 1–4.

**Figures 155–157.**
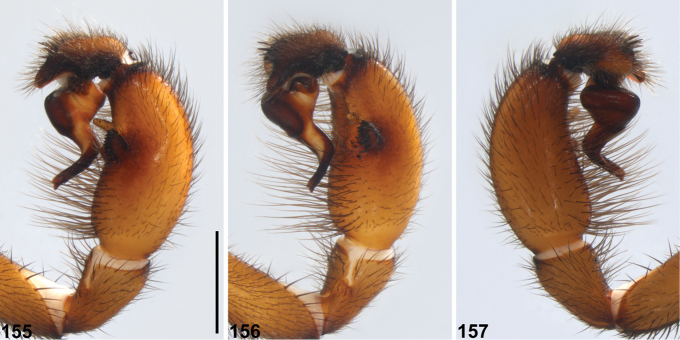
*Idiosoma formosum* sp. n., male holotype (WAM T139470) from Mount Gibson Station (Western Australia; AVW), pedipalp. **155** Retrolateral view **156** Retroventral view **157** Prolateral view. Note that the tip of the embolus has broken off this specimen. Scale bar 2.0.

Total length 18.1. Carapace 7.8 long, 5.9 wide. Abdomen 8.2 long, 5.6 wide. Carapace (Fig. 145) tan, with darker ocular region; lateral margins with uniformly-spaced fringe of porrect black setae; fovea slightly procurved. Eye group (Fig. 148) trapezoidal (anterior eye row strongly procurved), 0.7 × as long as wide, PLE–PLE/ALE–ALE ratio 2.0; ALE almost contiguous; AME separated by less than their own diameter; PME separated by 2.9 × their own diameter; PME and PLE separated by approximately diameter of PME, PME positioned in line with level of PLE. Maxillae with field of small cuspules confined to inner corner; labium without cuspules. Abdomen (Figs 146, 151) oval (slightly indented anteriorly), beige-brown in dorsal view with darker grey pattern medially and posteriorly, and assortment of stiff, porrect black setae, each with slightly raised, dark brown sclerotic base. Posterior abdomen moderately sigillate (Figs 146, 151); SP2 sclerites irregular, comma-shaped spots; SP3 sclerites subcircular with irregular margins, each with unsclerotised triangular ‘corner’ laterally; SP4 sclerites circular, each surrounded by chevron-like pad of unsclerotised cuticle laterally; SP5 obscured. Legs (Figs 152–154) variable shades of tan, with light scopulae on tarsi I–II; distal tibia I with pair of large prolateral clasping spurs oriented longitudinally. Leg I: femur 6.6; patella 3.5; tibia 4.2; metatarsus 4.7; tarsus 2.8; total 21.8. Leg I femur–tarsus/carapace length ratio 2.8. Pedipalpal tibia (Figs 155–157) 2.2 × longer than wide; RTA burr-like, with conical basal protuberance and field of retroventral spinules; digital process porrect, unmodified. Cymbium (Figs 155–157) setose, with field of spinules disto-dorsally. Embolus (Figs 155–157) broadly twisted and sharply tapering distally (broken at tip), with prominent longitudinal flange and triangular (sub-distal) embolic apophysis.

#### Description (female WAM T125751).

**Figures 158–166.**
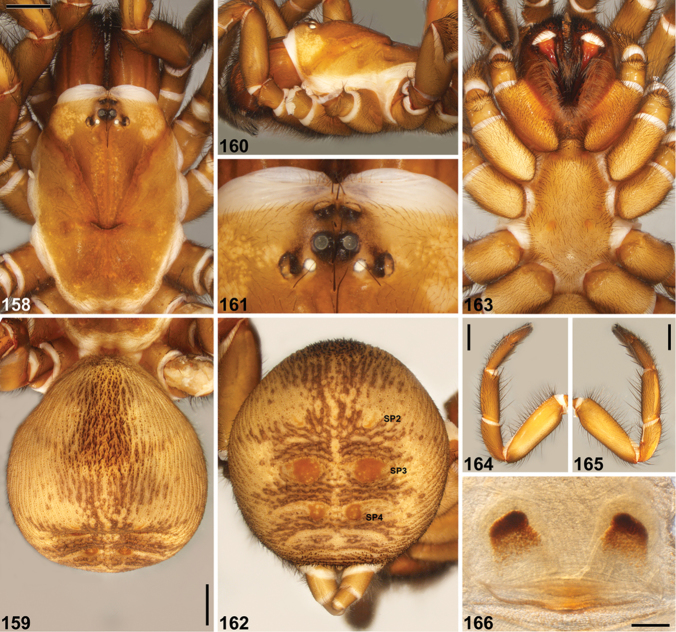
*Idiosoma formosum* sp. n., female paratype (WAM T125751) from Mummaloo (Western Australia; AVW). **158–159** Carapace and abdomen, dorsal view **160** Carapace, lateral view **161** Eyes, dorsal view **162** Abdomen, posterior view **163** Sternum, ventral view **164** Leg I, prolateral view **165** Leg I, retrolateral view **166** Spermathecae, dorsal view. Scale bars 2.0 (**158–159, 164–165**), 0.5 (**166**). SP2–4 = sigilla pairs 2–4.

Total length 24.3. Carapace 9.6 long, 9.8 wide. Abdomen 10.7 long, 9.8 wide. Carapace (Fig. 158) tan, with darker ocular region; fovea procurved. Eye group (Fig. 161) trapezoidal (anterior eye row strongly procurved), 0.7 × as long as wide, PLE–PLE/ALE–ALE ratio 2.4; ALE almost contiguous; AME separated by approximately their own diameter; PME separated by 3.5 × their own diameter; PME and PLE separated by more than diameter of PME, PME positioned in line with level of PLE. Maxillae with field of cuspules confined to inner corner (Fig. 163); labium without cuspules. Abdomen (Figs 159, 162) broadly oval and somewhat truncate posteriorly, beige-tan in dorsal view with darker brown pattern medially and posteriorly, with numerous stout setae on sclerotic bases and scattered sclerotic spots; longest stout setae clustered along median cardiac region. Posterior abdomen moderately sigillate (Figs 159, 162); SP2 sclerites irregular, comma-shaped spots; SP3 sclerites subcircular with irregular margins, each surrounded by pad of unsclerotised cuticle; SP4 sclerites subcircular with irregular margins, each surrounded by chevron-like pad of unsclerotised cuticle laterally; SP5 obscured; posterior margin of abdomen weakly corrugate, with rows of modified stout setae. Legs (Figs 164–165) variable shades of tan; scopulae present on tarsi and metatarsi I–II; tibia I with two stout pro-distal macrosetae and row of five longer retroventral macrosetae; metatarsus I with eight stout macrosetae; tarsus I with distal cluster of short macrosetae. Leg I: femur 5.9; patella 3.8; tibia 3.8; metatarsus 3.0; tarsus 1.9; total 18.6. Leg I femur–tarsus/carapace length ratio 1.9. Pedipalp tan, spinose on tibia and tarsus, with thick tarsal scopula. Genitalia (Fig. 166) with pair of obliquely angled spermathecae on broad ‘stems’, each bearing dense field of glandular vesicles distally, and more sparsely distributed glandular field sub-distally.

#### Distribution and remarks.

*Idiosoma formosum* (formerly known by WAM identification code ‘MYG262’), a member of the diverse *sigillatum*-clade (Fig. 25), is restricted to the Lake Moore catchment of south-western Australia, in a relatively small area near the junction of the Wheatbelt, Yalgoo, and Coolgardie bioregions (Fig. 375). It extends from near Rothsay in the north, south-east to Mungarri Nature Reserve and Dajoing Rock, both in the Cleary-Beacon-Wialki region. This distribution seems to be strongly correlated with annual rainfall of 250–300 mm, and red clay soils to the west and south-east of Lake Moore. North of Cleary and Beacon, the range of *I. formosum* overlaps the northern extent of the range of the closely related sister species *I. mcnamarai*.

Although first collected by Barbara Main at Dajoing Rock in the mid-1980s, and subsequently collected during the ‘Salinity Action Plan Survey’ of the late 1990s (Keighery 2004), this species came to prominence during environmental impact assessment surveys conducted in the resource-rich Mount Gibson and Mummaloo regions in the years 2001–2012. Morphological and molecular data were concordant in evidencing two *nigrum*-group species in this area, and *I. formosum* was designated the working codes ‘MYG262’ (WAM) and ‘sp. B01’ (Bennelongia Environmental Consultants) to distinguish it from what is now known to be *I. kopejtkaorum* (the latter formerly confused with *I. nigrum*). Under this ‘MYG262’ code name, *I. formosum* was also formally assessed in 2017 for threatened species listing under the *Western Australian Wildlife Conservation Act 1950* (see below). Burrows of this species are adorned with a ‘moustache-like’ arrangement of twig-lines, and males have been collected wandering in search of females in winter.

#### Conservation assessment.

In 2017, *Idiosoma formosum* was formally assessed and listed as Endangered (B1ab[iii] + B2ab[iii]) under the *Western Australian Wildlife Conservation Act 1950* (approved 16 January 2018; see W. A. Government Gazette 2018); this assessment incorporated the latest taxonomic, geographic and genetic data summarised in the current study (with a number of additional records also identified subsequently). In the heavily cleared north-eastern Wheatbelt, the threats to the species are manifold (as they are for *I. nigrum*; see above), and in the Mummaloo/Mount Gibson region to the west of Lake Moore, the species is (and will continue to be) at risk from mining and minerals resource development. It has a known extent of occurrence of nearly 4,000 km^2^ [3,780 km^2^], and an area of occupancy within that range of < 500 km^2^. Further close assessment under both Criteria A and B will be crucial to the continued survival of this species.

### Idiosoma gardneri

Taxon classificationAnimaliaAraneaeIdiopidae

Rix & Harvey sp. n.

http://zoobank.org/6C3BFD95-4502-4DDF-A235-EA9FA4DA31F7

Figs 167–176 , 177–179 , 375

#### Type material.

Holotype male. Mount Lesueur [Lesueur National Park] (IBRA_GES), Western Australia, Australia, 30°10'S, 115°12'E, pitfall trap, 1989, K. Gaull (WAM T139528).

#### Etymology.

The specific epithet is named in honour of the late Charles Gardner (1896–1970), former curator of the Western Australian Herbarium, whose conservation efforts were instrumental in protecting the Lesueur National Park for posterity.

#### Diagnosis.

*Idiosoma gardneri* is one of nine south-western Australian species in the *intermedium*- and *sigillatum*-clades which does not belong to the distinctive ‘sigillate complex’ (Fig. 25); these nine species can be distinguished from those ‘sigillate complex’ taxa (i.e., *I. arenaceum*, *I. clypeatum*, *I. dandaragan*, *I. kopejtkaorum*, *I. kwongan*, *I. nigrum* and *I. schoknechtorum*) by the absence of well-defined lateral sclerotic strips on the male abdomen (e.g., Figs 151, 212, 234), and by the significantly less sclerotised morphology of the female abdomen (which may be strongly corrugate but never leathery and ‘shield-like’) (e.g., Figs 4, 7, 8, 159, 220, 242). Males of *I. gardneri* can be further distinguished from those of *I. gutharuka* and *I. incomptum* by the presence of enlarged (i.e., clearly visible) SP4 sclerites (Fig. 173; cf. Figs 186, 199); from *I. jarrah* and *I. mcclementsorum* by the colour of the legs, which do not have strongly contrasting bright yellow or orange-yellow femora (Fig. 174; cf. Figs 235, 292); from *I. formosum*, *I. intermedium* and *I. mcnamarai* by the presence of well-defined dorso-lateral abdominal corrugations and striations (Figs 168, 173; cf. Figs 146, 151, 207, 212, 308, 313, Key pane 9.2); and from *I. sigillatum* by the larger, more closely spaced SP3 sclerites and the larger SP4 sclerites (Fig. 173, Key pane 10.2; cf. Fig. 357, Key pane 10.1). Males of this species can also be distinguished from those of *I. corrugatum* (from the Eyre Peninsula of South Australia) by the shape of the prolateral clasping spurs on tibia I, which are oriented longitudinally (Fig. 175; cf. Fig. 109). Females are unknown.

#### Description (male holotype).

**Figures 167–176.**
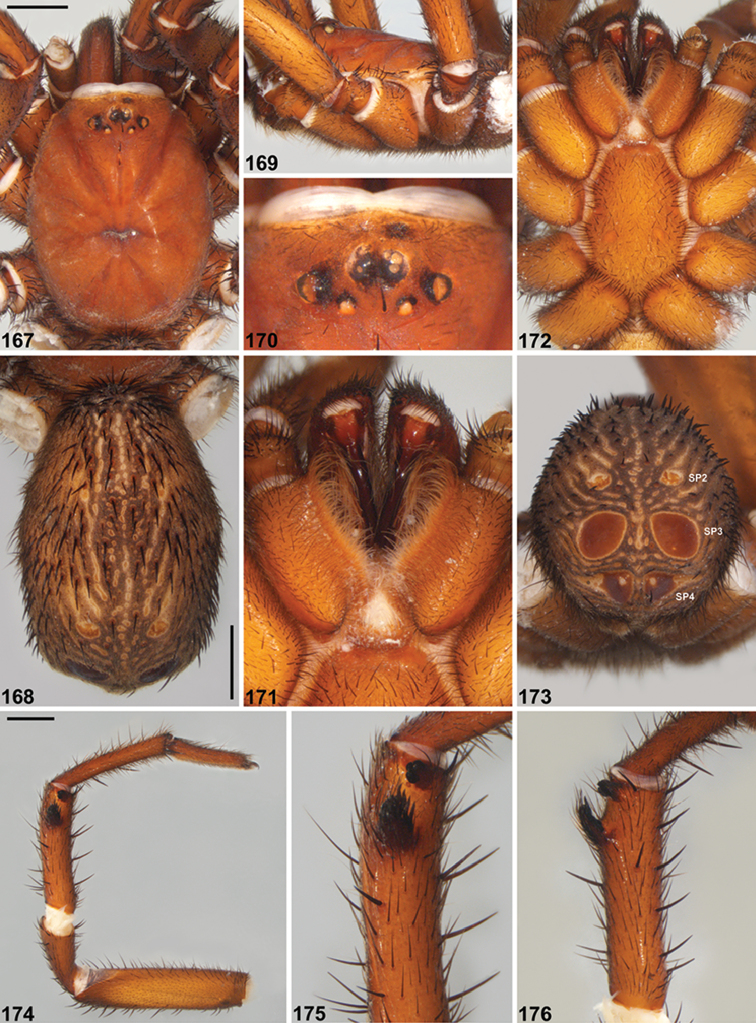
*Idiosoma gardneri* sp. n., male holotype (WAM T139528) from Lesueur National Park (Western Australia; GES), somatic morphology. **167–168** Carapace and abdomen, dorsal view **169** Carapace, lateral view **170** Eyes, dorsal view **171** Mouthparts, ventral view **172** Sternum, ventral view **173** Abdomen, posterior view **174** Leg I, prolateral view **175** Leg I tibia, clasping spurs, prolateral view **176** Leg I tibia, proventral view. Scale bars 2.0. SP2–4 = sigilla pairs 2–4.

**Figures 177–179.**
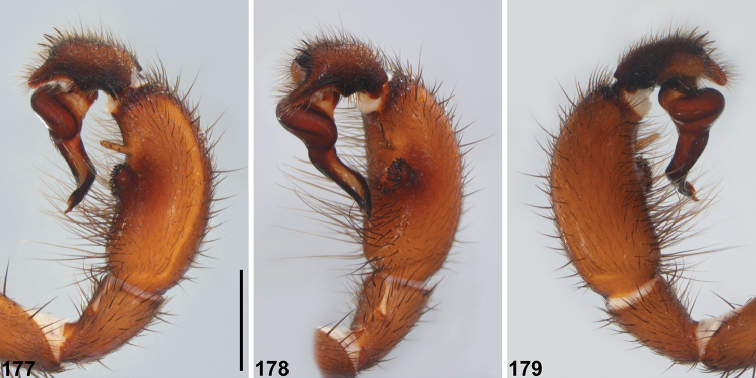
*Idiosoma gardneri* sp. n., male holotype (WAM T139528) from Lesueur National Park (Western Australia; GES), pedipalp. **177** Retrolateral view **178** Retroventral view **179** Prolateral view. Scale bar 2.0.

Total length 19.2. Carapace 8.1 long, 6.2 wide. Abdomen 8.4 long, 5.5 wide. Carapace (Fig. 167) dark tan, with darker ocular region; lateral margins with uniformly-spaced fringe of porrect black setae; fovea procurved. Eye group (Fig. 82) trapezoidal; anterior eye row developmentally disfigured, with ALE absent and AME each with multiple asymmetrical ocelli; PME separated by 3.6 × their own diameter; PME and PLE separated by slightly more than diameter of PME, PME positioned slightly posterior to level of PLE. Maxillae with field of small cuspules confined to inner corner; labium without cuspules. Abdomen (Figs 168, 173) oval, dark brown in dorsal view with paler tan striations, dorso-lateral corrugations and scattered dorsal sclerotic spots. Dorsal surface of abdomen (Fig. 168) more heavily setose anteriorly, with assortment of stiff, porrect black setae, each with slightly raised, dark brown sclerotic base. Posterior abdomen moderately sigillate (Figs 168, 173); SP2 sclerites irregular, comma-shaped spots; SP3 sclerites large and circular; SP4 sclerites subquadrate, each surrounded by chevron-like pad of unsclerotised cuticle laterally; SP5 obscured. Legs (Figs 174–176) variable shades of dark tan, with light scopulae on tarsi I–II; distal tibia I with pair of large prolateral clasping spurs oriented longitudinally. Leg I: femur 7.2; patella 3.7; tibia 5.2; metatarsus 5.6; tarsus 3.3; total 25.0. Leg I femur–tarsus/carapace length ratio 3.1. Pedipalpal tibia (Figs 177–179) 2.3 × longer than wide; RTA burr-like, with conical basal protuberance and field of retroventral spinules; digital process porrect, unmodified. Cymbium (Figs 177–179) setose, with field of spinules disto-dorsally. Embolus (Figs 177–179) broadly twisted and sharply tapering distally, with prominent longitudinal flange and triangular (sub-distal) embolic apophysis.

#### Distribution and remarks.

*Idiosoma gardneri* (formerly known by WAM identification code ‘MYG476’) is known only from Lesueur National Park, in the southern Geraldton Sandplains bioregion of south-western Western Australia (Fig. 375). Only a single specimen has ever been collected, and nothing is known of its biology. It is most similar to *I. sigillatum*, and the two are likely to be sister species.

#### Conservation assessment.

Due to the known occurrence of this species at only a single site, in an area with a relatively large amount of high quality and poorly surveyed heathland habitat, we consider it data deficient for the purposes of conservation assessment.

### Idiosoma gutharuka

Taxon classificationAnimaliaAraneaeIdiopidae

Rix & Harvey sp. n.

http://zoobank.org/E2EC1EB4-4422-494E-9A8E-58468891C86D

Figs 180–189 , 190–192 , 376

#### Type material.

Holotype male. Gutha (IBRA_AVW), Western Australia, Australia, 28°59'34"S, 115°56'23"E, wet pitfall trap, 23 May–17 September 1996, M.S. Harvey, J.M. Waldock (WAM T38517).

#### Etymology.

The specific epithet is a noun in apposition derived from a contraction of ‘Gutha’ and ‘Pintharuka’, in reference to the region where this species has been found.

#### Diagnosis.

*Idiosoma gutharuka* is one of nine south-western Australian species in the *intermedium*- and *sigillatum*-clades which does not belong to the distinctive ‘sigillate complex’ (Fig. 25); these nine species can be distinguished from those ‘sigillate complex’ taxa (i.e., *I. arenaceum*, *I. clypeatum*, *I. dandaragan*, *I. kopejtkaorum*, *I. kwongan*, *I. nigrum* and *I. schoknechtorum*) by the absence of well-defined lateral sclerotic strips on the male abdomen (e.g., Figs 151, 212, 234), and by the significantly less sclerotised morphology of the female abdomen (which may be strongly corrugate but never leathery and ‘shield-like’) (e.g., Figs 4, 7, 8, 159, 220, 242). Males of *I. gutharuka* can be further distinguished from those of *I. formosum*, *I. gardneri*, *I. intermedium*, *I. jarrah*, *I. mcclementsorum*, *I. mcnamarai* and *I. sigillatum* by the small size of the SP3 sclerites, which are only marginally larger than the SP2 sclerites (Fig. 186; cf. Figs 151, 173, 212, 234, 291, 313, 357), and by the absence of clearly visible SP4 sclerites (Fig. 186; cf. Figs 151, 173, 212, 234, 291, 313, 357); and from *I. incomptum* by the shape of the embolus, which is sharply tapering distally with a prominent longitudinal flange (Figs 190–192; cf. Figs 203–205). Males of this species can also be distinguished from those of *I. corrugatum* (from the Eyre Peninsula of South Australia) by the shape of the prolateral clasping spurs on tibia I, which are oriented longitudinally (Fig. 188; cf. Fig. 109). Females are unknown.

#### Description (male holotype).

**Figures 180–189.**
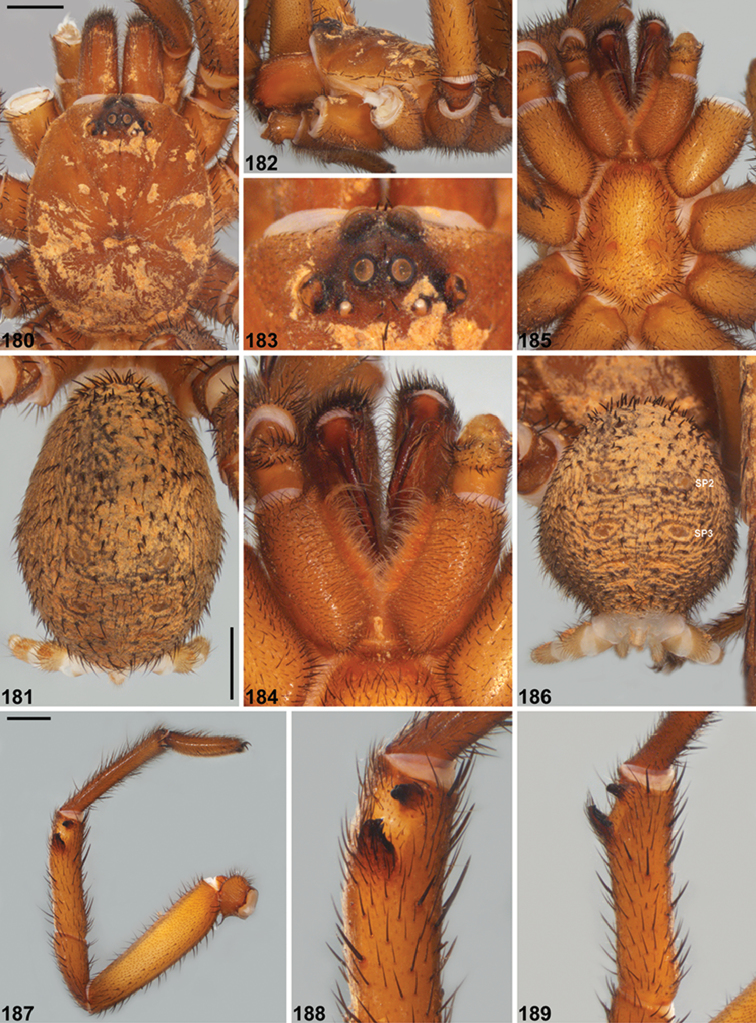
*Idiosoma gutharuka* sp. n., male holotype (WAM T38517) from Gutha (Western Australia; AVW), somatic morphology. **180–181** Carapace and abdomen, dorsal view **182** Carapace, lateral view **183** Eyes, dorsal view **184** Mouthparts, ventral view **185** Sternum, ventral view **186** Abdomen, posterior view **187** Leg I, prolateral view **188** Leg I tibia, clasping spurs, prolateral view **189** Leg I tibia, proventral view. Scale bars 2.0. SP2–3 = sigilla pairs 2–3.

**Figures 190–192.**
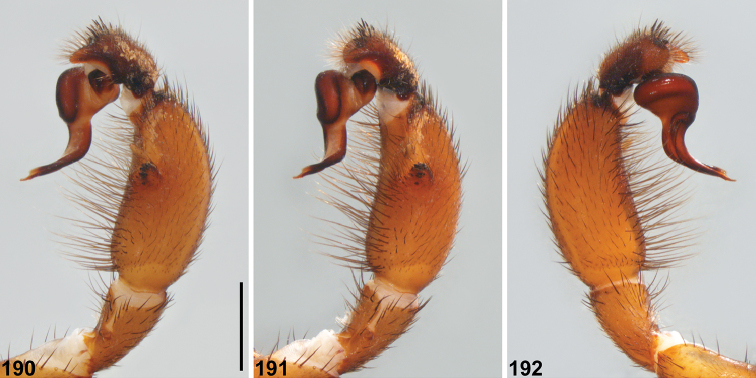
*Idiosoma gutharuka* sp. n., male holotype (WAM T38517) from Gutha (Western Australia; AVW), pedipalp. **190** Retrolateral view **191** Retroventral view **192** Prolateral view. Scale bar 2.0.

Total length 19.4. Carapace 8.6 long, 6.6 wide. Abdomen 8.1 long, 5.6 wide. Carapace (Fig. 180) tan, with darker ocular region and partial coating of dirt on dorsal surface; lateral margins with uniformly-spaced fringe of porrect black setae; fovea procurved. Eye group (Fig. 183) trapezoidal (anterior eye row strongly procurved), 0.6 × as long as wide, PLE–PLE/ALE–ALE ratio 2.1; ALE almost contiguous; AME separated by less than their own diameter; PME separated by 4.9 × their own diameter; PME and PLE separated by slightly more than diameter of PME, PME positioned in line with level of PLE. Maxillae and labium without cuspules. Abdomen (Figs 181, 186) oval, with thin coating of dirt on dorsal surface (obscuring most of underlying cuticle) and assortment of stiff, porrect black setae, each with slightly raised, dark brown sclerotic base. Posterior abdomen largely asigillate (Figs 181, 186); SP2 sclerites irregular, comma-shaped spots; SP3 sclerites only marginally larger than SP2 sclerites, each surrounded by pad of unsclerotised cuticle; SP4 and SP5 obscured. Legs (Figs 187–189) variable shades of tan, with light scopulae on tarsi I–II; distal tibia I with pair of large prolateral clasping spurs oriented longitudinally. Leg I: femur 7.8; patella 4.0; tibia 5.6; metatarsus 6.2; tarsus 3.4; total 27.0. Leg I femur–tarsus/carapace length ratio 3.1. Pedipalpal tibia (Figs 190–192) 2.4 × longer than wide; RTA burr-like, with conical basal protuberance and field of retroventral spinules; digital process porrect, unmodified. Cymbium (Figs 190–192) setose, with field of spinules disto-dorsally. Embolus (Figs 190–192) broadly twisted and sharply tapering distally, with prominent longitudinal flange and small triangular (sub-distal) embolic apophysis.

#### Distribution and remarks.

*Idiosoma gutharuka* (formerly known by WAM identification code ‘MYG157’) is known only from Gutha, near Pintharuka in the northern Wheatbelt bioregion of south-western Western Australia (Fig. 376). Only a single specimen has ever been collected, and nothing is known of its biology. Like *I. incomptum*, *I. gutharuka* exhibits a rudimentary, largely symplesiomorphic morphology between unmodified congeners and the more obviously phragmotic taxa in the *clypeatum*- and *sigillatum*-clades. It is most similar to *I. incomptum*, and the two are likely to be sister species.

#### Conservation assessment.

*Idiosoma gutharuka* is known from only a single site in the northern Wheatbelt. Given: (i) this highly restricted geographic range; (ii) the sampling effort that has occurred in surrounding areas as a result of a major biotic survey (see Keighery 2004) and a long history of incidental collecting; and (iii) the continuing decline in the area, extent and/or quality of habitat in the northern Wheatbelt agricultural zone (Laurance et al. 2011), this species is considered Critically Endangered (B1ab[iii] + B2ab[iii]). Urgent close assessment under both Criteria A and B will be crucial to the continued survival of this species.

### Idiosoma incomptum

Taxon classificationAnimaliaAraneaeIdiopidae

Rix & Harvey sp. n.

http://zoobank.org/2CFAAFD1-3C3A-4730-9487-64B17D485B95

Figs 25 , 193–202 , 203–205 , 376

#### Type material.

Holotype male. Carnarvon, Police and Citizens Youth Centre (IBRA_CAR), Western Australia, Australia, 24°52'08"S, 113°40'59"E, outside building, morning, after rain, 10 July 2009, J.M. Waldock (WAM T99997^DNA_Voucher_58^).

#### Other material examined.

**AUSTRALIA: *Western Australia***: 1 ♂, Boolathana Station, site BO1 (IBRA_CAR), 24°24'48"S, 113°39'47"E, wet pitfall trap, 29 May–25 August 1995, N. Hall, WAM/CALM Carnarvon Survey (WAM T98474); 1 ♂, same data except site BO2, 24°24'47"S, 113°40'30"E, 30 May–25 August 1995 (WAM T98469); 1 ♂, Bush Bay, site BB3 (IBRA_CAR), 25°04'40"S, 113°42'37"E, wet pitfall trap, July 1994, N. Hall, WAM/CALM Carnarvon Survey (WAM T98472); 2 ♂, Francois Peron National Park, site PE2 (IBRA_CAR), 25°52'31"S, 113°32'59"E, wet pitfall trap, 25 May–30 August 1995, N. Hall, WAM/CALM Carnarvon Survey (WAM T98473); 1 ♂, Nanga Station, site NA2 (IBRA_CAR), 26°29'23"S, 114°03'24"E, wet pitfall trap, 11 May–30 August 1995, N. Hall, WAM/CALM Carnarvon Survey (WAM T98470); 2 ♂, Zuytdorp, site ZU4 (IBRA_GES), 27°15'45"S, 114°09'13"E, wet pitfall trap, 18 May–16 August 1995, N. Hall, WAM/CALM Carnarvon Survey (WAM T98475); 1 ♂, same data except site ZU5, 27°14'43"S, 114°11'36"E, 17 May–16 August 1995 (WAM T98471).

#### Etymology.

The specific epithet is derived from the Latin *incomptus* (adjective: ‘unadorned’; see Brown 1956), in reference to the small sigilla and largely unsclerotised abdominal morphology of this species.

#### Diagnosis.

*Idiosoma incomptum* is one of nine south-western Australian species in the *intermedium*- and *sigillatum*-clades which does not belong to the distinctive ‘sigillate complex’ (Fig. 25); these nine species can be distinguished from those ‘sigillate complex’ taxa (i.e., *I. arenaceum*, *I. clypeatum*, *I. dandaragan*, *I. kopejtkaorum*, *I. kwongan*, *I. nigrum* and *I. schoknechtorum*) by the absence of well-defined lateral sclerotic strips on the male abdomen (e.g., Figs 151, 212, 234), and by the significantly less sclerotised morphology of the female abdomen (which may be strongly corrugate but never leathery and ‘shield-like’) (e.g., Figs 4, 7, 8, 159, 220, 242). Males of *I. incomptum* can be further distinguished from those of *I. formosum*, *I. gardneri*, *I. intermedium*, *I. jarrah*, *I. mcclementsorum*, *I. mcnamarai* and *I. sigillatum* by the small size of the SP3 sclerites, which are only marginally larger than the SP2 sclerites (Fig. 199; cf. Figs 151, 173, 212, 234, 291, 313, 357), and by the absence of clearly visible SP4 sclerites (Fig. 199; cf. Figs 151, 173, 212, 234, 291, 313, 357); and from *I. gutharuka* by the shape of the embolus, which gradually tapers distally and is without a prominent flange (Figs 203–205; cf. Figs 190–192). Males of this species can also be distinguished from those of *I. corrugatum* (from the Eyre Peninsula of South Australia) by the shape of the prolateral clasping spurs on tibia I, which are oriented longitudinally (Fig. 201; cf. Fig. 109). Females are unknown.

#### Description (male holotype).

**Figures 193–202.**
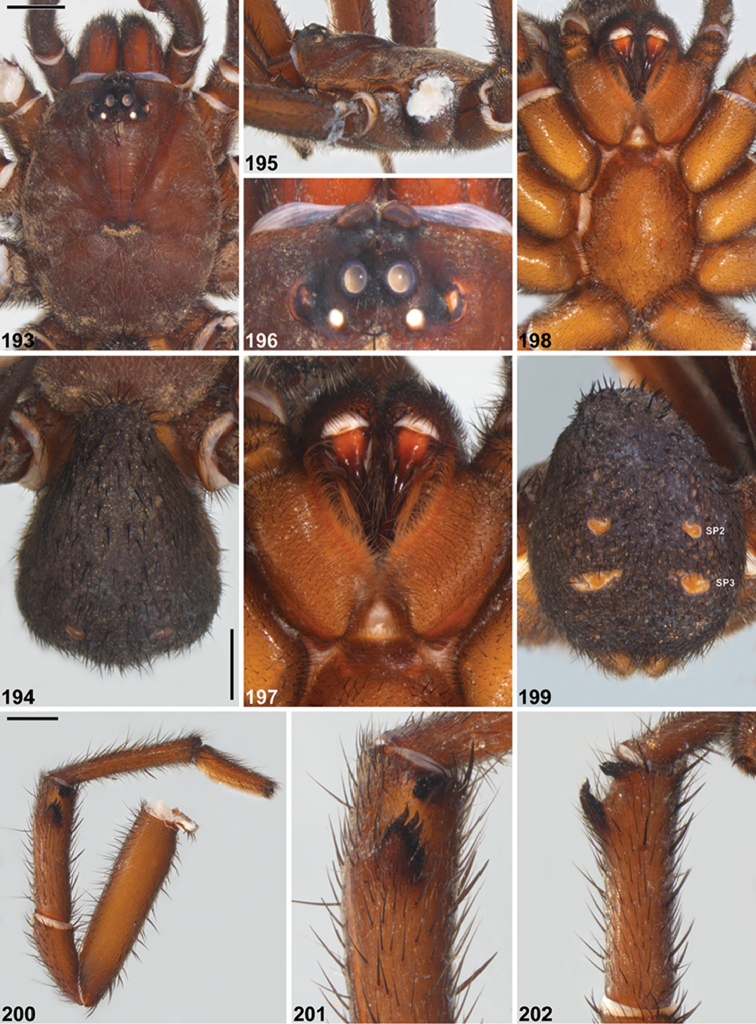
*Idiosoma incomptum* sp. n., male holotype (WAM T99997) from Carnarvon (Western Australia; CAR), somatic morphology. **193–194** Carapace and abdomen, dorsal view **195** Carapace, lateral view **196** Eyes, dorsal view **197** Mouthparts, ventral view **198** Sternum, ventral view **199** Abdomen, posterior view **200** Leg I, prolateral view **201** Leg I tibia, clasping spurs, prolateral view **202** Leg I tibia, proventral view. Scale bars 2.0. SP2–3 = sigilla pairs 2–3.

**Figures 203–205.**
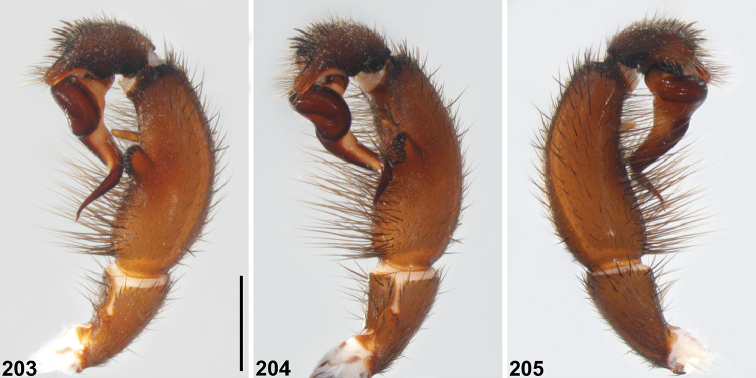
*Idiosoma incomptum* sp. n., male holotype (WAM T99997) from Carnarvon (Western Australia; CAR), pedipalp. **203** Retrolateral view **204** Retroventral view **205** Prolateral view. Scale bar 2.0.

Total length 18.7. Carapace 9.3 long, 7.0 wide. Abdomen 7.6 long, 5.8 wide. Carapace (Fig. 193) dark chocolate-brown, with darker ocular region; lateral margins with uniformly-spaced fringe of porrect black setae; fovea procurved. Eye group (Fig. 196) trapezoidal (anterior eye row strongly procurved), 0.7 × as long as wide, PLE–PLE/ALE–ALE ratio 2.2; ALE almost contiguous; AME separated by less than their own diameter; PME separated by 3.3 × their own diameter; PME and PLE separated by slightly more than diameter of PME, PME positioned slightly posterior to level of PLE. Maxillae with field of small cuspules confined to inner corner; labium without cuspules. Abdomen (Figs 194, 199) broadly oval, charcoal-coloured in dorsal view with faint tan mottling and assortment of stiff, porrect black setae, each with slightly raised, dark brown sclerotic base. Posterior abdomen largely asigillate (Figs 194, 199); SP2 sclerites comma-shaped spots; SP3 sclerites only marginally larger than SP2 sclerites, each surrounded by pad of unsclerotised cuticle; SP4 and SP5 obscured. Legs (Figs 200–202) variable shades of dark tan, with light scopulae on tarsi I–II; distal tibia I with pair of large prolateral clasping spurs oriented longitudinally. Leg I: femur 7.9; patella 4.1; tibia 5.5; metatarsus 6.3; tarsus 3.2; total 27.0. Leg I femur–tarsus/carapace length ratio 2.9. Pedipalpal tibia (Figs 203–205) 2.5 × longer than wide; RTA burr-like, with rounded basal protuberance and field of retroventral spinules; digital process porrect, unmodified. Cymbium (Figs 203–205) setose, with field of spinules disto-dorsally. Embolus (Figs 203–205) twisted and gradually tapering distally; embolic apophysis absent.

#### Distribution and remarks.

*Idiosoma incomptum* (formerly known by WAM identification code ‘MYG130’), a member of the *intermedium*-clade (Fig. 25), has a relatively widespread, near-coastal distribution in Western Australia’s southern Carnarvon Basin, from Zuytdorp north to at least Boolathana Station (Fig. 376). Like *I. gutharuka*, *I. incomptum* exhibits a rudimentary, largely symplesiomorphic morphology between unmodified congeners and the more obviously phragmotic taxa in the *clypeatum*- and *sigillatum*-clades. It is most similar to *I. gutharuka*, and the two are likely to be sister species. Little is known of the biology of *I. incomptum*, other than that males have been collected wandering in search of females in winter and possibly late autumn.

#### Conservation assessment.

*Idiosoma incomptum* has a known extent of occurrence of nearly 6,500 km^2^ [6,270 km^2^; with coastline as western margin excluding the Carrarang Peninsula], although this value is likely to be an underestimate (possibly severely so), due to a paucity of inland records. The area of occupancy within that range is similarly difficult to estimate based on past survey effort. As such, we consider this species data deficient for the purposes of conservation assessment.

### Idiosoma intermedium

Taxon classificationAnimaliaAraneaeIdiopidae

Rix & Harvey sp. n.

http://zoobank.org/6EB66C6F-97ED-45E9-9821-C533120D26A7

Figs 25 , 206–215 , 216–218 , 219–227 , 376

#### Type material.

Holotype male. Bodallin (IBRA_AVW), Western Australia, Australia, 31°22'S, 118°51'E, 26 June 1970, L.C. Birlles (WAM T139520).

#### Other material examined.

**AUSTRALIA: *Western Australia***: 1 ♂, Billiburning Rock (IBRA_COO), 30°10'S, 117°55'E, 19 May 1985, B.Y. Main (WAM T139494); 1 ♀, same data (WAM T144851); ♀, same data (WAM T144854); 1 ♀, ca. 80 km NE. of Bullfinch, north of J5 (IBRA_COO), 30°18'20"S, 119°36'06"E, hand collected, 20 November 2015, M.K. Curran, D. Harms (WAM T140940^DNA_Voucher_NCB_005^); 1 juvenile, same data except NE. of J5, 30°15'23"S, 119°24'03"E (WAM T140939); 1 juvenile, same data except J5 deposit, 30°21'55"S, 119°36'45"E (WAM T140938); 1 ♀, Bungalbin Hill, ca. 48.2 km NNE. of Koolyanobbing (IBRA_COO), 30°29'51"S, 119°35'59"E, 9–17 October 2012, N. Dight (WAM T127934); 1 ♀, 5.5 km SE. of Koolyanobbing (IBRA_COO), 30°51'06"S, 119°33'43"E, dug from burrow, 23 August 2009, R. Teale (WAM T99749^DNA_Voucher_133^); 1 juvenile, Mt Manning area, site CR4 (IBRA_COO), 30°28'53"S, 119°59'43"E, open tall eucalypt woodland with mixed shrubs, 20 June 2008, J, Francesconi et al. (WAM T92079^DNA_Voucher_310^); 1 ♂, Mungarri Nature Reserve, south, site BE13 (IBRA_AVW), 30°20'55"S, 117°45'29"E, wet pitfall traps, 15 September 1998–25 October 1999, L. King, CALM Survey (WAM T139517^DNA_Voucher_NCB_011^); 1 ♂, Warrachuppin North Road, site MN2 (IBRA_AVW), 31°00'06"S, 118°41'31"E, wet pitfall traps, 21 May–22 September 1998, N. Guthrie, CALM Survey (WAM T139519^DNA_Voucher_NCB_010^).

#### Etymology.

The specific epithet is derived from the Latin *intermedius* (adjective: ‘in between’, ‘intermediate’; see Brown 1956), in reference to the intermediate size of the sigilla and relatively unsclerotised abdominal morphology of this species.

#### Diagnosis.

*Idiosoma intermedium* is one of nine south-western Australian species in the *intermedium*- and *sigillatum*-clades which does not belong to the distinctive ‘sigillate complex’ (Fig. 25); these nine species can be distinguished from those ‘sigillate complex’ taxa (i.e., *I. arenaceum*, *I. clypeatum*, *I. dandaragan*, *I. kopejtkaorum*, *I. kwongan*, *I. nigrum* and *I. schoknechtorum*) by the absence of well-defined lateral sclerotic strips on the male abdomen (e.g., Figs 151, 212, 234), and by the significantly less sclerotised morphology of the female abdomen (which may be strongly corrugate but never leathery and ‘shield-like’) (e.g., Figs 4, 7, 8, 159, 220, 242). Males of *I. intermedium* can be further distinguished from those of *I. gutharuka* and *I. incomptum* by the presence of enlarged (i.e., clearly visible) SP4 sclerites (Fig. 212; cf. Figs 186, 199); from *I. jarrah* and *I. mcclementsorum* by the colour of the legs, which do not have strongly contrasting bright yellow or orange-yellow femora (Fig. 213; cf. Figs 235, 292); from *I. gardneri* and *I. sigillatum* by the absence of well-defined dorso-lateral abdominal corrugations or striations (Figs 207, 212, Key pane 9.2; cf. Figs 168, 173, 352, 357, Key pane 9.1); from *I. formosum* by the shape of tibia I, which is longer (with the prolateral clasping spurs occupying the distal third of the segment) (Fig. 213; cf. Fig. 152), and by the colour of the abdomen, which is more uniformly coloured dorsally (Figs 207, 212; cf. Figs 146, 151); and from *I. mcnamarai* by the smaller SP4 sclerites (Fig. 212; cf. Fig. 313), and by the morphology of the SP3 sclerites, each of which may have a laterally or postero-laterally directed triangular ‘corner’ laterally (as opposed to an antero-laterally directed triangular ‘corner’) (Fig. 212, Key pane 12.3; cf. Fig. 313, Key panes 12.1, 12.2).

Females can be distinguished from those of *I. mcclementsorum* and *I. sigillatum* by the absence of reinforced, sclerotised ridges on the abdomen (Figs 220, 223; cf. Figs 299, 302, 365, 368); from *I. formosum* and *I. mcnamarai* by the size of the SP4 sclerites, which are not significantly larger than the SP2 sclerites (Fig. 223; cf. Figs 162, 324); and from *I. jarrah* by the slightly larger size of the SP3 and SP4 sclerites (Fig. 223; cf. Fig. 245) [NB. females of *I. gardneri*, *I. gutharuka* and *I. incomptum* are unknown].

This species can also be distinguished from *I. corrugatum* (from the Eyre Peninsula of South Australia) by the shape of the prolateral clasping spurs on the male tibia I, which are oriented longitudinally (Fig. 214; cf. Fig. 109), and by the shape of the female eye group, which is broadly trapezoidal (Fig. 222; cf. Fig. 117).

#### Description (male holotype).

**Figures 206–215.**
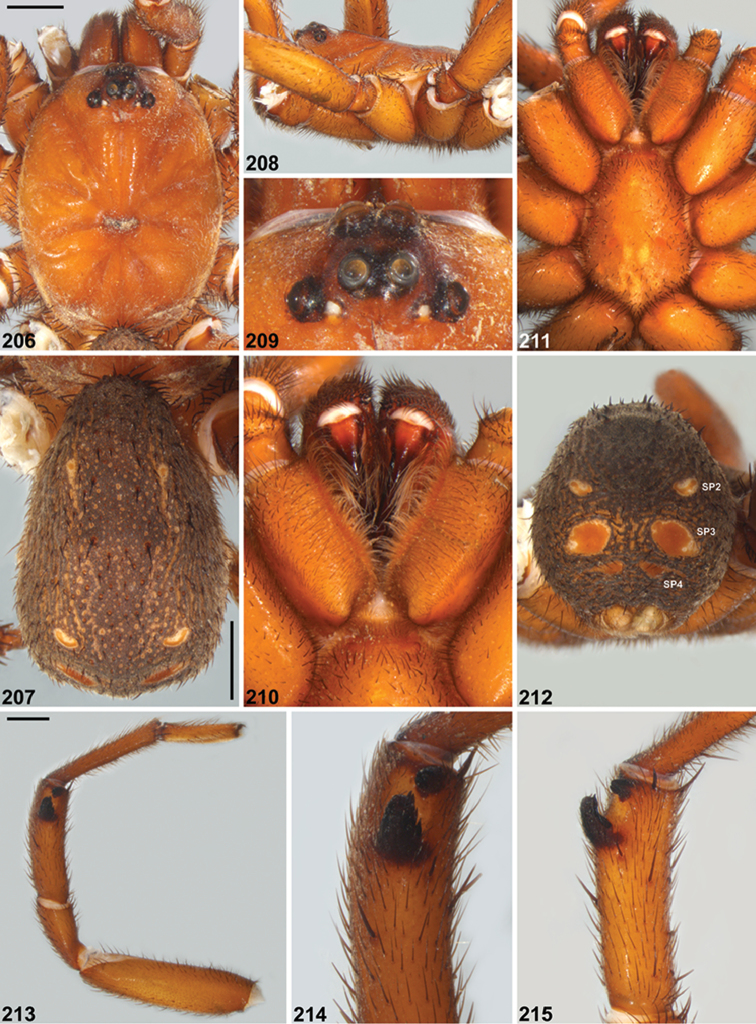
*Idiosoma intermedium* sp. n., male holotype (WAM T139520) from Bodallin (Western Australia; AVW), somatic morphology. **206–207** Carapace and abdomen, dorsal view **208** Carapace, lateral view **209** Eyes, dorsal view **210** Mouthparts, ventral view **211** Sternum, ventral view **212** Abdomen, posterior view **213** Leg I, prolateral view **214** Leg I tibia, clasping spurs, prolateral view **215** Leg I tibia, proventral view. Scale bars 2.0. SP2–4 = sigilla pairs 2–4.

**Figures 216–218.**
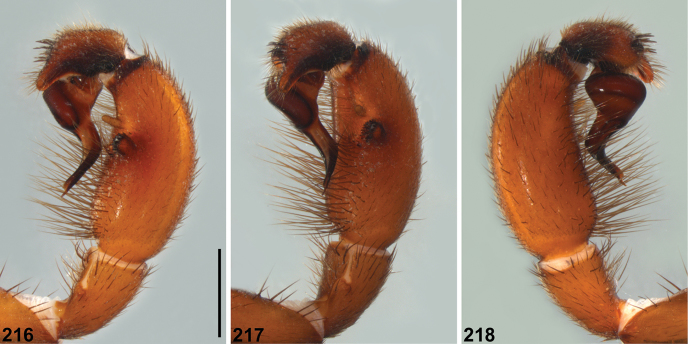
*Idiosoma intermedium* sp. n., male holotype (WAM T139520) from Bodallin (Western Australia’ AVW), pedipalp. **216** Retrolateral view **217** Retroventral view **218** Prolateral view. Scale bar 2.0.

Total length 19.8. Carapace 9.7 long, 7.3 wide. Abdomen 8.2 long, 5.4 wide. Carapace (Fig. 206) dark tan, with darker ocular region; lateral margins with uniformly-spaced fringe of porrect black setae; fovea procurved. Eye group (Fig. 209) trapezoidal (anterior eye row strongly procurved), 0.6 × as long as wide, PLE–PLE/ALE–ALE ratio 2.3; ALE almost contiguous; AME separated by less than their own diameter; PME separated by 5.3 × their own diameter; PME and PLE separated by slightly more than diameter of PME, PME positioned in line with level of PLE. Maxillae with field of small cuspules confined to inner corner; labium without cuspules. Abdomen (Figs 207, 212) oval, charcoal-brown in dorsal view with tan mottling and assortment of stiff, porrect black setae, each with slightly raised, dark brown sclerotic base. Posterior abdomen moderately sigillate (Figs 207, 212); SP2 sclerites comma-shaped spots; SP3 sclerites circular with irregular margins, each with unsclerotised triangular ‘corner’ laterally; SP4 sclerites oval, each surrounded by chevron-like pad of unsclerotised cuticle laterally; SP5 obscured. Legs (Figs 213–215) variable shades of dark tan, with light scopulae on tarsi I–II; distal tibia I with pair of large prolateral clasping spurs oriented longitudinally. Leg I: femur 8.0; patella 4.1; tibia 5.7; metatarsus 6.1; tarsus 3.7; total 27.5. Leg I femur–tarsus/carapace length ratio 2.8. Pedipalpal tibia (Figs 216–218) 2.4 × longer than wide; RTA burr-like, with conical basal protuberance and field of retroventral spinules; digital process porrect, unmodified. Cymbium (Figs 216–218) setose, with field of spinules disto-dorsally. Embolus (Figs 216–218) broadly twisted and sharply tapering distally, with prominent longitudinal flange and triangular (sub-distal) embolic apophysis.

#### Description (female WAM T99749).

**Figures 219–227.**
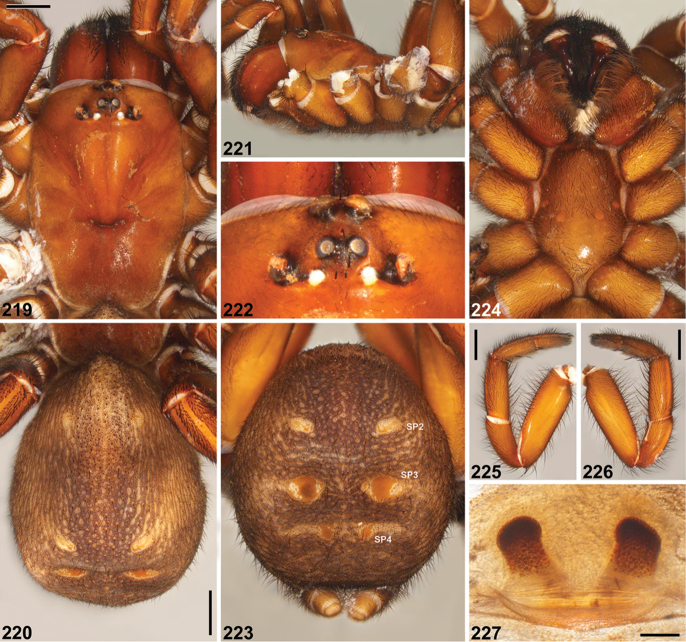
*Idiosoma intermedium* sp. n., female (WAM T99749) from SE. of Koolyanobbing (Western Australia; COO). **219–220** Carapace and abdomen, dorsal view **221** Carapace, lateral view **222** Eyes, dorsal view **223** Abdomen, posterior view **224** Sternum, ventral view **225** Leg I, prolateral view **226** Leg I, retrolateral view **227** Spermathecae, dorsal view. Scale bars 2.0 (**219–220, 225–226**), 0.5 (**227**). SP2–4 = sigilla pairs 2–4.

Total length 25.0. Carapace 10.5 long, 7.3 wide. Abdomen 11.6 long, 8.9 wide. Carapace (Fig. 219) dark tan, with darker ocular region; fovea procurved. Eye group (Fig. 222) trapezoidal (anterior eye row strongly procurved), 0.6 × as long as wide, PLE–PLE/ALE–ALE ratio 2.4; ALE almost contiguous; AME separated by approximately their own diameter; PME separated by 1.9 × their own diameter; PME and PLE separated by more than diameter of PME, PME positioned in line with level of PLE. Maxillae with field of cuspules confined to inner corner (Fig. 224); labium without cuspules. Abdomen (Figs 220, 223) broadly oval, dark grey-brown in dorsal view with tan mottling. Posterior abdomen moderately sigillate (Figs 220, 223); SP2 sclerites irregular, comma-shaped spots; SP3 sclerites subcircular with irregular margins, each surrounded by pad of unsclerotised cuticle; SP4 sclerites subcircular with irregular margins, each surrounded by chevron-like pad of unsclerotised cuticle laterally; SP5 obscured. Legs (Figs 225–226) variable shades of dark tan; scopulae present on tarsi and metatarsi I–II; tibia I with one stout pro-distal macroseta (broken at base) and row of seven longer retroventral macrosetae; metatarsus I with nine stout macrosetae; tarsus I with distal cluster of short macrosetae. Leg I: femur 6.4; patella 4.2; tibia 4.1; metatarsus 3.0; tarsus 2.4; total 20.1. Leg I femur–tarsus/carapace length ratio 1.9. Pedipalp dark tan, spinose on tibia and tarsus, with thick tarsal scopula. Genitalia (Fig. 227) with pair of short spermathecae on broad ‘stems’, each bearing dense field of glandular vesicles distally, and more sparsely distributed glandular field sub-distally.

#### Distribution and remarks.

*Idiosoma intermedium* (formerly known by WAM identification code ‘MYG475’), the nominate member of the *intermedium*-clade (Fig. 25), has a relatively widespread albeit poorly defined distribution in the eastern Wheatbelt and north-western Coolgardie bioregions of south-western Western Australia (Fig. 376). Its known range extends from Bodallin north to Billiburning Rock in the eastern Wheatbelt, and east to near the Helena-Aurora Range, Mount Manning, and Koolyanobbing in the Coolgardie bioregion. Like *I. jarrah*, *I. formosum* and *I. mcnamarai*, *I. intermedium* exhibits a transitional morphology between other species in the *intermedium*-clade (i.e. *I. incomptum* and *I. gutharuka*), and the more obviously phragmotic taxa in the *clypeatum*- and *sigillatum*-clades. Little is known of the biology of this species, other than that males have been collected wandering in search of females in late autumn and winter.

#### Conservation assessment.

In 2017, *Idiosoma intermedium* was formally assessed as ‘priority 3’ fauna; this assessment incorporated the latest taxonomic, geographic and genetic data summarised in the current study (with a number of additional records also identified subsequently). It has a known extent of occurrence of nearly 14,500 km^2^ [14,102 km^2^] (a likely underestimate due to limited survey effort), and while it therefore cannot be considered threatened under Criterion B, a ‘priority 3’ recommendation was made due to the occurrence of this species in areas prospective for mining and mineral resources. Further close assessment under both Criteria A and B is warranted in the future.

### Idiosoma jarrah

Taxon classificationAnimaliaAraneaeIdiopidae

Rix & Harvey sp. n.

http://zoobank.org/F0637000-C6D0-4AF3-85B2-BF8EEB76EC58

Figs 7 , 22 , 23 , 25 , 228–237 , 238–240 , 241–249 , 375

#### Type material.

Holotype male. Lesmurdie, 42 Wheelwright Road (IBRA_JAF), Western Australia, Australia, 32°00'20"S, 116°02'59"E, walking in garden on overcast day, 13 June 2012, D. & R. Roberts (WAM T124143^DNA_Voucher_124^).

Paratype. 1 ♀, Lesmurdie, Armour Way (IBRA_JAF), Western Australia, Australia, 32°01'S, 116°03'E, inside house, 28 July 1996, J. Barkla (WAM T44390).

#### Other material examined.

**AUSTRALIA: *Western Australia***: 1 ♀, Lesmurdie (IBRA_JAF), 32°01'S, 116°03'E, 1 November 1975, C. O’Neill (WAM T26108); 1 juvenile, off Albany Highway, just N. of Arthur River bridge (IBRA_JAF), 33°16'22"S, 117°00'54"E, hand collected, *Allocasuarina* forest, 4 October 2013, M.G. Rix, M.S. Harvey (WAM T131632^DNA_Voucher_90^); 1 ♂, SW. of Boddington, Worsley Alumina Overland Conveyor Belt, conveyor #1, line stand 5527 (IBRA_JAF), 33°02'S, 116°16'E, 29 June 2006, J. Hynes (WAM T99952^DNA_Voucher_131^); 1 ♂, SW. of Boddington, Worsley Alumina, near Lower Hotham Road, Overland Conveyor Belt #1, line stand 900 (IBRA_JAF), 32°57'S, 116°25'E, hand collected, Jan–November 2005, J. Hynes (WAM T74623); 1 ♀, Boya (IBRA_JAF), 31°54'56"S, 116°03'23"E, 24 November 1979, L. Webb (WAM T26821); 1 ♂, Bullsbrook (IBRA_SWA), 31°40'S, 116°02'E, 1 June 2001, B. Leslie (WAM T44228); 1 ♂, Bullsbrook, Smith Road (IBRA_JAF), 31°39'S, 116°06'E, 31 July 2015, T. Solig (WAM T136943^DNA_Voucher_NCB_002^); 1 ♂, Darlington (IBRA_JAF), 31°55'S, 116°04'E, 15 June 1986, M. McNab (WAM T26822); 1 ♀, same locality data except 260 Ryecroft Road, 31°55'18"S, 116°05'05"E, 31 July 2015, T. Rudas (WAM T136937); 1 ♂, Glen Forrest (IBRA_JAF), 31°54'50"S, 116°06'05"E, 4 July 1976, S.M. Postmus (WAM T26125); 1 ♂, Gooseberry Hill (IBRA_JAF), 31°58'S, 116°03'E, 14 July 1980, P. Kleins (WAM T139475); 1 ♀, same locality data except 31°57'20"S, 116°02'52"E, 15 January 1974, V.R. McDonald (WAM T18585); 1 ♀, same locality data except 3 Rich Road, 31°57'S, 116°03'E, 28 May 1998, C. Loos (WAM T44389); 1 ♀, Kalamunda (IBRA_JAF), 31°58'30"S, 116°03'25"E, 28 September 1959, M. Morton (WAM T26824); 1 ♀, same locality data, 12 August 1974, G.H. Lucas (WAM T26825); 1 ♀, Kelmscott (IBRA_SWA), 32°07'S, 116°01'E, 1 February 1977, J. Evans (WAM T26827); 1 ♀, Midland Junction (IBRA_SWA), 31°53'S, 115°59'E, 25 July 1928, Mr Marshall (WAM T1982); 1 ♂, Mount Cooke (IBRA_JAF), 32°25'S, 116°18'E, pitfall trap, 15 May–16 June 1991, M.S. Harvey, J.M. Waldock (WAM T26111); 1 ♂, Mount Helena (IBRA_JAF), 31°53'S, 116°12'E, 1 June 1999, K. Simmons (WAM T40601^DNA_Voucher_129^); 1 ♂, same locality data except 5 Treetop Way, 31°52'S, 116°13'E, 23 May 2005, T. Warda (WAM T63354); 1 ♂, Mount Nasura, 12 Westview Close (IBRA_JAF), 32°08'18"S, 116°01'50"E, 3 August 2006, C. & F. Motas (WAM T77021); 1 ♂, Mundaring (IBRA_JAF), 31°53'44"S, 116°10'11"E, 4 July 1972, N. Giles (WAM T26830); 1 ♀, same locality data except Primary School, 31°54'S, 116°10'E, 12 September 2000, C. Sander (WAM T42205); 1 ♂, Mundaring Weir Road, Kalamunda (IBRA_JAF), 31°58'30"S, 116°03'25"E, hand collected, 27 June 1976, K.L. Morrison (WAM T18582); 1 juvenile, Red Hill Valley, site 6 (IBRA_JAF), 31°51'S, 116°06'E, 22 June 1967, L.E. Koch, L.N. McKenna (WAM T28385); 1 ♂, Roleystone (IBRA_JAF), 32°06'51"S, 116°04'21"E, hand collected, 5 June 1984, A. Wright (WAM T18583); 1 ♀, same data except 6 August 1983, R. Herdsman (WAM T18586); 1 ♀, same data except 6 June 1987, R.E. Alexander (WAM T18587); 1 ♂, same locality data except 32°06'S, 116°04'E, 7 May 1977, D. Edward (WAM T139474); 1 ♂, Sawyers Valley (IBRA_JAF), 31°53'55"S, 116°12'05"E, hand collected, 8 September 1975, D. Parkinson (WAM T18584); 1 ♂, Stoneville (IBRA_JAF), 31°53'S, 116°10'E, 27 June 1999, K.W. Thomas (WAM T40632^DNA_Voucher_130^); 1 ♀, same locality data except 31°52'43"S, 116°10'03"E, 3 June 1982, L. Bosworth (WAM T26829); 1 ♂, Talbot Road Reserve, site TR3 (IBRA_SWA), 31°52'25"S, 116°03'03"E, wet pitfall trap, 24 June–28 July 1993, M.S. Harvey, J.M. Waldock (WAM T30019); 1 ♂, same data except site TR4, 31°52'23"S, 116°02'46"E (WAM T30020); 1 ♀, Walyunga National Park (IBRA_JAF), 31°44'S, 116°05'E, 17 April 1986, J. Wheeler (WAM T21181); 1 ♀, same data except 22 April 1986, K. Huskin (WAM T26831); 1 ♀, same data (WAM T26832); 1 juvenile, same data (WAM T26833); 1 juvenile, same data (WAM T26834); 1 juvenile, same data (WAM T26835); 1 juvenile, same data (WAM T26836); 1 juvenile, same data (WAM T26837); 1 juvenile, same data (WAM T26838); 1 juvenile, same data (WAM T26839); 1 juvenile, same data (WAM T26840); 1 juvenile, same data (WAM T26841); 1 juvenile, same data (WAM T26842); 1 juvenile, same data (WAM T26843); 1 juvenile, same data (WAM T26844); 1 juvenile, same data (WAM T26845); 1 juvenile, same data (WAM T26846); 1 juvenile, same data (WAM T26847); 1 juvenile, same data (WAM T26848); 1 juvenile, same data (WAM T26849); 1 juvenile, same data (WAM T26850); 1 juvenile, same data (WAM T26851); 1 juvenile, same data (WAM T26852); 1 juvenile, same data (WAM T26853); 1 juvenile, same data (WAM T26854); 1 ♂, West Midland (IBRA_SWA), 31°53'S, 116°00'E, August 1949, E. Clough (WAM T139473); 1 ♀, Whistlepipe Gully, Forrestfield (IBRA_SWA), 31°59'S, 116°01'E, 1 July 1965, W. Greenham (WAM T26823).

#### Etymology.

The specific epithet is a noun in apposition, in reference to the Jarrah Forest bioregion in which this species occurs.

#### Diagnosis.

*Idiosoma jarrah* is one of nine south-western Australian species in the *intermedium*- and *sigillatum*-clades which does not belong to the distinctive ‘sigillate complex’ (Fig. 25); these nine species can be distinguished from those ‘sigillate complex’ taxa (i.e., *I. arenaceum*, *I. clypeatum*, *I. dandaragan*, *I. kopejtkaorum*, *I. kwongan*, *I. nigrum* and *I. schoknechtorum*) by the absence of well-defined lateral sclerotic strips on the male abdomen (e.g., Figs 151, 212, 234), and by the significantly less sclerotised morphology of the female abdomen (which may be strongly corrugate but never leathery and ‘shield-like’) (e.g., Figs 4, 7, 8, 159, 220, 242). Males of *I. jarrah* can be further distinguished from those of *I. gutharuka* and *I. incomptum* by the presence of enlarged (i.e., clearly visible) SP4 sclerites (Fig. 234; cf. Figs 186, 199); from *I. formosum*, *I. gardneri*, *I. intermedium*, *I. mcnamarai* and *I. sigillatum* by the colour of the legs, which are bi-coloured with strongly contrasting bright yellow or orange-yellow femora (Fig. 235; cf. Figs 152, 174, 213, 314, 358); and from *I. mcclementsorum* by the size of the SP3 sclerites, which are relatively small (Fig. 234; cf. Fig. 291), and by the size of the SP4 sclerites, which are weakly sclerotised spots (Fig. 234; cf. Fig. 291).

Females can be distinguished from those of *I. mcclementsorum* and *I. sigillatum* by the absence of reinforced, sclerotised ridges on the abdomen (Figs 220, 223; cf. Figs 299, 302, 365, 368); from *I. formosum* and *I. mcnamarai* by the size of the SP4 sclerites, which are not significantly larger than the SP2 sclerites (Fig. 245; cf. Figs 162, 324); and from *I. intermedium* by the slightly smaller size of the SP3 and SP4 sclerites (Fig. 245; cf. Fig. 223) [NB. females of *I. gardneri*, *I. gutharuka* and *I. incomptum* are unknown].

This species can also be distinguished from *I. corrugatum* (from the Eyre Peninsula of South Australia) by the shape of the prolateral clasping spurs on the male tibia I, which are oriented longitudinally (Fig. 236; cf. Fig. 109), and by the shape of the female eye group, which is broadly trapezoidal (Fig. 244; cf. Fig. 117).

#### Description (male holotype).

**Figures 228–237.**
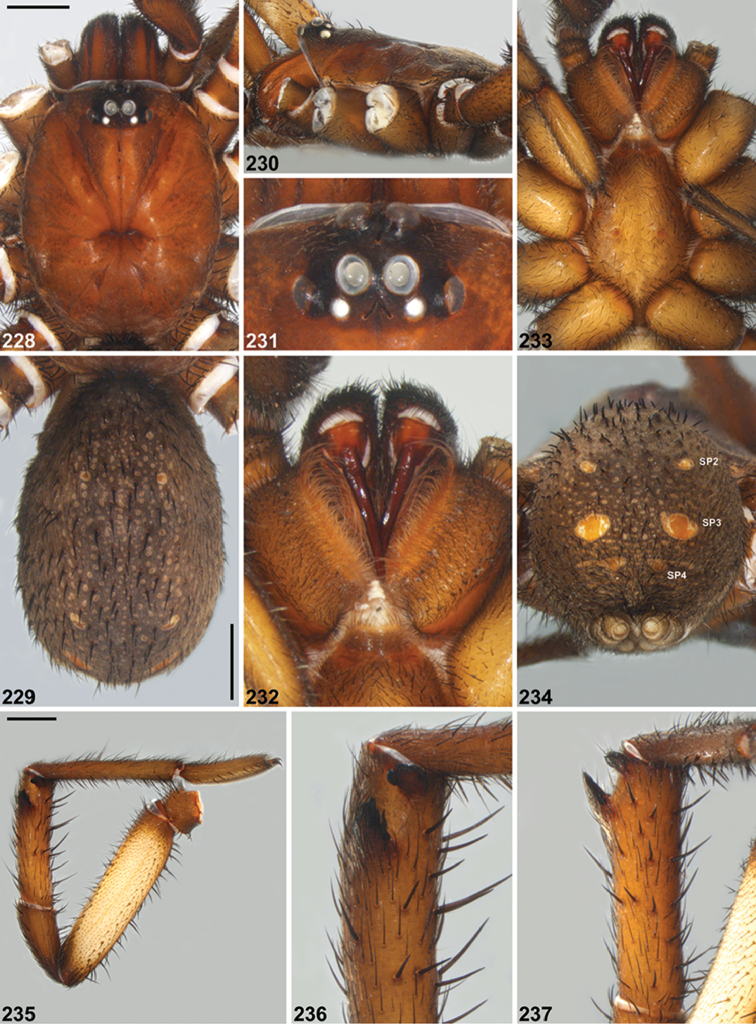
*Idiosoma jarrah* sp. n., male holotype (WAM T124143) from Lesmurdie (Western Australia; JAF), somatic morphology. **228–229** Carapace and abdomen, dorsal view **230** Carapace, lateral view **231** Eyes, dorsal view **232** Mouthparts, ventral view **233** Sternum, ventral view **234** Abdomen, posterior view **235** Leg I, prolateral view **236** Leg I tibia, clasping spurs, prolateral view **237** Leg I tibia, proventral view. Scale bars 2.0. SP2–4 = sigilla pairs 2–4.

**Figures 238–240.**
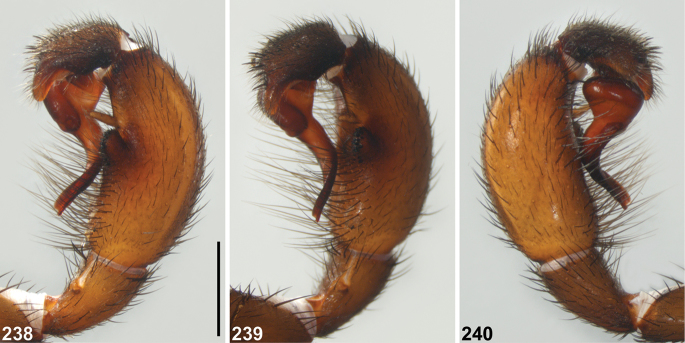
*Idiosoma jarrah* sp. n., male holotype (WAM T124143) from Lesmurdie (Western Australia; JAF), pedipalp. **238** Retrolateral view **239** Retroventral view **240** Prolateral view. Note that the tip of the embolus has broken off this specimen. Scale bar 2.0.

Total length 18.7. Carapace 8.3 long, 6.5 wide. Abdomen 8.3 long, 5.4 wide. Carapace (Fig. 228) dark tan and chocolate-brown, with darker ocular region; lateral margins with uniformly-spaced fringe of porrect black setae; fovea procurved. Eye group (Fig. 231) trapezoidal (anterior eye row strongly procurved), 0.7 × as long as wide, PLE–PLE/ALE–ALE ratio 2.1; ALE almost contiguous; AME separated by less than their own diameter; PME separated by 3.3 × their own diameter; PME and PLE separated by slightly more than diameter of PME, PME positioned in line with level of PLE. Maxillae with field of small cuspules confined to inner corner; labium without cuspules. Abdomen (Figs 229, 234) oval, charcoal-brown in dorsal view with tan mottling and assortment of stiff, porrect black setae, each with slightly raised, dark brown sclerotic base. Posterior abdomen weakly sigillate (Figs 229, 234); SP2 sclerites comma-shaped spots; SP3 sclerites subcircular with irregular margins, each surrounded by pad of unsclerotised cuticle; SP4 sclerites oval, each surrounded by chevron-like pad of unsclerotised cuticle laterally; SP5 obscured. Legs (Figs 235–237) bicoloured, variable shades of dark brown on patellae, tibiae, metatarsi and tarsi, and bright beige-tan on femora, with light scopulae on tarsi I–II; distal tibia I with pair of large prolateral clasping spurs oriented longitudinally. Leg I: femur 7.7; patella 3.8; tibia 5.4; metatarsus 6.4; tarsus 3.5; total 26.8. Leg I femur–tarsus/carapace length ratio 3.2. Pedipalpal tibia (Figs 238–240) 2.3 × longer than wide; RTA burr-like, with conical basal protuberance and field of retroventral spinules; digital process porrect, unmodified. Cymbium (Figs 238–240) setose, with field of spinules disto-dorsally. Embolus (Figs 238–240) broadly twisted and sharply tapering distally (broken at tip), with prominent longitudinal flange and very small triangular (sub-distal) embolic apophysis.

#### Description (female WAM T44390).

**Figures 241–249.**
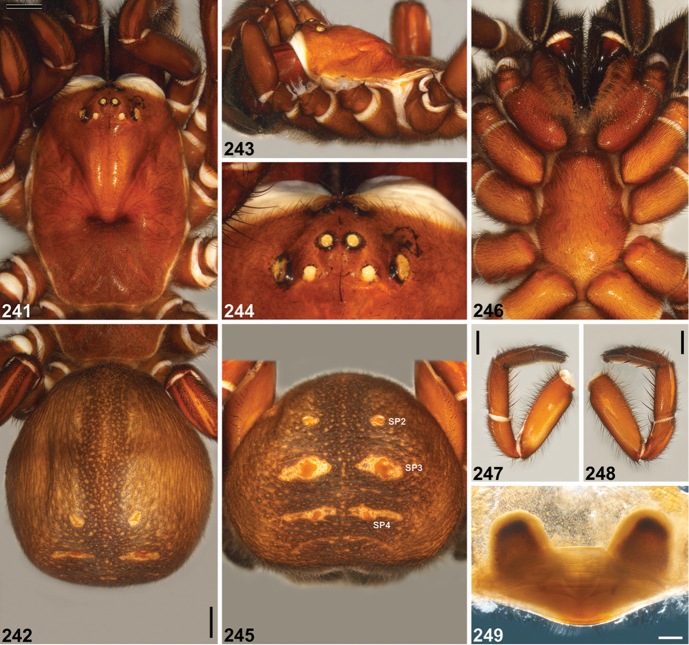
*Idiosoma jarrah* sp. n., female paratype (WAM T44390) from Lesmurdie (Western Australia; JAF). **241–242** Carapace and abdomen, dorsal view **243** Carapace, lateral view **244** Eyes, dorsal view **245** Abdomen, posterior view **246** Sternum, ventral view **247** Leg I, prolateral view **248** Leg I, retrolateral view **249** Spermathecae, dorsal view. Scale bars 2.0 (**241–242, 247–248**), 0.5 (**249**). SP2–4 = sigilla pairs 2–4.

Total length 31.2. Carapace 12.7 long, 8.9 wide. Abdomen 14.6 long, 13.5 wide. Carapace (Fig. 241) dark tan and chocolate-brown, with darker ocular region; fovea procurved. Eye group (Fig. 244) trapezoidal (anterior eye row strongly procurved), 0.6 × as long as wide, PLE–PLE/ALE–ALE ratio 2.3; ALE almost contiguous; AME separated by approximately their own diameter; PME separated by 3.3 × their own diameter; PME and PLE separated by more than diameter of PME, PME positioned in line with level of PLE. Maxillae with field of cuspules confined to inner corner (Fig. 246); labium without cuspules. Abdomen (Figs 242, 245) broadly oval, dark brown in dorsal view with tan mottling. Posterior abdomen weakly sigillate (Figs 242, 245); SP2 sclerites irregular, comma-shaped spots; SP3 sclerites subcircular with irregular margins, each surrounded by broad pad of unsclerotised cuticle; SP4 sclerites oval, each surrounded by chevron-like pad of unsclerotised cuticle laterally; SP5 obscured. Legs (Figs 247–248) variable shades of dark tan; scopulae present on tarsi and metatarsi I–II; tibia I with one stout pro-distal macroseta and row of five longer retroventral macrosetae; metatarsus I with six stout macrosetae; tarsus I with distal cluster of short macrosetae. Leg I: femur 7.3; patella 4.6; tibia 4.6; metatarsus 3.7; tarsus 2.7; total 22.9. Leg I femur–tarsus/carapace length ratio 1.8. Pedipalp dark tan, spinose on tibia and tarsus, with thick tarsal scopula. Genitalia (Fig. 249) with pair of short, obliquely angled spermathecae, each bearing dense field of glandular vesicles distally, and more sparsely distributed glandular field sub-distally.

#### Distribution and remarks.

*Idiosoma jarrah* (formerly known by WAM identification code ‘MYG156’) (Fig. 7), a member of the yellow legs-clade within the diverse *sigillatum*-clade (Fig. 25), is endemic to south-western Australia’s Jarrah Forest bioregion, where it occurs widely in mixed jarrah (*Eucalyptus marginata*) and marri (*Corymbia calophylla*) forest on and east of the Darling Escarpment, from Bullsbrook south to at least Boddington and Arthur River (Fig. 375). North of the Avon Valley it is replaced by its closely related sister species *I. mcclementsorum*, both of which are characterised by yellow leg femora in males (Figs 235, 292). Like *I. formosum*, *I. intermedium*, and *I. mcnamarai*, *I. jarrah* exhibits a transitional morphology between largely unmodified species in the *intermedium*-clade (i.e. *I. incomptum* and *I. gutharuka*), and the more obviously phragmotic taxa in the *clypeatum*- and *sigillatum*-clades. Burrows of this species are adorned with a ‘moustache-like’ arrangement of twig-lines (Figs 22, 23), and often occur under *Casuarina* or *Allocasuarina*, the leaves of which are used as twig-lines. Like *I. sigillatum* (see below), most males have been collected wandering in search of females during May–July (82% of *n* = 17), with a sudden onset of activity in May (with the first winter rains) and a peak of activity in June.

#### Conservation assessment.

*Idiosoma jarrah* has a known extent of occurrence (EOO) of nearly 4,000 km^2^ [3,907 km^2^], although this value is likely to be an underestimate due to the paucity of records throughout the south of its range. The area of occupancy within that range is similarly difficult to estimate, although is likely to be quite high as a proportion of EOO due to the amount of forest still present throughout most of its range. As such, we do not currently consider this species to be of conservation concern.

### Idiosoma kopejtkaorum

Taxon classificationAnimaliaAraneaeIdiopidae

Rix & Harvey sp. n.

http://zoobank.org/323EA3E4-D841-46AF-B8E2-BCF4B104174F

Figs 9 , 21 , 250–259 , 260–262 , 263–271 , 374

Idiosoma ‘*nigrum*’ Main, 1957b: 440 (in part; cited specimens from NE. of Rabbit Proof Fence and N. of Mt Gibson turnoff).

#### Type material.

Holotype male. Snake Gully Nature Reserve, site WU11 (IBRA_AVW), Western Australia, Australia, 30°13'05"S, 116°56'36"E, wet pitfall traps, 15 September 1997–7 April 1998, N. Guthrie, CALM Survey (WAM T144621).

Paratypes. 1 ♂, same data as holotype (WAM T139498); 1 ♂, same data (WAM T139499).

#### Other material examined.

**AUSTRALIA: *Western Australia***: 1 juvenile, Buntine Nature Reserve (IBRA_AVW), 29°59'58"S, 116°38'06"E, 6 January 2010, B. Durrant (WAM T108518^DNA_Voucher_147^); 1 ♀, Charles Darwin Nature Reserve (IBRA_AVW), 29°39'44"S, 117°07'15"E, 14 January 2010, B. Durrant (WAM T108519^DNA_Voucher_144^); 1 ♀, same locality data except 29°33'28"S, 117°01'28"E, 4 May 2016, K. Gruber, D. Schoknecht, J.A. Huey, M.S. Harvey (WAM T140751^DNA_Voucher_NCB_003^); 1 ♀, E. of Coorow (IBRA_AVW), 29°52'05"S, 116°09'07"E, 29 May 2008, F. Falconer (WAM T56344); 1 ♀, NE. of Goodlands (IBRA_AVW), 30°07'S, 117°10'E, 4 June 1984, B.Y. Main (WAM T144625); 1 ♂, Lake Goorly, north-west, site WU9 (IBRA_AVW), 29°49'50"S, 116°57'19"E, wet pitfall traps, 15 September 1998–25 October 1999, L. King, CALM Survey (WAM T139497); 1 ♀, Maya Nature Reserve (IBRA_AVW), 29°49'24"S, 116°31'48"E, 7 January 2010, B. Durrant (WAM T108523^DNA_Voucher_145^); 1 ♀, Mummaloo, ca. 72 km NE. of Wubin (IBRA_AVW), 29°40'18"S, 117°10'59"E, 13 August 2012, M.K. Curran, S.R. Bennett (WAM T126455^DNA_Voucher_NCB_019^); 1 juvenile, same data except 29°38'17"S, 117°09'05"E, 7 August 2012 (WAM T126451); 1 juvenile, same data except 29°38'46"S, 117°10'17"E, 8 August 2012 (WAM T126452); 1 ♀, same data except ca. 75 km NE. of Wubin, 29°38'17"S, 117°13'52"E, 4 July 2012 (WAM T125765^DNA_Voucher_143^); 1 ♀, same data except ca. 76 km NE. of Wubin, 29°40'20"S, 117°14'27"E, 10 August 2012 (WAM T126453); 1 ♀, same data except 29°41'07"S, 117°16'04"E, 9 August 2012 (WAM T126454); 1 juvenile, same data except ca. 58 km NE. of Wubin, 29°46'04"S, 117°04'39"E, 16 August 2012 (WAM T126457); 1 juvenile, same data except 29°48'20"S, 117°07'24"E (WAM T126456); 1 juvenile, same data except ca. 65 km NE. of Wubin, 29°41'03"S, 117°06'12"E (WAM T126458); 1 ♀, 17 miles NE. of Rabbit Proof Fence no. 2, 32 miles NE. of Wubin (IBRA_AVW), 29°47'S, 117°01'E, 4 September 1955, B.Y. Main (WAM T144795); 1 juvenile, same data (WAM T144796); 1 ♀, 39 miles NE. of Rabbit Proof Fence, 5 miles N. of Mt Gibson turnoff (IBRA_AVW), 29°35'S, 117°07'E, 4 September 1955, B.Y. Main (WAM T144797).

#### Etymology.

The specific epithet is named in honour of Paul and Karen Kopejtka, in recognition of their generous support for the Western Australian Museum Foundation.

#### Diagnosis.

*Idiosoma kopejtkaorum* is one of seven highly autapomorphic species in the polyphyletic ‘sigillate complex’ (Fig. 25); members of this complex can be distinguished from all other species in the *nigrum*-group from south-western Australia (i.e., *I. formosum*, *I. gardneri*, *I. gutharuka*, *I. incomptum*, *I. intermedium*, *I. jarrah*, *I. mcclementsorum*, *I. mcnamarai* and *I. sigillatum*) by the presence of well-defined lateral sclerotic strips on the male abdomen (e.g., Figs 32, 63, 256), and by the very heavily sclerotised, leathery, ‘shield-like’ morphology of the female abdomen (e.g., Figs 1–3, 9–12, 52, 74, 96). Males of *I. kopejtkaorum* can be further distinguished from those of *I. arenaceum* by the shape of the SP4 sclerites, which are not elongate-oval (Fig. 256; cf. Fig. 63); from *I. kwongan* by the absence of semi-circular lateral indentations adjacent to the SP4 sclerites (Fig. 256; cf. Fig. 278, Key pane 13.1); from *I. dandaragan*, *I. nigrum* and *I. schoknechtorum* by the absence of a prominent sub-distal embolic apophysis (Key pane 14.2; cf. Key pane 14.1); and from *I. clypeatum* by the less heavily setose morphology of metatarsus I (Fig. 257, Key pane 16.2; cf. Fig. 86, Key pane 16.1).

Females can be distinguished from those of *I. arenaceum* by the shape of the SP4 sclerites, which are not elongate-oval (Fig. 267, Key pane 22.1; cf. Fig. 74, Key pane 21.1); and from *I. dandaragan*, *I. nigrum* and *I. schoknechtorum* by the size of the SP4 sclerites, which are approximately half the size of the SP3 sclerites (Fig. 267, Key pane 22.1; cf. Figs 43, 52, 140, 346, Key panes 23.1–23.3) [NB. females of *I. kwongan* are unknown]. By our assessment, females of *I. kopejtkaorum* are morphologically indistinguishable from those of *I. clypeatum*; males, molecular data (Fig. 25) or geographic distribution (Fig. 374) are required for accurate identification.

This species can also be distinguished from *I. corrugatum* (from the Eyre Peninsula of South Australia) by the shape of the prolateral clasping spurs on the male tibia I, which are oriented longitudinally (Fig. 258; cf. Fig. 109), and by the shape of the female eye group, which is broadly trapezoidal (Fig. 266; cf. Fig. 117).

#### Description (male holotype).

**Figures 250–259.**
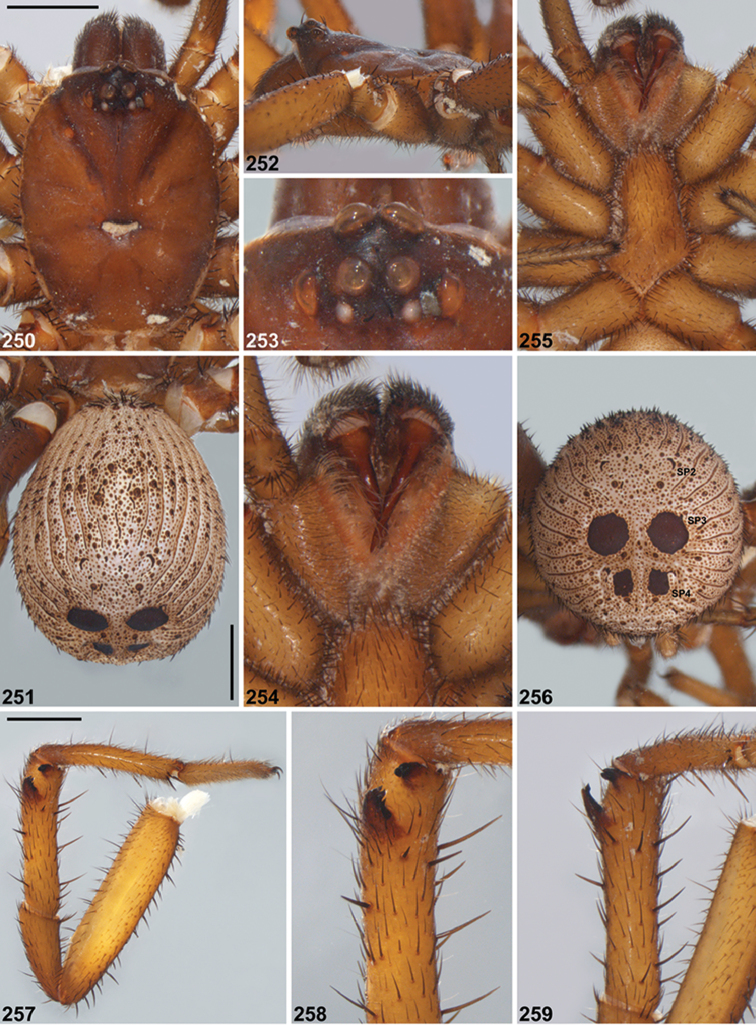
*Idiosoma kopejtkaorum* sp. n., male holotype (WAM T144621) from Snake Gully Nature Reserve (Western Australia; AVW), somatic morphology. **250–251** Carapace and abdomen, dorsal view **252** Carapace, lateral view **253** Eyes, dorsal view **254** Mouthparts, ventral view **255** Sternum, ventral view **256** Abdomen, posterior view **257** Leg I, prolateral view **258** Leg I tibia, clasping spurs, prolateral view **259** Leg I tibia, proventral view. Scale bars 2.0. SP2–4 = sigilla pairs 2–4.

**Figures 260–262.**
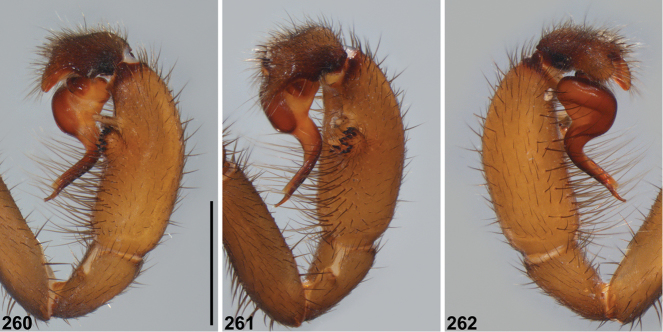
*Idiosoma kopejtkaorum* sp. n., male holotype (WAM T144621) from Snake Gully Nature Reserve (Western Australia; AVW), pedipalp. **260** Retrolateral view **261** Retroventral view **262** Prolateral view. Scale bar 2.0.

Total length 14.1. Carapace 5.9 long, 4.4 wide. Abdomen 7.2 long, 5.6 wide. Carapace (Fig. 250) dark tan and chocolate-brown, with darker ocular region; lateral margins with uniformly-spaced fringe of porrect black setae; fovea procurved. Eye group (Fig. 253) trapezoidal (anterior eye row strongly procurved), 0.7 × as long as wide, PLE–PLE/ALE–ALE ratio 1.9; ALE almost contiguous; AME separated by less than their own diameter; PME separated by 2.6 × their own diameter; PME and PLE separated by slightly more than diameter of PME, PME positioned slightly posterior to level of PLE. Maxillae with field of small cuspules confined to inner corner; labium without cuspules. Abdomen (Figs 251, 256) broadly oval, light beige-brown in dorsal view with lateral sclerotic strips, dorso-lateral corrugations, and scattered dorsal sclerotic spots. Dorsal surface of abdomen (Fig. 251) more heavily setose anteriorly, with assortment of stiff, porrect black setae, each with slightly raised, dark brown sclerotic base. Posterior abdomen strongly sigillate (Figs 251, 256); SP2 sclerites comma-shaped spots; SP3 sclerites very large and circular; SP4 sclerites broadly oval; SP5 obscured. Legs (Figs 257–259) variable shades of dark tan, with light scopulae on tarsi I–II; distal tibia I with pair of large prolateral clasping spurs oriented longitudinally. Leg I: femur 5.8; patella 2.9; tibia 4.1; metatarsus 4.1; tarsus 2.4; total 19.3. Leg I femur–tarsus/carapace length ratio 3.2. Pedipalpal tibia (Figs 260–262) 2.4 × longer than wide; RTA burr-like, with conical basal protuberance and field of retroventral spinules; digital process porrect, unmodified. Cymbium (Figs 260–262) setose, with field of spinules disto-dorsally. Embolus (Figs 260–262) broadly twisted and sharply tapering distally, with prominent longitudinal flange; embolic apophysis absent.

#### Description (female WAM T108519).

**Figures 263–271.**
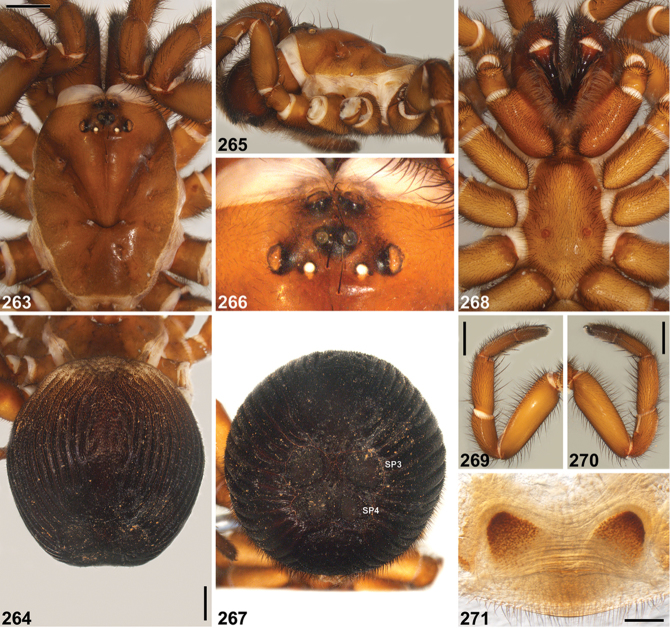
*Idiosoma kopejtkaorum* sp. n., female (WAM T108519) from Charles Darwin Nature Reserve (Western Australia; AVW). **263–264** Carapace and abdomen, dorsal view **265** Carapace, lateral view **266** Eyes, dorsal view **267** Abdomen, posterior view **268** Sternum, ventral view **269** Leg I, prolateral view **270** Leg I, retrolateral view **271** Spermathecae, dorsal view. Scale bars 2.0 (**263–264, 269–270**), 0.5 (***271***). SP3–4 = sigilla pairs 3–4.

Total length 25.4. Carapace 9.1 long, 6.6 wide. Abdomen 12.4 long, 11.7 wide. Carapace (Fig. 263) dark tan, with darker ocular region; fovea procurved. Eye group (Fig. 266) trapezoidal (anterior eye row strongly procurved), 0.6 × as long as wide, PLE–PLE/ALE–ALE ratio 2.2; ALE almost contiguous; AME separated by approximately their own diameter; PME separated by 3.4 × their own diameter; PME and PLE separated by more than diameter of PME, PME positioned in line with level of PLE. Maxillae with field of cuspules confined to inner corner (Fig. 268); labium without cuspules. Abdomen (Figs 264, 267) dark brown-black, corrugate and highly sclerotised, with leathery appearance typical of those species in the ‘sigillate complex’ (see Fig. 25). Posterior face of abdomen (Fig. 267, Key pane 22.1) with truncate ‘shield-like’ morphology; SP3 sclerites very large and circular; SP4 sclerites broadly oval; SP5 obscured by thickened cuticle. Legs (Figs 269–270) variable shades of dark tan; scopulae present on tarsi and metatarsi I–II; tibia I with one stout pro-distal macroseta and row of six longer retroventral macrosetae; metatarsus I with 9 stout macrosetae; tarsus I with distal cluster of short macrosetae. Leg I: femur 5.4; patella 3.4; tibia 3.3; metatarsus 2.7; tarsus 1.9; total 16.7. Leg I femur–tarsus/carapace length ratio 1.8. Pedipalp dark tan, spinose on tibia and tarsus, with thick tarsal scopula. Genitalia (Fig. 271) with pair of short, obliquely angled spermathecae, each bearing dense field of glandular vesicles distally, and more sparsely distributed glandular field sub-distally.

#### Distribution and remarks.

*Idiosoma kopejtkaorum* (formerly known by WAM identification code ‘MYG521’) (Fig. 9), a ‘sigillate complex’ member of the northern *clypeatum*-clade (Fig. 25), is restricted to the north-eastern Wheatbelt bioregion of south-western Western Australia, in a relatively small area surrounding Lake Goorly (Fig. 374). It extends from Charles Darwin Nature Reserve, Mount Gibson, and Mummaloo-Wyebubba Hill in the north, south-west to near Coorow, and south to near Goodlands and the Maya, Buntine and Snake Gully Nature Reserves, all north of Dalwallinu. South of Dalwallinu it is replaced by *I. nigrum*, to the west it is replaced by *I. dandaragan*, and to the north it is replaced by *I. clypeatum*; all four species have a strongly sigillate (sclerotised) abdominal morphology. This distribution seems to be strongly correlated with annual rainfall of 250–300 mm, and red clay soils in the Lake Goorly and southern Lake Moore catchments.

Although first collected by Barbara Main near Mount Gibson in the 1950s, and subsequently collected from a handful of nature reserves during the ‘Salinity Action Plan Survey’ of the late 1990s (Keighery 2004), this species came to prominence during environmental impact assessment surveys conducted in the resource-rich Mummaloo region in 2012. Although it was originally confused with *I. nigrum*, morphological and molecular data were concordant in evidencing two *nigrum*-group species in the Mummaloo/Mount Gibson area. Following detailed molecular analysis of a suite northern *I. nigrum*-like taxa, *I. kopejtkaorum* was designated the working code ‘MYG521’, to distinguish it from what is now known to be *I. formosum*. Under this code name, *I. kopejtkaorum* was also formally assessed in 2017 for threatened species listing under the *Western Australian Wildlife Conservation Act 1950* (see below). Burrows of this species are adorned with a ‘moustache-like’ arrangement of twig-lines (Fig. 21), and males likely wander in search of females in winter.

#### Conservation assessment.

In 2017, *Idiosoma kopejtkaorum* was formally assessed and listed as Endangered (B1ab[iii] + B2ab[iii]) under the *Western Australian Wildlife Conservation Act 1950* (approved 16 January 2018; see W. A. Government Gazette 2018); this assessment incorporated the latest taxonomic, geographic and genetic data summarised in the current study (with a number of additional records also identified subsequently). In the heavily cleared north-eastern Wheatbelt, the threats to the species are manifold (as they are for *I. nigrum*; see above), and in the Mummaloo/Mount Gibson region to the west of Lake Moore, the species is (and will continue to be) at risk from mining and minerals resource development. It has a known extent of occurrence of nearly 4,000 km^2^ [3,571 km^2^], and an area of occupancy within that range of < 500 km^2^. Further close assessment under both Criteria A and B will be crucial to the continued survival of this species.

### Idiosoma kwongan

Taxon classificationAnimaliaAraneaeIdiopidae

Rix & Harvey sp. n.

http://zoobank.org/D17D5265-1612-4B92-A3CB-F70EB4CEEAB3

Figs 25 , 272–281 , 282–284 , 374

#### Type material.

Holotype male. 10 km E. of Green Head (IBRA_GES), Western Australia, Australia, 30°04'S, 114°58'E, laterite heath, 31 August 1982, R.P. McMillan (WAM T27142).

#### Other material examined.

**AUSTRALIA: *Western Australia***: 1 ♂, Eneabba, AMC Minesite, area #5 (IBRA_GES), 29°49'S, 115°16'E, hand collected, 23 November 1987, R.P. McMillan (WAM T27117^DNA_Voucher_NCB_018^); 1 ♂, same data (WAM T27118); 1 ♂, Mount Lesueur [Lesueur National Park] (IBRA_GES), 30°10'S, 115°12'E, 1989, K. Gaull (WAM T139468^DNA_Voucher_NCB_007^).

#### Etymology.

The specific epithet is a noun in apposition, in reference to the distribution of this species in the ‘kwongan’ *Banksia* heathlands of south-western Australia’s northern sandplains.

#### Diagnosis.

*Idiosoma kwongan* is one of seven highly autapomorphic species in the polyphyletic ‘sigillate complex’ (Fig. 25); members of this complex can be distinguished from all other species in the *nigrum*-group from south-western Australia (i.e., *I. formosum*, *I. gardneri*, *I. gutharuka*, *I. incomptum*, *I. intermedium*, *I. jarrah*, *I. mcclementsorum*, *I. mcnamarai* and *I. sigillatum*) by the presence of well-defined lateral sclerotic strips on the male abdomen (e.g., Figs 32, 63, 256), and by the very heavily sclerotised, leathery, ‘shield-like’ morphology of the female abdomen (e.g., Figs 1–3, 9–12, 52, 74, 96). Males of *I. kwongan* can be further distinguished from those of *I. arenaceum* by the shape of the SP4 sclerites, which are not elongate-oval (Fig. 278; cf. Fig. 63); and from *I. clypeatum*, *I. dandaragan*, *I. kopejtkaorum*, *I. nigrum* and *I. schoknechtorum* by the presence of semi-circular lateral indentations adjacent to the SP4 sclerites (Fig. 278, Key pane 13.1; cf. Figs 32, 85, 129, 256, 335). Males of this species can also be distinguished from those of *I. corrugatum* (from the Eyre Peninsula of South Australia) by the shape of the prolateral clasping spurs on tibia I, which are oriented longitudinally (Fig. 280; cf. Fig. 109). Females are unknown.

#### Description (male holotype).

**Figures 272–281.**
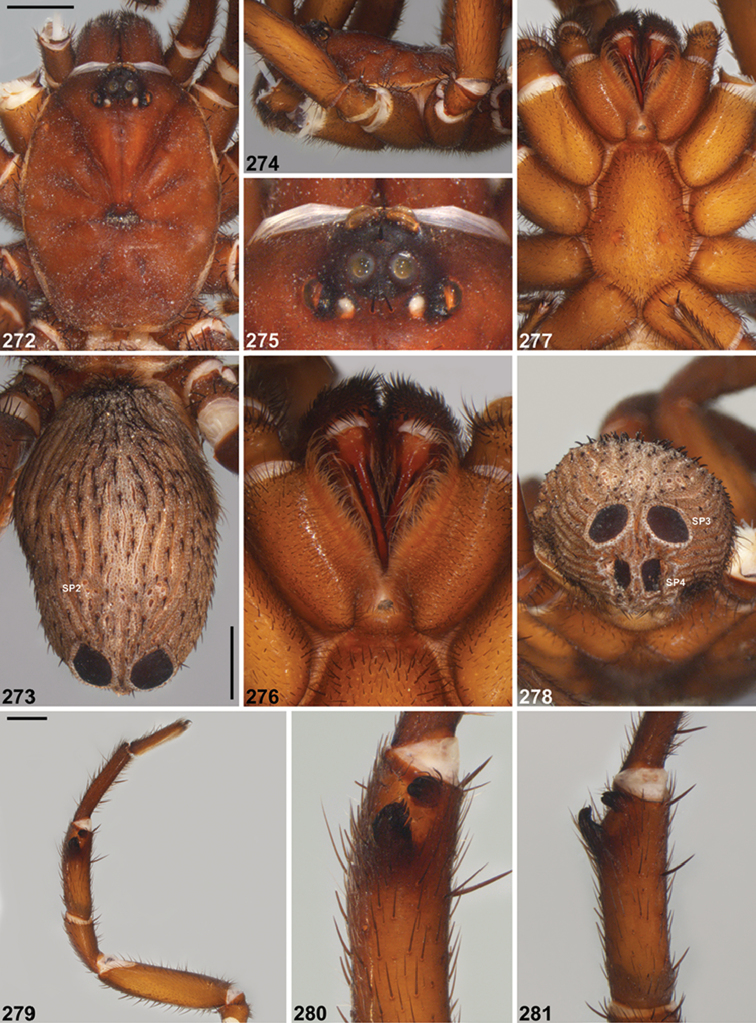
*Idiosoma kwongan* sp. n., male holotype (WAM T27142) from E. of Greenhead (Western Australia; GES), somatic morphology. **272–273** Carapace and abdomen, dorsal view **274** Carapace, lateral view **275** Eyes, dorsal view **276** Mouthparts, ventral view **277** Sternum, ventral view **278** Abdomen, posterior view **279** Leg I, prolateral view **280** Leg I tibia, clasping spurs, prolateral view **281** Leg I tibia, proventral view. Scale bars 2.0. SP2–4 = sigilla pairs 2–4.

**Figures 282–284.**
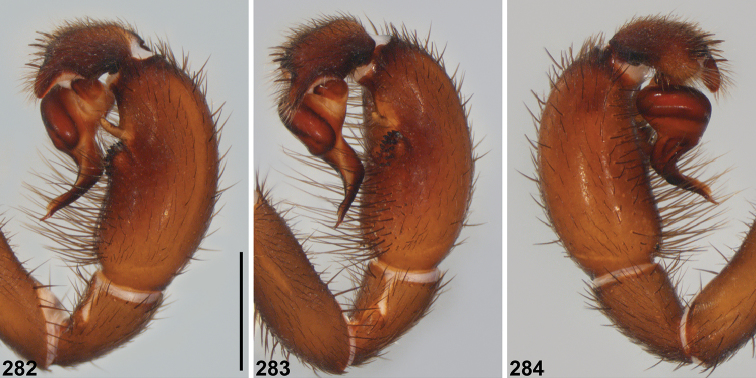
*Idiosoma kwongan* sp. n., male holotype (WAM T27142) from E. of Greenhead (Western Australia; GES), pedipalp. **282** Retrolateral view **283** Retroventral view **284** Prolateral view. Scale bar 2.0.

Total length 18.1. Carapace 7.9 long, 6.0 wide. Abdomen 8.6 long, 5.5 wide. Carapace (Fig. 272) dark tan and chocolate-brown, with darker ocular region; lateral margins with uniformly-spaced fringe of porrect black setae; fovea slightly procurved. Eye group (Fig. 275) trapezoidal (anterior eye row strongly procurved), 0.7 × as long as wide, PLE–PLE/ALE–ALE ratio 2.2; ALE almost contiguous; AME separated by less than their own diameter; PME separated by 3.0 × their own diameter; PME and PLE separated by slightly more than diameter of PME, PME positioned slightly posterior to level of PLE. Maxillae and labium without cuspules. Abdomen (Figs 273, 278) irregularly oval, dark beige-brown in dorsal view with lateral sclerotic strips, dorso-lateral corrugations, and scattered dorsal sclerotic spots. Dorsal surface of abdomen (Fig. 273) more heavily setose anteriorly, with assortment of stiff, porrect black setae, each with slightly raised, dark brown sclerotic base. Posterior abdomen strongly sigillate (Figs 273, 278); SP2 sclerites irregular spots; SP3 sclerites very large and circular; SP4 sclerites broadly oval, each with adjacent semi-circular lateral indentation; SP5 obscured. Legs (Figs 279–281) variable shades of dark tan, with light scopulae on tarsi I–II; distal tibia I with pair of large prolateral clasping spurs oriented longitudinally. Leg I: femur 6.6; patella 3.6; tibia 4.6; metatarsus 4.8; tarsus 3.0; total 22.5. Leg I femur–tarsus/carapace length ratio 2.8. Pedipalpal tibia (Figs 282–284) 2.2 × longer than wide; RTA burr-like, with conical basal protuberance and field of retroventral spinules; digital process porrect, unmodified. Cymbium (Figs 282–284) setose, with field of spinules disto-dorsally. Embolus (Figs 282–284) broadly twisted and sharply tapering distally, with prominent longitudinal flange and triangular (sub-distal) embolic apophysis.

#### Distribution and remarks.

*Idiosoma kwongan* (formerly known by WAM identification code ‘MYG472’) is a poorly known species with an apparently restricted distribution in the southern Geraldton Sandplains bioregion of south-western Western Australia, from Eneabba south to Green Head and the Lesueur National Park (Fig. 374). It is closely related to the three other ‘sigillate complex’ species in the northern *clypeatum*-clade: *I. arenaceum*, *I. clypeatum*, and *I. kopejtkaorum* (Fig. 25). Little is known of the biology of this species, other than that males have been collected wandering in search of females in August and November.

#### Conservation assessment.

*Idiosoma kwongan* has a known extent of occurrence of nearly 500 km^2^ [473 km^2^], although this value is likely to be a severe underestimate as it is based on only three data points, and a relatively large amount of high quality (and poorly surveyed) heathland habitat still exists throughout the southern Geraldton Sandplains. As such, we consider this species data deficient for the purposes of conservation assessment.

### Idiosoma mcclementsorum

Taxon classificationAnimaliaAraneaeIdiopidae

Rix & Harvey sp. n.

http://zoobank.org/35E27F10-28B7-4814-99EC-2DC8622F8D13

Figs 8 , 24 , 25 , 285–294 , 295–297 , 298–306 , 375

Idiosoma ‘*sigillatum*’ Main, 1957b: 439 (in part; cited specimens from SW. of Bolgart, Chittering Lakes, Mogumber, E. of Mogumber and Gillingarra).

#### Type material.

Holotype male. Julimar Conservation Park, Heine Road, site JB10 (IBRA_JAF), Western Australia, Australia, 31°27'03"S, 116°14'42"E, wet pitfall traps, 15 September 1998–4 November 1999, L. King, CALM Survey (WAM T139471).

Paratypes. 1 ♂, same data as holotype (WAM T139472); 1 ♀, Julimar Conservation Park, Mortimer Road (IBRA_JAF), Western Australia, Australia, 31°28'46"S, 116°12'18"E, hand collected, 23 April 2016, M.G. Rix, M.S. Harvey (WAM T139469^DNA_Voucher_NCB_001^).

#### Other material examined.

**AUSTRALIA: *Western Australia***: 1 ♀, 7 juveniles, 9.6 miles SW. of Bolgart (IBRA_JAF), 31°22'S, 116°25'E, 4 June 1953, B.Y. Main (WAM T144861); 1 juvenile, Chittering Lake, just E. of [Great] Northern Highway on Chittering Road (IBRA_JAF), 31°26'S, 116°05'E, 10 October 1953, B.Y. Main (WAM T144863); 1 juvenile, Gillingarra (IBRA_JAF), 30°56'S, 116°03'E, 4 May 1956, B.Y. Main (WAM T144867); 1 ♀, S. of 7 Mile Well Nature Reserve (IBRA_JAF), 31°04'03"S, 116°12'13"E, burrow excavation, 6 March 2015, J. Clark (WAM T139832^DNA_Voucher_NCB_015^); 1 ♀, 5 miles E. of Mogumber in Moore River (IBRA_JAF), 31°01'S, 116°05'E, 4 May 1953, B.Y. Main (WAM T144860); 1 ♀, 10.6 miles N. of Mooliabeenee turnoff from Great Northern Highway (IBRA_JAF), 31°13'S, 116°05'E, 4 March 1953, B.Y. Main (WAM T144857); 1 juvenile, Moore River gorge at Mogumber (IBRA_JAF), 31°02'S, 116°02'E, 4 March 1953, B.Y. Main (WAM T144859); 1 ♂, Toodyay (IBRA_AVW), 31°33'S, 116°28'E, on verandah, January 1997, F. Turnbull (WAM T44388); 1 ♂, Toodyay, Lot 2516 Bindoon Road (IBRA_JAF), 31°33'S, 116°28'E, 1 January 1993, M. & M. Heath (WAM T29779).

#### Etymology.

The specific epithet is named in honour of James and Meredith McClements, in recognition of their generous support for the Western Australian Museum Foundation.

#### Diagnosis.

*Idiosoma mcclementsorum* is one of nine south-western Australian species in the *intermedium*- and *sigillatum*-clades which does not belong to the distinctive ‘sigillate complex’ (Fig. 25); these nine species can be distinguished from those ‘sigillate complex’ taxa (i.e., *I. arenaceum*, *I. clypeatum*, *I. dandaragan*, *I. kopejtkaorum*, *I. kwongan*, *I. nigrum* and *I. schoknechtorum*) by the absence of well-defined lateral sclerotic strips on the male abdomen (e.g., Figs 151, 212, 234), and by the significantly less sclerotised morphology of the female abdomen (which may be strongly corrugate but never leathery and ‘shield-like’) (e.g., Figs 4, 7, 8, 159, 220, 242). Males of *I. mcclementsorum* can be further distinguished from those of *I. gutharuka* and *I. incomptum* by the presence of enlarged (i.e., clearly visible) SP4 sclerites (Fig. 291; cf. Figs 186, 199); from *I. formosum*, *I. gardneri*, *I. intermedium*, *I. mcnamarai* and *I. sigillatum* by the colour of the legs, which are bi-coloured with strongly contrasting bright yellow or orange-yellow femora (Fig. 292; cf. Figs 152, 174, 213, 314, 358); and from *I. jarrah* by the size of the SP3 and SP4 sclerites, which are relatively large (Fig. 291; cf. Fig. 234).

Females can be distinguished from those of *I. formosum*, *I. intermedium*, *I. jarrah* and *I. mcnamarai* by the presence of reinforced, sclerotised ridges on the abdomen, these separated by longitudinal rows of less sclerotised cuticle (Figs 299, 302; cf. Figs 159, 162, 220, 223, 242, 245, 321, 324); and from *I. sigillatum* by the larger size of the SP3 sclerites (Fig. 302; cf. Fig. 368) [NB. females of *I. gardneri*, *I. gutharuka*, and *I. incomptum* are unknown].

This species can also be distinguished from *I. corrugatum* (from the Eyre Peninsula of South Australia) by the shape of the prolateral clasping spurs on the male tibia I, which are oriented longitudinally (Fig. 293; cf. Fig. 109), and by the shape of the female eye group, which is broadly trapezoidal (Fig. 301; cf. Fig. 117).

#### Description (male holotype).

**Figures 285–294.**
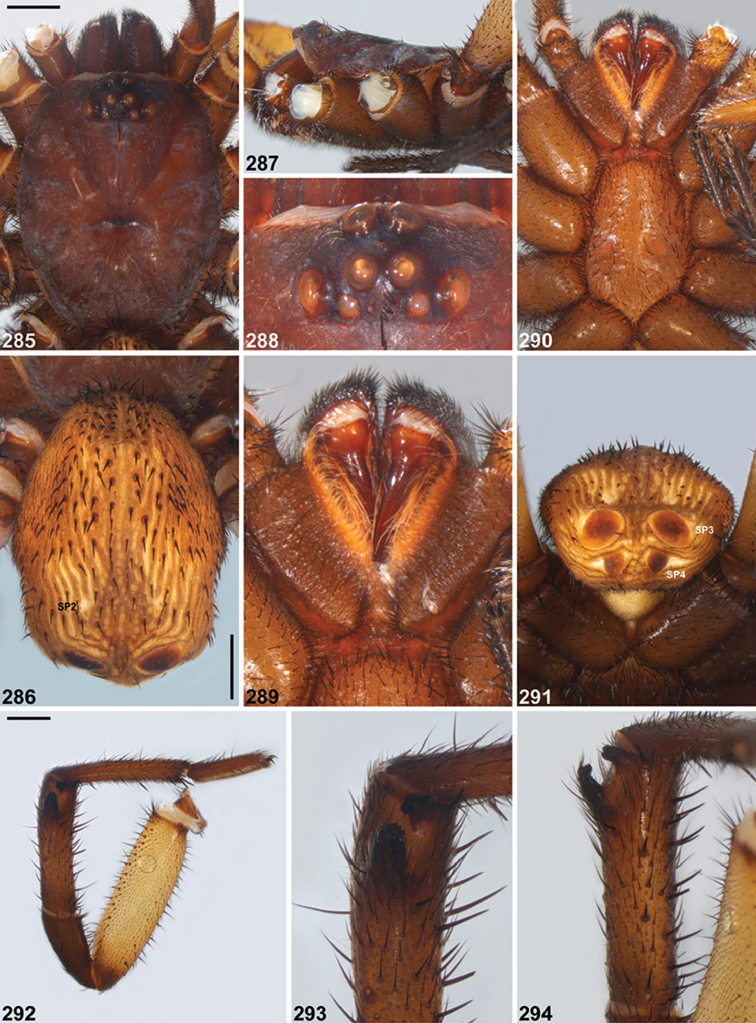
*Idiosoma mcclementsorum* sp. n., male holotype (WAM T139471) from Julimar Conservation Park (Western Australia; JAF), somatic morphology. **285–286** Carapace and abdomen, dorsal view **287** Carapace, lateral view **288** Eyes, dorsal view **289** Mouthparts, ventral view **290** Sternum, ventral view **291** Abdomen, posterior view **292** Leg I, prolateral view **293** Leg I tibia, clasping spurs, prolateral view **294** Leg I tibia, proventral view. Scale bars 2.0. SP2–4 = sigilla pairs 2–4.

**Figures 295–297.**
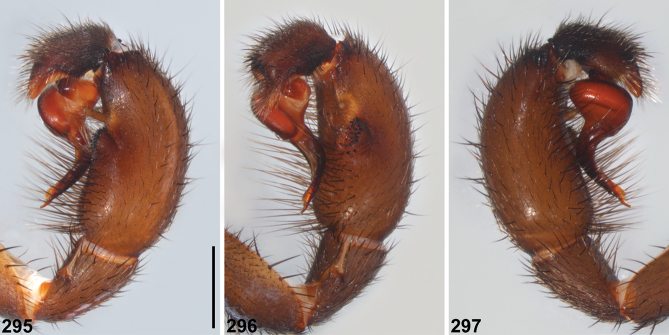
*Idiosoma mcclementsorum* sp. n., male holotype (WAM T139471) from Julimar Conservation Park (Western Australia; JAF), pedipalp. **295** Retrolateral view **296** Retroventral view **297** Prolateral view. Scale bar 2.0.

Total length 21.2. Carapace 9.9 long, 7.7 wide. Abdomen 9.1 long, 6.6 wide. Carapace (Fig. 285) dark chocolate-brown, with darker ocular region; lateral margins with uniformly-spaced fringe of porrect black setae; fovea slightly procurved. Eye group (Fig. 288) trapezoidal (anterior eye row strongly procurved), 0.7 × as long as wide, PLE–PLE/ALE–ALE ratio 2.1; ALE almost contiguous; AME separated by less than their own diameter; PME separated by 2.8 × their own diameter; PME and PLE separated by slightly more than diameter of PME, PME positioned in line with level of PLE. Maxillae and labium without cuspules. Abdomen (Figs 286, 291) irregularly oval, dark tan in dorsal view with paler tan striations, dorso-lateral corrugations, and scattered dorsal sclerotic spots. Dorsal surface of abdomen (Fig. 286) more heavily setose anteriorly, with assortment of stiff, porrect black setae, each with slightly raised, dark brown sclerotic base. Posterior abdomen moderately sigillate (Figs 286, 291); SP2 sclerites irregular spots; SP3 sclerites large and circular, each with unsclerotised triangular ‘corner’ laterally; SP4 sclerites subcircular, each surrounded by chevron-like pad of unsclerotised cuticle laterally; SP5 obscured. Legs (Figs 292–294) bicoloured, variable shades of dark brown on patellae, tibiae, metatarsi and tarsi, and bright tan-yellow on femora, with light scopulae on tarsi I–II; distal tibia I with pair of large prolateral clasping spurs oriented longitudinally. Leg I: femur 8.7; patella 4.6; tibia 6.3; metatarsus 6.6; tarsus 3.5; total 29.7. Leg I femur–tarsus/carapace length ratio 3.0. Pedipalpal tibia (Figs 295–297) 2.2 × longer than wide; RTA burr-like, with conical basal protuberance and field of retroventral spinules; digital process porrect, unmodified. Cymbium (Figs 295–297) setose, with field of spinules disto-dorsally. Embolus (Figs 295–297) broadly twisted and sharply tapering distally, with prominent longitudinal flange and triangular (sub-distal) embolic apophysis.

#### Description (female WAM T139469).

**Figures 298–306.**
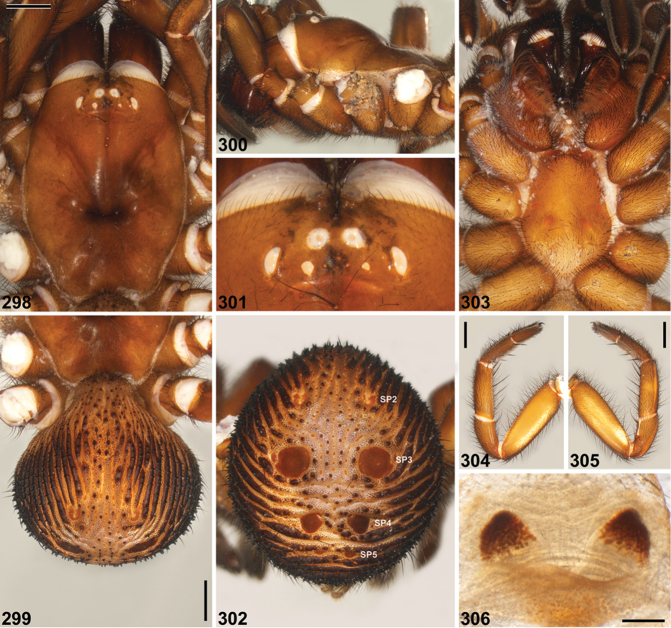
*Idiosoma mcclementsorum* sp. n., female paratype (WAM T139469) from Julimar Conservation Park (Western Australia; JAF). **298–299** Carapace and abdomen, dorsal view **300** Carapace, lateral view **301** Eyes, dorsal view **302** Abdomen, posterior view **303** Sternum, ventral view **304** Leg I, prolateral view **305** Leg I, retrolateral view **306** Spermathecae, dorsal view. Scale bars 2.0 (**298–299, 304–305**), 0.5 (**306**). SP2–5 = sigilla pairs 2–5.

Total length 22.4. Carapace 10.0 long, 7.3 wide. Abdomen 9.6 long, 9.3 wide. Carapace (Fig. 298) dark tan and chocolate-brown, with darker ocular region; fovea procurved. Eye group (Fig. 301) trapezoidal (anterior eye row strongly procurved), 0.6 × as long as wide, PLE–PLE/ALE–ALE ratio 2.5; ALE almost contiguous; AME separated by approximately their own diameter; PME separated by 3.0 × diameter of left PME (right PME disfigured); PME and PLE separated by more than diameter of PME, PME positioned in line with level of PLE. Maxillae with field of cuspules confined to inner corner (Fig. 303); labium without cuspules. Abdomen (Figs 299, 302) truncate, tan, with reinforced dark maroon-black corrugate ridges separated by longitudinal rows of less sclerotised cuticle, each ridge bearing row of modified stout setae. Posterior face of abdomen (Fig. 302) with rudimentary shield-like morphology; SP3 sclerites large and circular with irregular margins; SP4 sclerites subcircular; SP5 sclerites small and oval. Legs (Figs 304–305) variable shades of dark tan; scopulae present on tarsi and metatarsi I–II; tibia I with one stout pro-distal macroseta and row of five longer retroventral macrosetae; metatarsus I with eight stout macrosetae; tarsus I with distal cluster of short macrosetae. Leg I: femur 6.6; patella 4.0; tibia 4.2; metatarsus 3.2; tarsus 2.4; total 20.4. Leg I femur–tarsus/carapace length ratio 2.0. Pedipalp dark tan, spinose on tibia and tarsus, with thick tarsal scopula. Genitalia (Fig. 306) with pair of short, rounded-subtriangular spermathecae, each bearing dense field of glandular vesicles distally, and more sparsely distributed glandular field sub-distally.

#### Distribution and remarks.

*Idiosoma mcclementsorum* (formerly known by WAM identification code ‘MYG474’) (Fig. 8), a member of the yellow legs-clade within the diverse *sigillatum*-clade (Fig. 25), is a rare species with a highly restricted distribution in the northern Jarrah Forest bioregion of south-western Western Australia, from Chittering Lakes, Julimar, and Toodyay north to Gillingarra. South of the Avon Valley it is replaced by its closely related sister species *I. jarrah*, both of which are characterised by yellow leg femora in males (Figs 235, 292). Burrows are adorned with a ‘moustache-like’ arrangement of twig-lines (Fig. 24), and have been found on sandy substrates overlaying laterite. Both males from Toodyay were collected in January, and both males from Julimar were collected between September and November, suggesting that this species may be an unusual spring and summer breeder.

#### Conservation assessment.

*Idiosoma mcclementsorum* has a known extent of occurrence (EOO) of nearly 1,500 km^2^ [1,347 km^2^], although this value is possibly a slight underestimate. The area of occupancy within that range is difficult to estimate, but is unlikely to be larger than 100 km^2^. Given: (i) this geographic range; (ii) the sampling effort that has occurred in surrounding areas as a result of a major biotic survey (see Keighery 2004) and a long history of incidental collecting; (iii) the occurrence of the species at < 10 severely fragmented sites; and (iv) the continuing decline in the area, extent and/or quality of habitat north-east of Perth, this species is considered Endangered (B1ab[iii] + B2ab[iii]). Further close assessment under both Criteria A and B will be crucial to the continued survival of this species.

### Idiosoma mcnamarai

Taxon classificationAnimaliaAraneaeIdiopidae

Rix & Harvey sp. n.

http://zoobank.org/8D6E2EE6-E357-45A1-8CC2-287925E7A180

Figs 25 , 307–316 , 317–319 , 320–328 , 375

#### Type material.

Holotype male. Trayning (IBRA_AVW), Western Australia, Australia, 31°06'S, 117°47'E, 1 July 1992, A. Dugand (WAM T26107^DNA_Voucher_NCB_009^).

#### Other material examined.

**AUSTRALIA: *Western Australia***: 1 ♂, Bruce Rock-Doodlakine Road, site KL4 (IBRA_AVW), 31°51'26"S, 118°06'14"E, wet pitfall traps, 30 October 1997–22 May 1998, P. Van Heurck, N. Guthrie, CALM Survey (WAM T139518^DNA_Voucher_NCB_008^); 1 ♀, 7 km. N. of Cleary (IBRA_AVW), 30°22'S, 117°39'E, 22 July 1983, B.Y. Main (WAM T139496^DNA_Voucher_NCB_014^); 1 ♀, same data except at water catchment, 16 July 1984 (WAM T144848); 1 ♀, same data (WAM T144849); 1 ♀, same data (WAM T144850); 1 ♀, 30.7 km N. of Cleary on Paynes Find Road (IBRA_AVW), 30°10'S, 117°42'E, 23 July 1983, B.Y. Main (WAM T144846); 1 ♀, same data (WAM T144847); 1 ♀, Dajoing Rock (IBRA_AVW), 30°26'S, 118°04'E, 17 March 1985, B.Y. Main (WAM T144853); 1 ♂, East Yorkrakine Nature Reserve, site EYRJ1 (IBRA_AVW), 31°23'S, 117°40'E, wet pitfall traps, 24 July–3 August 1989, G. Friend et al. (WAM T44169).

#### Etymology.

The specific epithet is a named in honour of the late Keiran McNamara (1954–2013), in recognition of his considerable efforts in securing critical funding for the Salinity Action Plan Survey (later ‘State Salinity Strategy’) of the Western Australian agricultural zone (run by the then Department of Conservation and Land Management from 1997–2000), which resulted in the collection of this and numerous other species of *Idiosoma*.

#### Diagnosis.

*Idiosoma mcnamarai* is one of nine south-western Australian species in the *intermedium*- and *sigillatum*-clades which does not belong to the distinctive ‘sigillate complex’ (Fig. 25); these nine species can be distinguished from those ‘sigillate complex’ taxa (i.e., *I. arenaceum*, *I. clypeatum*, *I. dandaragan*, *I. kopejtkaorum*, *I. kwongan*, *I. nigrum* and *I. schoknechtorum*) by the absence of well-defined lateral sclerotic strips on the male abdomen (e.g., Figs 151, 212, 234), and by the significantly less sclerotised morphology of the female abdomen (which may be strongly corrugate but never leathery and ‘shield-like’) (e.g., Figs 4, 7, 8, 159, 220, 242). Males of *I. mcnamarai* can be further distinguished from those of *I. gutharuka* and *I. incomptum* by the presence of enlarged (i.e., clearly visible) SP4 sclerites (Fig. 313; cf. Figs 186, 199); from *I. jarrah* and *I. mcclementsorum* by the colour of the legs, which do not have strongly contrasting bright yellow or orange-yellow femora (Fig. 314; cf. Figs 235, 292); from *I. gardneri* and *I. sigillatum* by the absence of well-defined dorso-lateral abdominal corrugations or striations (Figs 308, 313; cf. Figs 168, 173, 352, 357, Key pane 9.1); from *I. formosum* by the shape of tibia I, which is longer (with the prolateral clasping spurs occupying the distal third of the segment) (Fig. 314; cf. Fig. 152), and by the colour of the abdomen, which is more uniformly coloured dorsally (Figs 308, 313; cf. Figs 146, 151); and from *I. intermedium* by the larger SP4 sclerites (Fig. 313; cf. Fig. 212), and by the morphology of the SP3 sclerites, each of which have an anteriorly directed triangular corner laterally (as opposed to a laterally or postero-laterally directed triangular corner) (Fig. 313, Key panes 12.1, 12.2; cf. Fig. 212, Key pane 12.3)

Females can be distinguished from those of *I. mcclementsorum* and *I. sigillatum* by the absence of reinforced, sclerotised ridges on the abdomen (Figs 321, 324; cf. Figs 299, 302, 365, 368); from *I. intermedium* and *I. jarrah* by the size of the SP4 sclerites, which are significantly larger than the SP2 sclerites (Fig. 324; cf. Figs 223, 245); and from *I. formosum* by the colour of the abdomen, which is more uniformly coloured dorsally (Figs 321, 324; cf. Figs 159, 162) [NB. females of *I. gardneri*, *I. gutharuka* and *I. incomptum* are unknown].

This species can also be distinguished from *I. corrugatum* (from the Eyre Peninsula of South Australia) by the shape of the prolateral clasping spurs on the male tibia I, which are oriented longitudinally (Fig. 315; cf. Fig. 109), and by the shape of the female eye group, which is broadly trapezoidal (Fig. 323; cf. Fig. 117).

#### Description (male holotype).

**Figures 307–316.**
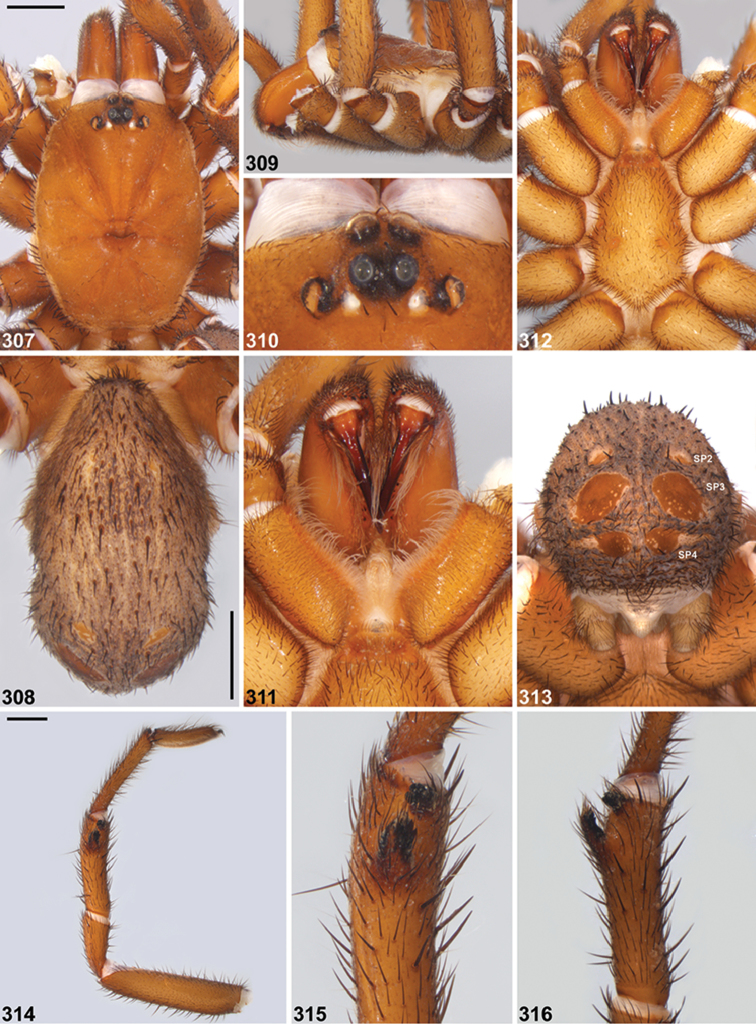
*Idiosoma mcnamarai* sp. n., male holotype (WAM T26107) from Trayning (Western Australia; AVW), somatic morphology. **307–308** Carapace and abdomen, dorsal view **309** Carapace, lateral view **310** Eyes, dorsal view **311** Mouthparts, ventral view **312** Sternum, ventral view **313** Abdomen, posterior view **314** Leg I, prolateral view **315** Leg I tibia, clasping spurs, prolateral view **316** Leg I tibia, proventral view. Scale bars 2.0. SP2–4 = sigilla pairs 2–4.

**Figures 317–319.**
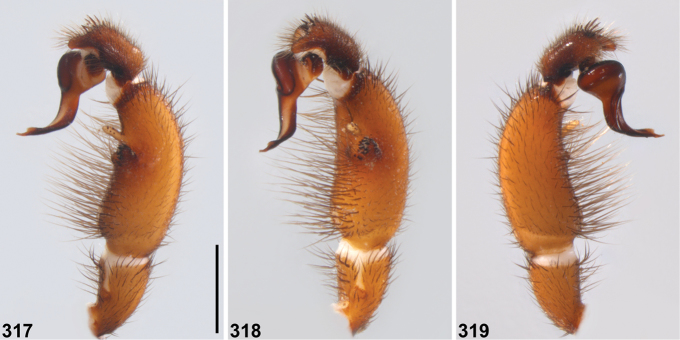
*Idiosoma mcnamarai* sp. n., male holotype (WAM T26107) from Trayning (Western Australia; AVW), pedipalp. **317** Retrolateral view **318** Retroventral view **319** Prolateral view. Scale bar 2.0.

Total length 18.4. Carapace 8.3 long, 6.0 wide. Abdomen 7.2 long, 4.1 wide. Carapace (Fig. 307) tan, with darker ocular region; lateral margins with uniformly spaced fringe of porrect black setae; fovea procurved. Eye group (Fig. 310) trapezoidal (anterior eye row strongly procurved), 0.6 × as long as wide, PLE–PLE/ALE–ALE ratio 2.1; ALE almost contiguous; AME separated by less than their own diameter; PME separated by 3.3 × their own diameter; PME and PLE separated by slightly more than diameter of PME, PME positioned in line with level of PLE. Maxillae and labium without cuspules. Abdomen (Figs 308, 313) irregularly oval, grey-brown in dorsal view with tan mottling and assortment of stiff, porrect black setae, each with slightly raised, dark brown sclerotic base. Posterior abdomen moderately sigillate (Figs 308, 313); SP2 sclerites irregular spots; SP3 sclerites circular, each with unsclerotised, anteriorly directed triangular ‘corner’ laterally; SP4 sclerites broadly oval, each surrounded by chevron-like pad of unsclerotised cuticle laterally; SP5 obscured. Legs (Figs 314–316) variable shades of tan, with light scopulae on tarsi I–II; distal tibia I with pair of large prolateral clasping spurs oriented longitudinally. Leg I: femur 7.2; patella 3.5; tibia 5.0; metatarsus 5.2; tarsus 3.2; total 24.1. Leg I femur–tarsus/carapace length ratio 2.9. Pedipalpal tibia (Figs 317–319) 2.5 × longer than wide; RTA burr-like, with conical basal protuberance and field of retroventral spinules; digital process porrect, unmodified. Cymbium (Figs 317–319) setose, with field of spinules disto-dorsally. Embolus (Figs 317–319) broadly twisted and sharply tapering distally, with prominent longitudinal flange and triangular (sub-distal) embolic apophysis.

#### Description (female WAM T139496).

**Figures 320–328.**
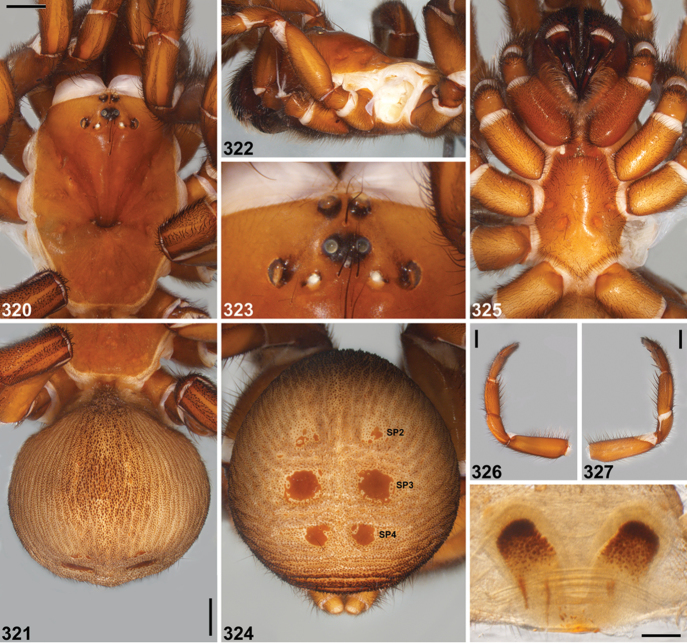
*Idiosoma mcnamarai* sp. n., female (WAM T139496) from N. of Cleary (Western Australia; AVW). **320–321** Carapace and abdomen, dorsal view **322** Carapace, lateral view **323** Eyes, dorsal view **324** Abdomen, posterior view **325** Sternum, ventral view **326** Leg I, prolateral view **32** Leg I, retrolateral view **328** Spermathecae, dorsal view. Scale bars 2.0 (**320–321, 326–327**), 0.5 (**328**). SP2–4 = sigilla pairs 2–4.

Total length 26.0. Carapace 10.8 long, 7.4 wide. Abdomen 10.6 long, 10.9 wide. Carapace (Fig. 320) tan, with darker ocular region; fovea procurved. Eye group (Fig. 323) trapezoidal (anterior eye row strongly procurved), 0.6 × as long as wide, PLE–PLE/ALE–ALE ratio 3.0; ALE almost contiguous; AME separated by approximately their own diameter; PME separated by 4.7 × their own diameter; PME and PLE separated by more than diameter of PME, PME positioned in line with level of PLE. Maxillae with field of cuspules confined to inner corner (Fig. 325); labium without cuspules. Abdomen (Figs 321, 324) broadly oval and somewhat truncate posteriorly, beige-brown in dorsal view, with numerous stout setae on sclerotic bases and scattered sclerotic spots; longest stout setae clustered along median cardiac region. Posterior abdomen moderately sigillate (Figs 321, 324); SP2 sclerites irregular spots; SP3 sclerites subcircular with irregular margins, each surrounded by pad of unsclerotised cuticle; SP4 sclerites subcircular with irregular margins, each surrounded by chevron-like pad of unsclerotised cuticle laterally; SP5 obscured; posterior margin of abdomen weakly corrugate, with rows of modified stout setae. Legs (Figs 326–327) variable shades of tan; scopulae present on tarsi and metatarsi I–II; tibia I with one stout pro-distal macroseta and row of five longer retroventral macrosetae; metatarsus I with eight stout macrosetae; tarsus I with distal cluster of short macrosetae. Leg I: femur 6.7; patella 4.2; tibia 4.0; metatarsus 3.2; tarsus 2.4; total 20.5. Leg I femur–tarsus/carapace length ratio 1.9. Pedipalp tan, spinose on tibia and tarsus, with thick tarsal scopula. Genitalia (Fig. 328) with pair of obliquely angled spermathecae, each bearing dense field of glandular vesicles distally, and more sparsely distributed glandular field sub-distally.

#### Distribution and remarks.

*Idiosoma mcnamarai* (formerly known by WAM identification code ‘MYG520’), a member of the diverse *sigillatum*-clade (Fig. 25), is a rare species with a restricted distribution in the central-eastern Wheatbelt bioregion of south-western Western Australia, from Bruce Rock north to Lake Moore (Fig. 375). Its range follows a thin longitudinal band, likely associated with substrate and rainfall characteristics just west of the red soil transition. North of Cleary, this distribution overlaps the southern extent of the range of its closely related sister species *I. formosum*, and in the Durokoppin/East Yorkrakine region it overlaps with *I. nigrum*. Like *I. formosum*, *I. intermedium*, and *I. jarrah*, *I. mcnamarai* exhibits a transitional morphology between largely unmodified species in the *intermedium*-lineage’ (i.e. *I. incomptum* and *I. gutharuka*), and the more obviously phragmotic taxa in the *clypeatum*- and *sigillatum*-clades. Little is known of the biology of this species, other than that males have been collected wandering in search of females in winter.

#### Conservation assessment.

*Idiosoma mcnamarai* has a known extent of occurrence of nearly 6,000 km^2^ [5,862 km^2^], and an area of occupancy within that range of < 500 km^2^. Given: (i) this geographic range; (ii) the sampling effort that has occurred in surrounding areas as a result of a major biotic survey (see Keighery 2004); (iii) the occurrence of the species at < 10 severely fragmented sites; and (iv) the continuing decline in the area, extent and/or quality of habitat in the central-eastern Wheatbelt agricultural zone (Laurance et al. 2011), this species is considered Endangered (B1ab[iii] + B2ab[iii]). Further close assessment under both Criteria A and B will be crucial to the continued survival of this species.

### Idiosoma schoknechtorum

Taxon classificationAnimaliaAraneaeIdiopidae

Rix & Harvey sp. n.

http://zoobank.org/0ABF532A-049D-41BB-B63F-455F657420DE

Figs 10 , 25 , 18 , 329–338 , 339–341 , 342–350 , 374

Idiosoma ‘*nigrum*’ Main, 1952: 135 (in part; cited specimens from Mount Dick and Northam). Main 1957b: 440 (in part; cited specimens from Beverley, Connolly Gully, Flint Gully, Jimperding Hill, Mount Dick, Northam, Quairading, Toodyay and E. of York).

#### Type material.

Holotype male. Beverley (IBRA_AVW), Western Australia, Australia, 32°06'S, 116°55'E, May 1954, H.W. Norris (WAM T139512).

#### Other material examined.

**AUSTRALIA: *Western Australia***: 1 ♀, Bakers Hill (IBRA_JAF), 31°45'S, 116°27'E, 1 October 1966, G.H. Lowe (WAM T27116); 1 ♀, ‘Connolly Gully’, 26 miles NW. of Brookton, 38 miles S. of Karragullen turnoff on Kelmscott-Brookton Road (IBRA_JAF), 32°20'S, 116°38'E, 18 May 1955, B.Y. Main (WAM T144801); 1 juvenile, Cunaring Hill, 2 miles off Brookton-Kelmscott Road on track S. to Williams (IBRA_AVW), 32°25'S, 116°48'E, 5 March 1959, B.Y. Main (WAM T144841); 1 ♀, Jimperding Hill, Avon Valley (IBRA_JAF), 31°35'S, 116°20'E, 17 November 1952, B.Y. Main (WAM T144776); 1 ♀, same data (WAM T144777); 1 ♀, same data (WAM T144778); 1 juvenile, 32 miles S. of Karragullen turnoff on Kelmscott-Brookton Road (IBRA_JAF), 32°16'S, 116°24'E, 18 May 1955, B.Y. Main (WAM T144800); 1 ♂, Meckering (IBRA_AVW), 31°37'S, 117°00'E, 11 May 1963, H.E. Lamont (WAM T27121); 1 ♀, Meenaar Nature Reserve, 9 km E. of Grass Valley, off Great Eastern Highway (IBRA_AVW), 31°38'19"S, 116°53'41"E, hand collected, under mallee in woodland, 15 July 2014, M.G. Rix, M.S. Harvey (WAM T133465^DNA_Voucher_154^); 1 ♀, 105 Mile Peg, Quairading Road, E. of Perth (IBRA_AVW), 32°01'S, 117°26'E, 14 January 1954, M. Littlejohn (WAM T144782); 1 ♀, Mount Dick (via Northam) (IBRA_AVW), 31°35'S, 116°42'E, 17 June 1952, B.Y. Main (WAM T144763); 1 juvenile, same data (WAM T144803); 1 ♀, same data (WAM T144804); 1 ♀, Northam (IBRA_AVW), 31°39'S, 116°40'E, 5 June 1974, D. Martin (WAM T27122); 1 ♀, same locality data, June 1952, C.G. Jessup (WAM T144772); 1 juvenile, Red Hill Road, near Jimperding, several miles W. of Toodyay (IBRA_JAF), 31°35'S, 116°20'E, 21 June 1952, B.Y. Main (WAM T144771); 1 juvenile, about 6 miles W. of Toodyay on Red Hill Road (IBRA_AVW), 31°37'S, 116°24'E, 17 November 1952, B.Y. Main (WAM T144774); 1 juvenile, same data (WAM T144775); 1 juvenile, same data (WAM T144809); 1 ♂, Westdale, S. side of road (IBRA_JAF), 32°19'S, 116°37'E, pitfall trap, 20 July 1969, B.Y. Main (WAM T139513); 1 ♀, Wongamine Nature Reserve (IBRA_AVW), 31°29'S, 116°34'E, September 1990, B.Y. Main (WAM T144845); 1 ♀, York (IBRA_AVW), 31°53'S, 116°46'E, 3 May 1971, P.A. Cray (WAM T27124); 1 ♀, same data except 1 January 1974, N. Giles (WAM T27125); 1 ♀, 4.2 km SW. of York (IBRA_AVW), 31°55'03"S, 116°44'30"E, dug from burrow, 17 May 2016, D. Schoknecht (WAM T140765^DNA_Voucher_NCB_004^); 1 juvenile, 18 miles E. of York (IBRA_AVW), 31°52'S, 117°06'E, 6 March 1955, B.Y. Main (WAM T144826).

#### Etymology.

The specific epithet is named in honour of Daniel and Noel Schoknecht, for collecting an important (and sequenceable) female specimen of this species on their property SW. of York.

#### Diagnosis.

*Idiosoma schoknechtorum* is one of seven highly autapomorphic species in the polyphyletic ‘sigillate complex’ (Fig. 25); members of this complex can be distinguished from all other species in the *nigrum*-group from south-western Australia (i.e., *I. formosum*, *I. gardneri*, *I. gutharuka*, *I. incomptum*, *I. intermedium*, *I. jarrah*, *I. mcclementsorum*, *I. mcnamarai* and *I. sigillatum*) by the presence of well-defined lateral sclerotic strips on the male abdomen (e.g., Figs 32, 63, 256), and by the very heavily sclerotised, leathery, ‘shield-like’ morphology of the female abdomen (e.g., Figs 1–3, 9–12, 52, 74, 96). Males of *I. schoknechtorum* can be further distinguished from those of *I. arenaceum* by the shape of the SP4 sclerites, which are not elongate-oval (Fig. 335; cf. Fig. 63); from *I. kwongan* by the absence of semi-circular lateral indentations adjacent to the SP4 sclerites (Fig. 335; cf. Fig. 278, Key pane 13.1); from *I. clypeatum* and *I. kopejtkaorum* by the presence of a prominent sub-distal embolic apophysis (Key pane 14.1; cf. Key panes 14.2, 14.3); and from *I. nigrum* by the more heavily setose morphology of the dorsal abdomen (Fig. 330; cf. Fig. 27), and by the shape of the SP4 sclerites, which are circular or oval (Fig. 335; cf. Fig. 32). By our assessment, males of *I. schoknechtorum* are morphologically indistinguishable from those of *I. dandaragan*; molecular data (Fig. 25) or geographic distribution (Fig. 374) are required for accurate identification.

Females can be distinguished from those of *I. arenaceum* by the shape of the SP4 sclerites, which are not elongate-oval (Fig. 343, Key pane 23.3; cf. Fig. 74, Key pane 21.1); from *I. clypeatum* and *I. kopejtkaorum* by the size of the SP4 sclerites, which are greater than half the size of the SP3 sclerites (Fig. 343, Key pane 23.3; cf. Figs 96, 267, Key panes 22.1, 22.2); and from *I. nigrum* by the shape of the SP4 sclerites, which are circular or broadly oval (Fig. 343, Key pane 23.3; cf. Figs 43, 52, Key pane 23.1), and by the presence of well-defined SP5 sclerites (Fig. 343, Key pane 23.3; cf. Figs 43, 52, Key pane 23.1) [NB. females of *I. kwongan* are unknown]. By our assessment, females of *I. schoknechtorum* are morphologically indistinguishable from those of *I. dandaragan*; molecular data (Fig. 25) or geographic distribution (Fig. 374) are required for accurate identification.

This species can also be distinguished from *I. corrugatum* (from the Eyre Peninsula of South Australia) by the shape of the prolateral clasping spurs on the male tibia I, which are oriented longitudinally (Fig. 337; cf. Fig. 109), and by the shape of the female eye group, which is broadly trapezoidal (Fig. 345; cf. Fig. 117).

#### Description (male holotype).

**Figures 329–338.**
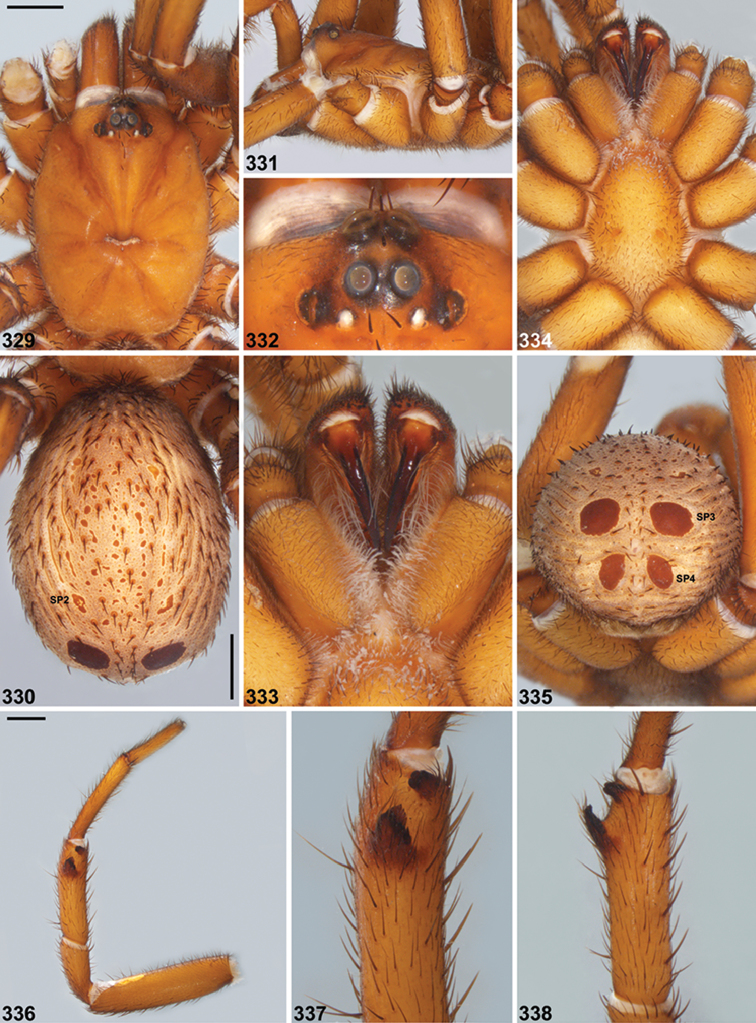
*Idiosoma schoknechtorum* sp. n., male holotype (WAM T139512) from Beverley (Western Australia; AVW), somatic morphology. **329–330** Carapace and abdomen, dorsal view **331** Carapace, lateral view **332** Eyes, dorsal view **333** Mouthparts, ventral view **334** Sternum, ventral view **335** Abdomen, posterior view **336** Leg I, prolateral view **337** Leg I tibia, clasping spurs, prolateral view **338** Leg I tibia, proventral view. Scale bars 2.0. SP2–4 = sigilla pairs 2–4.

**Figures 339–341.**
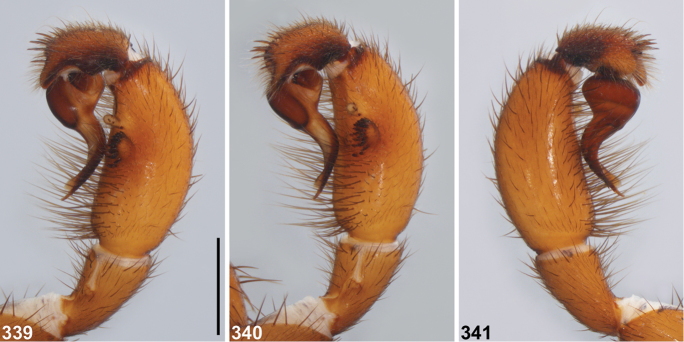
*Idiosoma schoknechtorum* sp. n., male holotype (WAM T139512) from Beverley (Western Australia; AVW), pedipalp. **339** Retrolateral view **340** Retroventral view **341** Prolateral view. Scale bar 2.0.

Total length 20.6. Carapace 8.6 long, 6.4 wide. Abdomen 9.3 long, 6.7 wide. Carapace (Fig. 329) tan, with darker ocular region; lateral margins with uniformly spaced fringe of porrect black setae; fovea procurved. Eye group (Fig. 332) trapezoidal (anterior eye row strongly procurved), 0.7 × as long as wide, PLE–PLE/ALE–ALE ratio 2.1; ALE almost contiguous; AME separated by less than their own diameter; PME separated by 3.7 × their own diameter; PME and PLE separated by slightly more than diameter of PME, PME positioned in line with level of PLE. Maxillae with field of small cuspules confined to inner corner; labium without cuspules. Abdomen (Figs 330, 335) broadly oval, beige-brown in dorsal view with lateral sclerotic strips, dorso-lateral corrugations, and scattered dorsal sclerotic spots. Dorsal surface of abdomen (Fig. 330) more heavily setose anteriorly, with assortment of stiff, porrect black setae, each with slightly raised, dark brown sclerotic base. Posterior abdomen strongly sigillate (Figs 330, 335); SP2 sclerites irregular, comma-shaped spots; SP3 sclerites very large and circular; SP4 sclerites broadly oval; SP5 obscured. Legs (Figs 336–338) variable shades of tan, with light scopulae on tarsi I–II; distal tibia I with pair of large prolateral clasping spurs oriented longitudinally. Leg I: femur 7.7; patella 3.9; tibia 5.2; metatarsus 5.5; tarsus 3.4; total 25.6. Leg I femur–tarsus/carapace length ratio 3.0. Pedipalpal tibia (Figs 339–341) 2.3 × longer than wide; RTA burr-like, with conical basal protuberance and field of retroventral spinules; digital process porrect, unmodified. Cymbium (Figs 339–341) setose, with field of spinules disto-dorsally. Embolus (Figs 339–341) broadly twisted and sharply tapering distally, with prominent longitudinal flange and triangular (sub-distal) embolic apophysis.

#### Description (female WAM T140765).

**Figures 342–350.**
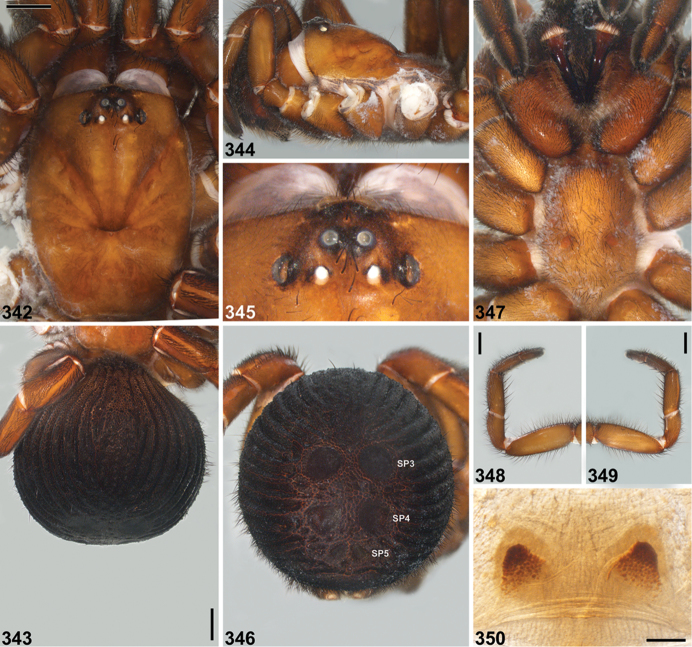
*Idiosoma schoknechtorum* sp. n., female (WAM T140765) from SW. of York (Western Australia; AVW). **342–343** Carapace and abdomen, dorsal view **344** Carapace, lateral view **345** Eyes, dorsal view **346** Abdomen, posterior view **347** Sternum, ventral view **348** Leg I, prolateral view **349** Leg I, retrolateral view **350** Spermathecae, dorsal view. Scale bars 2.0 (**342–343, 348–349**), 0.5 (**350**). SP3–5 = sigilla pairs 3–5.

Total length 25.8. Carapace 10.4 long, 7.5 wide. Abdomen 12.2 long, 12.1 wide. Carapace (Fig. 342) dark tan, with darker ocular region; fovea procurved. Eye group (Fig. 345) trapezoidal (anterior eye row strongly procurved), 0.6 × as long as wide, PLE–PLE/ALE–ALE ratio 2.4; ALE almost contiguous; AME separated by approximately their own diameter; PME separated by 2.75 × their own diameter; PME and PLE separated by more than diameter of PME, PME positioned in line with level of PLE. Maxillae with field of cuspules confined to inner corner (Fig. 347); labium without cuspules. Abdomen (Figs 343, 346) dark brown-black, corrugate and highly sclerotised, with leathery appearance typical of those species in the ‘sigillate complex’ (see Fig. 25). Posterior face of abdomen (Fig. 346, Key pane 23.3) with truncate ‘shield-like’ morphology; SP3 sclerites very large and circular; SP4 sclerites broadly oval; SP5 sclerites small and oval. Legs (Figs 348–349) variable shades of dark tan; scopulae present on tarsi and metatarsi I–II; tibia I with one stout pro-distal macroseta and row of five longer retroventral macrosetae; metatarsus I with eight stout macrosetae; tarsus I with distal cluster of short macrosetae. Leg I: femur 6.3; patella 4.0; tibia 3.9; metatarsus 3.1; tarsus 2.3; total 19.6. Leg I femur–tarsus/carapace length ratio 1.9. Pedipalp dark tan, spinose on tibia and tarsus, with thick tarsal scopula. Genitalia (Fig. 350) with pair of short, subtriangular spermathecae, each bearing dense field of glandular vesicles distally, and more sparsely distributed glandular field sub-distally.

#### Distribution and remarks.

*Idiosoma schoknechtorum* (formerly known by WAM identification code ‘MYG518’) (Fig. 10), a ‘sigillate complex’ member of the diverse *sigillatum*-clade (Fig. 25), is a recently recognised species with a restricted distribution in the central-western Wheatbelt and north-eastern Jarrah Forest bioregion of south-western Western Australia (east of Perth) (Fig. 374). Long confused with its closely related sister species *I. nigrum*, the range of *I. schoknechtorum* extends from near Toodyay, the Wongamine Nature Reserve and Meckering in the north, south to near Jarrahdale and Westdale, and east to near Quairading. Within this range it is the only species with a highly sigillate (sclerotised) abdominal morphology, although the distribution of *I. nigrum* closely approaches that of *I. schoknechtorum* north of Meckering and Wongamine.

This species is very similar to *I. nigrum* and *I. dandaragan* in most respects, and burrows are adorned with a typical ‘moustache-like’ arrangement of twig-lines (Fig. 18). At some localities (e.g., Grass Valley) it may be locally common, although little is known of its biology, and surprisingly few specimens have been collected despite its proximity to Perth. Males have been collected wandering in search of females in May and July.

#### Conservation assessment.

*Idiosoma schoknechtorum* has a known extent of occurrence (EOO) of nearly 5,500 km^2^ [5,296 km^2^]. The area of occupancy within that range is difficult to estimate given the distributional division between the Jarrah Forest bioregion and the more heavily cleared Wheatbelt bioregion, but is certainly less than 2,000 km^2^. Given: (i) this geographic range; (ii) the sampling effort that has occurred in surrounding areas as a result of a major biotic survey (see Keighery 2004) and a long history of incidental collecting; (iii) the occurrence of the species at a number of severely fragmented sites; and (iv) the continuing decline in the area, extent and/or quality of habitat in the western Wheatbelt agricultural zone (Laurance et al. 2011), this species is considered Vulnerable (B1ab[iii] + B2ab[iii]). Further close assessment under both Criteria A and B is warranted in the future.

### Idiosoma sigillatum

Taxon classificationAnimaliaAraneaeIdiopidae

(O. P.-Cambridge, 1870)

Figs 4–6 , 16 , 17 , 25 , 351–360 , 361–363 , 364–372 , 373 , 375

Idiops sigillatus O. P.-Cambridge, 1870: 105, pl. 8, fig. 2A–H.
Idiosoma sigillatum (O. P-Cambridge): Ausserer 1871: 150.
Acanthodon sigillatum (O. P.-Cambridge): Simon 1892: 91.
Idiosoma sigillatum (O. P.-Cambridge): Pocock 1897: 109. Simon 1903: 901, fig. 1053. Main 1952: 131, figs 1A–C, 2A. Main 1957b: 438, fig. 14C (in part; cited specimens from Floreat Park, Inglewood, Kings Park, West Midland, Mount Hawthorn, N. of Muchea, Naval Base, Rockingham and Rottnest Island). Main 1964: 32, figs A–D, F. Main 1985: 14, figs 26, 190, 216. Castalanelli et al. 2014: 383. Rix et al. 2017d: 593, figs 83, 84, 87, 91, 93, 97.
Idiosoma hirsutum Main, 1952: 132, fig. 2B. Main 1957b: 440, fig. 15A–B. Main 1985: 54. Synonymised by Rix et al. 2017d: 593.

#### Type material (of *Idiops sigillatus*).

Holotype male. Swan River [Perth] (IBRA_SWA), Western Australia, Australia (OUM; not examined).

#### Type material (of *Idiosoma hirsutum*).

Holotype female. Victoria Park [Perth] (IBRA_SWA), Western Australia, Australia, 10 August 1929, L.W. Gibbs (WAM T2172; examined).

#### Other material examined.

**AUSTRALIA: *Western Australia***: 1 ♂, Alexander Heights (IBRA_SWA), 31°50'S, 115°52'E, 12 June 1994, H. Smithies (WAM T31063); 1 ♂, Applecross (IBRA_SWA), 32°01'S, 115°50'E, hand collected, 12 June 1956, J.A. Dobson (WAM T3920); 1 juvenile, same locality data, 16 May 1957, W. Porter (WAM T3941); 1 ♂, same data except 18 July 1968, P. Vredenbreat (WAM T18533); 1 ♂, same locality data, dead in swimming pool, 3 July 1994, T. Dunlop (WAM T31064); 1 ♂, Attadale (IBRA_SWA), 32°01'34"S, 115°48'02"E, hand collected, 20 May 1971, R. Reed (WAM T18534); 1 ♀, same locality data, 29 April 1989, S.W. Munsie (WAM T27138); 1 ♂, same locality data except 32°01'S, 115°48'E, June 1960, W. Lane (WAM T144870); 1 ♂, Australind (IBRA_SWA), 33°17'01"S, 115°42'42"E, hand collected, 2 June 1984, C. Thomas (WAM T18562); 1 ♂, same locality data except 33°16'S, 115°44'E, 1 July 1991, K.F. Longbottom (WAM T26112); 1 ♀, same locality data except 9 Laura Avenue, off Coast Road, N. of Bunbury (IBRA_SWA), 33°17'40"S, 115°42'39"E, 25 April 2011, submission to Bunbury DEC Office, via A. Longbottom (WAM T113274); 1 ♂, Avalon, 5 miles S. of Mandurah (IBRA_SWA), 32°33'S, 115°42'E, hand collected, 5 June 1976, L. Freeman (WAM T18569); 1 ♀, Balcatta (IBRA_SWA), 31°53'S, 115°49'E, 25 June 1985, N. Bowie (WAM T18463); 1 ♀, Balga (IBRA_SWA), 31°51'38"S, 115°50'14"E, 16 April 1971, T. Dawson (WAM T18464); 1 ♀, same data except 3 November 1983, J. Rasmussen (WAM T18465); 1 ♀, same locality data except 31°52'S, 115°50'E, 1 January 1997, S. James (WAM T38487); 1 ♀, same data (WAM T38488); 1 ♀, Bayswater (IBRA_SWA), 31°55'S, 115°54'E, 16 May 1938, J. Green (WAM T3073); 1 ♀, same locality data except 31°55'08"S, 115°54'53"E, 7 November 1966, Mrs Little (WAM T18466); 1 ♀, same data except 1 August 1974, F. Graham (WAM T18467); 1 ♂, Beeliar, 29 Waitch Loop (IBRA_SWA), 32°08'S, 115°47'E, 12 July 2001, V. Pinnell (WAM T44290); 1 ♀, Bentley (IBRA_SWA), 32°00'09"S, 115°55'21"E, 1 January 1975, V. Sullivan (WAM T26113); 1 ♀, Bibra Lake (IBRA_SWA), 32°06'S, 115°49'E, 17 December 1949, G. Wills-Johnson (WAM T3554); 1 ♂, Bold Park, site BP5 (IBRA_SWA), 31°57'14"S, 115°46'16"E, wet pitfall trap, 20 May–20 July 1993, M.S. Harvey, J.M. Waldock, A. Sampey (WAM T29776); 1 ♂, same data except 20 July–24 September 1993, J.M. Waldock (WAM T29777); 1 ♂, Booragoon (IBRA_SWA), 32°02'24"S, 115°49'46"E, hand collected, 1 October 1978, W.T. Heron (WAM T18563); 1 ♂, Boyanup, Christopher Way (IBRA_SWA), 33°29'S, 115°44'E, 1 July 1993, D. Anderson (WAM T29941); 1 ♂, same locality data except Williams Road, 33°29'12"S, 115°43'35"E, 16 May 1991, A. Harbeck (WAM T26115); 1 ♂, Brookdale, site BW3 (IBRA_SWA), 32°09'15"S, 115°57'55"E, wet pitfall trap, *Banksia attenuata*, *B. menziesii* woodland, 6 September 2006, J. Oats (WAM T84300); 1 ♂, same data except site MW1, 32°09'13"S, 115°57'45"E, *Melaleuca pressiana*, *M. rhaphiophylla* open woodland (WAM T84301); 1 ♂, same data except site MW3, 32°09'13"S, 115°57'43"E, 12 July 2006 (WAM T84302); 1 ♂, same data except 6 September 2006 (WAM T84303); 1 ♀, Brunswick Junction (IBRA_SWA), 33°15'S, 115°50'E, April 1960 (WAM T144869); 1 ♀, same data except 24 September 1999, A. Lorkiewicz (for school student) (WAM T45976); 1 ♂, Bunbury (IBRA_SWA), 33°20'29"S, 115°38'26"E, hand collected, 19 May 1980, T. Locke (WAM T18564); 1 juvenile, same data except 20 May 1975, Mr Martinson (WAM T26116); 1 ♂, same locality data except 4 Clifton Street, 33°19'S, 115°38'E, hand collected in garden, 26 June 1988, W. Wood (WAM T18588); 1 ♀, same locality data except Ashmere Heights, 33°20'S, 115°38'E, 29 August 1995, K.F. Longbottom (WAM T32531); 1 ♂, Canning Vale (IBRA_SWA), 32°08'S, 115°55'E, 8 June 1971, F.L. Sykes (WAM T18535); 1 ♀, same data except 8 January 1929, J. Eichner (WAM T2038); 1 ♀, Cannington (IBRA_SWA), 32°01'S, 115°57'E, 15 January 1971, E.E. Leach (WAM T18468); 1 ♂, Carine (IBRA_SWA), 31°51'12"S, 115°46'45"E, hand collected, 10 July 1988, R. Thomas (WAM T 18619); 1 ♂, same data except 9 July 1988, A. Bradbrook (WAM T18620); 1 ♂, same data (WAM T18621); 1 ♂, same data except 31°51'S, 115°47'E, hand collected inside garage, 15 August 1993, R. Thomas (WAM T28479); 1 juvenile, Carlisle (IBRA_SWA), 31°59'S, 115°55'E, 31 August 1951 (WAM T3639); 1 ♀, City Beach (IBRA_SWA), 31°56'08"S, 115°45'47"E, 19 April 1986, R. Eldred (WAM T18469); 1 ♂, Claremont (IBRA_SWA), 31°59'S, 115°47'E, hand collected, 26 June 1939, R.R. Everall (WAM T3180); 1 ♀, same locality data except 31°59'S, 115°47'E, 21 March 1965, R.A. Farmer (WAM T18470); 1 ♀, same locality data except 66 Watkins Road, 31°59'33"S, 115°47'27"E, 22 March 1968, M. Thomson (WAM T144757); 1 ♂, same locality data except Bishop Road Reserve, Victoria Ave, 32°00'S, 115°47'E, 8 June 1979, C. Graham (WAM T102066); 1 ♀, Clarkson, 40 Willoughby Retreat (IBRA_SWA), 31°42'S, 115°44'E, 21 May 2000, M. Kelly (WAM T41608); 1 juvenile, Como (IBRA_SWA), 32°00'S, 115°52'E, 27 July 1953, J.T. Hatton (WAM T3729); 1 ♀, same locality data except 31°59'59"S, 115°52'30"E, 18 July 1971, I. Daniel (WAM T18578); 1 ♀, same data except 18 June 1989, C. Skehan (WAM T21255); 1 ♂, same locality data except Mount Henry Hospital (IBRA_SWA), 32°01'S, 115°52'E, 29 May 1989, E. Finch (WAM T139482); 1 ♀, Cooberlup, North Lake Primary School (IBRA_SWA), 32°05'S, 115°49'E, 6 November 1975, P. O’Malley (WAM T26129); 1 ♂, Cottesloe (IBRA_SWA), 31°59'53"S, 115°45'34"E, hand collected at base of tree in undisturbed area of yard, 9 July 1988, I.N. McDonald (WAM T18622); 1 ♂, same locality data, 1 July 1986, R.P. McMillan (WAM T27140); 1 ♀, same data except 7 August 1988 (WAM T27139); 1 ♀, same data except 28 May 1977, B. Stynes (WAM T18471); 1 ♂, same data except dead on ground, 13 July 1993 (WAM T29778); 1 ♂, Crawley (IBRA_SWA), 31°59'S, 115°49'E, 15 May 2014, via M. Plant (WAM T132564^DNA_Voucher_ID^); 1 ♀, same locality data except Park Avenue, 8 May 1992 (WAM T144756); 1 ♀, same locality data except University of Western Australia, 31°59'S, 115°48'E, June 2002, J. Dunlop (WAM T144623); 1 ♂, Curtin University of Technology, Biology Field Trial Area, corner of Townsing and Manning Roads (IBRA_SWA), 32°00'44"S, 115°53'44"E, hand collected, July 2003, P. Mioduszewski, K.E.C. Brennan (WAM T55925^DNA_Voucher_127^); 1 ♂, Daglish, Troy Terrace (IBRA_SWA), 31°57'S, 115°49'E, 30 July 1989, R. Hughes (WAM T21789); 1 ♂, Dalkeith (IBRA_SWA), 31°59'47"S, 115°47'54"E, hand collected, 31 May 1962, C. Fyfe (WAM T18536); 1 ♂, same data except 17 July 1979, Mrs Hutchinson (WAM T29845); 1 ♀, same data except 13 November 1964, J.S. Flower (WAM T18472); 1 ♂, Dalyellup (IBRA_SWA), 33°24'S, 115°37'E, hand collected on driveway, 4 June 2002, K.F. Longbottom (WAM T46054^DNA_Voucher_128^); 1 ♂, Dardanup, Lot 63 Padbury Road, Padbury Fields (IBRA_SWA), 33°24'S, 115°45'E, hand collected, 4 May 1992, B. Brittain (WAM T27984); 1 ♂, Dawesville (IBRA_SWA), 32°39'S, 115°38'E, 26 May 2010, H.H. Adamson (WAM T126089); 1 ♀, Dianella (IBRA_SWA), 31°53'20"S, 115°52'22"E, 30 June 1983, J. Hanlon (WAM T18473); 1 ♀, same locality data except 31°53'S, 115°52'E, 14 December 2000, C. Broder (WAM T44179); 1 ♂, Doubleview, 130 Alice Street (IBRA_SWA), 31°53'S, 115°46'E, 10 September 1998, L. Ogilvie (WAM T45994); 1 ♂, Duncraig (IBRA_SWA), 31°50'S, 115°47'E, hand collected on brickwork surrounding barbeque, 9 August 1985, P. Jernakoff (WAM T18538); 1 ♂, same locality data except Lilburne Road, half-way down road, 31°50'S, 115°46'E, 7 June 2005, A. Wilson (WAM T139480); 1 ♀, same locality data except 31°50'02"S, 115°46'34"E, 8 October 1988, C.F. Chester (WAM T27141); 1 ♀, same data except 29 April 1981, R.J. Catto (WAM T18474); 1 ♀, East Perth, 15 Joel Terrace (IBRA_SWA), 31°58'S, 115°52'E, 13 April 1928, J. Morley (WAM T1881); 1 ♀, Eaton (IBRA_SWA), 33°18'55"S, 115°42'39"E, 5 May 1980, V. Skinner (WAM T18475); 1 ♀, Elgin (IBRA_SWA), 33°30'49"S, 115°37'49"E, 3 May 1976, R. Fester (WAM T26118); 1 ♀, Falcon (IBRA_SWA), 32°35'S, 115°40'E, 19 August 2004, via WAM Discovery Centre (WAM T58494); 1 ♂, Floreat Park (IBRA_SWA), 31°56'S, 115°47'E, 24 June 1953, W.H. Butler (WAM T139478); 1 ♀, same data except 27 July 1953, H. Kent (WAM T3728); 1 ♀, same locality data except 3 Kinross Crescent, 19 September 1958, A. Stable (WAM T18476); 1 ♂, Forrestdale (IBRA_SWA), 32°08'57"S, 115°56'22"E, hand collected, 17 May 1984, G. Murphy (WAM T18539); 1 ♀, same locality data, 15 May 1983, G. Murphey (WAM T18477); 1 ♂, same locality data except Stirling Road Golf Club, Forrest Road, 15 May 1977, J. Davey (WAM T18565); 1 ♀, same locality data except 32°08'S, 115°56'E, 29 November 1933, U. Skeet (WAM T2647); 1 ♀, Forrestfield (IBRA_SWA), 31°59'S, 115°58'E, 9 May 1968, M.E. Ryding (WAM T18478); 1 ♂, Fremantle (IBRA_SWA), 32°03'26"S, 115°44'40"E, 24 May 1979, R. Treloar (WAM T26119); 1 ♂, Gelorup (IBRA_SWA), 33°23'S, 115°39'E, 4 July 1994, P. van Dijk (WAM T31152); 1 ♀, same locality data except 33°23'12"S, 115°38'43"E, 6 April 1987, S. Platts (WAM T18479); 1 ♂, same locality data except Lot 101, Gelorup Rise, 1 May 1991, K.F. Longbottom (WAM T26121); 1 ♂, same data except 1 July 1991 (WAM T26122); 1 ♂, same data except 20 July 1991 (WAM T26124); 1 ♂, same data except in swimming pool, 1 July 1991 (WAM T26120); 1 ♂, same data except 29 May 1991 (WAM T26123); 1 ♀, Gingin, 2 Robinson Street (IBRA_SWA), 31°20'40"S, 115°54'20"E, ex. burrow in backyard, 27 July 2011, M. Wolters (WAM T116016); 1 ♀, same locality data except Fraser Street, 31°21'S, 115°54'E, 29 September 1994, M. Vallentine (WAM T31790); 1 ♂, Girrawheen (IBRA_SWA), 31°50'32"S, 115°50'18"E, hand collected, 14 June 1977, D. Bell (WAM T18566); 1 ♂, same data except 17 June 1987, J. Napier-Winch (WAM T18567); 1 ♂, same locality data except building site, 31°50'S, 115°50'E, hand collected under brick, 18 July 1972, R.J. Pushman (WAM T18537); 1 ♂, Gnangara East (IBRA_SWA), 31°46'27"S, 115°51'33"E, hand collected, 10 June 1969, A.M. Douglas (WAM T18540); 1 ♀, Gosnells (IBRA_SWA), 32°04'23"S, 115°59'36"E, 13 April 1986, J. Hopkins (WAM T18480); 1 ♀, Greenwood (IBRA_SWA), 31°49'42"S, 115°48'02"E, 4 July 1976, G.L. Bodycoat (WAM T18481); 1 ♀, same data except 12 May 1985, P.A. Miller (WAM T18482); 1 ♀, Inglewood (IBRA_SWA), 31°55'S, 115°53'E, November 1952, W.H. Butler (WAM T144855); 1 ♀, Jandakot (IBRA_SWA), 32°06'13"S, 115°53'13"E, 15 May 1988, M. Patterson (WAM T18483); 1 ♀, Joondanna (IBRA_SWA), 31°54'40"S, 115°50'04"E, 24 October 1977, J.G. Murry (WAM T18484); 1 ♀, Karnup, 301 Amarillo Drive (IBRA_SWA), 32°24'46"S, 115°48'23"E, on mat on porch, 5 May 2013, M. Pollock (WAM T129191^DNA_Voucher_134^); 1 ♂, Karrakatta, Irwin Barracks (IBRA_SWA), 31°57'58"S, 115°47'43"E, in compound before storm, 29 July 2011, S. van de Wilde (WAM T115195); 1 ♂, Karrinyup (IBRA_SWA), 31°52'41"S, 115°46'24"E, hand collected, 9 August 1972, Mrs Granland (WAM T18542); 1 ♀, same data except 11 June 1971, A. Neille (WAM T18485); 1 ♀, same locality data except 31°52'S, 115°46'E, M. Denby (WAM T144872); 1 ♀, Kings Park, Perth (IBRA_SWA), 31°57'S, 115°50'E, 15 September 1953, B.Y. Main (WAM T144862); 1 ♀, same locality data except 31°57'10"S, 115°49'55"E, 22 May 1973, R. James (WAM T18486); 1 ♂, same locality data except site 1/2, pitfall trap, 17 July 1992, J. Dell (WAM T27137); 1 ♂, same data except site 2/6, 19 July 1992 (WAM T27136); 1 ♂, same data except site 2/7 (WAM T27135); 1 ♂, Kingsley (IBRA_SWA), 32°08'20"S, 116°00'31"E, 20 May 1994, S. Carey (WAM T31153); 1 ♂, same locality data except Goollelal Drive, 31°48'S, 115°47'E, 18 September 2008, M. Fraser (WAM T93320); 1 ♀, Kwinana (IBRA_SWA), 32°14'S, 115°46'E, 6 November 1968, H. Burton (WAM T18487); 1 ♀, Ledge Point (IBRA_SWA), 31°06'S, 115°22'E, 22 November 1967, R. Shaw (WAM T18488); 1 ♀, Leschenault, 41 Roberts Road, NE. of Bunbury (IBRA_SWA), 33°14'59"S, 115°43'08"E, hand collected after rain, on suburban patio, surrounded by Jarrah-*Banksia* forest, 29 April 2012, T. Edwards (WAM T123102^DNA_Voucher_157^); 1 ♂, Lynwood (IBRA_SWA), 32°02'51"S, 115°55'01"E, hand collected, 6 May 1973, R.J. Blair (WAM T18568); 1 ♀, same locality data except 32°03'S, 115°55'E, 7 July 1975, M. Sillis (WAM T10215); 1 ♂, Maida Vale, Bruce Road (IBRA_SWA), 31°57'00"S, 115°59'45"E, hand collected on front doorstep, 25 June 1989, S. Gilligan (WAM T20602); 1 ♂, Malaga (IBRA_SWA), 31°52'S, 115°54'E, 4 August 1994, V. Hopkins (WAM T31154); 1 juvenile, Mandurah (IBRA_SWA), 32°32'S, 115°43'E, hand collected, 11 March 1969, R. Prestage (WAM T18489); 1 juvenile, same data (WAM T18490); 1 juvenile, same data (WAM T18491); 1 juvenile, same data (WAM T18492); 1 juvenile, same data (WAM T18493); 1 juvenile, same data (WAM T18494); 1 juvenile, same data (WAM T18495); 1 juvenile, same data (WAM T18496); 1 juvenile, same data (WAM T18497); 1 juvenile, same data (WAM T18498); 1 juvenile, same data (WAM T18499); 1 juvenile, same data (WAM T18500); 1 juvenile, same data (WAM T18501); 1 juvenile, same data (WAM T18502); 1 juvenile, same data (WAM T18503); 1 juvenile, same data (WAM T18504); 1 juvenile, same data (WAM T18505); 1 juvenile, same data (WAM T18506); 1 juvenile, same data (WAM T18507); 1 juvenile, same data (WAM T18508); 1 juvenile, same data (WAM T18509); 1 juvenile, same data (WAM T18510); 1 ♂, same locality data except 32°31'S, 115°43'E, June 1996, C. Duffus (WAM T46829); 1 ♂, same locality data except Halls Head, 32°31'40"S, 115°42'03"E, hand collected, 19 June 1987, P. Ashburn (WAM T18541); 1 ♀, Melros, S. of Mandurah (IBRA_SWA), 32°38'S, 115°37'E, 1 October 1989, R.A. Sewell (WAM T27143); 1 ♂, Melville (IBRA_SWA), 32°02'30"S, 115°47'59"E, hand collected, 4 June 1988, B.M. Grierson (WAM T18589); 1 ♂, same locality data, 14 July 1992, T. Portelli (WAM T26126); 1 ♀, same data except 10 March 1961, A.J. Forrester (WAM T18511); 1 ♀, same data except 19 June 1978, R.G. Mill (WAM T18513); 1 ♀, same data except Melville Heights, 16 May 1967, L. Powell (WAM T18512); 1 ♂, Miami, via Mandurah (IBRA_SWA), 32°35'S, 115°39'E, hand collected, 13 August 1987, T.G. Spalding (WAM T18543); 1 ♂, Morley (IBRA_SWA), 31°53'18"S, 115°54'20"E, hand collected, 16 July 1971, C. Johnston (WAM T18545); 1 ♂, same data except 5 July 1972, C.J. Glass (WAM T18546); 1 ♂, same locality data except 42 Shadwell Way, 31°53'S, 115°54'E, in swimming pool, September 2008–January 2009, J. Mahoney (WAM T95764); 1 ♂, Morley Park (IBRA_SWA), 31°53'18"S, 115°54'20"E, hand collected, 2 May 1965, K.E. Panten (WAM T18544); 1 ♂, Mosman Park (IBRA_SWA), 32°01'01"S, 115°45'40"E, hand collected, 28 June 1971, J. Luce (WAM T18547); 1 ♂, same data except 16 June 1980, A. Symington (WAM T29847); 1 ♂, Mount Claremont, site MC1 (IBRA_SWA), 31°57'40"S, 115°46'00"E, wet pitfall trap, 4 May–6 July 1995, J.M. Waldock, M.S. Harvey (WAM T34261); 1 ♀, same locality data except 31°57'47"S, 115°46'58"E, 24 May 1991, L. Herbison (WAM T26127); 1 ♀, same data except 14 November 1978, J. Brown (WAM T29844); 1 ♂, Mount Hawthorn, Lynton Street (IBRA_SWA), 31°55'S, 115°50'E, 9 June 1956, J.H. Calaby (WAM T139489); 1 ♀, Mount Lawley (IBRA_SWA), 31°56'S, 115°53'E, 12 August 1929, S. Easter (WAM T2173); 1 ♂, Mount Pleasant (IBRA_SWA), 32°02'S, 115°51'E, 14 August 1994, J. DeLauey (WAM T31155); 1 ♀, same locality data except 12 March 1990 (WAM T20760); 1 ♂, same locality data except 32°02'42"S, 115°50'48"E, 14 June 1966, I. Pelliele (WAM T18548); 1 ♂, same data except 1 January 1975, S.J. Edwards (WAM T18570); 1 ♀, same data except 21 May 1964, A.W. Jones (WAM T18515); 1 ♀, same data except 8 September 1972, T. Bailey (WAM T18516); 1 ♀, same locality data, 23 September 1960 (WAM T18514); 1 ♀, Mount Yokine (IBRA_SWA), 31°54'S, 115°51'E, 23 November 1968, R.G. Howell (WAM T18517); 1 ♀, about 7 miles N. of Muchea on Gingin Road (IBRA_SWA), 31°29'S, 115°59'E, 3 January 1953, B.Y. Main (WAM T144856); 1 ♂, Mundijong (IBRA_SWA), 32°18'S, 115°59'E, 3 May 2000, H. Hunter (WAM T41569); 1 ♂, Murdoch (IBRA_SWA), 32°04'18"S, 115°49'26"E, 13 September 1989, D. Mead-Hunter (WAM T21808); 1 ♂, same data except 19 July 1989 (WAM T21809); 1 ♂, Murdoch University (IBRA_SWA), 32°04'18"S, 115°49'26"E, hand collected, 1 June 1986, D. Mead-Hunter (WAM T18549); 1 ♂, Myaree (IBRA_SWA), 32°02'33"S, 115°48'49"E, hand collected, 29 April 1973, T. Hall (WAM T18550); 1 ♂, same data except 8 July 1984, A. Gurrier-Jones (WAM T18551); 1 ♂, Naval Base [Garden Island] (IBRA_SWA), 32°11'51"S, 115°46'47"E, hand collected, B. Rundle (WAM T18552); 1 ♂, same locality data except 32°13'S, 115°41'E, 1954, V.N. Serventy (WAM T139479); 1 ♂, Nedlands (IBRA_SWA), 31°58'S, 115°48'E, hand collected, 18 June 1961, Mr Simon (WAM T18561); 1 ♂, same data except 11 July 1978, S.C. Smith (WAM T18571); 1 ♂, same locality data, 20 May 1989, R. Albert (WAM T27144); 1 ♀, same data except 8 July 1938, J. Marriott (WAM T3085); 1 ♀, same data except 23 September 1963, E.W. Felton (WAM T18518); 1 ♀, same data (WAM T18519); 1 ♀, same locality data except 50 Langham Street, 25 October 1988, S. Edmonds (WAM T21148); 1 ♂, same locality data except Kinninmont [sic “Kinnamont”] Avenue,13 May 1977 (WAM T139485); 1 ♀, same data except December 1960 (WAM T144871); 1 ♂, same locality data except Monash Avenue, 23 May 1988, E. Armstrong (WAM T139493); 1 ♂, same locality data except Napier Street, June 1970, J. Henegan (WAM T139483); 1 ♂, same locality data except Zoology Department, University [of Western Australia] Grounds, 31°59'S, 115°49'E, on ground, June 1957, B.Y. Main (WAM T139492); 1 ♀, same locality data except Zoology Department Car Park, University of Western Australia, 31°58'59"S, 115°48'21"E, 22 June 1984, E. Cannella (WAM T26131); 1 ♀, same locality data except Nedland Bowling Green, 25 May 1984, W. Gibb (WAM T144873); 1 ♂, Nollamarra (IBRA_SWA), 31°53'00"S, 115°50'39"E, hand collected, 19 June 1963, R. Cross (WAM T18553); 1 ♀, same locality data, 10 January 1982, A. Paxman (WAM T26128); 1 ♀, North Fremantle (IBRA_SWA), 32°02'S, 115°45'E, 24 March 1937, R. Paton (WAM T2969); 1 ♂, North Yunderup (IBRA_SWA), 32°35'S, 115°46'E, hand collected, 27 July 1988, M. Ryan (WAM T18623); 1 ♀, Orelia (IBRA_SWA), 32°14'12"S, 115°49'18"E, 4 March 1973, D. Wood (WAM T18520); 1 ♀, same data except 5 January 1977, A.J. Hood (WAM T22555); 1 ♀, Osborne Park (IBRA_SWA), 31°53'S, 115°48'E, 24 April 1957, C.H. Locke (WAM T3940); 1 ♂, Padbury (IBRA_SWA), 31°48'31"S, 115°46'05"E, hand collected, 5 June 1981, R. Van Der Burg (WAM T18572); 1 ♂, Palmyra (IBRA_SWA), 32°02'S, 115°47'E, July 1982, B. Pusey (WAM T139484); 1 ♂, Parmelia (IBRA_SWA), 32°14'35"S, 115°50'00"E, in swimming pool, 16 June 1992, A.E. de Jong (WAM T28543); 1 ♀, same locality data except 32°15'S, 115°50'E, 12 July 1995, A.E. de Jong (WAM T 34262); 1 ♂, same locality data except 59 Sicklemore Road, 32°15'S, 115°49'E, 6 June 1999, A.E. de Jong (WAM T41864); 1 ♂, Perth (IBRA_SWA), 31°57'S, 115°51'E, 25 May 1971, B. Roberts (WAM T18580); 1 ♀, same data except 1 September 1979, P. Owens (WAM T29848); 1 ♀, 4 miles SE. of Perth (IBRA_SWA), 31°59'S, 115°54'E, 11 May 1959, N. Douglas (WAM T18579); 1 ♂, Perth Airport, site PA8 (IBRA_SWA), 31°58'36"S, 115°58'28"E, wet pitfall trap, 10 May–24 June 1993, M.S. Harvey, J.M. Waldock (WAM T30024); 1 ♂, Pinjarra (IBRA_SWA), 32°37'52"S, 115°52'11"E, hand collected, 8 June 1971, T. Venn (WAM T18555); 1 ♀, same locality data except 32°37'S, 115°52'E, 20 May 1955 (WAM T3798); 1 ♂, Riverton, 224 Corinthian Road East (IBRA_SWA), 32°02'00"S, 115°53'50"E, D. Crook (WAM T139491); 1 ♂, Rockingham (IBRA_SWA), 32°17'S, 115°43'E, 30 May 1995, J. Koning (WAM T32556); 1 ♀, same data except 2 April 1986, P. Stuthridge (WAM T26981); 1 ♂, same locality data except 32°17'S, 115°45'E, 25 May 1980, C. Barth (WAM T139490); 1 ♀, railway crossing E. of Rockingham (IBRA_SWA), 32°17'S, 115°47'E, 3 July 1955, B.Y. Main (WAM T144865); 1 juvenile, same data (WAM T144866); 1 ♂, Rottnest Island (IBRA_SWA), 32°00'S, 115°31'E, under bush, 1 April 1982, S. Bunn (WAM T139486); 1 ♂, same locality data, 13 June 1982, T. Elder, D. Edward (WAM T139488); 1 ♂, same locality data except knoll on airfield, 32°00'S, 115°32'E, 5 July 1959, G. Storr (WAM T139481); 1 ♀, same locality data except west end of island, 32°01'S, 115°29'E, 20 November 1956, J.A. Watson (WAM T144868); 1 ♀, Salter Point (IBRA_SWA), 32°01'37"S, 115°52'14"E, 3 May 1973, L. Doran (WAM T18581); 1 ♀, Scarborough (IBRA_SWA), 31°53'55"S, 115°45'57"E, 11 February 1960, C. Burton (WAM T18521); 1 ♀, same data except 20 October 1964, M.G. Campbell (WAM T18522); 1 ♀, Shelley (IBRA_SWA), 32°01'47"S, 115°53'16"E, 29 April 1976, G. Reddall (WAM T26130); 1 ♂, Shenton Park (IBRA_SWA), 31°57'37"S, 115°48'00"E, hand collected, 22 June 1973, J. Francis (WAM T18556); 1 juvenile, Shenton Park Bushland, off Lemnos Street, Perth (IBRA_SWA), 31°57'39"S, 115°48'01"E, hand collected, coastal plain woodland, 26 April 2014, M.G. Rix (WAM T132756^DNA_Voucher_NCB_016^); 1 ♂, Shenton Park Rehabilitation Hospital, Selby Street (IBRA_SWA), 31°58'S, 115°48'E, under rocks, 22 June 1997, G. Pickles (WAM T46830); 1 ♀, Sorrento (IBRA_SWA), 31°49'05"S, 115°44'33"E, 2 May 1976 (WAM T18523); 1 ♀, same data except 1 July 1977, D. Maxwell (WAM T18524); 1 ♂, same locality data except 101 Cliff Street, 31°50'05"S, 115°45'10"E, 1998, W. O’Brien (WAM T139487); 1 ♂, South Como (IBRA_SWA), 31°59'59"S, 115°52'30"E, hand collected, 27 June 1961, W. Liddle (WAM T18576); 1 ♀, South Perth (IBRA_SWA), 31°59'S, 115°52'E, 12 October 1914, W.H. Mathews (WAM T358); 1 ♀, Spearwood (IBRA_SWA), 32°06'02"S, 115°47'46"E, 10 February 2009, N. Mitchell (WAM T96800); 1 ♀, Swanbourne (IBRA_SWA), 31°59'S, 115°46'E, 26 November 1936, M. Bemrose (WAM T2960); 1 ♂, same locality data except 31°58'12"S, 115°45'57"E, hand collected, 14 July 1985, R. Atkinson (WAM T18557); 1 ♂, Tuart Hill (IBRA_SWA), 31°53'56"S, 115°49'57"E, hand collected, 20 June 1960, J. Rockley-Warne (WAM T18558); 1 ♀, same locality data, 12 April 1965, M. Ward (WAM T18525); 1 ♀, same data except 1 October 1970, R.J. Warne (WAM T18526); 1 ♀, same data except 18 March 1980, J. Siddons (WAM T18527); 1 juvenile, same locality data except site TH1, 31°52'49"S, 115°51'30"E, wet pitfall trap, 10 May–20 July 1993, M.S. Harvey, J.M. Waldock (WAM T30023); 1 ♀, Victoria Park (IBRA_SWA), 31°58'S, 115°54'E, 11 August 1930, A. Hand (WAM T2257); 1 ♀, Wangara (IBRA_SWA), 31°47'31"S, 115°49'39"E, 29 September 1986, J.M. Lund (WAM T18528); 1 ♂, same locality data except Ausfutons factory, 11 Niche Parade, 31°47'35"S, 115°51'11"E, inside factory building, 24 July 2015, M. Wilton (WAM T136936); 1 ♂, ex. Wangara Bush Block (IBRA_SWA), [no other data] (WAM T139477); 1 ♂, Wanneroo (IBRA_SWA), 31°44'54"S, 115°48'06"E, hand collected, 31 May 1971, M. Duffy (WAM T18559); 1 ♂, same data except 8 August 1983, R. D’Olimpia (WAM T18573); 1 ♂, same data except 17 July 1990, C. Guy (WAM T21204); 1 ♂, Warnbro (IBRA_SWA), 32°20'S, 115°45'E, 1 July 1976 (WAM T26132); 1 ♂, Warwick Road Reserve, site WR2 (IBRA_SWA), 31°50'33"S, 115°49'00"E, 30 June–5 July 1996, J.M. Waldock, P. West, M. Palacios, S. Chatterton (WAM T125366); 1 ♀, Wattle Grove (IBRA_SWA), 31°59'39"S, 115°58'51"E, 4 October 1971, G. Clinckers (WAM T18529); 1 ♂, Wattleup, 66 Wattleup Road (IBRA_SWA), 32°10'36"S, 115°48'00"E, G. Thornton (WAM T139476); 1 ♂, Wembley Downs (IBRA_SWA), 31°55'08"S, 115°46'38"E, 21 May 1968, A.B. Waters (WAM T18560); 1 ♀, same data except 28 May 1973, K. Jerrat (WAM T18530); 1 ♀, same data except 21 November 1983, J.W. Newton (WAM T18531); 1 ♀, Wembley Park [now Wembley], 19 Harborne Street (IBRA_SWA), 31°56'S, 115°49'E, 26 January 1950, W. Boyd (WAM T3555); 1 ♂, Westminster Primary School (IBRA_SWA), 31°52'S, 115°50'E, hand collected, 6 July 1979, Mrs Ritchie (WAM T18574); 1 ♂, Yanchep Park (IBRA_SWA), 31°33'S, 115°41'E, hand collected, 1 December 1981–1 January 1982, A. Smith (WAM T 18575); 1 ♂, Yarloop (IBRA_SWA), 32°58'S, 115°54'E, hand collected, 1 June 1959, H. Riegurt (WAM T18577).

#### Diagnosis.

*Idiosoma sigillatum* is one of nine south-western Australian species in the *intermedium*- and *sigillatum*-clades which does not belong to the distinctive ‘sigillate complex’ (Fig. 25); these nine species can be distinguished from those ‘sigillate complex’ taxa (i.e., *I. arenaceum*, *I. clypeatum*, *I. dandaragan*, *I. kopejtkaorum*, *I. kwongan*, *I. nigrum* and *I. schoknechtorum*) by the absence of well-defined lateral sclerotic strips on the male abdomen (e.g., Figs 151, 212, 234), and by the significantly less sclerotised morphology of the female abdomen (which may be strongly corrugate but never leathery and ‘shield-like’) (e.g., Figs 4, 7, 8, 159, 220, 242). Males of *I. sigillatum* can be further distinguished from those of *I. gutharuka* and *I. incomptum* by the presence of enlarged (i.e., clearly visible) SP4 sclerites (Fig. 357; cf. Figs 186, 199); from *I. jarrah* and *I. mcclementsorum* by the colour of the legs, which do not have strongly contrasting bright yellow or orange-yellow femora (Fig. 358; cf. Figs 235, 292); from *I. formosum*, *I. intermedium* and *I. mcnamarai* by the presence of well-defined dorso-lateral abdominal corrugations and striations (Figs 352, 357, Key pane 9.1; cf. Figs 146, 151, 207, 212, 308, 313, Key pane 9.2); and from *I. gardneri* by the smaller, more widely spaced SP3 sclerites and the smaller SP4 sclerites (Fig. 357, Key pane 10.1; cf. Fig. 173, Key pane 10.2).

Females can be distinguished from those of *I. formosum*, *I. intermedium*, *I. jarrah* and *I. mcnamarai* by the presence of reinforced, sclerotised ridges on the abdomen, these separated by longitudinal rows of less sclerotised cuticle (Figs 365, 368; cf. Figs 159, 162, 220, 223, 242, 245, 321, 324); and from *I. mcclementsorum* by the smaller size of the SP3 sclerites (Fig. 368; cf. Fig. 302) [NB. females of *I. gardneri*, *I. gutharuka* and *I. incomptum* are unknown].

This species can also be distinguished from *I. corrugatum* (from the Eyre Peninsula of South Australia) by the shape of the prolateral clasping spurs on the male tibia I, which are oriented longitudinally (Fig. 359; cf. Fig. 109), and by the shape of the female eye group, which is broadly trapezoidal (Fig. 367; cf. Fig. 117).

#### Description (male WAM T139480).

**Figures 351–360.**
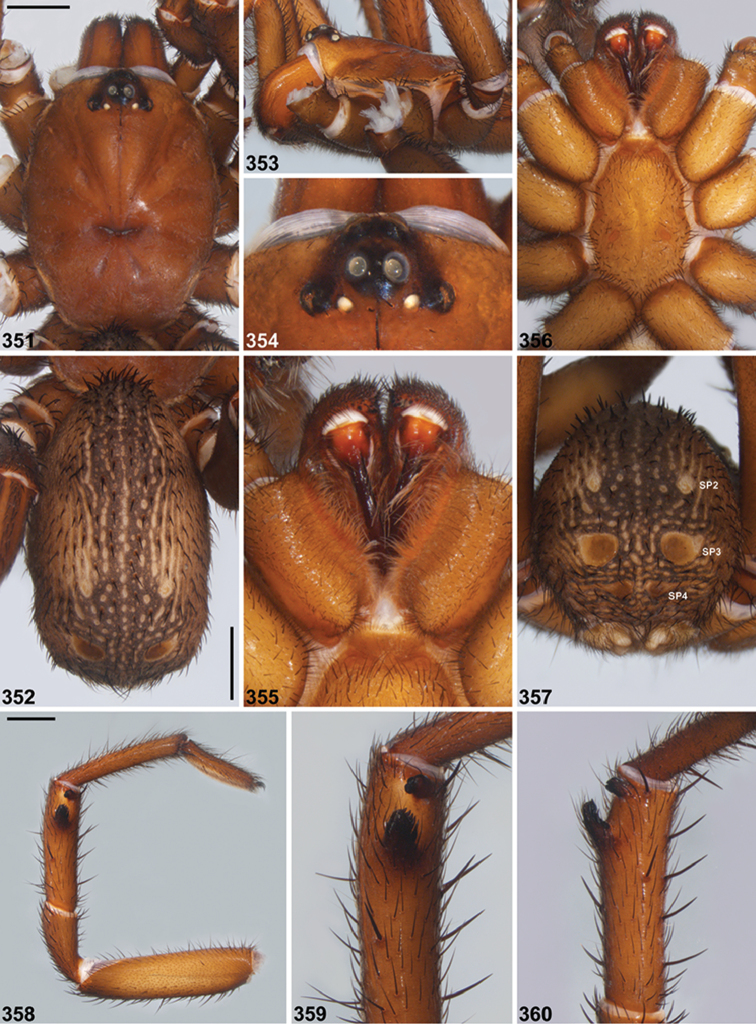
*Idiosoma sigillatum* (O.P.-Cambridge, 1870), male (WAM T139480) from Duncraig, Perth (Western Australia; SWA), somatic morphology. **351–352** Carapace and abdomen, dorsal view **353** Carapace, lateral view **354** Eyes, dorsal view **355** Mouthparts, ventral view **356** Sternum, ventral view **357** Abdomen, posterior view **358** Leg I, prolateral view **359** Leg I tibia, clasping spurs, prolateral view **360** Leg I tibia, proventral view. Scale bars 2.0. SP2–4 = sigilla pairs 2–4.

**Figures 361–363.**
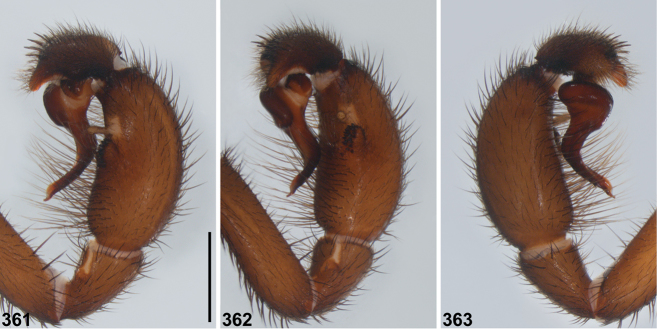
*Idiosoma sigillatum* (O.P.-Cambridge, 1870), male (WAM T139480) from Duncraig, Perth (Western Australia; SWA), pedipalp. **361** Retrolateral view **362** Retroventral view **363** Prolateral view. Scale bar 2.0.

Total length 18.8. Carapace 8.5 long, 6.3 wide. Abdomen 8.5 long, 5.0 wide. Carapace (Fig. 351) dark tan, with darker ocular region; lateral margins with uniformly spaced fringe of porrect black setae; fovea procurved. Eye group (Fig. 354) trapezoidal (anterior eye row strongly procurved), 0.7 × as long as wide, PLE–PLE/ALE–ALE ratio 2.4; ALE almost contiguous; AME separated by less than their own diameter; PME separated by 3.5 × their own diameter; PME and PLE separated by slightly more than diameter of PME, PME positioned in line with level of PLE. Maxillae and labium without cuspules. Abdomen (Figs 352, 357) oval, dark grey-brown in dorsal view with paler tan striations, dorso-lateral corrugations, and scattered dorsal sclerotic spots. Dorsal surface of abdomen (Fig. 352) more heavily setose anteriorly, with assortment of stiff, porrect black setae, each with slightly raised, dark brown sclerotic base. Posterior abdomen moderately sigillate (Figs 352, 357); SP2 sclerites comma-shaped spots; SP3 sclerites circular with irregular margins, each with unsclerotised triangular ‘corner’ laterally; SP4 sclerites oval, each surrounded by chevron-like pad of unsclerotised cuticle laterally; SP5 obscured. Legs (Figs 358–350) variable shades of dark tan, with light scopulae on tarsi I–II; distal tibia I with pair of large prolateral clasping spurs oriented longitudinally. Leg I: femur 7.4; patella 3.8; tibia 5.3; metatarsus 5.8; tarsus 3.4; total 25.6. Leg I femur–tarsus/carapace length ratio 3.0. Pedipalpal tibia (Figs 361–363) 2.2 × longer than wide; RTA burr-like, with conical basal protuberance and field of retroventral spinules; digital process porrect, unmodified. Cymbium (Figs 361–363) setose, with field of spinules disto-dorsally. Embolus (Figs 361–363) broadly twisted and sharply tapering distally, with prominent longitudinal flange and triangular (sub-distal) embolic apophysis.

#### Description (female WAM T129191).

**Figures 364–372.**
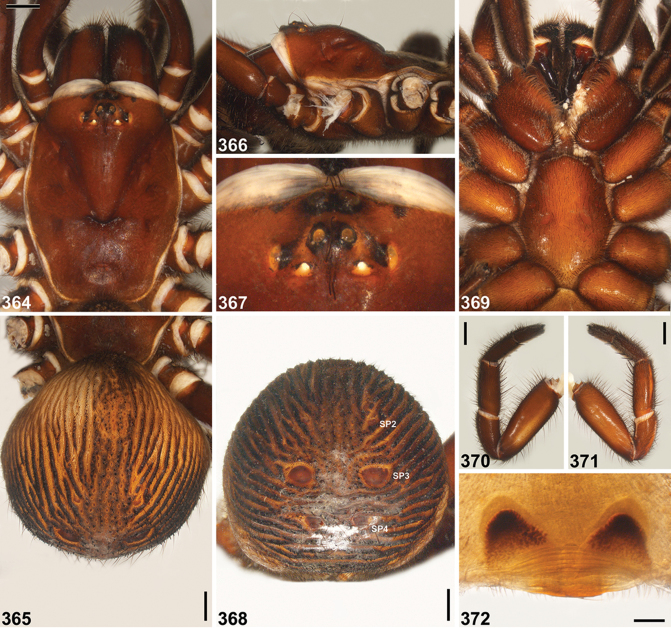
*Idiosoma sigillatum* (O.P.-Cambridge, 1870), female (WAM T129191) from Karnup, Perth (Western Australia; SWA). **364–365** Carapace and abdomen, dorsal view **366** Carapace, lateral view **367** Eyes, dorsal view **368** Abdomen, posterior view **369** Sternum, ventral view **370** Leg I, prolateral view **371** Leg I, retrolateral view **372** Spermathecae, dorsal view. Scale bars 2.0 (**364–365, 370–371**), 0.5 (**372**). SP2–4 = sigilla pairs 2–4.

Total length 30.7. Carapace 12.6 long, 9.3 wide. Abdomen 14.3 long, 14.5 wide. Carapace (Fig. 364) dark chocolate-brown, with darker ocular region; fovea procurved. Eye group (Fig. 367) trapezoidal (anterior eye row strongly procurved), 0.6 × as long as wide, PLE–PLE/ALE–ALE ratio 2.5; ALE almost contiguous; AME separated by approximately their own diameter; PME separated by 5.0 × their own diameter; PME and PLE separated by more than diameter of PME, PME positioned in line with level of PLE. Maxillae with field of cuspules confined to inner corner (Fig. 369); labium without cuspules. Abdomen (Figs 365, 368) truncate, tan, with reinforced dark brown-black corrugate ridges separated by longitudinal rows of less sclerotised cuticle, each ridge bearing row of modified stout setae. Posterior face of abdomen (Fig. 368) with rudimentary ‘shield-like’ morphology; SP3 sclerites circular with irregular margins, each surrounded by pad of unsclerotised cuticle; SP4 sclerites oval, each surrounded by chevron-like pad of unsclerotised cuticle laterally; SP5 sclerites obscured. Legs (Figs 370–371) variable shades of dark brown; scopulae present on tarsi and metatarsi I–II; tibia I with two stout pro-distal macrosetae and row of five longer retroventral macrosetae; metatarsus I with 10 stout macrosetae; tarsus I with distal cluster of short macrosetae. Leg I: femur 7.9; patella 4.9; tibia 4.5; metatarsus 3.9; tarsus 2.5; total 23.6. Leg I femur–tarsus/carapace length ratio 1.9. Pedipalp dark brown, spinose on tibia and tarsus, with thick tarsal scopula. Genitalia (Fig. 372) with pair of short, subtriangular spermathecae, each bearing dense field of glandular vesicles distally, and more sparsely distributed glandular field sub-distally.

#### Distribution and remarks.

**Figure 373.**
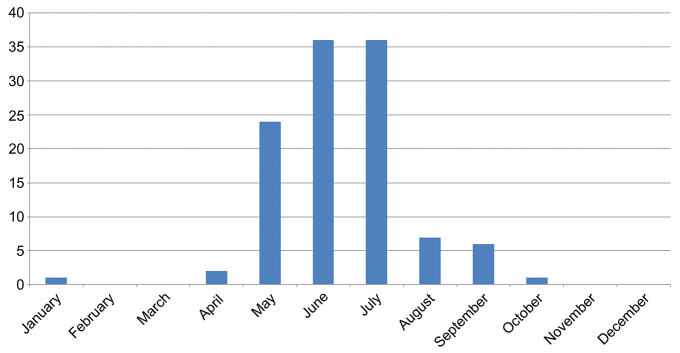
Graph summarising phenology data for 113 male specimens of *Idiosoma sigillatum* for which an accurate collection month could be ascertained. The y-axis denotes the number of specimens collected per month. Note the peak of activity in the coolest, wettest months of May–July.

**Figure 374.**
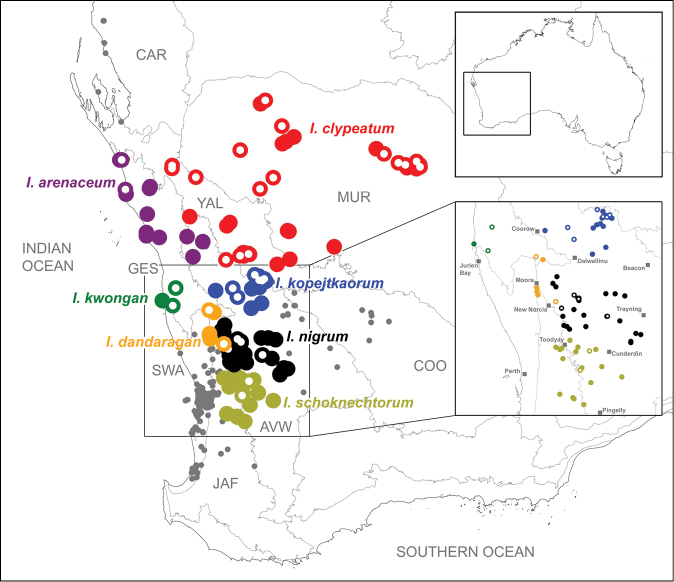
Map showing collection records of *Idiosoma* species in the ‘sigillate complex’ from south-western Australia; base map shows collection records for all other species in the *nigrum*-group as smaller grey dots. Lower inset shows detailed collection records for *I. nigrum* and other southern taxa, along with select metropolitan centres. Specimens sequenced for the molecular analyses are denoted by open circles, and relevant IBRA 7.0 bioregions are as follows: AVW, Avon Wheatbelt; CAR, Carnarvon; COO, Coolgardie; GES, Geraldton Sandplains; JAF, Jarrah Forest; MUR, Murchison; SWA, Swan Coastal Plain; YAL, Yalgoo.

**Figure 375.**
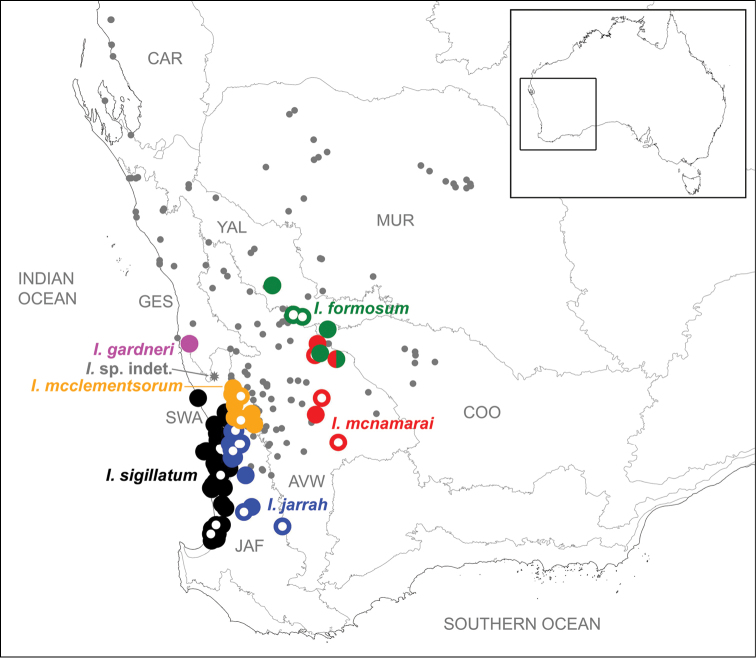
Map showing collection records of non-‘sigillate complex’ *Idiosoma* species in the *sigillatum*-clade from south-western Australia; base map shows collection records for all other species in the *nigrum*-group as smaller grey dots. Specimens sequenced for the molecular analyses are denoted by open circles, and relevant IBRA 7.0 bioregions are as follows: AVW, Avon Wheatbelt; CAR, Carnarvon; COO, Coolgardie; GES, Geraldton Sandplains; JAF, Jarrah Forest; MUR, Murchison; SWA, Swan Coastal Plain; YAL, Yalgoo.

**Figure 376.**
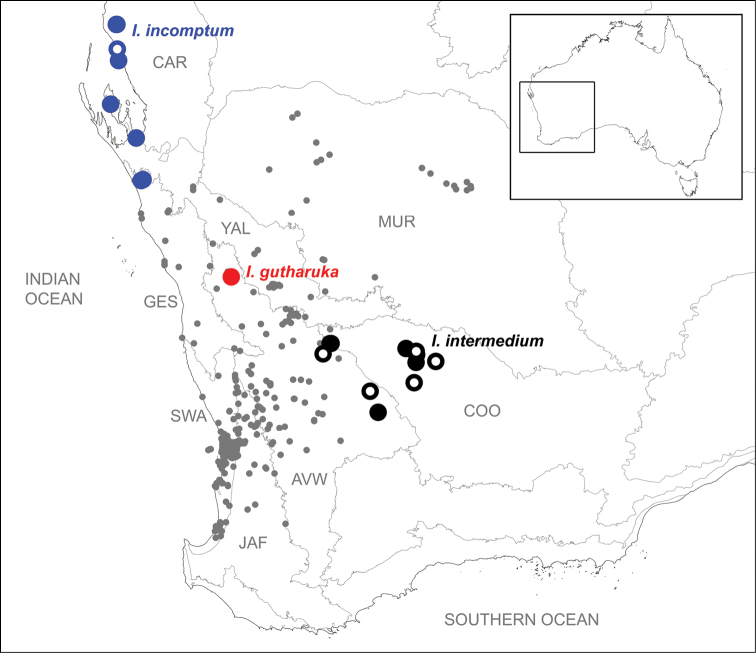
Map showing collection records of *Idiosoma* species in the *intermedium*-clade from south-western Australia; base map shows collection records for all other species in the *nigrum*-group as smaller grey dots. Specimens sequenced for the molecular analyses are denoted by open circles, and relevant IBRA 7.0 bioregions are as follows: AVW, Avon Wheatbelt; CAR, Carnarvon; COO, Coolgardie; GES, Geraldton Sandplains; JAF, Jarrah Forest; MUR, Murchison; SWA, Swan Coastal Plain; YAL, Yalgoo.

**Figure 377.**
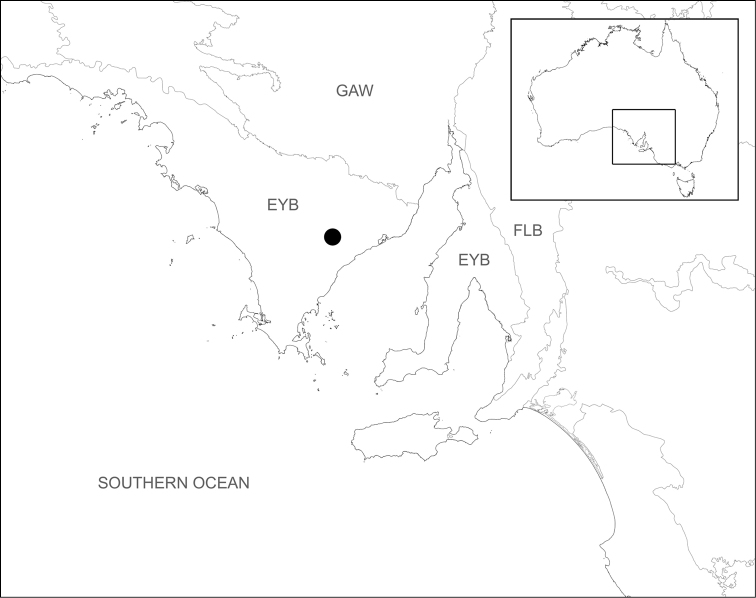
Map showing collection records of *Idiosoma corrugatum* from the Eyre Peninsula of South Australia. Relevant IBRA 7.0 bioregions are as follows: EYB, Eyre Yorke Block; FLB, Flinders Lofty Block; GAW, Gawler.

*Idiosoma sigillatum* (Figs 4–6), a member of the diverse *sigillatum*-clade (Fig. 25), has a relatively widespread although strictly bioregion- and substrate-specific distribution along the Swan Coastal Plain of south-western Western Australia, from Dalyellup north to at least Ledge Point (including Rottnest Island and Garden Island) (Fig. 375). The eastern limit of its range along the sandy foothills of the Darling Escarpment, from Boyanup north to at least Gingin, abuts the western limits of the ranges of *I. jarrah* and *I. mcclementsorum*. *Idiosoma sigillatum* is the dominant idiopid trapdoor spider on the Swan Coastal Plain, with a previously ubiquitous distribution throughout the Greater Perth region, where it can still be found in remnant habitats (e.g., Kings Park, Bold Park, and Shenton Park bushland). Burrows of this species usually occur in *Banksia* woodland and heathland on sandy soils, and are adorned with a typical ‘moustache-like’ arrangement of twig-lines (Figs 16, 17). Thanks to a detailed collection record of males submitted to the WAM since 1939, we now have a good understanding of the phenology of this species. The vast majority (85%) of males have been collected wandering in search of females in the cool (mostly winter) months of May–July, with a sudden peak in May corresponding to the first winter rains (Fig. 373).

#### Conservation assessment.

In 2017, *Idiosoma sigillatum* was assessed as Vulnerable (B1ab[ii-iv] + B2ab[ii-iv]) according to IUCN criteria (see Rix et al. 2017c). It has a known extent of occurrence of nearly 7,500 km^2^ [7,100 km^2^; with coastline as western margin including Rottnest and Garden Islands, and SWA bioregion boundary as eastern margin], and an area of occupancy within that range of < 3,000 km^2^ (see Rix et al. 2017c). Older museum records reflect the urban expansion of the Perth metropolitan region throughout the 20^th^ Century (Main 1990), and the spiders continue to be collected as remnant woodland habitats are cleared for development. Indeed, throughout most of its range, *I. sigillatum* is now locally extinct due to extensive land clearing. Further close assessment under both Criteria A and B will be crucial to the continued survival of this species.

### Idiosoma sp. indet.

Taxon classificationAnimaliaAraneaeIdiopidae

Fig. 375

Idiosoma ‘sigillatum’ Main, 1957b: 439 (in part; cited specimen from Koodiewoodie [sic] Ranges).

#### Material examined.

**AUSTRALIA: *Western Australia***: 1 ♀, Koodiwoodle Range (IBRA_SWA), 30°44'S, 115°41'E, 4 April 1953, B.Y. Main (WAM T144858).

#### Remarks.

A single female specimen in the *sigillatum*-clade, most similar to *I. mcclementsorum* and *I. sigillatum*, cannot be confidently assigned to a species in the absence of a male from this locality or molecular data. This specimen has features similar to both species, and is geographically isolated in a poorly collected area of the northern Swan Coastal Plain (Fig. 375). Additional specimens are required to confirm its identification.

## Supplementary Material

XML Treatment for Idiosoma

XML Treatment for Idiosoma nigrum

XML Treatment for Idiosoma arenaceum

XML Treatment for Idiosoma clypeatum

XML Treatment for Idiosoma corrugatum

XML Treatment for Idiosoma dandaragan

XML Treatment for Idiosoma formosum

XML Treatment for Idiosoma gardneri

XML Treatment for Idiosoma gutharuka

XML Treatment for Idiosoma incomptum

XML Treatment for Idiosoma intermedium

XML Treatment for Idiosoma jarrah

XML Treatment for Idiosoma kopejtkaorum

XML Treatment for Idiosoma kwongan

XML Treatment for Idiosoma mcclementsorum

XML Treatment for Idiosoma mcnamarai

XML Treatment for Idiosoma schoknechtorum

XML Treatment for Idiosoma sigillatum

XML Treatment for Idiosoma sp.

## Figures and Tables

**Figures 39–47.**
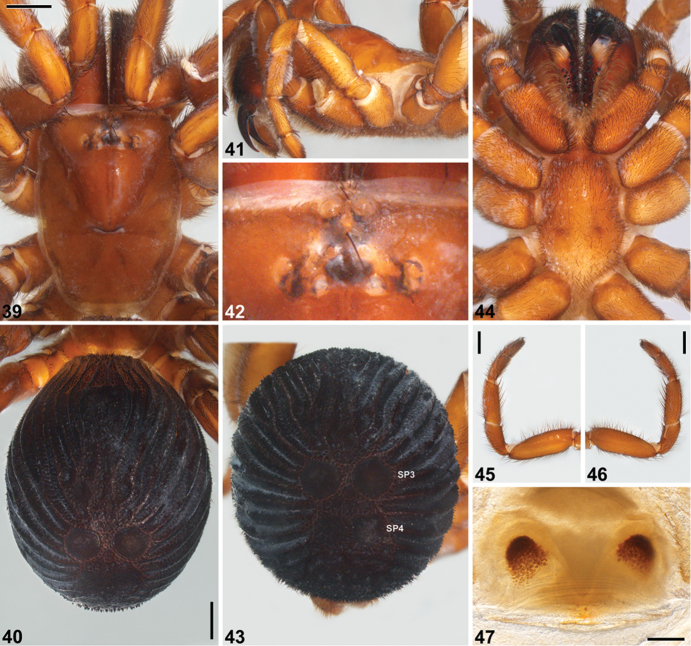
*Idiosoma nigrum* Main, 1952, female holotype (WAM T3960) from Wongan Hills (Western Australia; AVW). **39–40** Carapace and abdomen, dorsal view **41** Carapace, lateral view **42** Eyes, dorsal view **43** Abdomen, posterior view **44** Sternum, ventral view **45** Leg I, prolateral view **46** Leg I, retrolateral view **47** Spermathecae, dorsal view. Scale bars 2.0 **(39–40, 45–46**), 0.5 (**47**). SP3–4 = sigilla pairs 3–4.

## Supplementary materials

Supplementary material 1 Atlas of morphology Data type: Images Explanation note: Atlas of morphology for shield-backed trapdoor spiders of the *Idiosoma nigrum*-group, illustrating a representative selection of male specimens for each species, in five standard views. This is an open access article distributed under the terms of the Creative Commons Attribution License (CC BY 4.0), which permits unrestricted use, distribution, and reproduction in any medium, provided the original author and source are credited.Michael G. Rix, Joel A. Huey, Steven J.B. Cooper, Andrew D. Austin, Mark S. Harvey

Supplementary material 2 GenBank data Data type: Table Explanation note: Spreadsheet of specimens sequenced for the molecular analyses, with associated collection data and GenBank accession numbers. This is an open access article distributed under the terms of the Creative Commons Attribution License (CC BY 4.0), which permits unrestricted use, distribution, and reproduction in any medium, provided the original author and source are credited.Michael G. Rix, Joel A. Huey, Steven J.B. Cooper, Andrew D. Austin, Mark S. Harvey

Supplementary material 3
*COI* dataset Data type: Multiple sequence alignment Explanation note: Nexus file of the aligned COI dataset for 82 taxa. This is an open access article distributed under the terms of the Creative Commons Attribution License (CC BY 4.0), which permits unrestricted use, distribution, and reproduction in any medium, provided the original author and source are credited.Michael G. Rix, Joel A. Huey, Steven J.B. Cooper, Andrew D. Austin, Mark S. Harvey

Supplementary material 4 FULL dataset Data type: Multiple sequence alignment Explanation note: Nexus file of the aligned FULL dataset for 82 taxa. This is an open access article distributed under the terms of the Creative Commons Attribution License (CC BY 4.0), which permits unrestricted use, distribution, and reproduction in any medium, provided the original author and source are credited.Michael G. Rix, Joel A. Huey, Steven J.B. Cooper, Andrew D. Austin, Mark S. Harvey

Supplementary material 5
*COI* p-distances Data type: Table Explanation note: Spreadsheet with matrix of COI p-distances for 61 sequenced specimens of *Idiosoma* in the *nigrum*-group, along with a summary of mean inter- and intra-specific p-distances. This is an open access article distributed under the terms of the Creative Commons Attribution License (CC BY 4.0), which permits unrestricted use, distribution, and reproduction in any medium, provided the original author and source are credited.Michael G. Rix, Joel A. Huey, Steven J.B. Cooper, Andrew D. Austin, Mark S. Harvey
