# Supplementary material for: Conservation systematics of the shield-backed trapdoor spiders of the nigrum-group (Mygalomorphae, Idiopidae, Idiosoma): integrative taxonomy reveals a diverse and threatened fauna from south-western Australia
Source: Zookeys. 2018 May 9;(756):1–121. doi: 10.3897/zookeys.756.24397 (PMC5956031; doi:10.3897/zookeys.756.24397)
Supplement: Supplementary material 1 — Atlas of morphology [file zookeys-756-001-s001.pdf]

Atlas of Male Morphology:

Shield-Backed Trapdoor Spiders (*Idiosoma nigrum*-group)

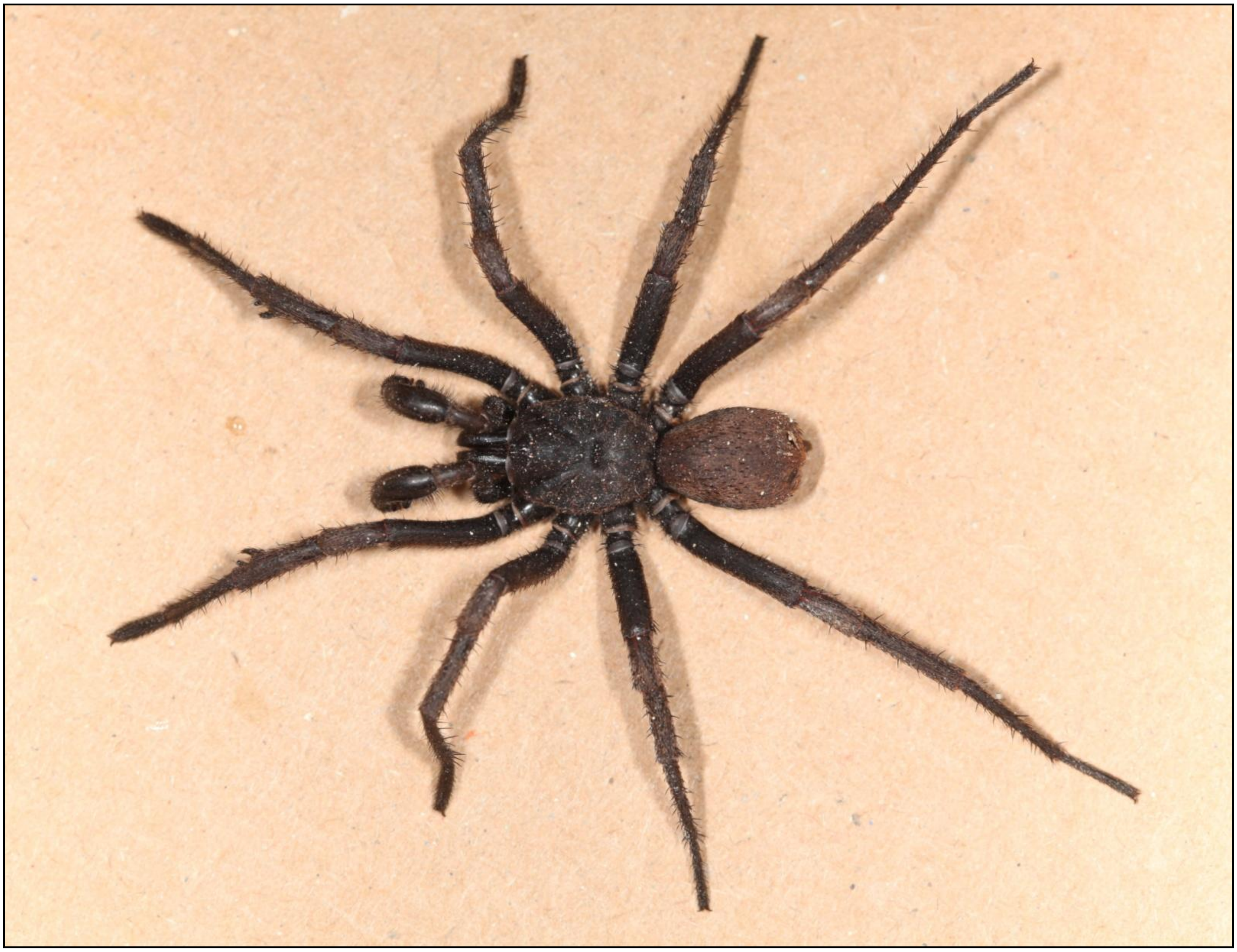

Live male *Idiosoma sigillatum* (O. P.-Cambridge, 1870) from Perth, Western Australia (image by M. Rix)

This document is published as an Appendix 1 supplement to:
Rix MG, Huey JA, Cooper SJB, Austin AD, Harvey MS (2018) Conservation systematics of the shield-backed trapdoor spiders of the '*nigrum*-group' (Mygalomorphae: Idiopidae: *Idiosoma*): integrative taxonomy reveals a diverse and threatened fauna from south-western Australia. ZooKeys.

INDEX

(alphabetical, by species, type material and site [after *I. nigrum*]; holotypes and paratypes* highlighted; sequenced specimens denoted by "DNA" superscripts)**

Idiosoma nigrum **Main, 1952**		Collection records 1
WAM T3301	*I. nigrum* (♂)*	WA: Walk Walkin, via Koorda 1
WAM T139511	*I. nigrum* (♂)	WA: Durokoppin Nature Reserve 1
WAM T139514	*I. nigrum* (♂)	WA: North Bungulla 2
WAM T139510	*I. nigrum* (♂)	WA: Walk Walkin Nature Reserve 2
WAM T139515	*I. nigrum* (♂)	WA: Wroth Road Nature Reserve 2
Idiosoma arenaceum **sp. n. [MYG478]**		Collection records 3
WAM T139527	*I. arenaceum* (♂)**	WA: Zuytdorp, site ZU1 3
WAM T41787	*I. arenaceum* (♂)*	WA: Zuytdorp, site ZU1 3
WAM T41364[DNA]	*I. arenaceum* (♂)	WA: Zuytdorp, site ZU3 4
WAM T41788	*I. arenaceum* (♂)	WA: Zuytdorp Nature Reserve, site ZU2 4
AMS KS17388	*I. arenaceum* (♂)	WA: Geraldton 4
WAM T27119	*I. arenaceum* (♂)	WA: Geraldton, Minnenooka 4
WAM T27123	*I. arenaceum* (♂)	WA: Northampton 5
Idiosoma clypeatum **sp. n. [MYG018]**		Collection records 6
WAM T96452[DNA]	*I. clypeatum* (♂)**	WA: Albion Downs 6
WAM T96467	*I. clypeatum* (♂)*	WA: Albion Downs 6
WAM T96463	*I. clypeatum* (♂)	WA: Albion Downs 7
WAM T139501	*I. clypeatum* (♂)	WA: Browns Soak, N. of Lake Barlee 7
WAM T98142	*I. clypeatum* (♂)	WA: Glen Station, off Kalli Road 7
WAM T136251	*I. clypeatum* (♂)	WA: Jack Hills, SW. of Mount Hale 7
WAM T136252[DNA]	*I. clypeatum* (♂)	WA: Jack Hills, SW. of Mount Hale 7
WAM T139505	*I. clypeatum* (♂)	WA: Urawa Nature Reserve, north 8
WAM T139506	*I. clypeatum* (♂)	WA: Weld Range North, site WN8 8
WAM T139503	*I. clypeatum* (♂)	WA: Weld Range North, site WN11 8
Idiosoma corrugatum **sp. n.**		Collection records 9
SAM NN29858	*I. corrugatum* (♂)**	SA: SW. of Kimba, Eyre Peninsula 9
Idiosoma dandaragan **sp. n. [MYG477]**		Collection records 10
WAM T139522	*I. dandaragan* (♂)**	WA: S. of Moora 10
WAM T139521	*I. dandaragan* (♂)*	WA: S. of Moora 10
WAM T139523	*I. dandaragan* (♂)*	WA: S. of Moora 11
WAM T139524	*I. dandaragan* (♂)*	WA: S. of Moora 11
WAM T139525	*I. dandaragan* (♂)*	WA: S. of Moora 11
WAM T139526	*I. dandaragan* (♂)*	WA: S. of Moora 11

INDEX (cont.)

***Idiosoma formosum* sp. n. [MYG262]**		Collection records	12
WAM T139470[DNA]	*I. formosum* (♂)**	WA: Mt Gibson	12
WAM T139495	*I. formosum* (♂)	WA: Dajoing Rock	12
WAM T139516a	*I. formosum* (♂)	WA: Mungarri Nature Reserve (N.)	13
WAM T139516b	*I. formosum* (♂)	WA: Mungarri Nature Reserve (N.)	13
***Idiosoma gardneri* sp. n. [MYG476]**		Collection records	14
WAM T139528	*I. gardneri* (♂)**	WA: Lesueur National Park	14
***Idiosoma gutharuka* sp. n. [MYG157]**		Collection records	15
WAM T38517	*I. gutharuka* (♂)**	WA: Gutha	15
***Idiosoma incomptum* sp. n. [MYG130]**		Collection records	16
WAM T99997[DNA]	*I. incomptum* (♂)**	WA: Carnarvon	16
WAM T98469	*I. incomptum* (♂)	WA: Boolathana Station	16
WAM T98474	*I. incomptum* (♂)	WA: Boolathana Station	17
WAM T98472	*I. incomptum* (♂)	WA: Bush Bay	17
WAM T98473a	*I. incomptum* (♂)	WA: Francois Peron National Park	17
WAM T98473b	*I. incomptum* (♂)	WA: Francois Peron National Park	17
WAM T98470	*I. incomptum* (♂)	WA: Nanga Station	18
WAM T98471	*I. incomptum* (♂)	WA: Zuytdorp	18
WAM T98475a	*I. incomptum* (♂)	WA: Zuytdorp	18
WAM T98475b	*I. incomptum* (♂)	WA: Zuytdorp	18
***Idiosoma intermedium* sp. n. [MYG475]**		Collection records	19
WAM T139520	*I. intermedium* (♂)**	WA: Bodallin	19
WAM T139494	*I. intermedium* (♂)	WA: Billiburning Rock	19
WAM T139517[DNA]	*I. intermedium* (♂)	WA: Mungarri Nature Reserve (S.)	20
WAM T139519[DNA]	*I. intermedium* (♂)	WA: Warrachuppin North Road	20
***Idiosoma jarrah* sp. n. [MYG156]**		Collection records	21
WAM T124143[DNA]	*I. jarrah* (♂)**	WA: Lesmurdie	21
WAM T74623	*I. jarrah* (♂)	WA: SW. of Boddington	21
WAM T99952[DNA]	*I. jarrah* (♂)	WA: SW. of Boddington	22
WAM T136943[DNA]	*I. jarrah* (♂)	WA: Bullsbrook, Smith Road	22
WAM T26822	*I. jarrah* (♂)	WA: Darlington	22
WAM T139475	*I. jarrah* (♂)	WA: Gooseberry Hill	22
WAM T63354	*I. jarrah* (♂)	WA: Mount Helena	23
WAM T18582	*I. jarrah* (♂)	WA: Mundaring Weir Road, Kalamunda	23
WAM T139474	*I. jarrah* (♂)	WA: Roleystone	23
WAM T30019	*I. jarrah* (♂)	WA: Talbot Road Reserve, site TR3	23

INDEX (cont.)

***Idiosoma kopejtkaorum* sp. n. [MYG521]** Collection records 24
WAM T144621 *I. kopejtkaorum* (♂)** WA: Snake Gully Nature Reserve.............. 24
WAM T139498 *I. kopejtkaorum* (♂)* WA: Snake Gully Nature Reserve.............. 24
WAM T139499 *I. kopejtkaorum* (♂) WA: Snake Gully Nature Reserve.............. 25
WAM T139497 *I. kopejtkaorum* (♂) WA: Lake Goorly (NW.) 25

***Idiosoma kwongan* sp. n. [MYG472]** Collection records 26
WAM T27142 *I. kwongan* (♂)** WA: E. of Greenhead................................ 26
WAM T27117[DNA] *I. kwongan* (♂) WA: Eneabba, AMC Minesite 26
WAM T27118 *I. kwongan* (♂) WA: Eneabba, AMC Minesite 27
WAM T139468[DNA] *I. kwongan* (♂) WA: Lesueur National Park........................ 27

***Idiosoma mcclementsorum* sp. n. [MYG474]** Collection records 28
WAM T139471 *I. mcclementsorum* (♂)** WA: Julimar State Forest 28
WAM T139472 *I. mcclementsorum* (♂)* WA: Julimar State Forest 28
WAM T29779 *I. mcclementsorum* (♂) WA: Toodyay, Bindoon Road.................... 29
WAM T44388 *I. mcclementsorum* (♂) WA: Toodyay 29

***Idiosoma mcnamarai* sp. n. [MYG520]** Collection records 30
WAM T26107[DNA] *I. mcnamarai* (♂)** WA: Trayning... 30
WAM T139518[DNA] *I. mcnamarai* (♂) WA: Bruce Rock-Doodlakine Road............ 30
WAM T44169 *I. mcnamarai* (♂) WA: East Yorkrakine Nature Reserve......... 31

***Idiosoma schoknechtorum* sp. n. [MYG518]** Collection records 32
WAM T139512 *I. schoknechtorum* (♂)** WA: Beverley... 32
WAM T27121 *I. schoknechtorum* (♂) WA: Meckering.. 32
WAM T139513 *I. schoknechtorum* (♂) WA: Westdale 33

***Idiosoma sigillatum* (O. P.-Cambridge, 1870)** Collection records 34
WAM T139480 *I. sigillatum* (♂) WA: Duncraig, Perth................................ 34
WAM T132564[DNA] *I. sigillatum* (♂) WA: Crawley, Perth 34
WAM T27984 *I. sigillatum* (♂) WA: Dardanup.. 35
WAM T18552 *I. sigillatum* (♂) WA: Garden Island [sic “Naval Base”]........ 35
WAM T31152 *I. sigillatum* (♂) WA: Gelorup... 35
WAM T27135 *I. sigillatum* (♂) WA: Kings Park, Perth.............................. 35
WAM T46829 *I. sigillatum* (♂) WA: Mandurah.. 36
WAM T41569 *I. sigillatum* (♂) WA: Mundijong 36
WAM T139486 *I. sigillatum* (♂) WA: Rottnest Island 36
WAM T18575 *I. sigillatum* (♂) WA: Yanchep Park 36

Idiosoma nigrum Main, 1952

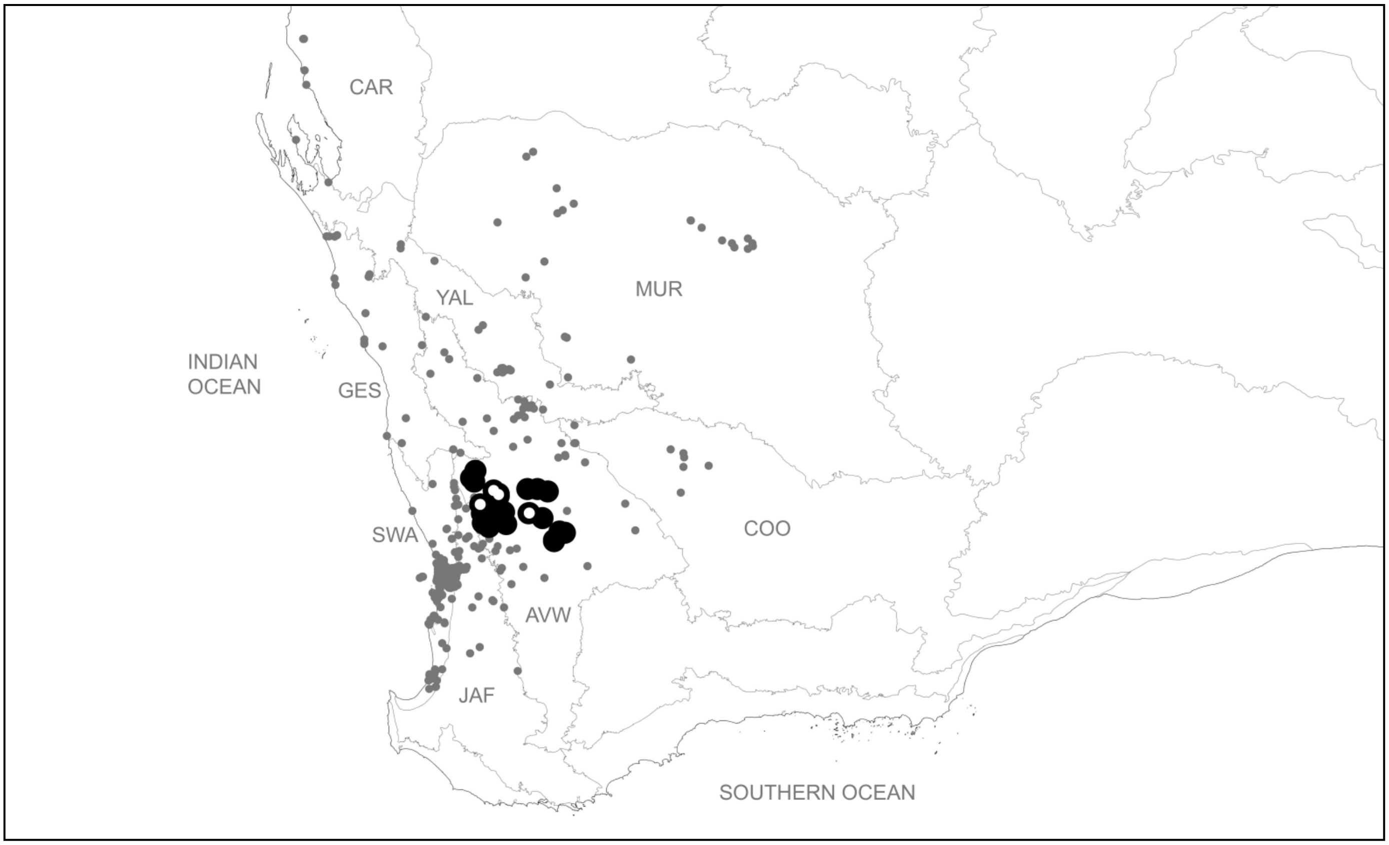

Collection records (open circles = DNA)

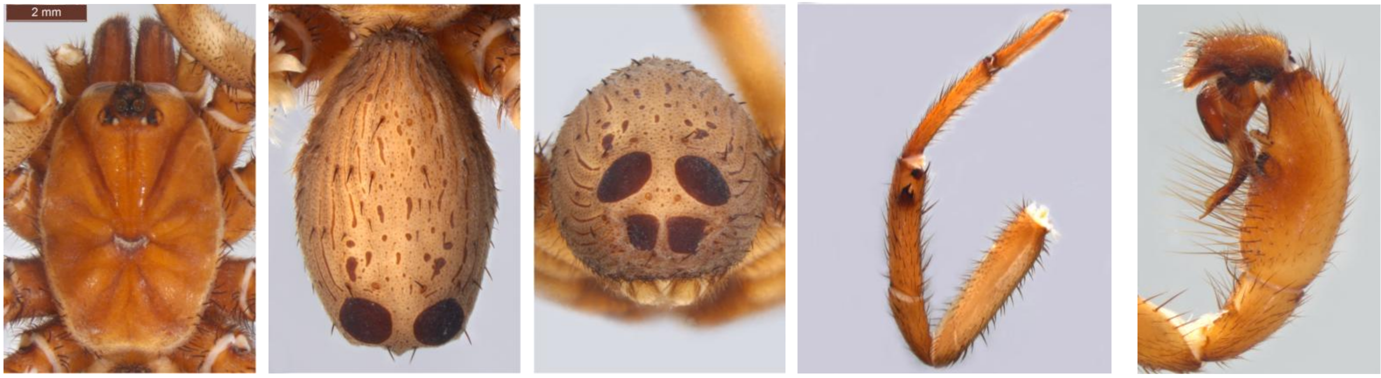

WAM T3301 male: Walk Walkin, via Koorda, WA [reference specimen]

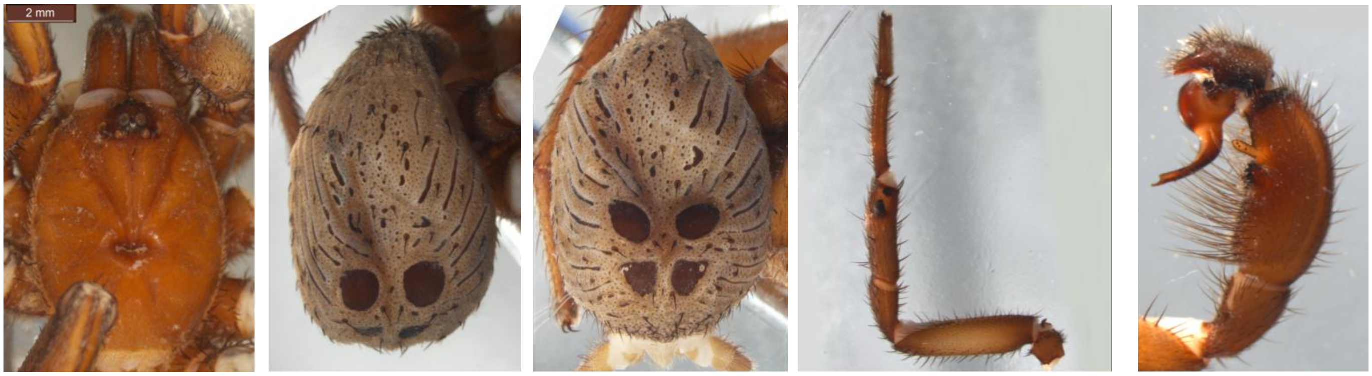

WAM T139511 male: Durokoppin Nature Reserve, WA

Idiosoma nigrum Main, 1952 (cont.)

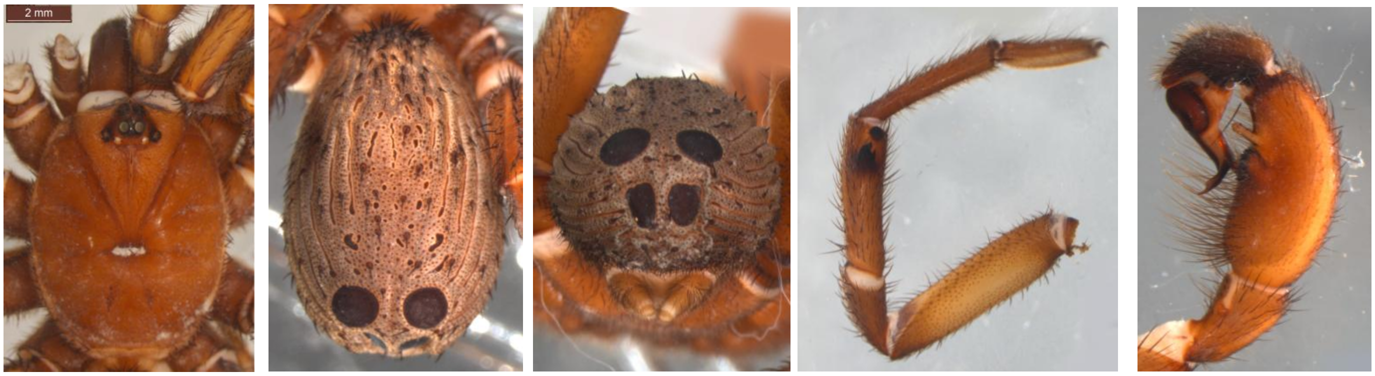

WAM T139514 male: North Bungulla, WA

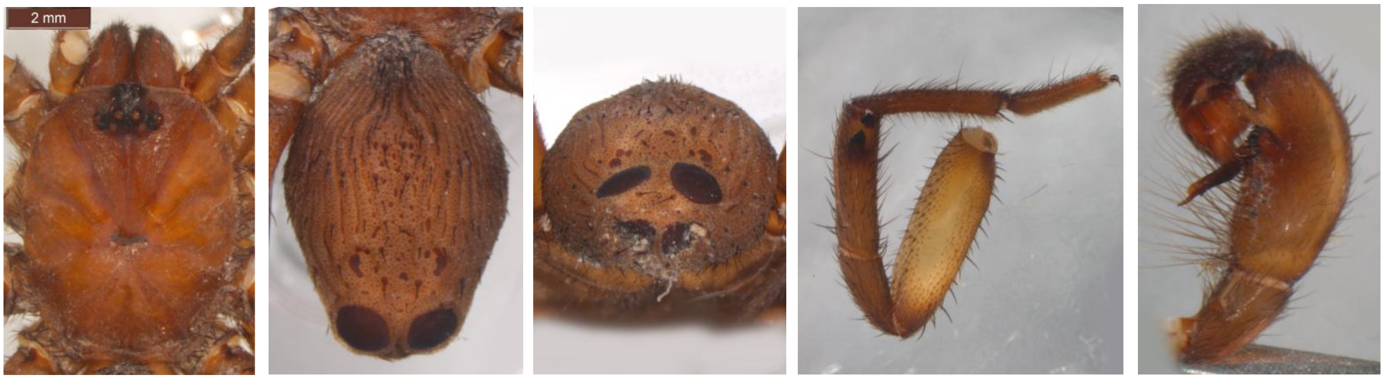

WAM T139510 male: Walk Walkin Nature Reserve, WA

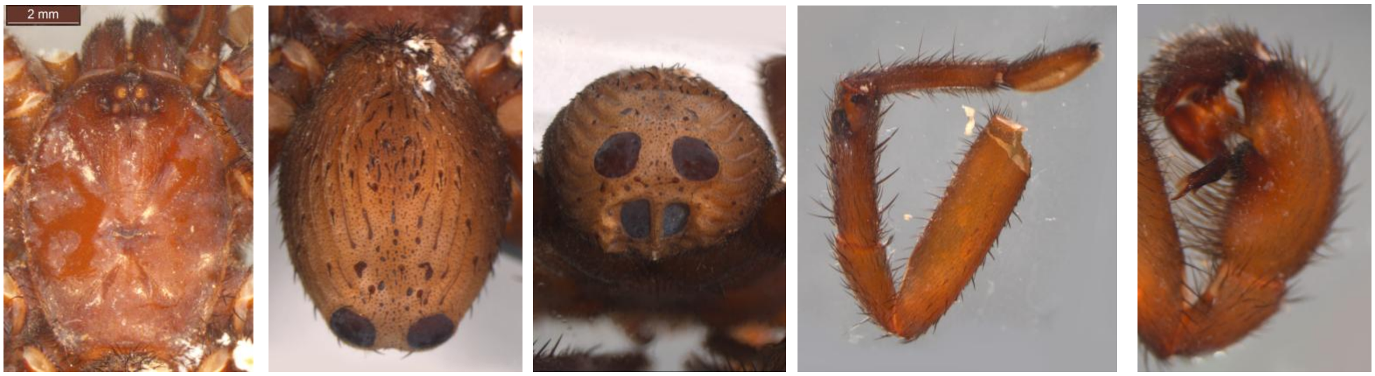

WAM T139515 male: Wroth Road Nature Reserve, WA

Idiosoma arenaceum sp. n. [MYG478]

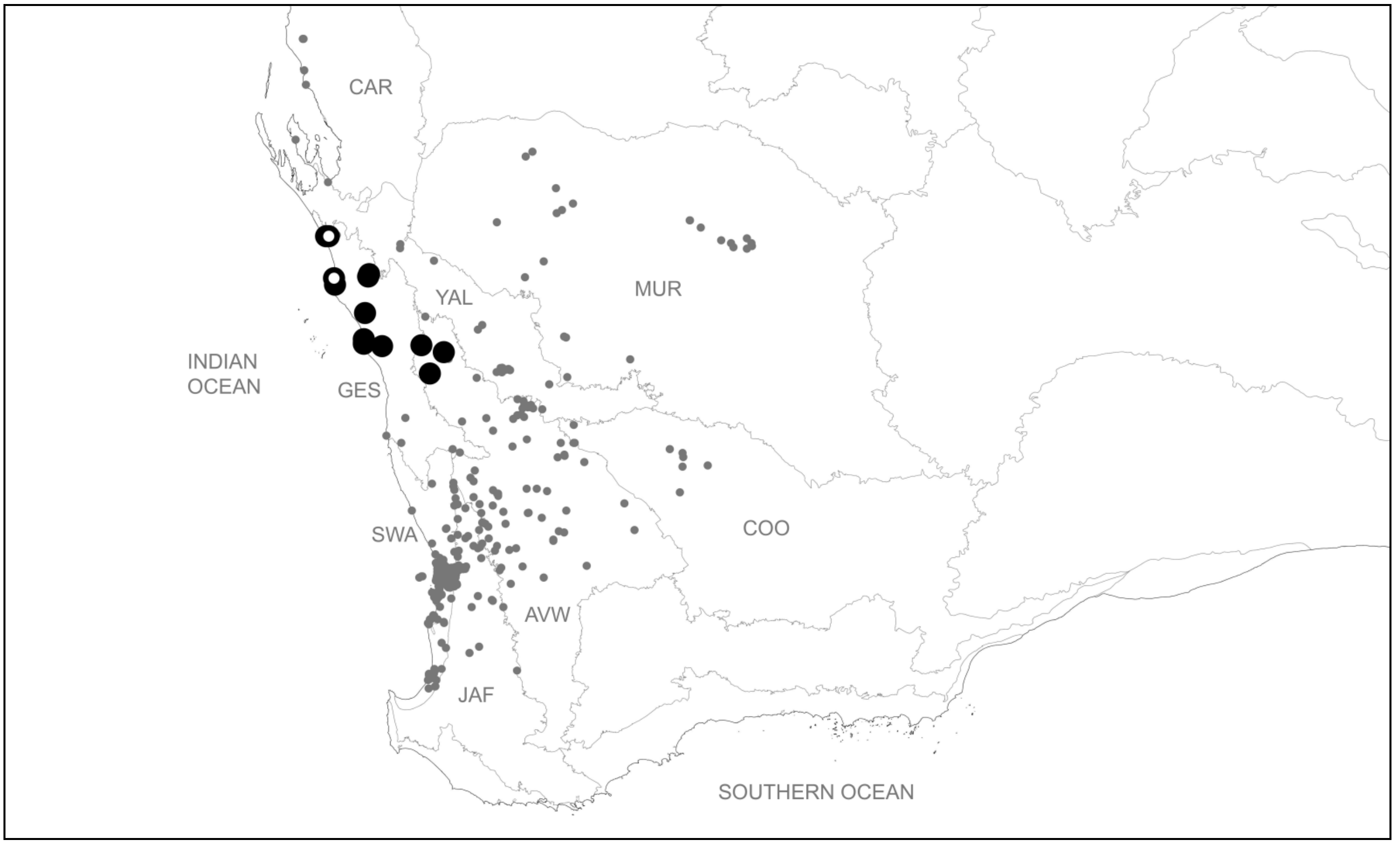

Collection records (open circles = DNA)

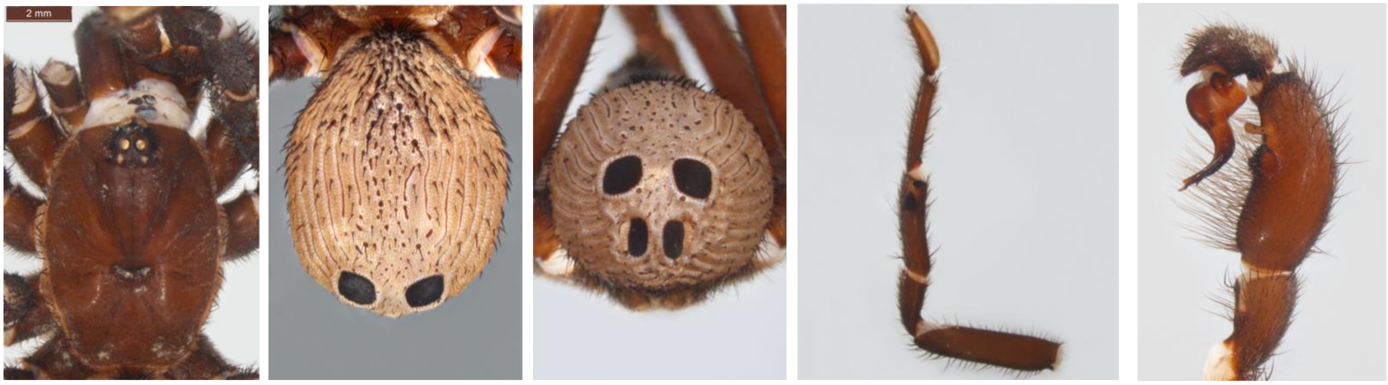

Holotype (WAM T139527) male: Zuytdorp, site ZU1, WA

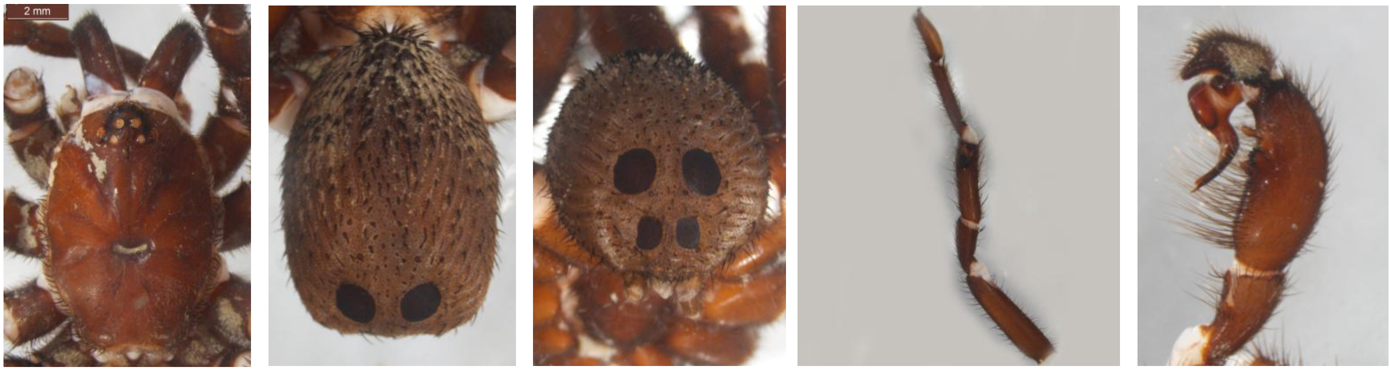

Paratype (WAM T41787) male: Zuytdorp, site ZU1, WA

Idiosoma arenaceum sp. n. [MYG478] (cont.)

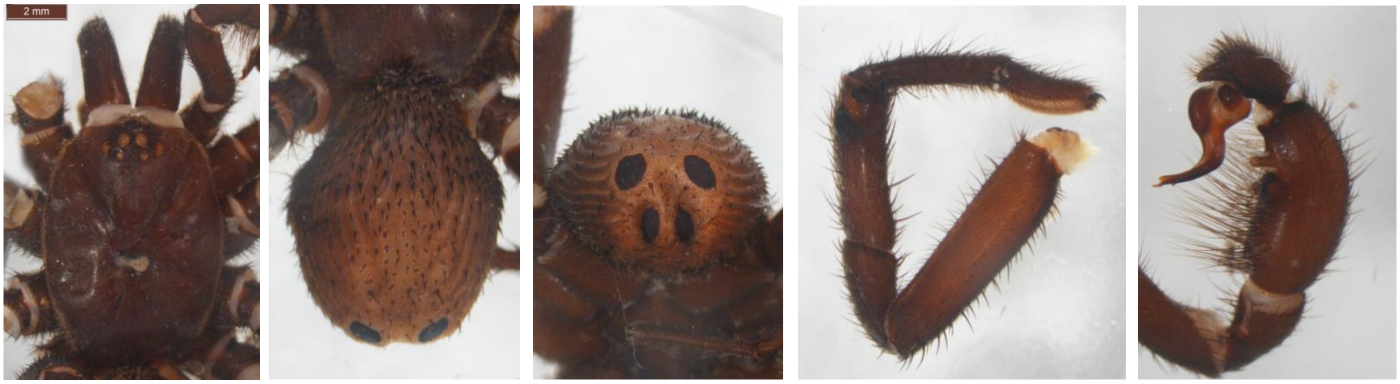

WAM T41364 male[DNA]: Zuytdorp, site ZU3, WA

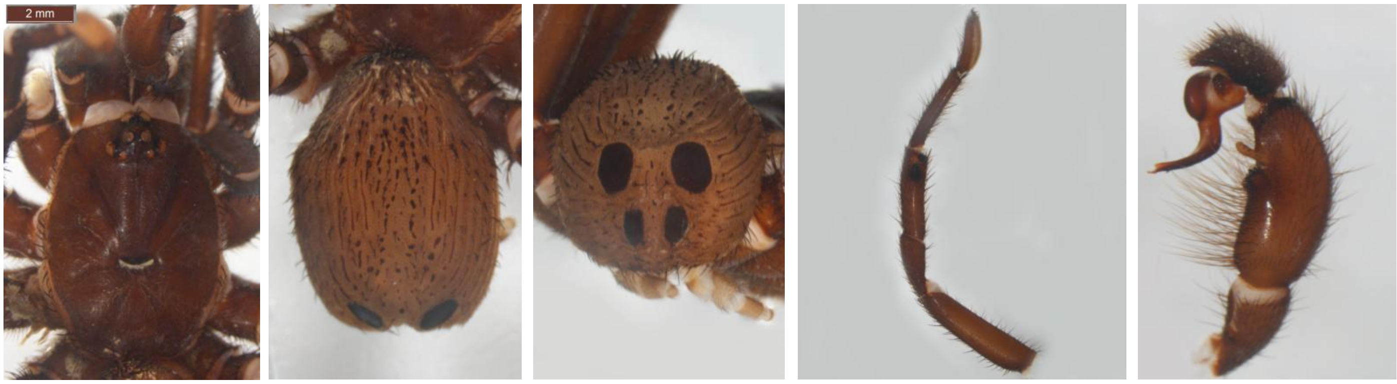

WAM T41788 male: Zuytdorp Nature Reserve, site ZU2, WA

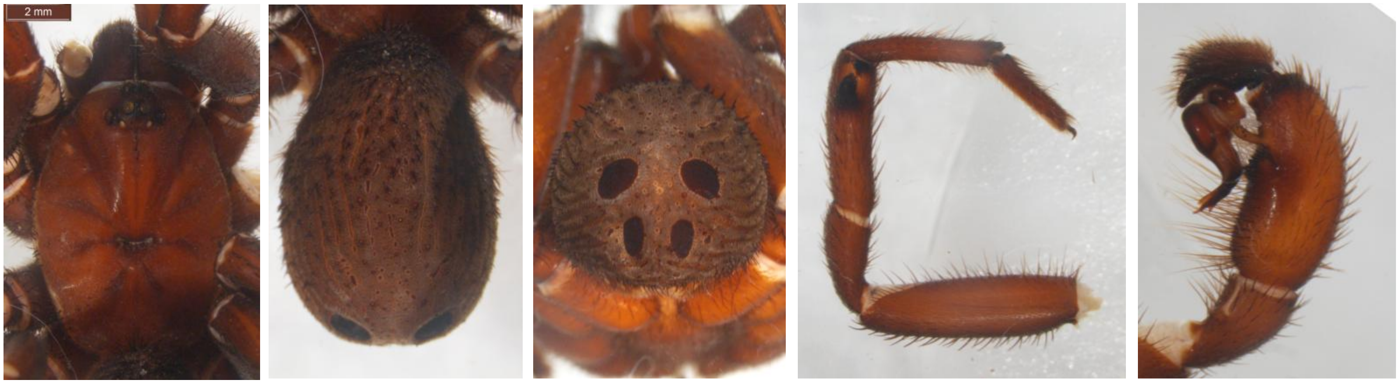

AMS KS17388 male: Geraldton, WA

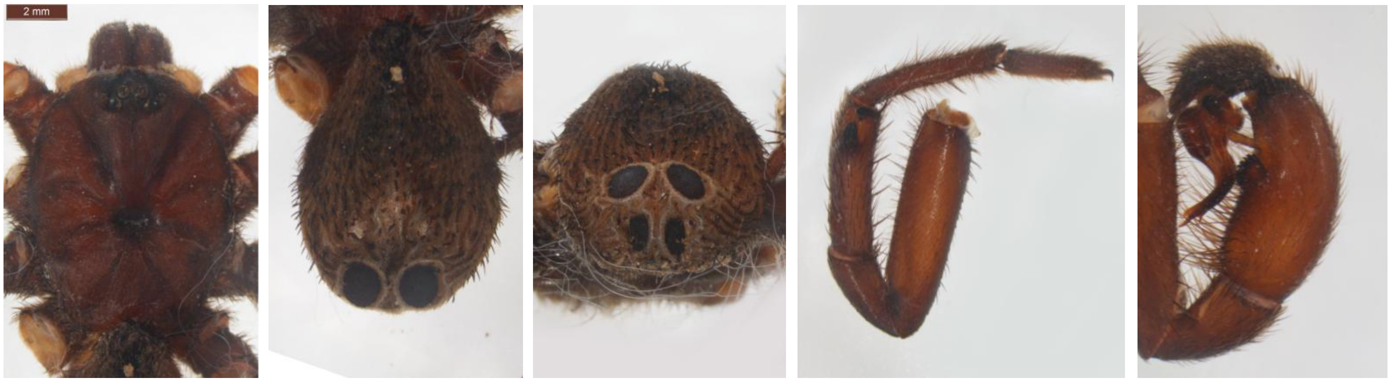

WAM T27119 male: Geraldton, Minnenooka, WA

Idiosoma arenaceum sp. n. [MYG478] (cont.)

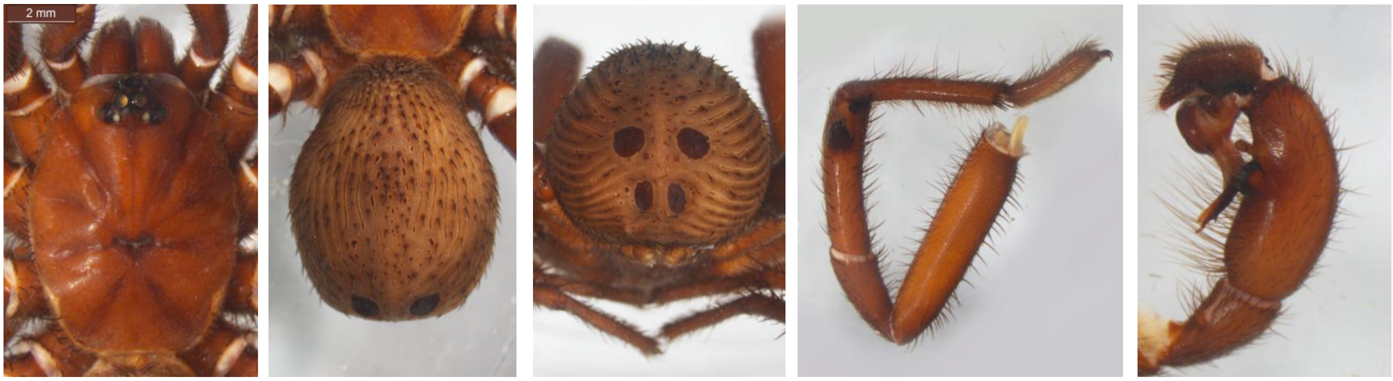

WAM T27123 male: Northampton, WA

Idiosoma clypeatum sp. n. [MYG018]

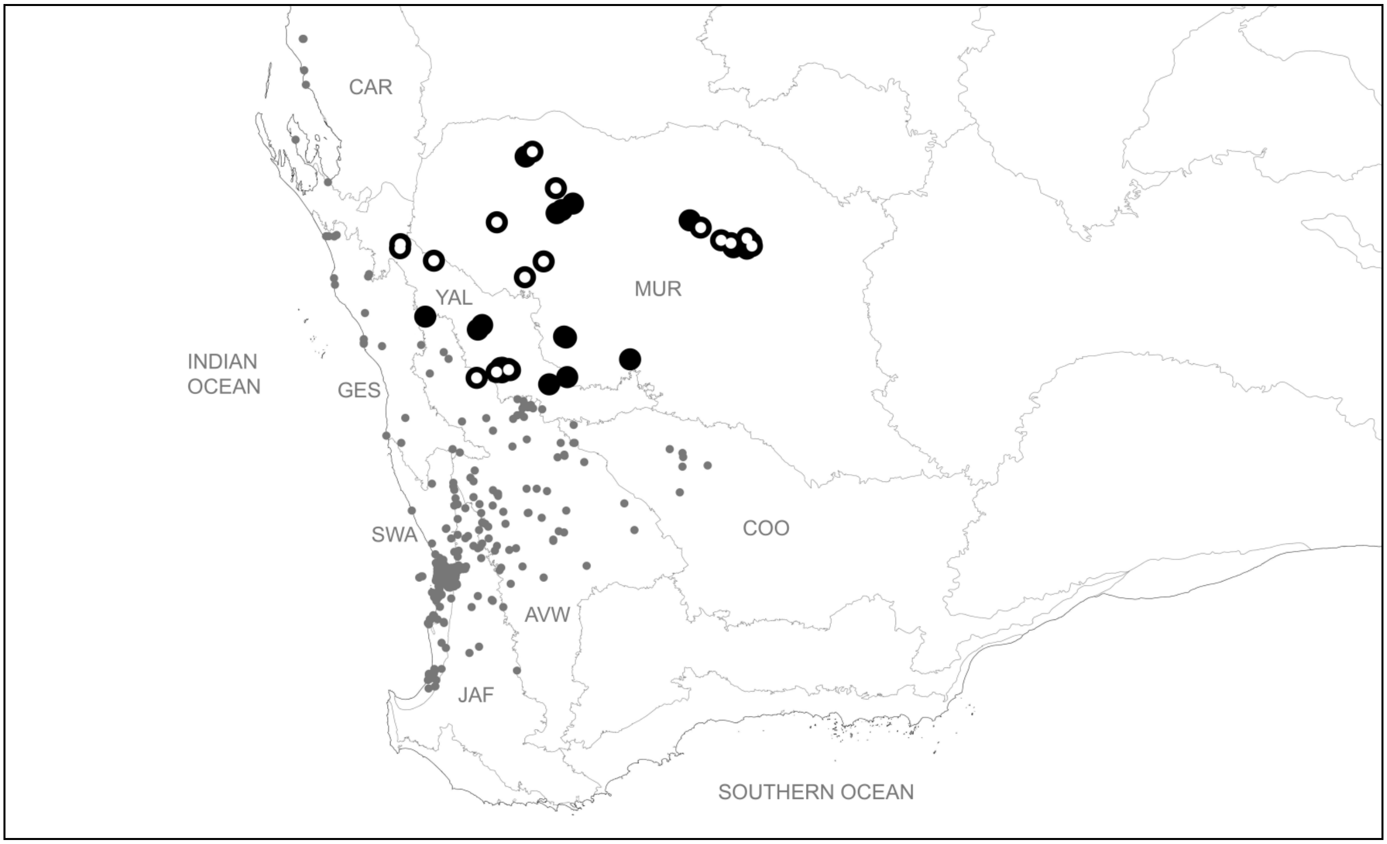

Collection records (open circles = DNA)

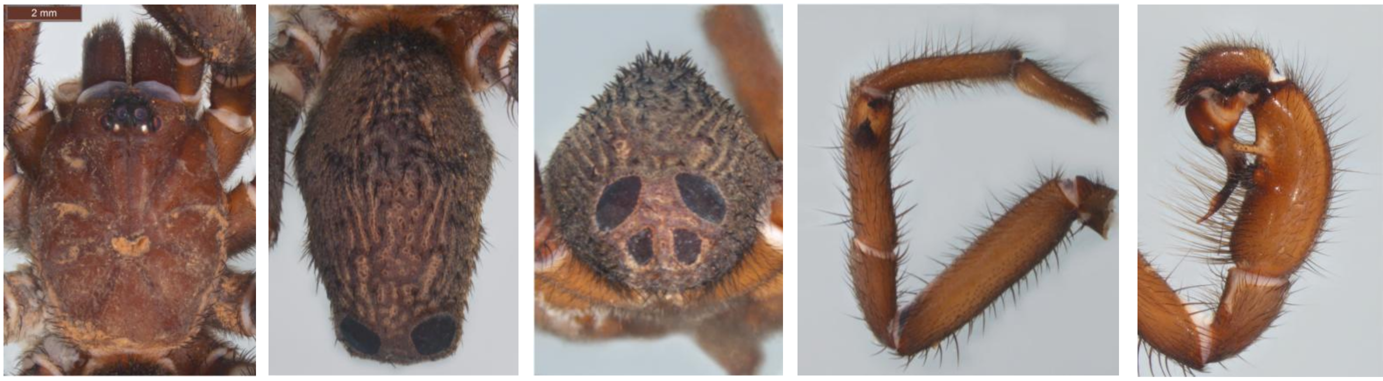

Holotype (WAM T96452) male[DNA]: Albion Downs, WA

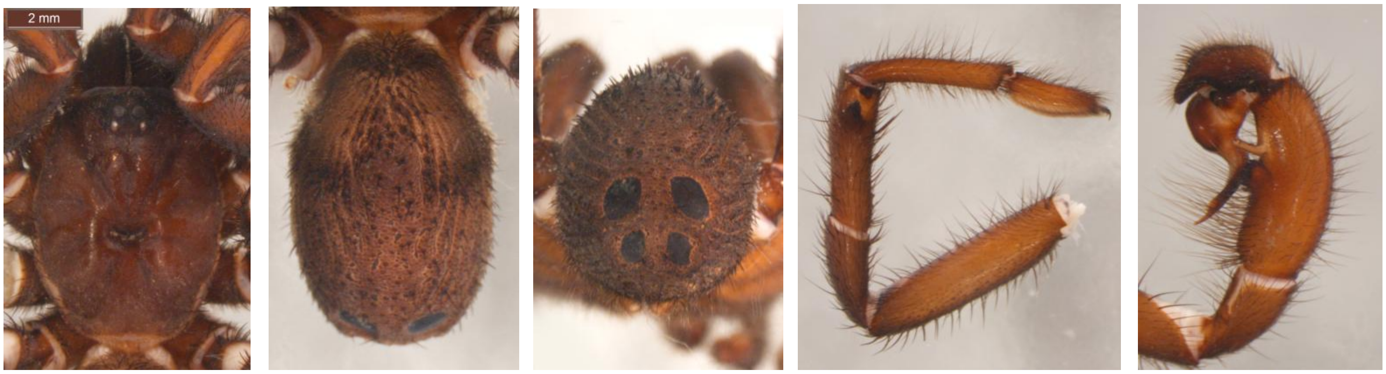

Paratype (WAM T96467) male: Albion Downs, WA

Idiosoma clypeatum sp. n. [MYG018] (cont.)

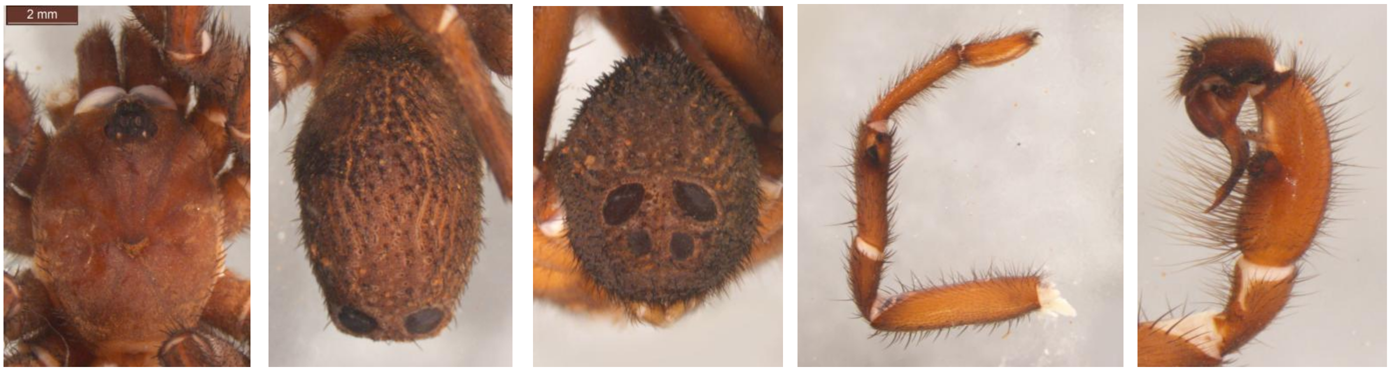

WAM T96463 male: Albion Downs, WA

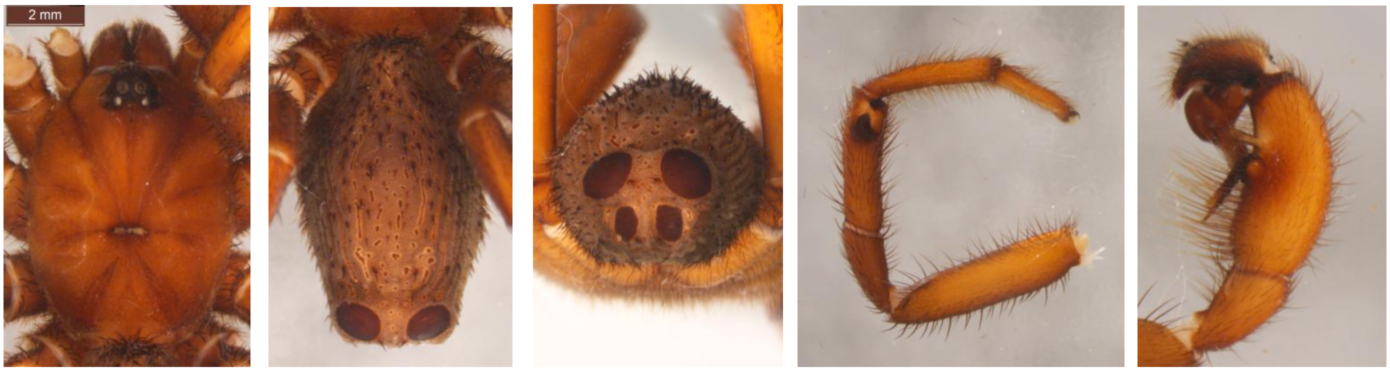

WAM T139501 male: Browns Soak, 20 miles N. of Lake Barlee, WA

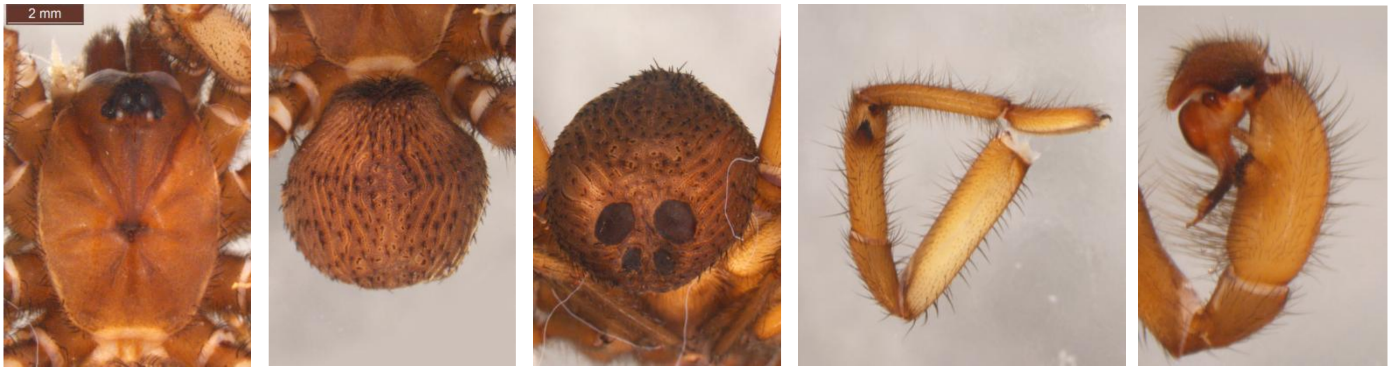

WAM T98142 male: Glen Station, off Kalli Road, WA

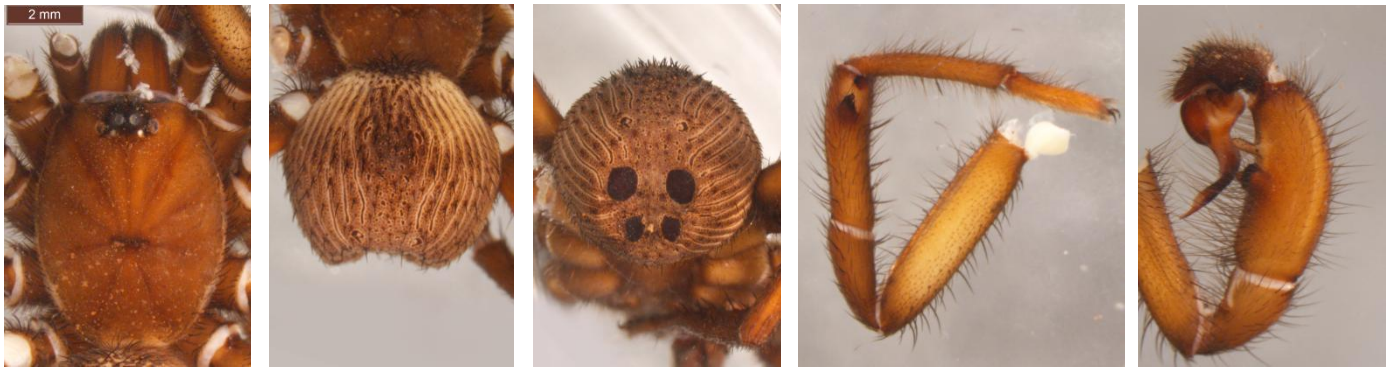

WAM T136251 male: Jack Hills, ca. 1 km SW. of Mount Hale, WA

Idiosoma clypeatum sp. n. [MYG018] (cont.)

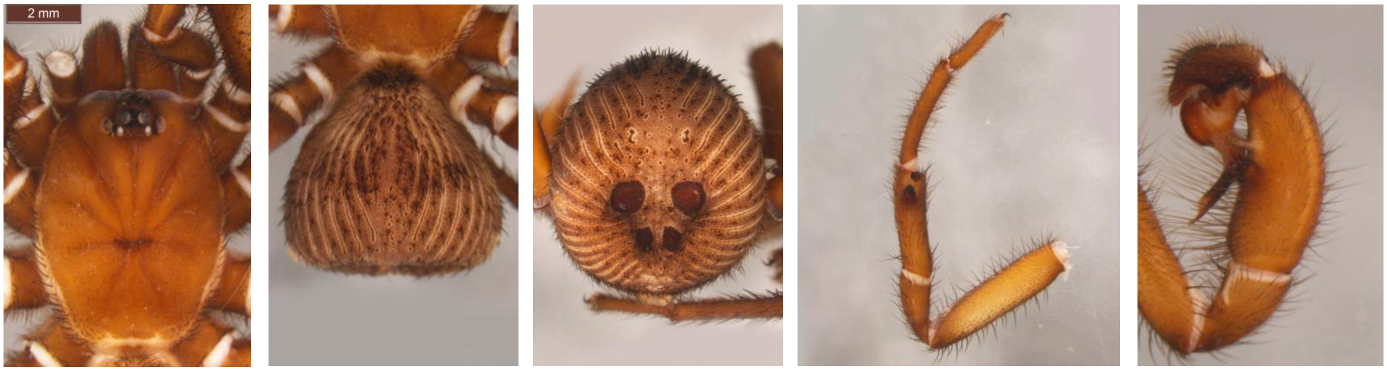

WAM T136252 male[DNA]: Jack Hills, ca. 1 km SW. of Mount Hale, WA

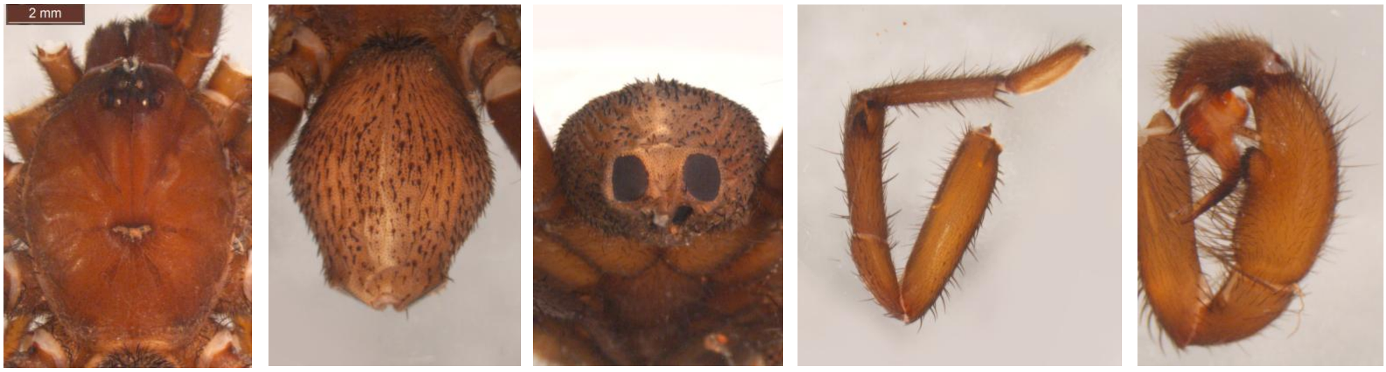

WAM T139505 male: Urawa Nature Reserve, north, site ML7, WA [*NB. right leg I flipped horizontal*]

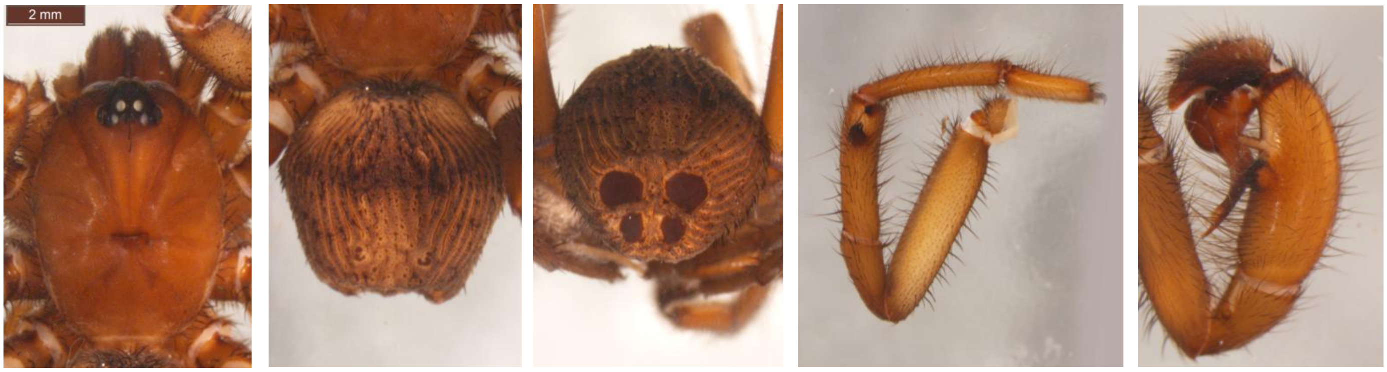

WAM T139506 male: Weld Range North, site WN8, WA

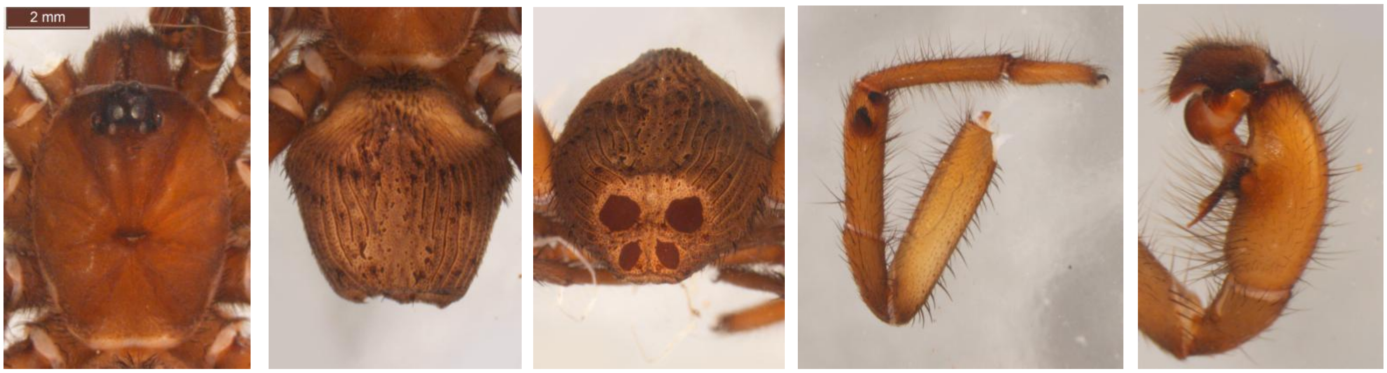

WAM T139503 male: Weld Range North, site WN11, WA

Idiosoma corrugatum sp. n.

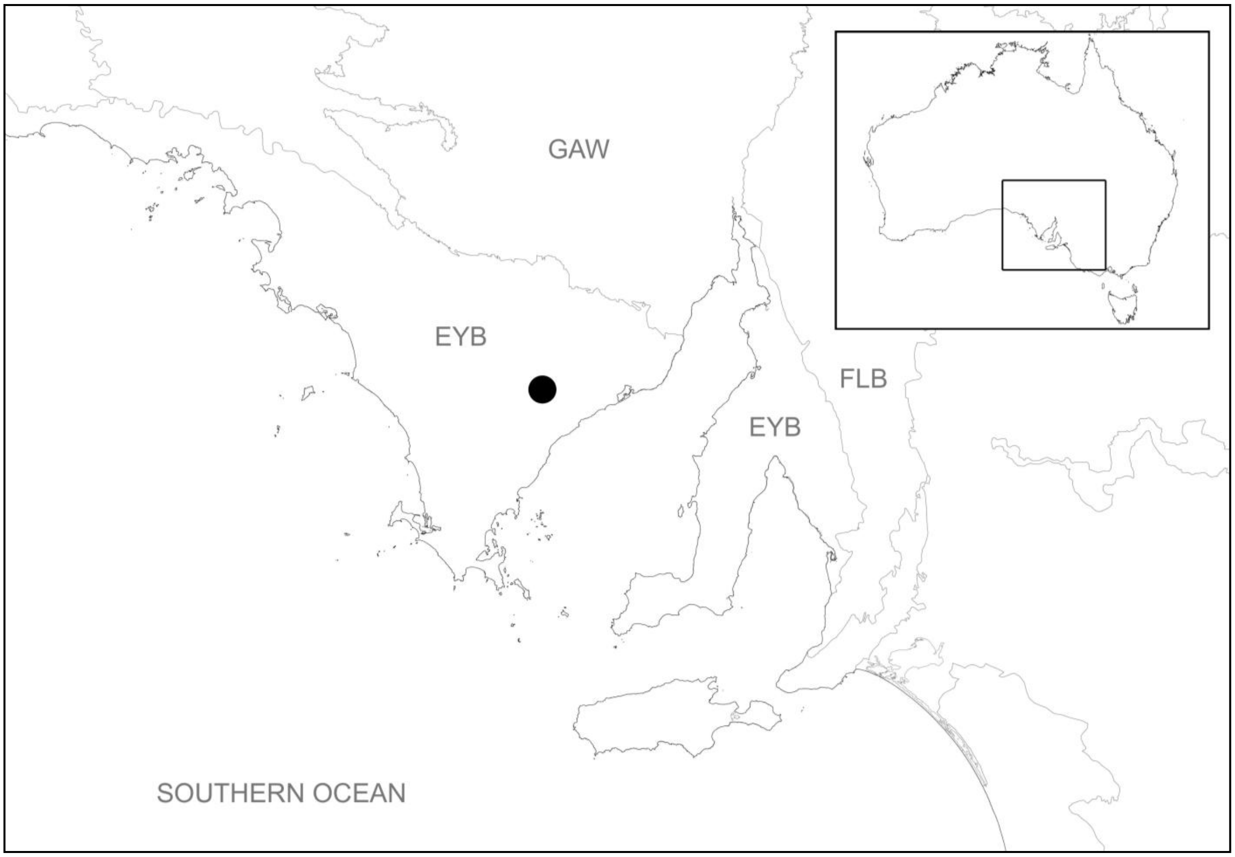

Collection records (NB. not DNA sequenced)

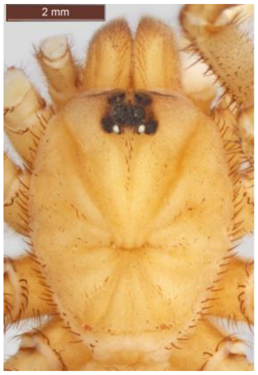

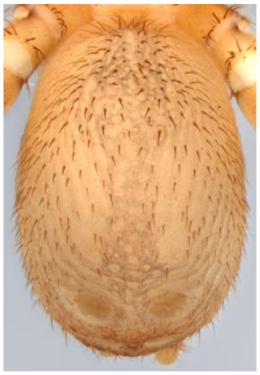
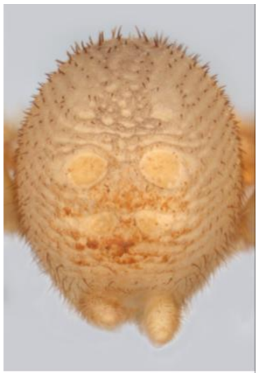
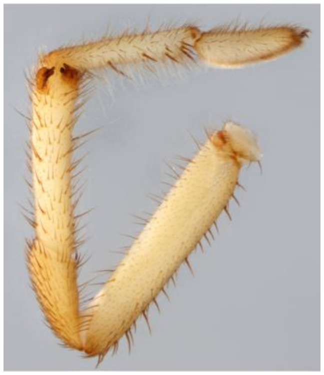
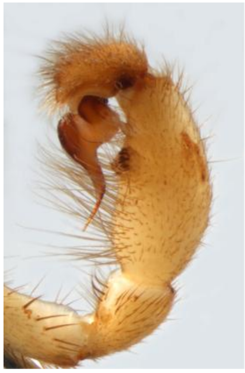

Holotype (SAM NN29858) male: SW. of Kimba, Eyre Peninsula, SA

Idiosoma dandaragan sp. n. [MYG477]

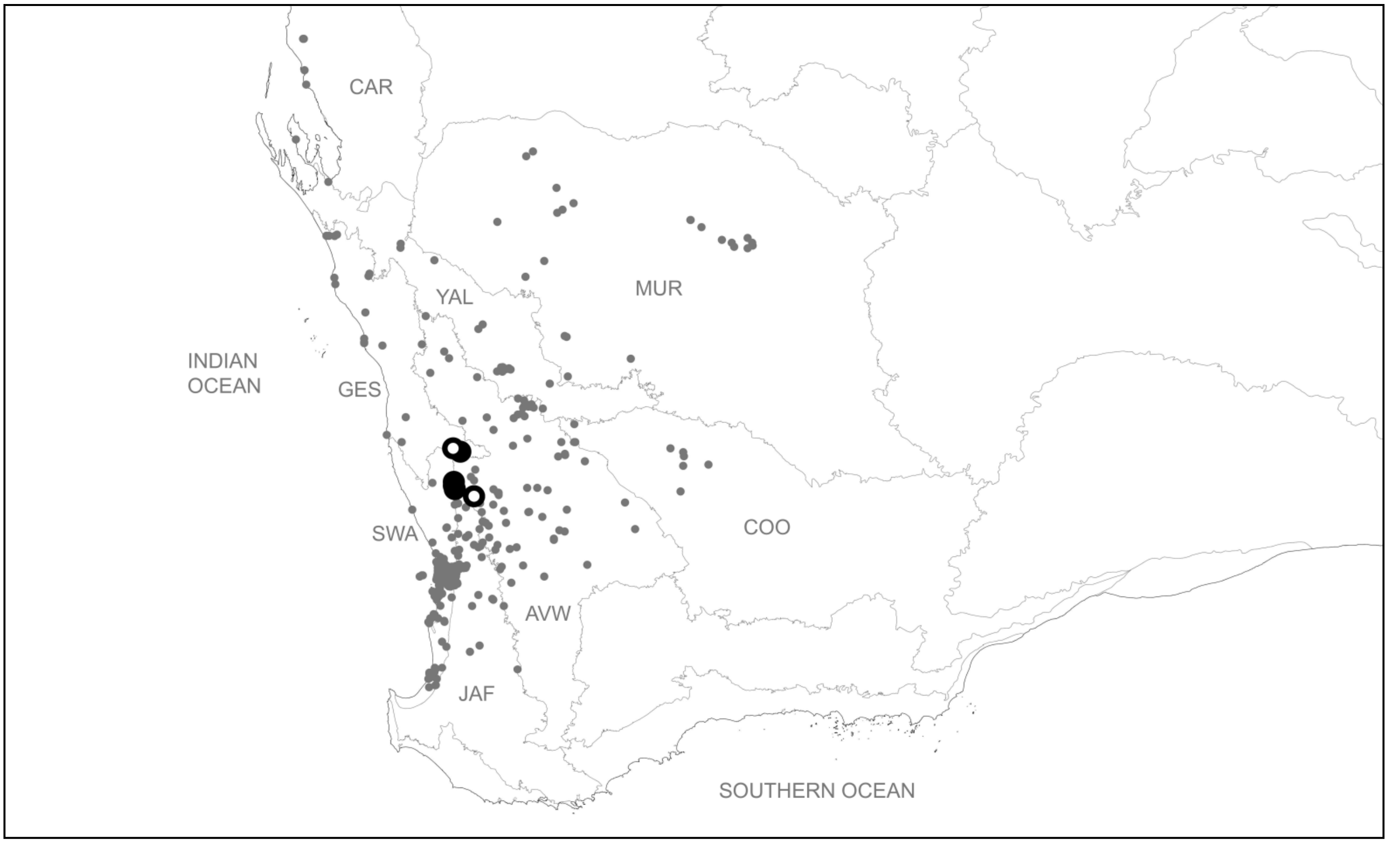

Collection records (open circles = DNA)

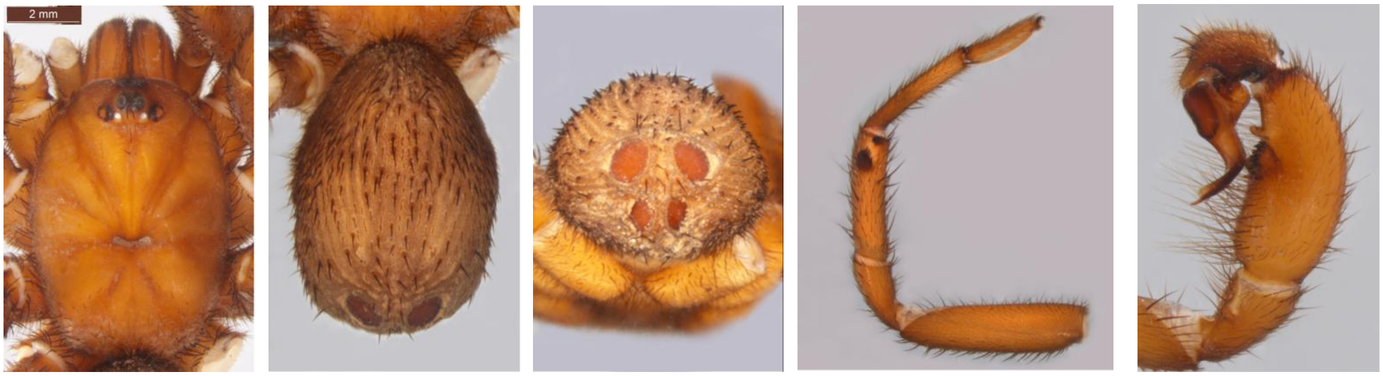

Holotype (WAM T139522) male: S. of Moora on Mogumber Road, WA

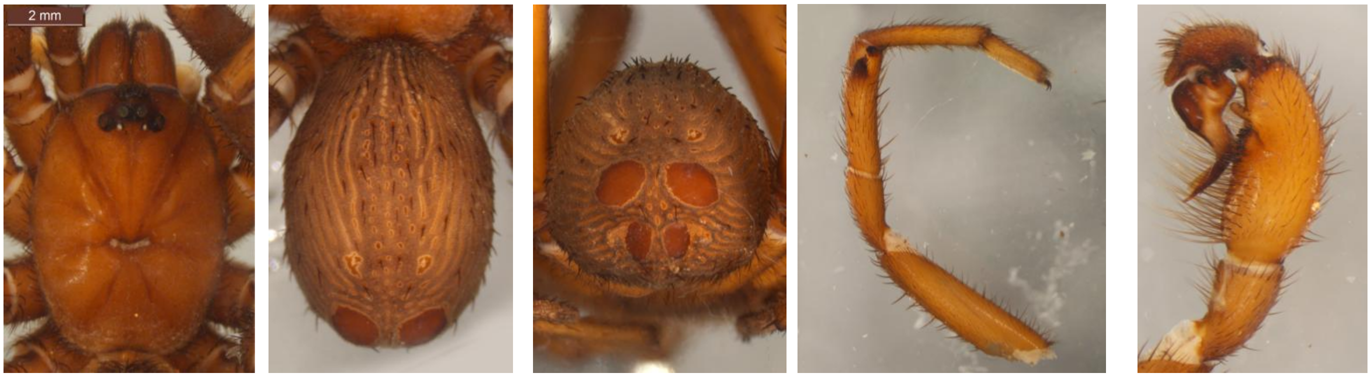

WAM T139521 male: S. of Moora on Mogumber Road, WA

Idiosoma dandaragan sp. n. [MYG477] (cont.)

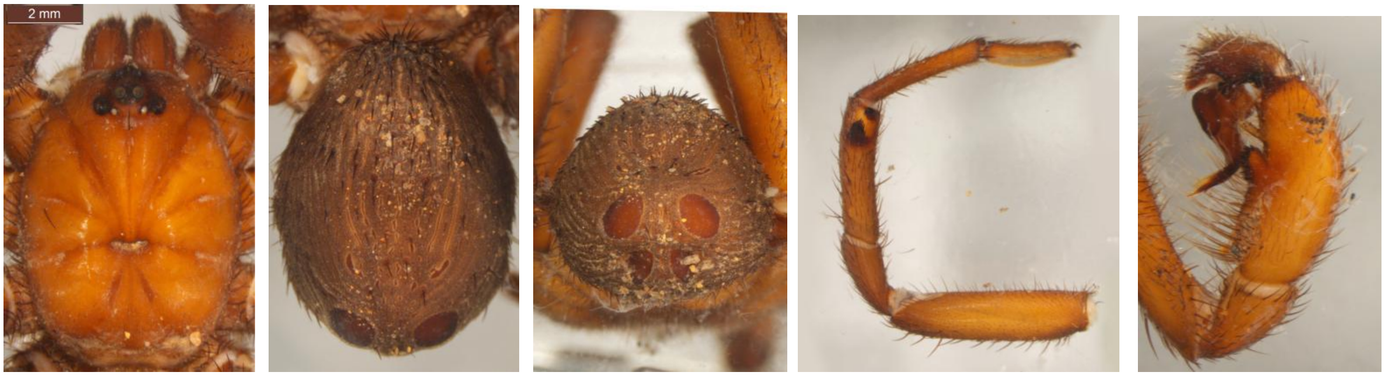

WAM T139523 male: S. of Moora on Mogumber Road, WA

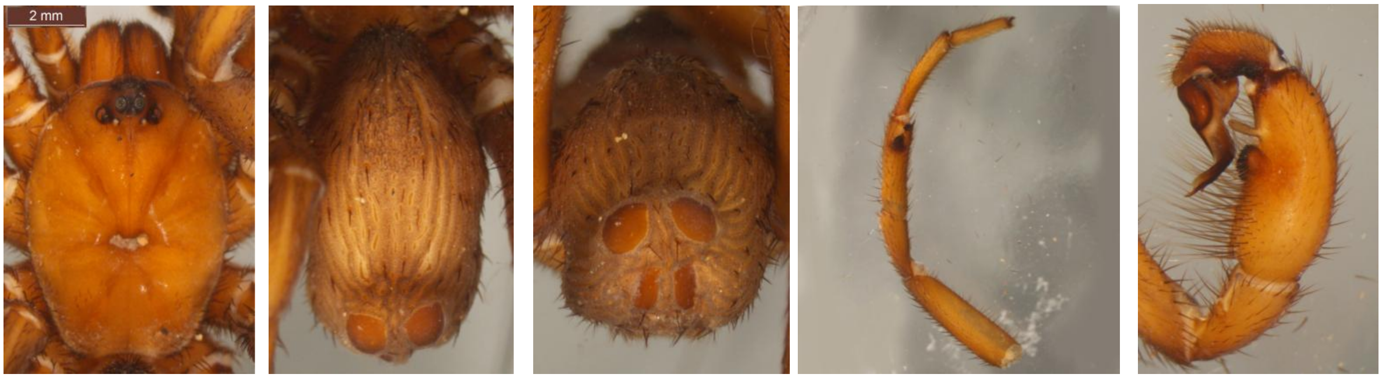

WAM T139524 male: S. of Moora on Mogumber Road, WA

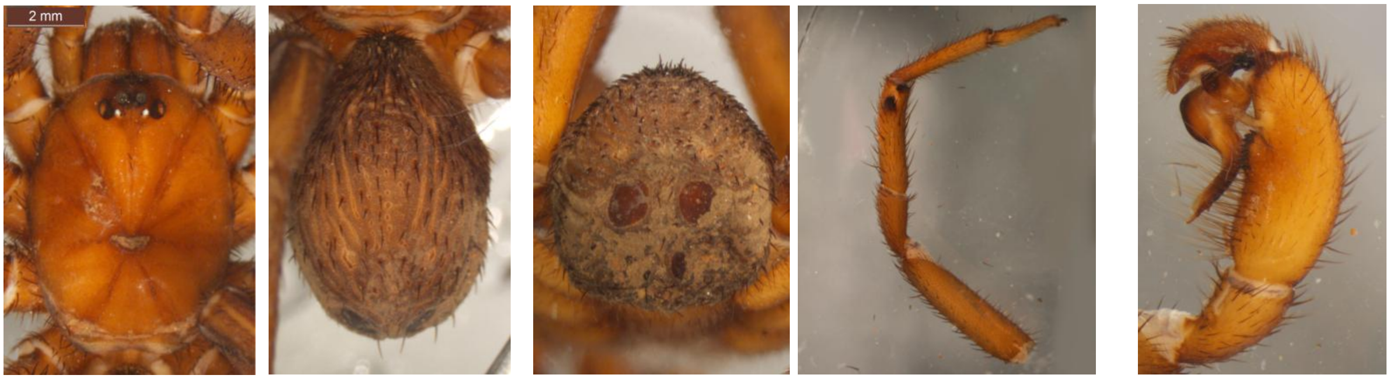

WAM T139525 male: S. of Moora on Mogumber Road, WA

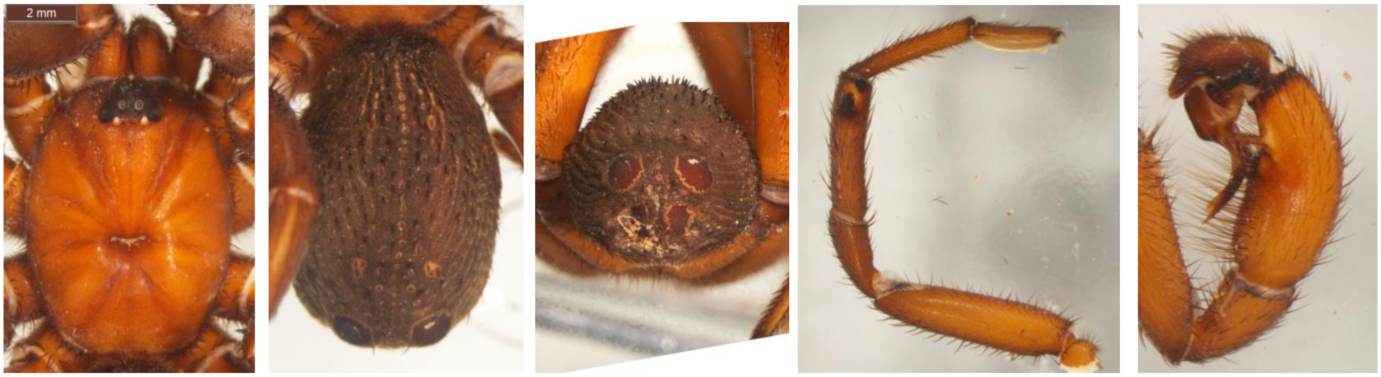

WAM T139526 male: S. of Moora on Mogumber Road, WA

Idiosoma formosum sp. n. [MYG262]

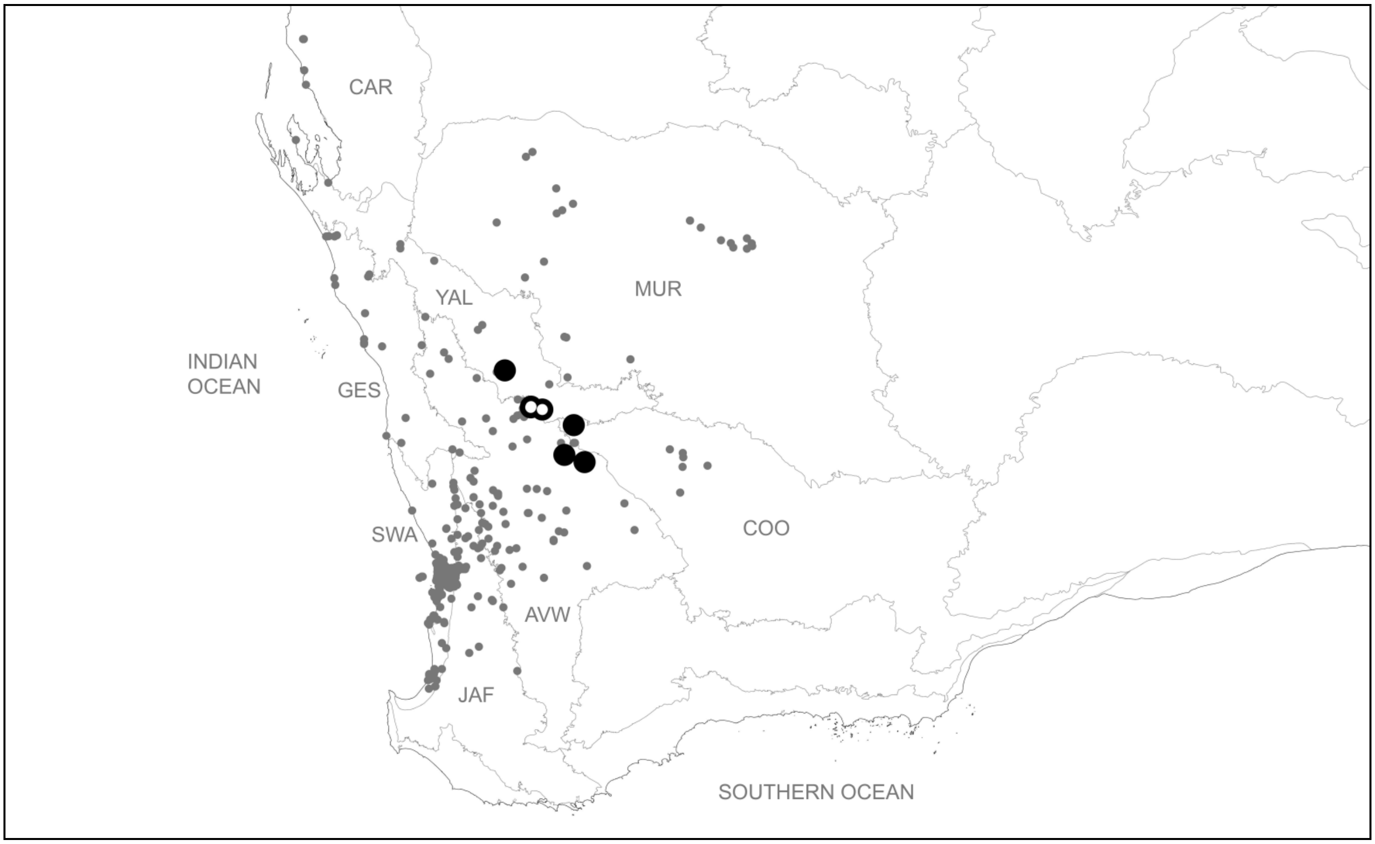

Collection records (open circles = DNA)

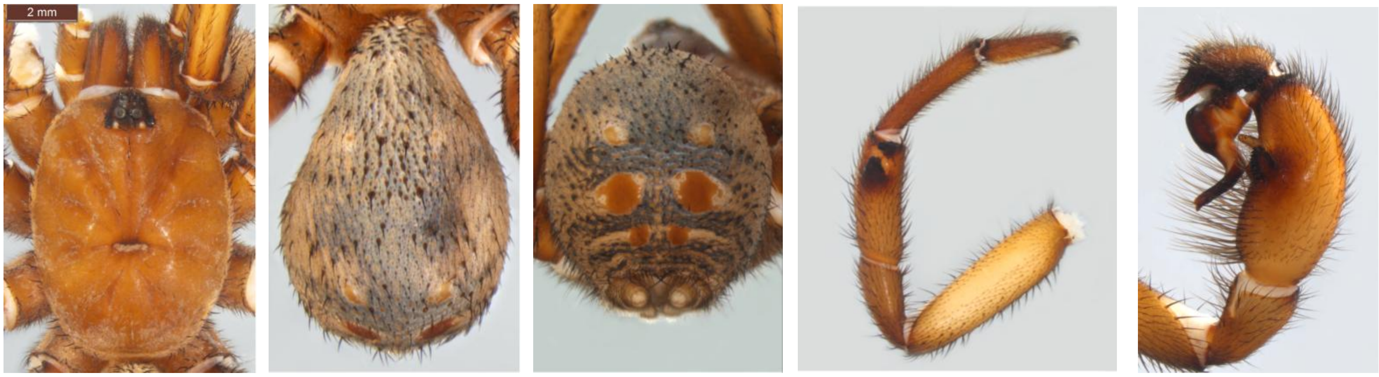

Holotype (WAM T139470) male[DNA]: Mt Gibson, WA

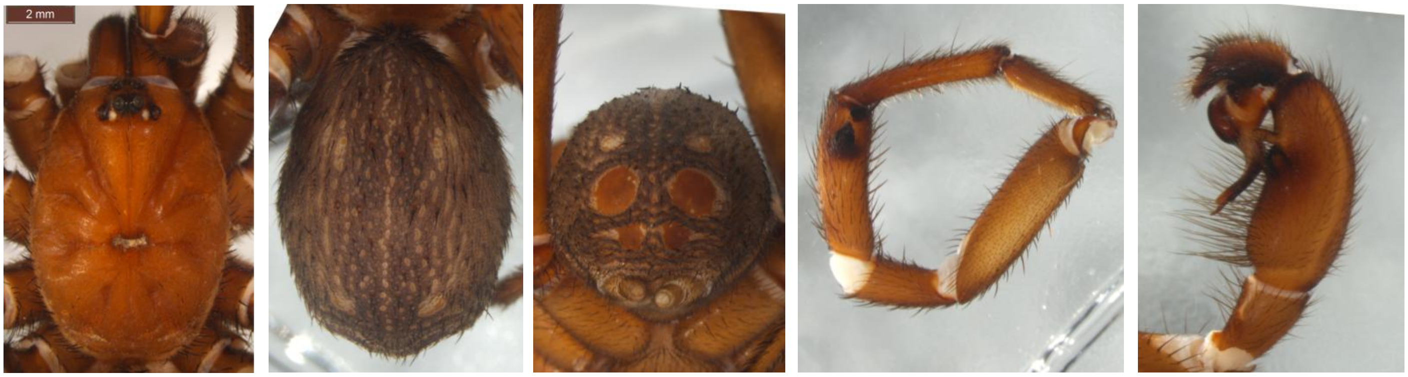

WAM T139495 male: Dajoing Rock, WA

Idiosoma formosum sp. n. [MYG262] (cont.)

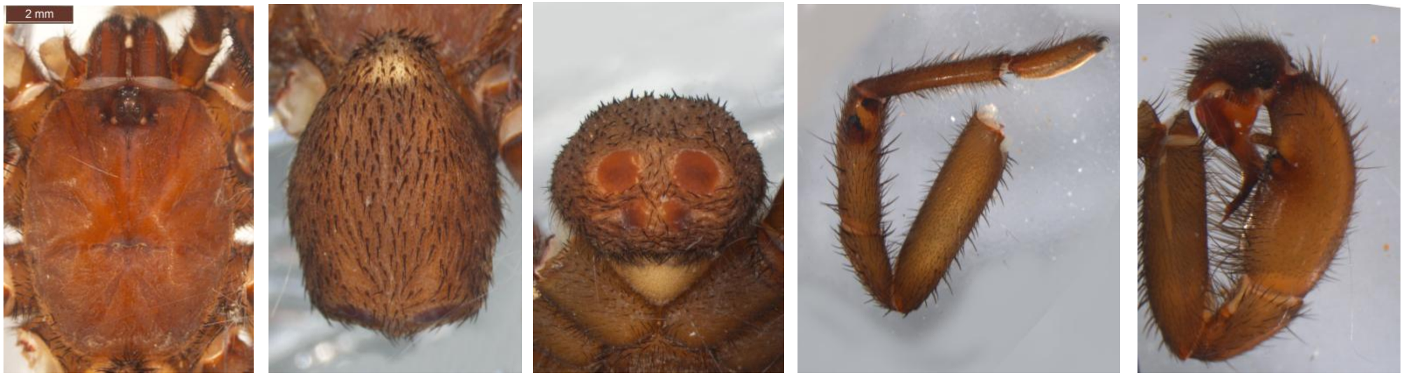

WAM T139516a male: Mungarri Nature Reserve (North), WA

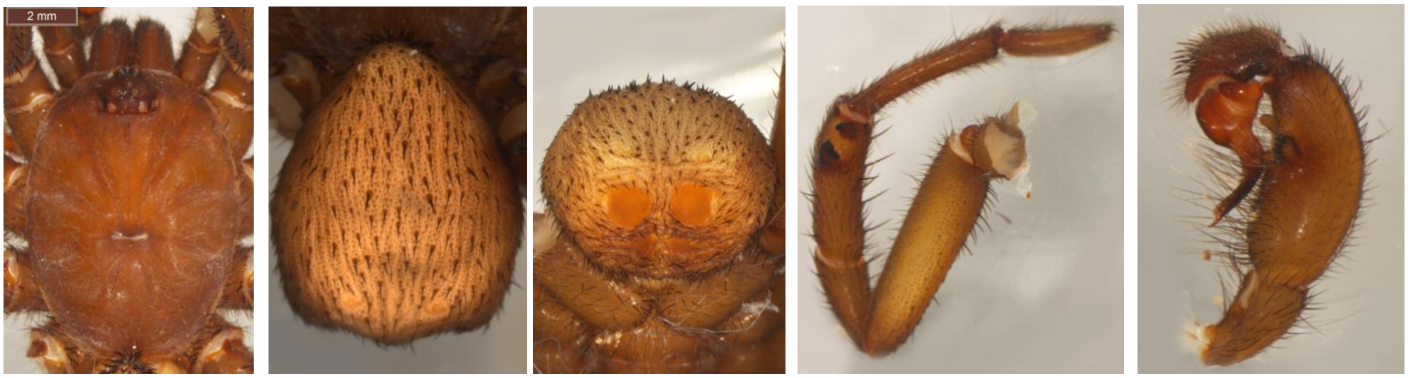

WAM T139516b male: Mungarri Nature Reserve (North), WA

Idiosoma gardneri sp. n. [MYG476]

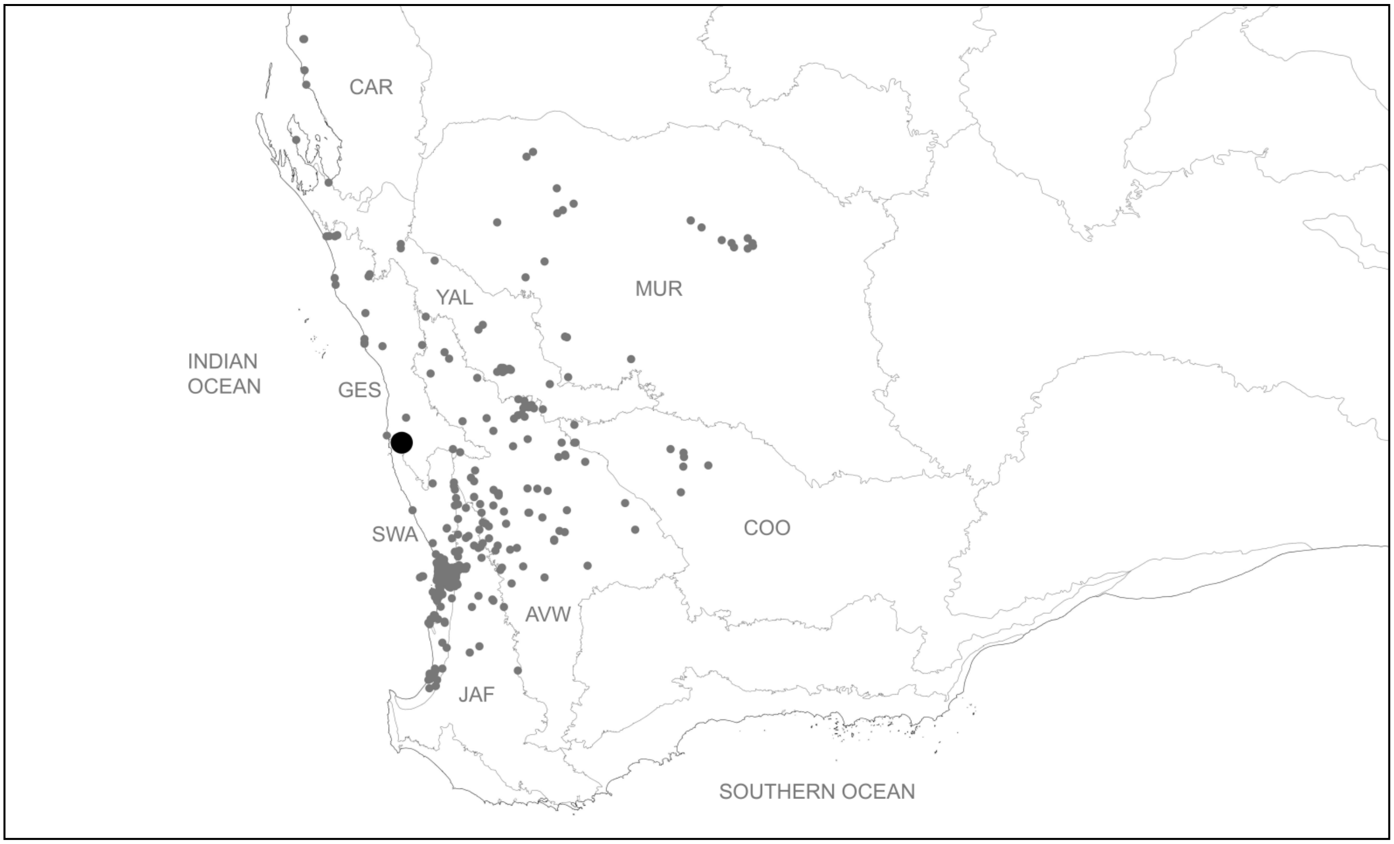

Collection records (NB. not DNA sequenced)

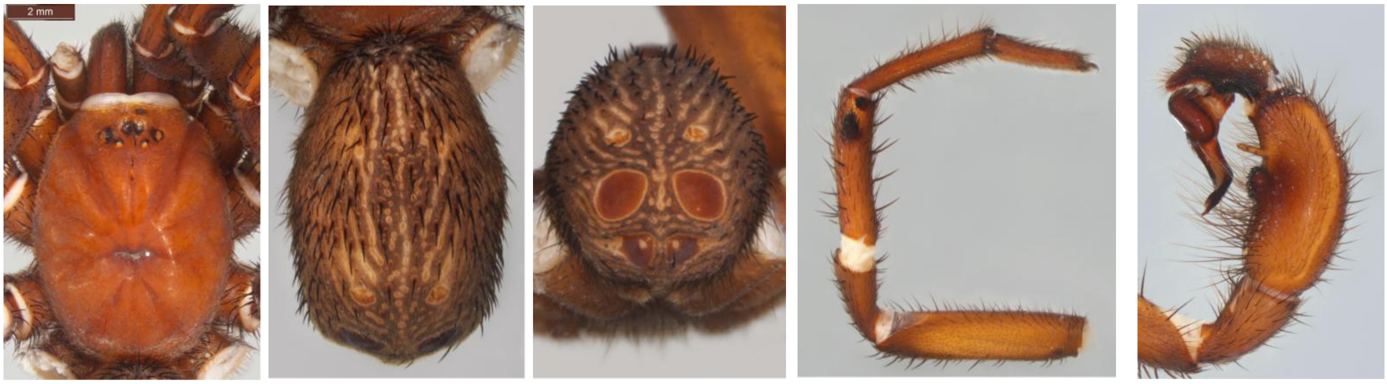

Holotype (WAM T139528) male: Lesueur National Park, WA

Idiosoma gutharuka sp. n. [MYG157]

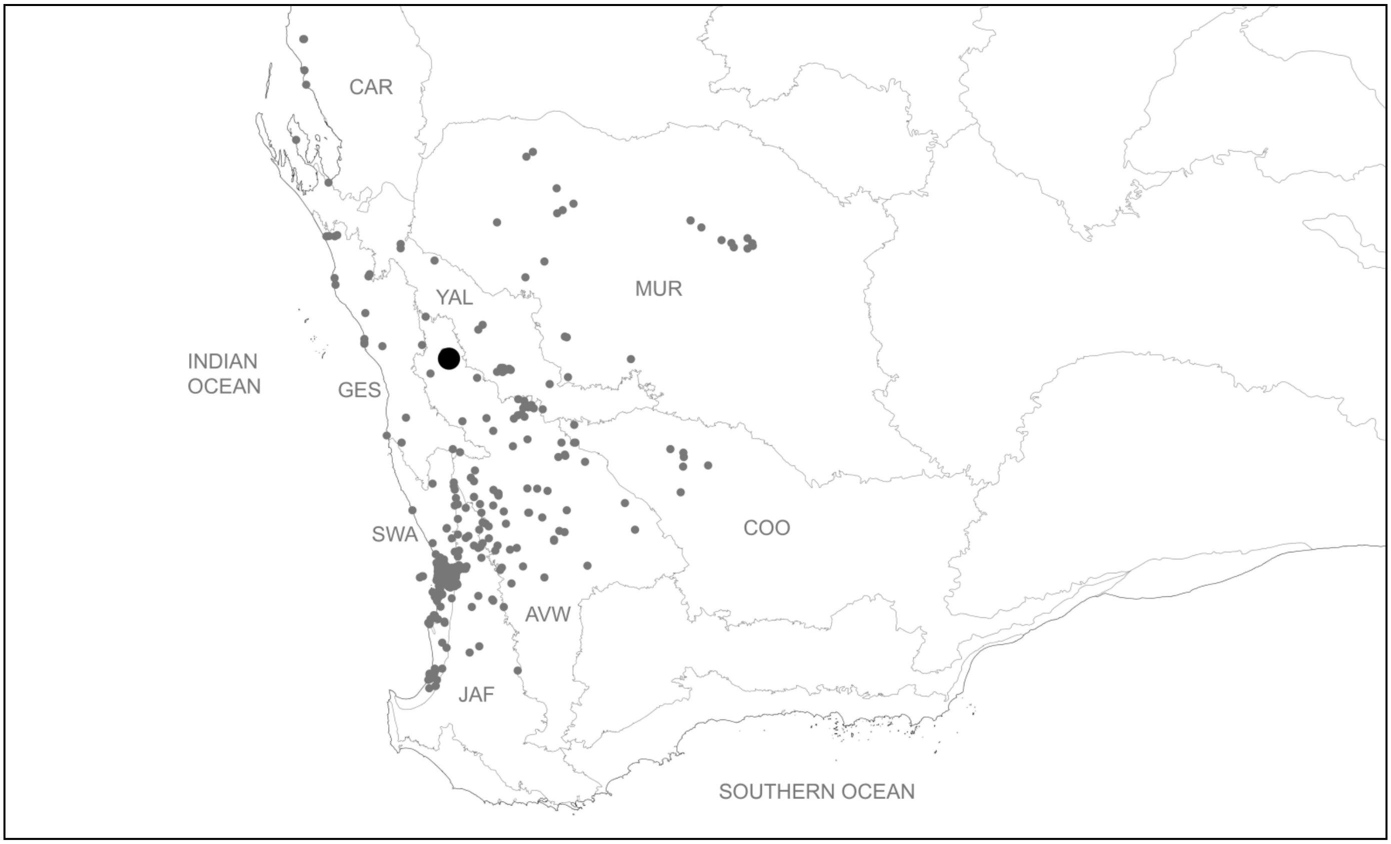

Collection records (NB. not DNA sequenced)

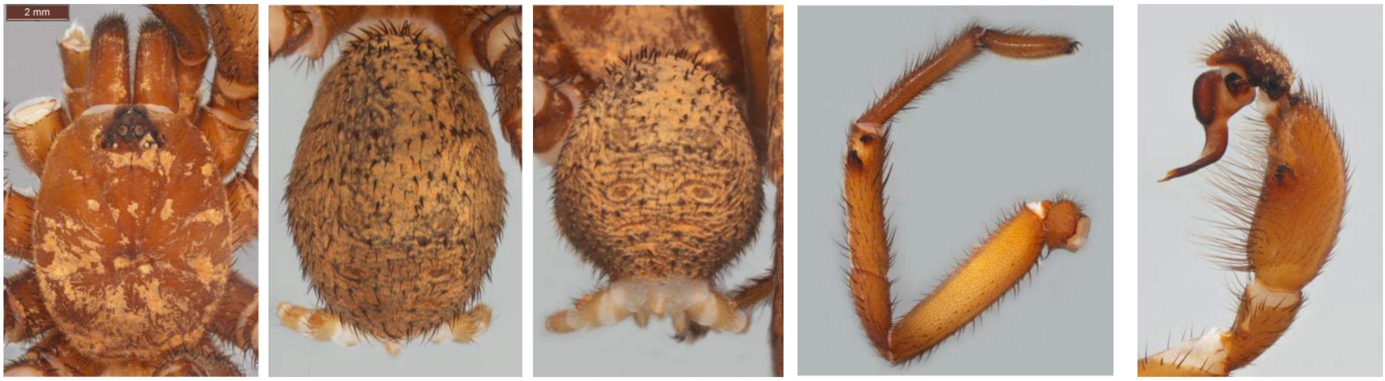

Holotype (WAM T38517) male: Gutha, WA

Idiosoma incomptum sp. n. [MYG130]

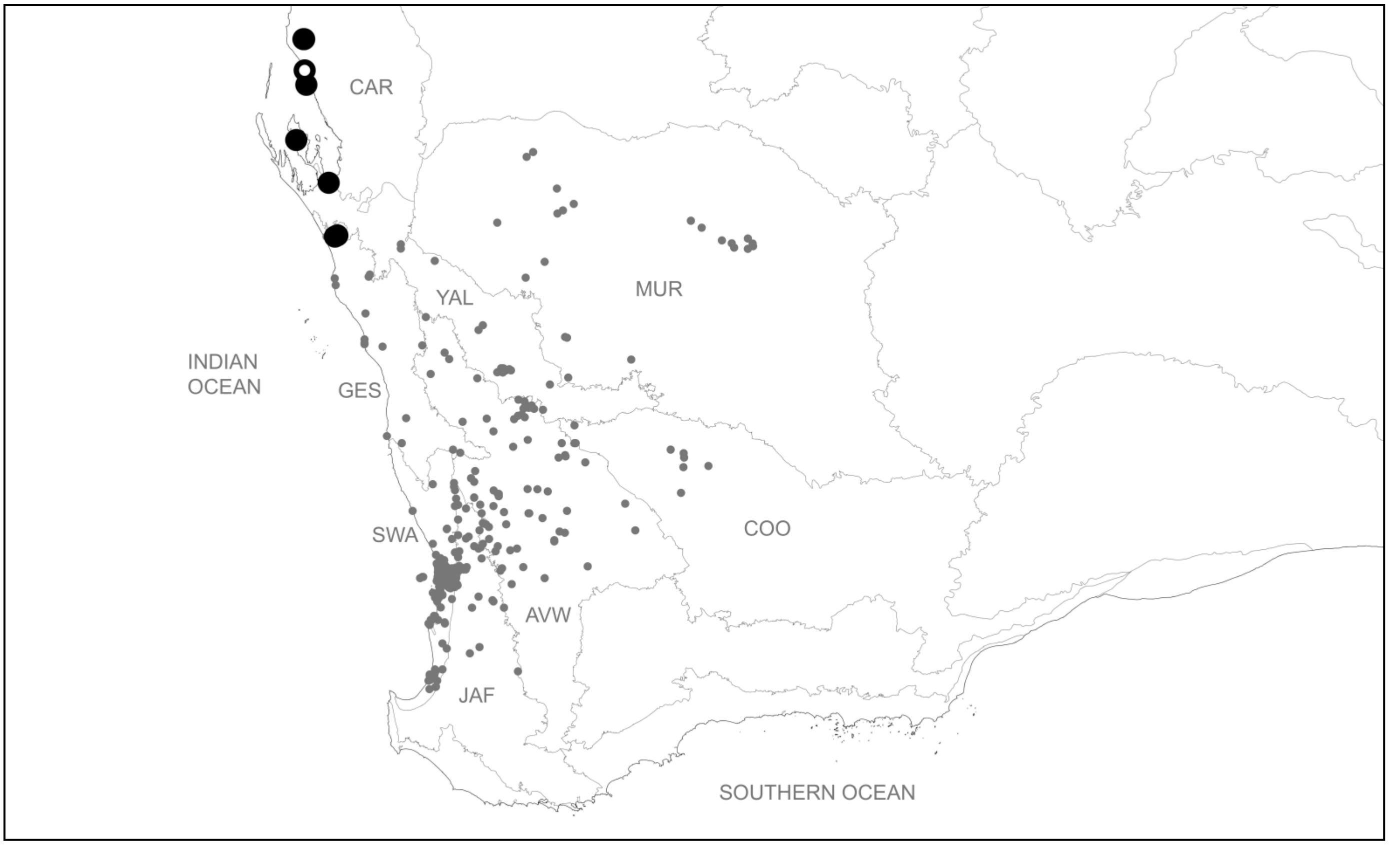

Collection records (open circles = DNA)

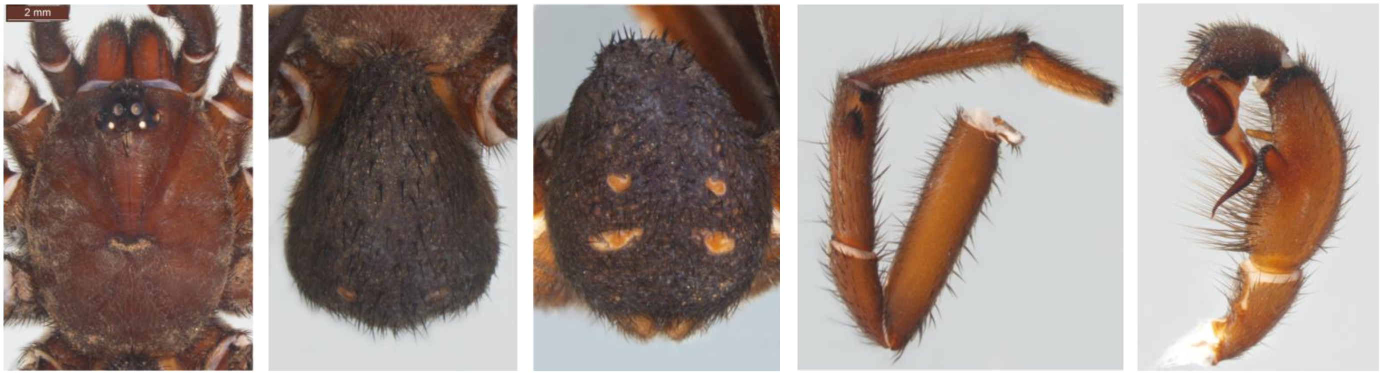

Holotype (WAM T99997) male[DNA]: Carnarvon, WA

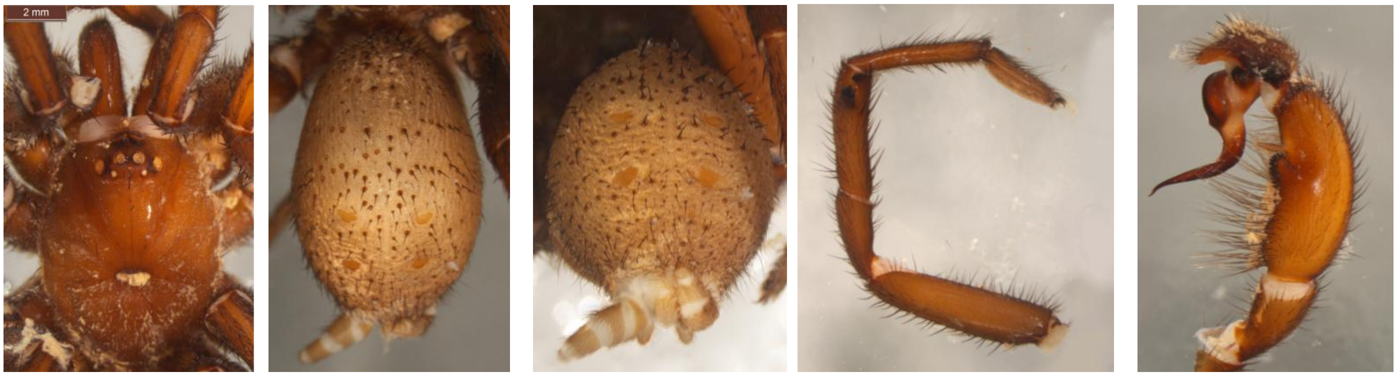

WAM T98469 male: Boolathana Station, WA

Idiosoma incomptum sp. n. [MYG130] (cont.)

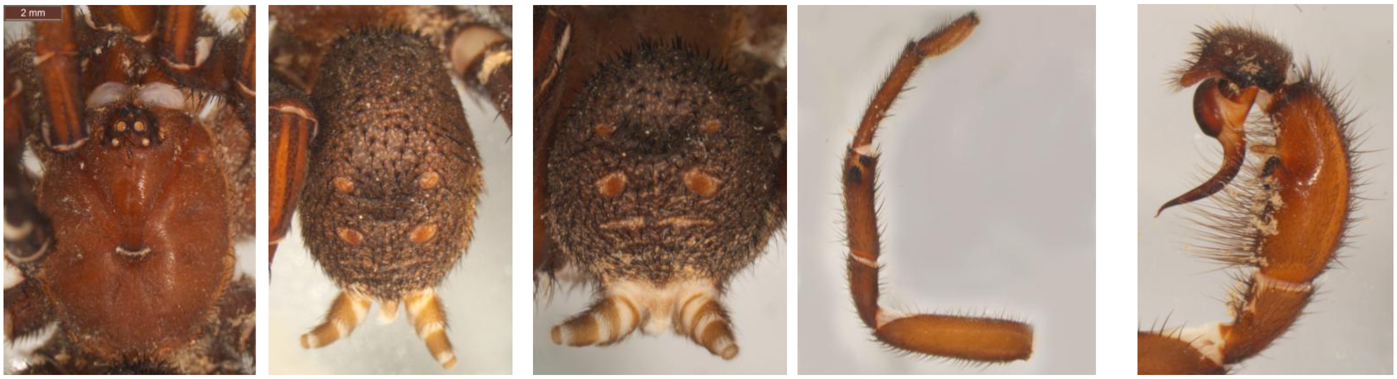

WAM T98474 male: Boolathana Station, WA

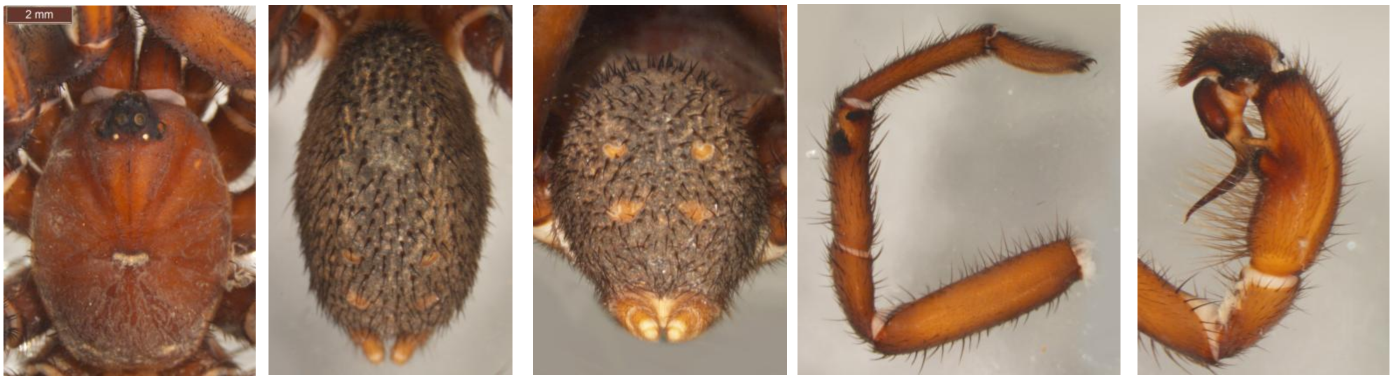

WAM T98472 male: Bush Bay, WA

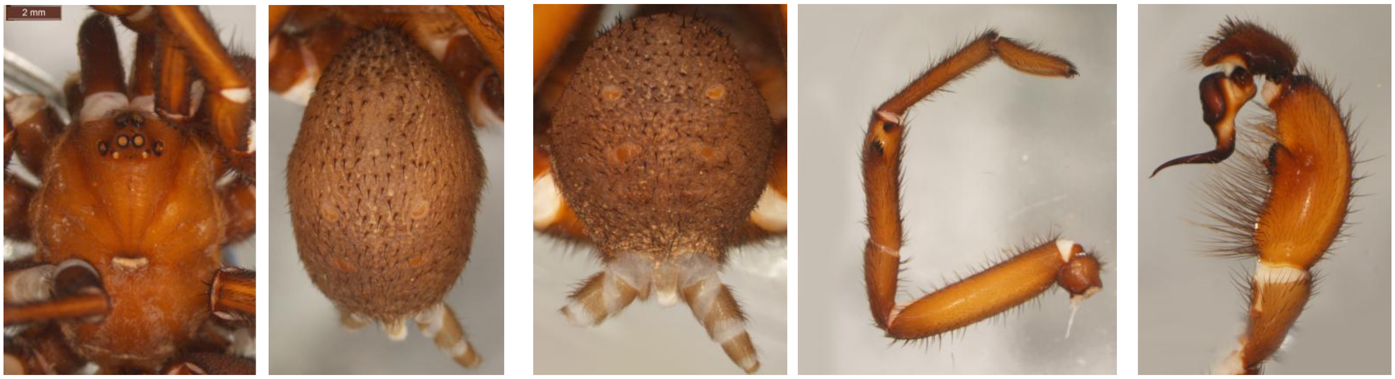

WAM T98473a male: Francois Peron National Park, WA

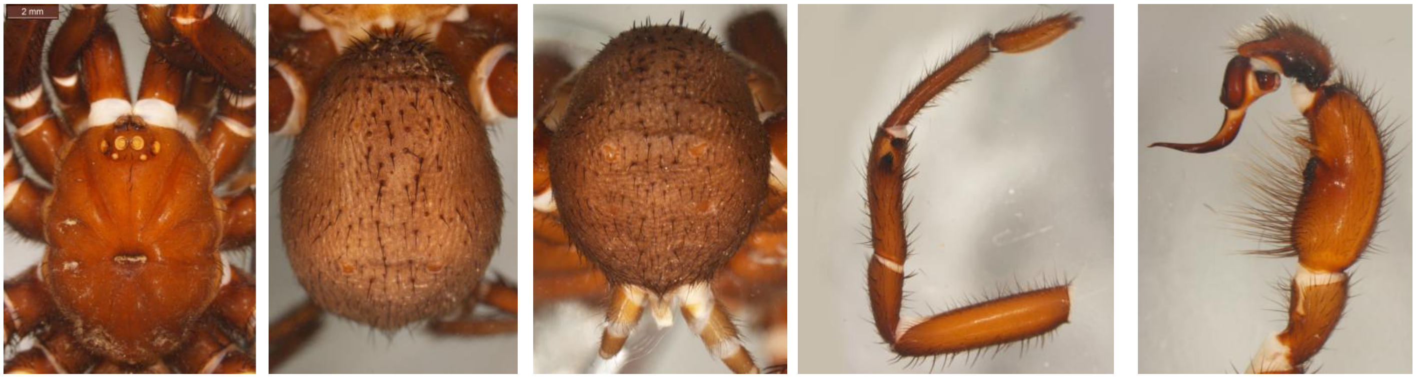

WAM T98473b male: Francois Peron National Park, WA

Idiosoma incomptum sp. n. [MYG130] (cont.)

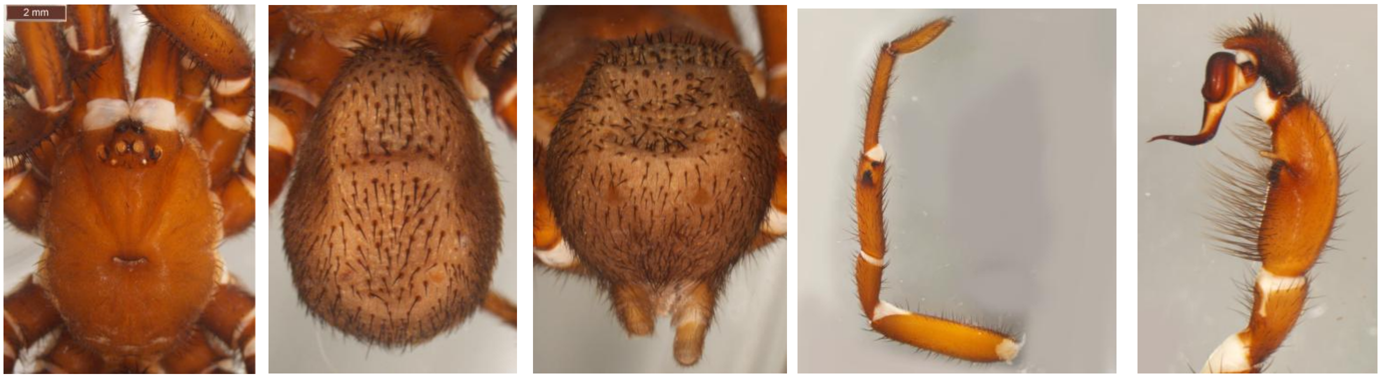

WAM T98470 male: Nanga Station, WA

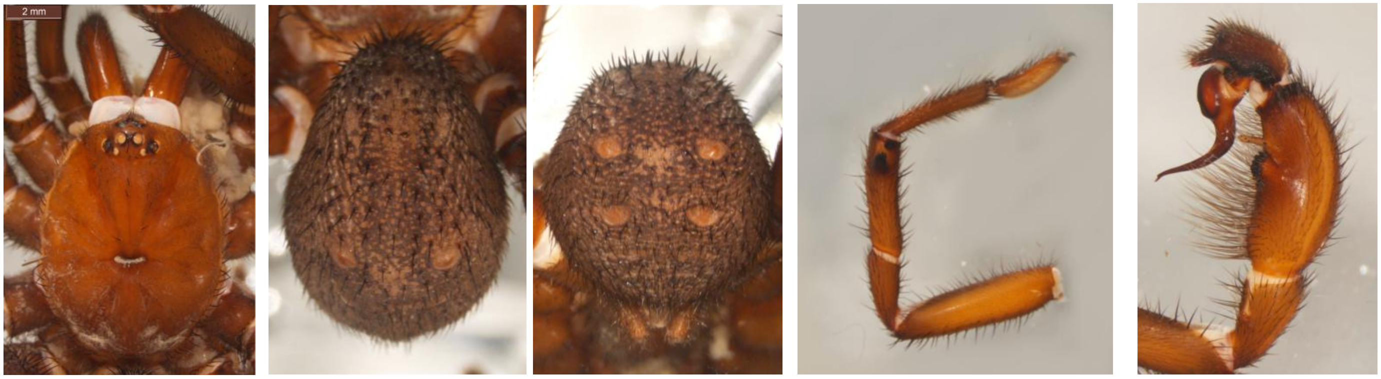

WAM T98471 male: Zuytdorp, WA

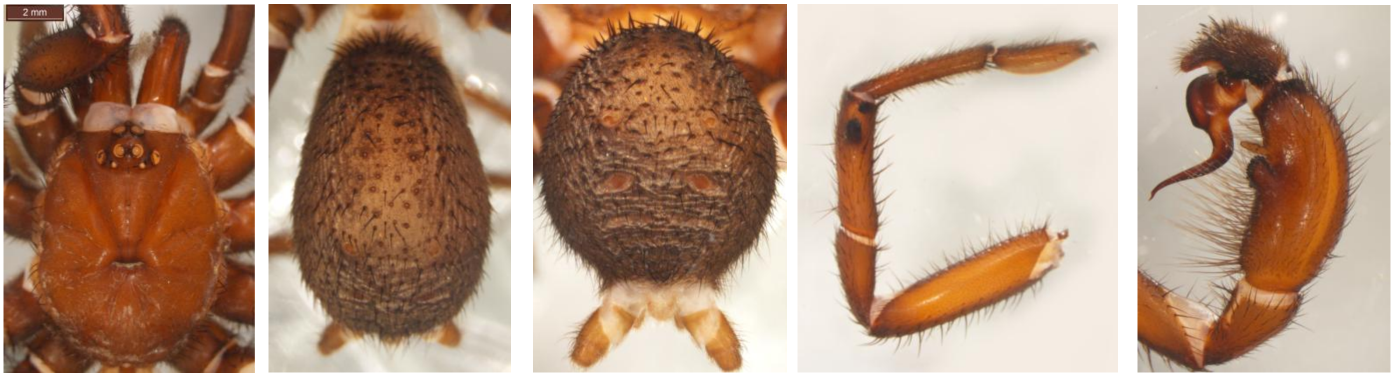

WAM T98475a male: Zuytdorp, WA

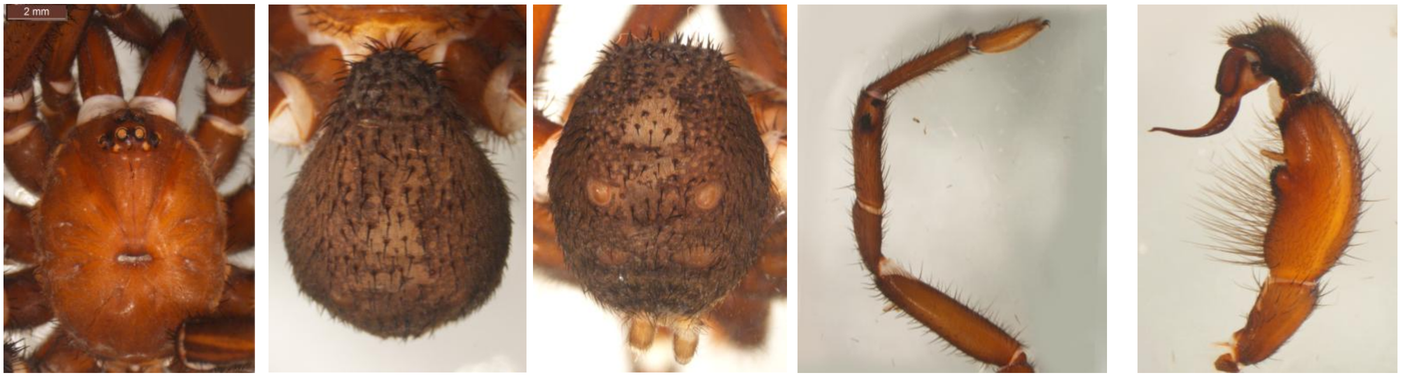

WAM T98475b male: Zuytdorp, WA

Idiosoma intermedium sp. n. [MYG475]

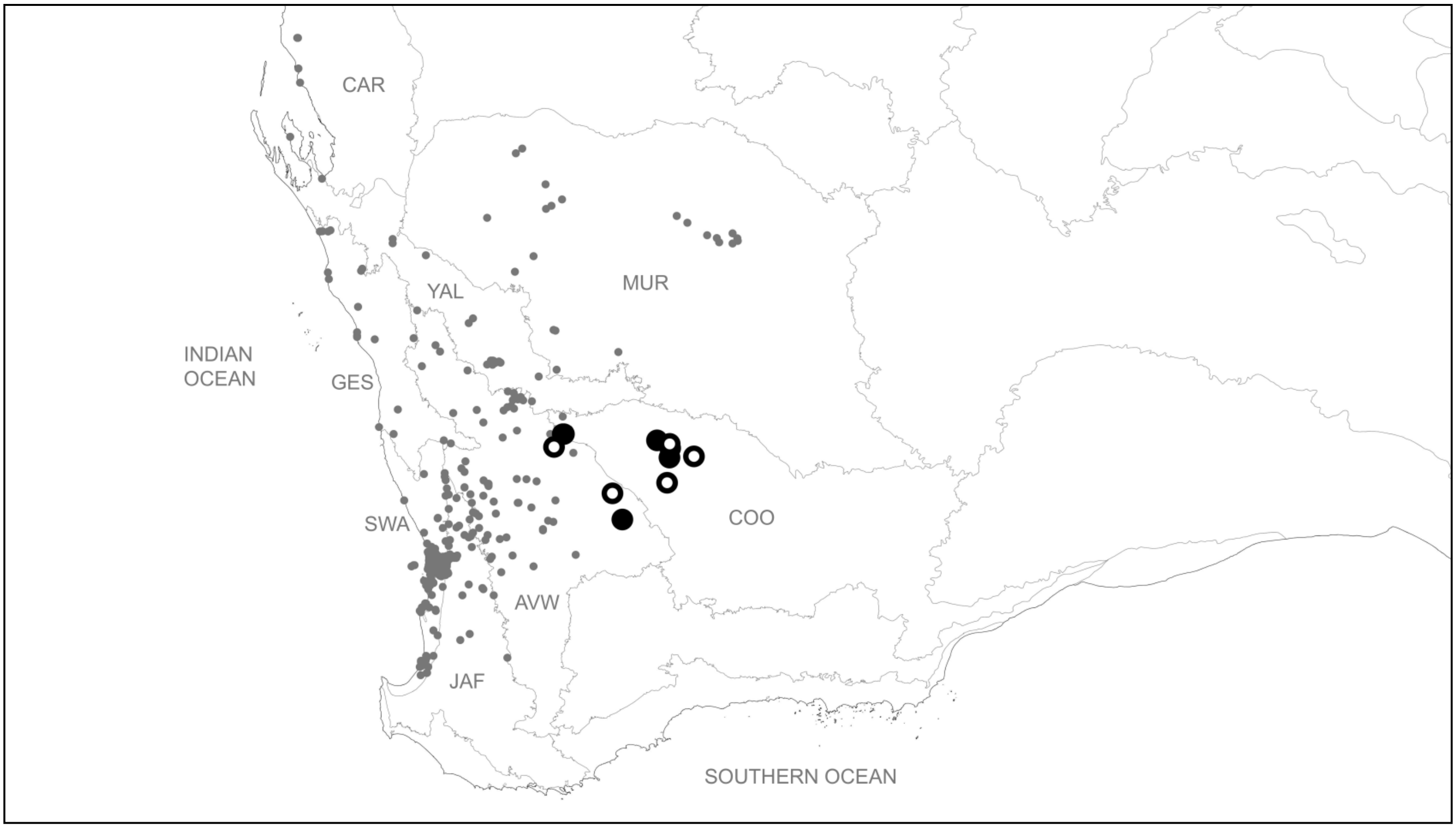

Collection records (open circles = DNA)

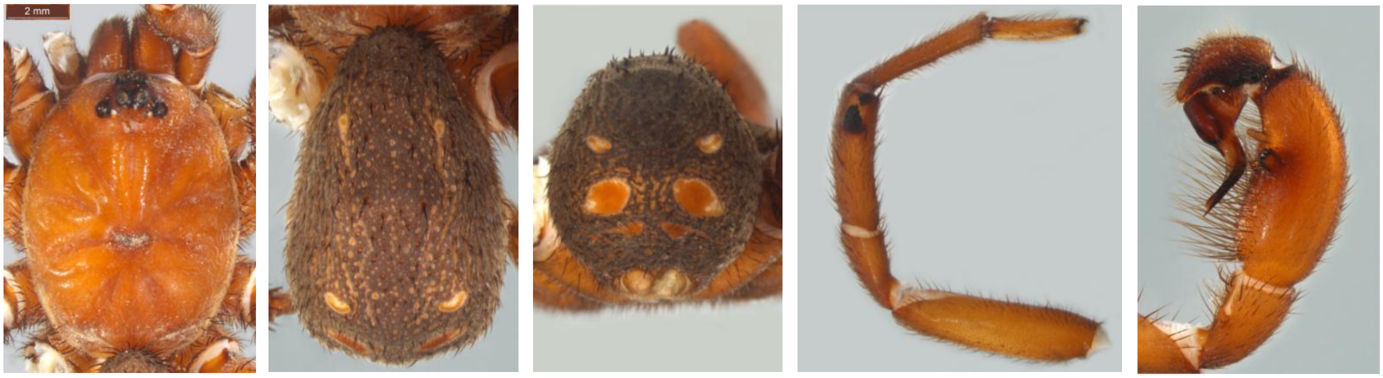

Holotype (WAM T139520) male: Bodallin, WA

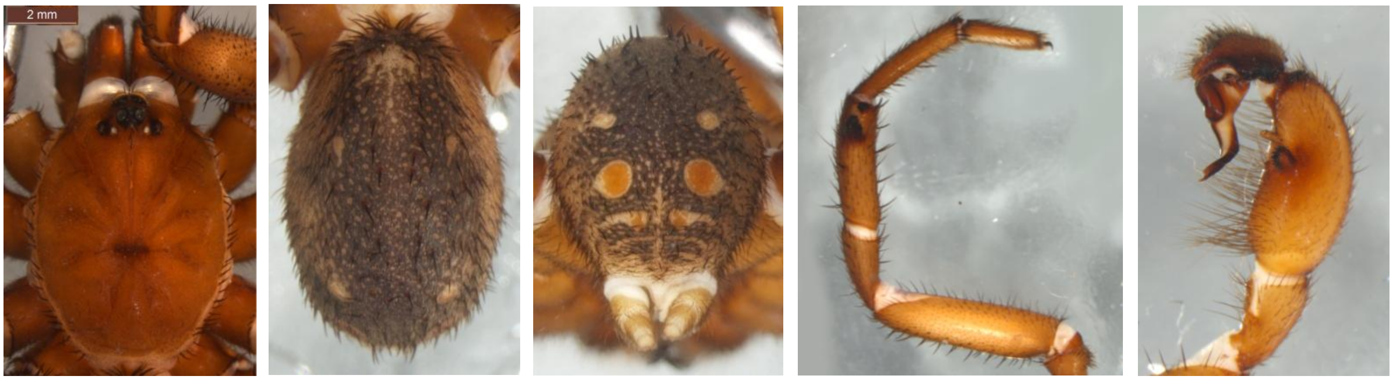

WAM T139494 male: Billiburning Rock, WA

Idiosoma intermedium sp. n. [MYG475] (cont.)

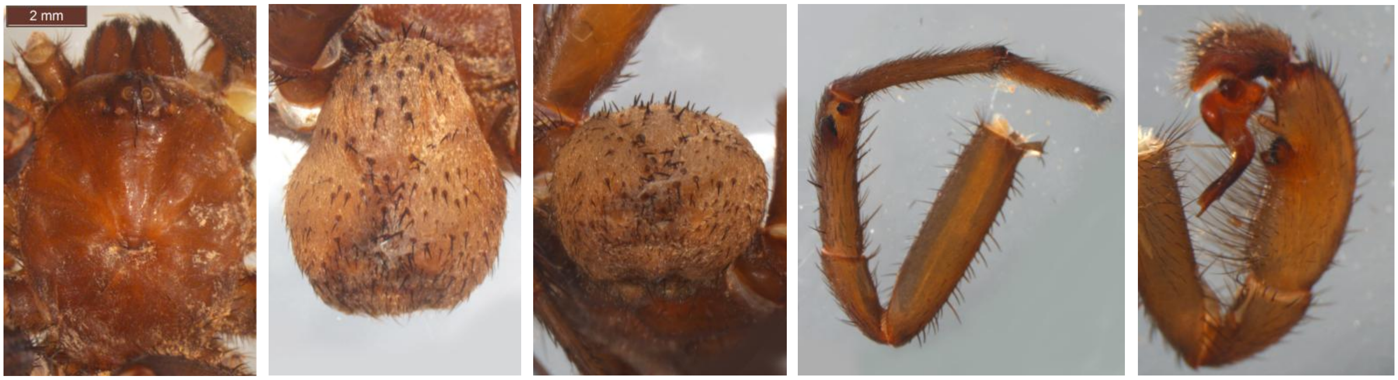

WAM T139517 male[DNA]: Mungarri Nature Reserve (South), WA

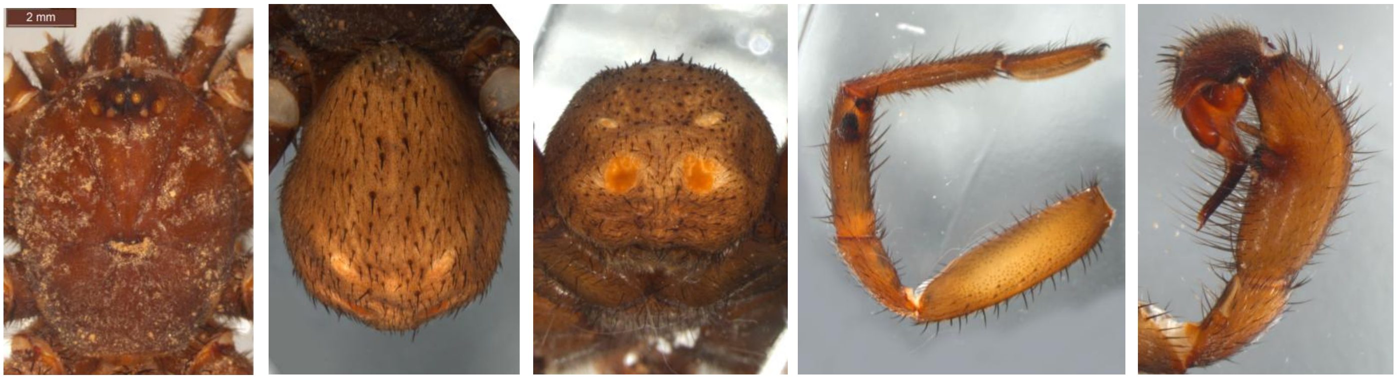

WAM T139519 male[DNA]: Warrachuppin North Road, WA

Idiosoma jarrah sp. n. [MYG156]

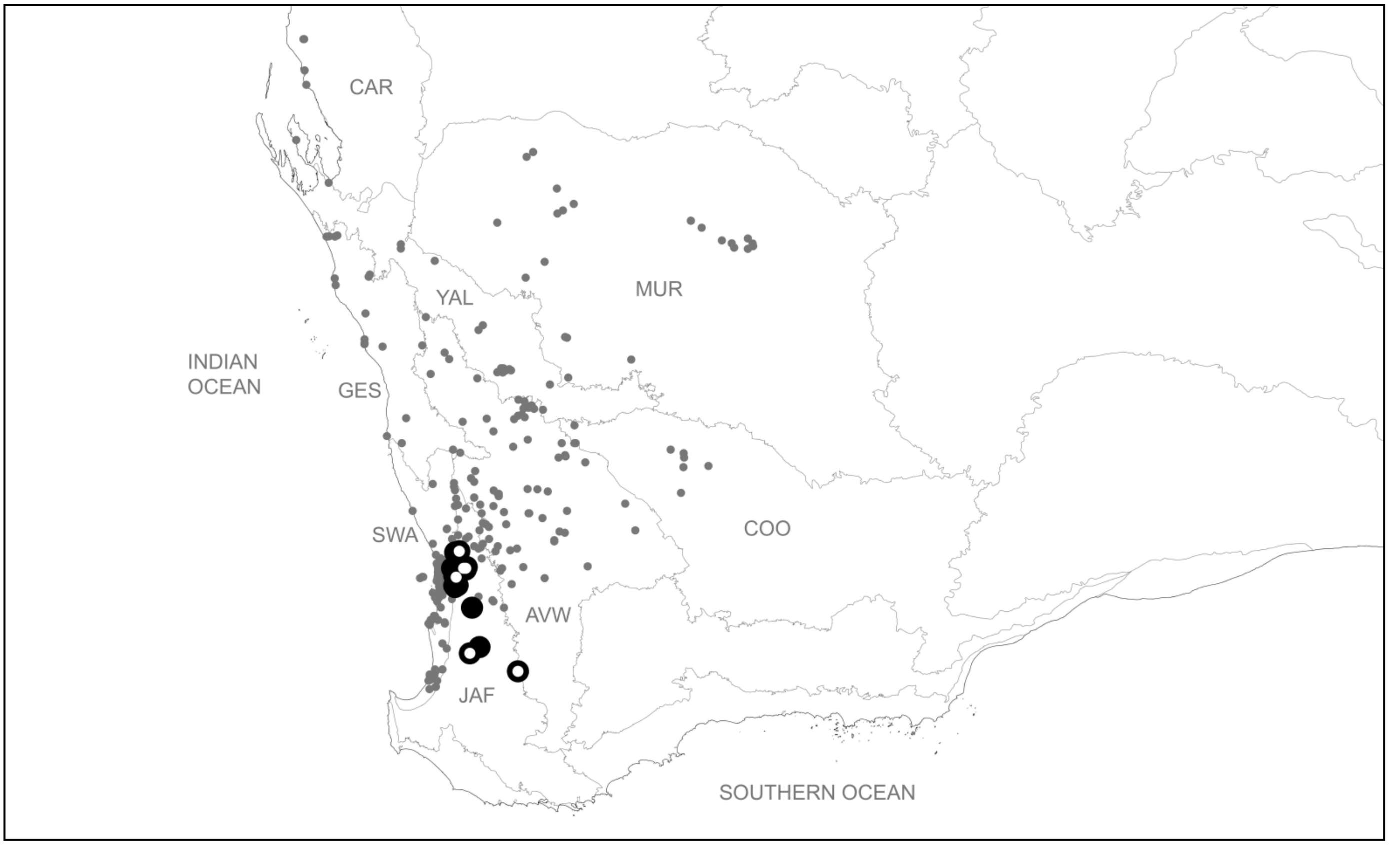

Collection records (open circles = DNA)

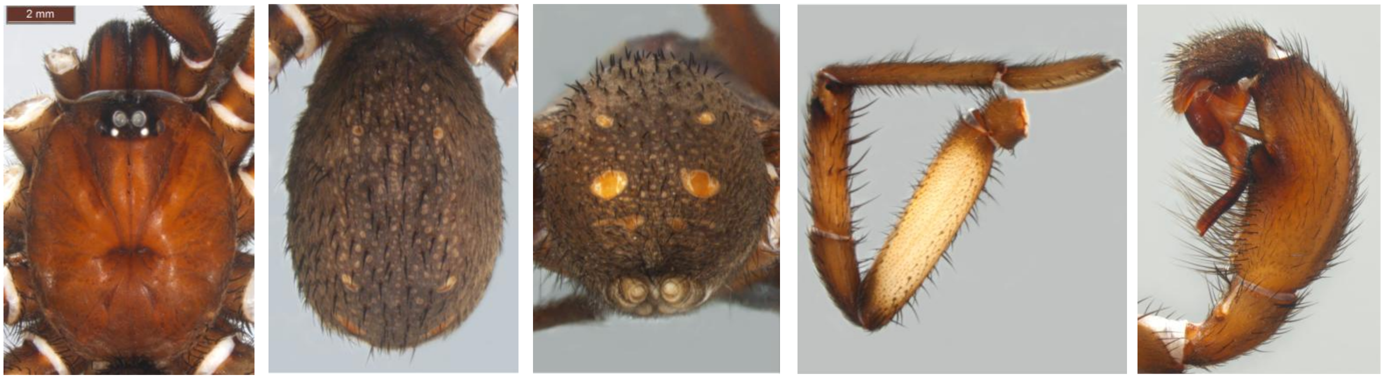

Holotype (WAM T124143) male$^{\underline{DNA}}$: Lesmurdie, WA

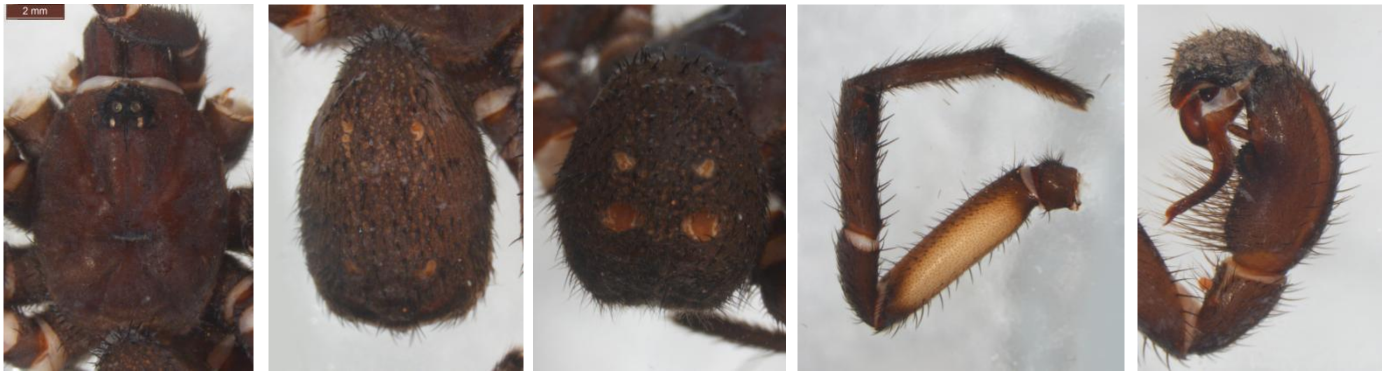

WAM T74623 male: SW. of Boddington, WA

Idiosoma jarrah sp. n. [MYG156] (cont.)

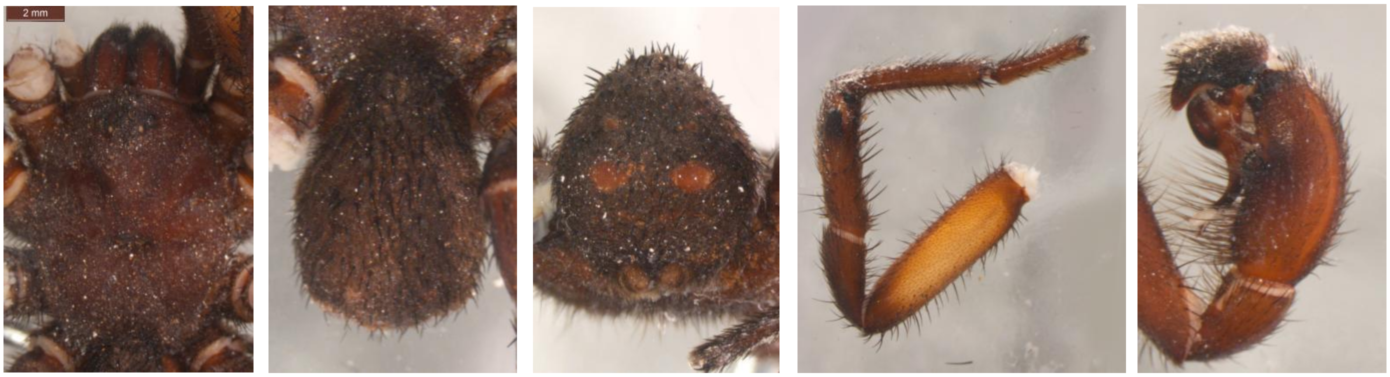

WAM T99952 male[DNA]: SW. of Boddington, WA

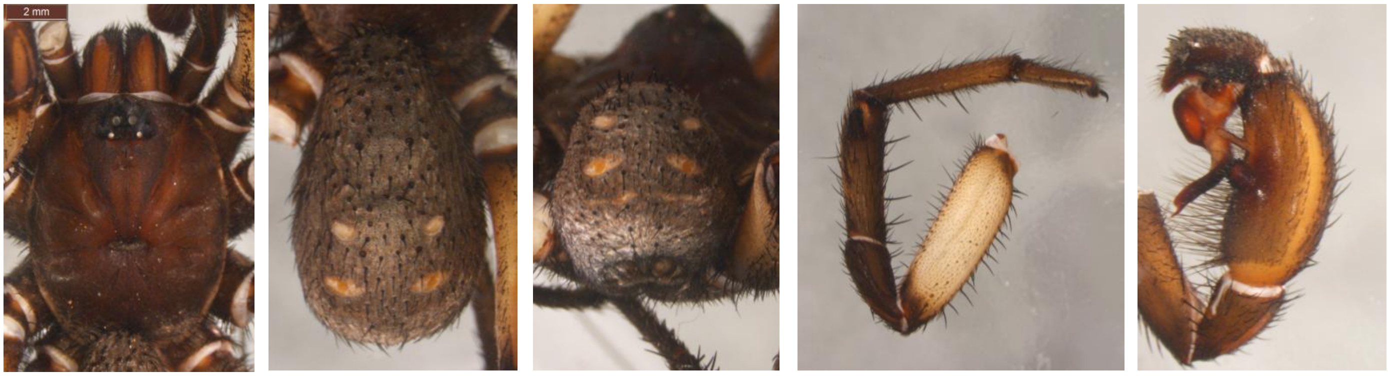

WAM T136943 male[DNA]: Bullsbrook, Smith Road, WA

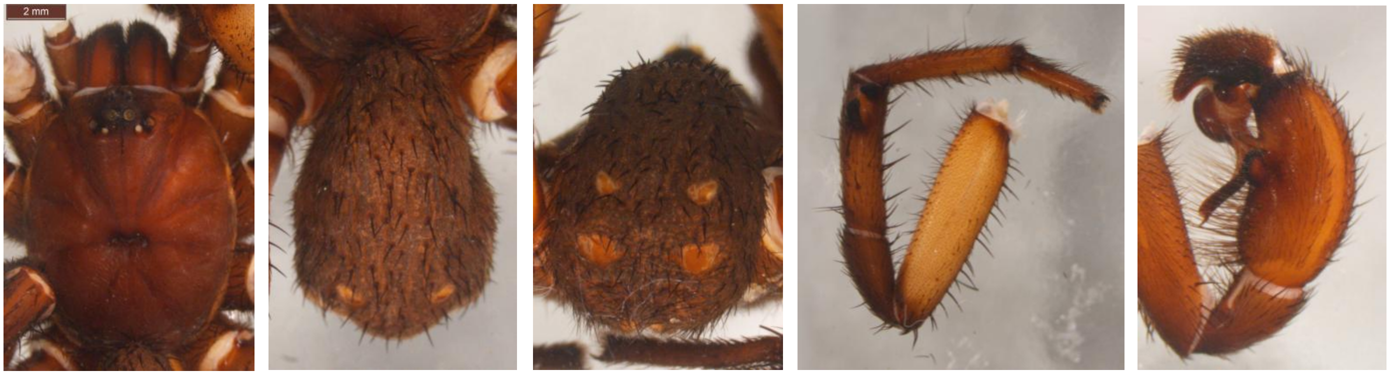

WAM T26822 male: Darlington, WA

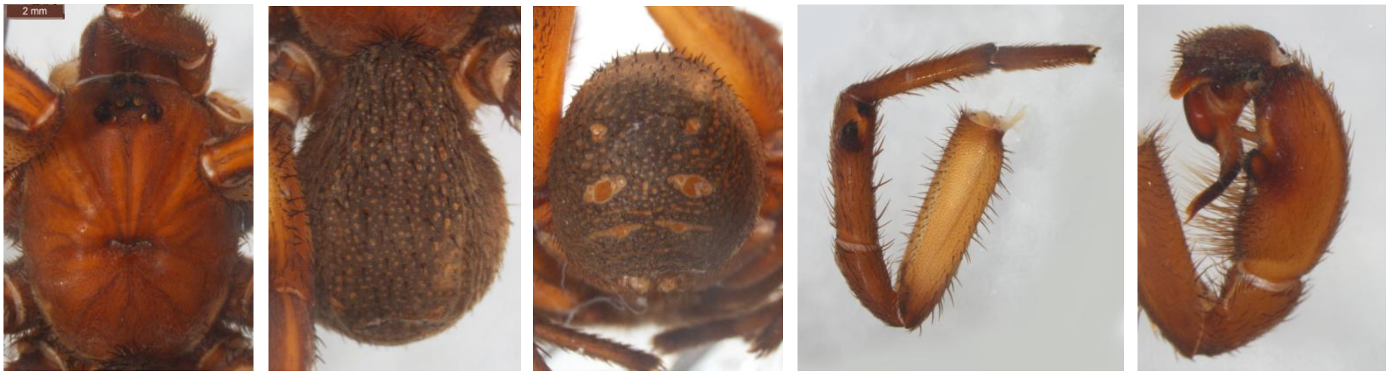

WAM T139475 male: Gooseberry Hill, WA

Idiosoma jarrah sp. n. [MYG156] (cont.)

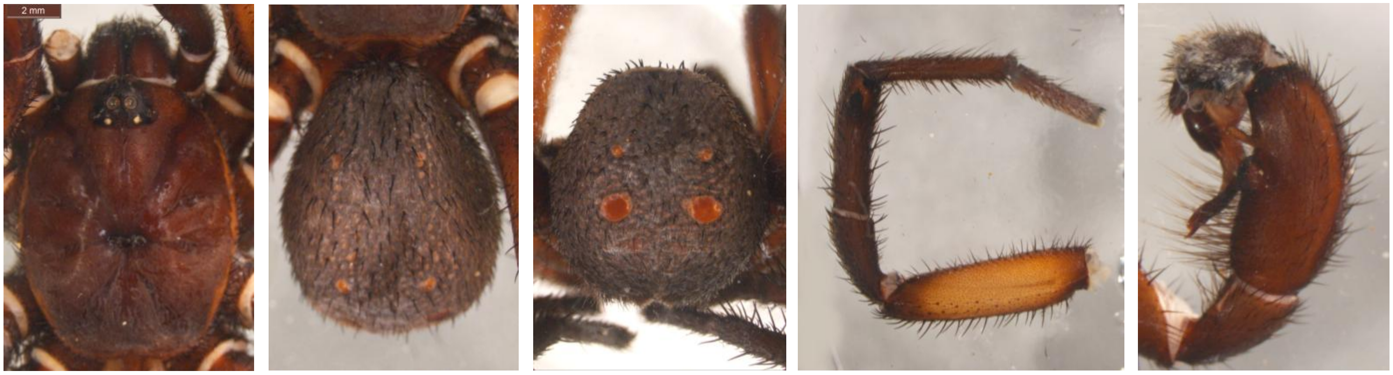

WAM T63354 male: Mount Helena, WA

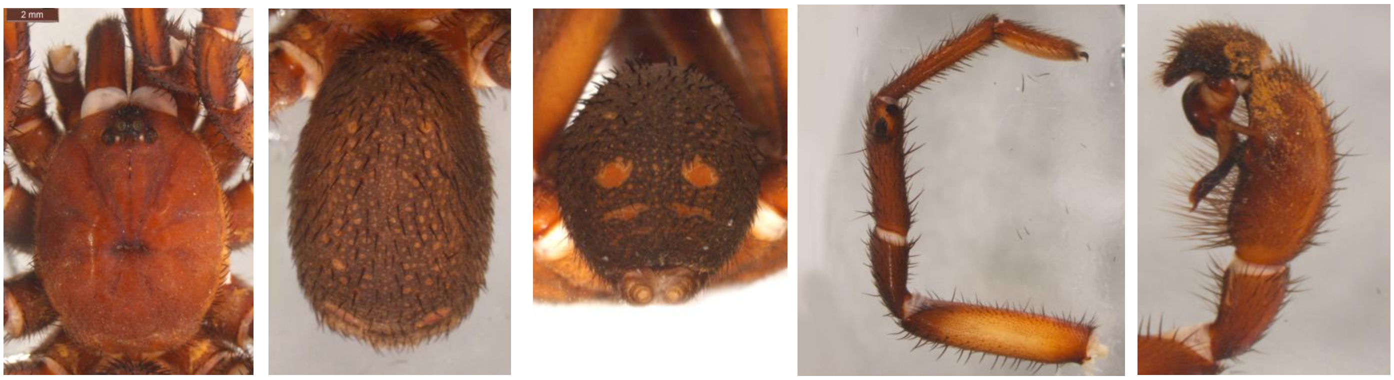

WAM T18582 male: Mundaring Weir Road, Kalamunda, WA

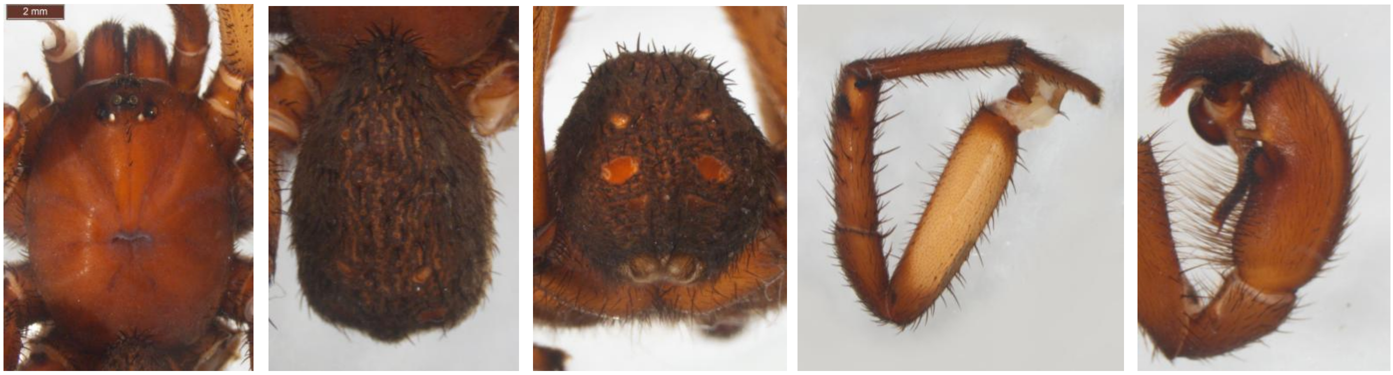

WAM T139474 male: Roleystone, WA

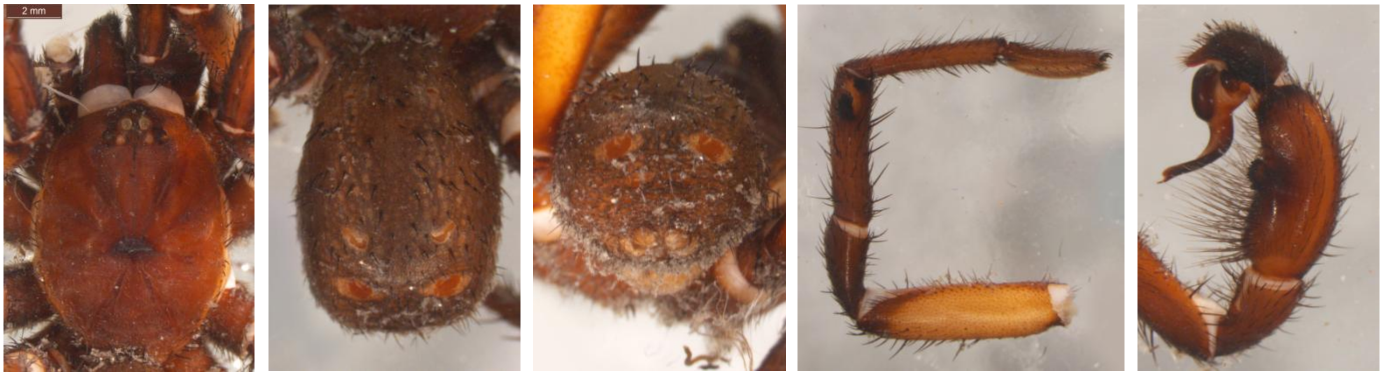

WAM T30019 male: Talbot Road Reserve, site TR3, WA

Idiosoma kopejtkaorum sp. n. [MYG521]

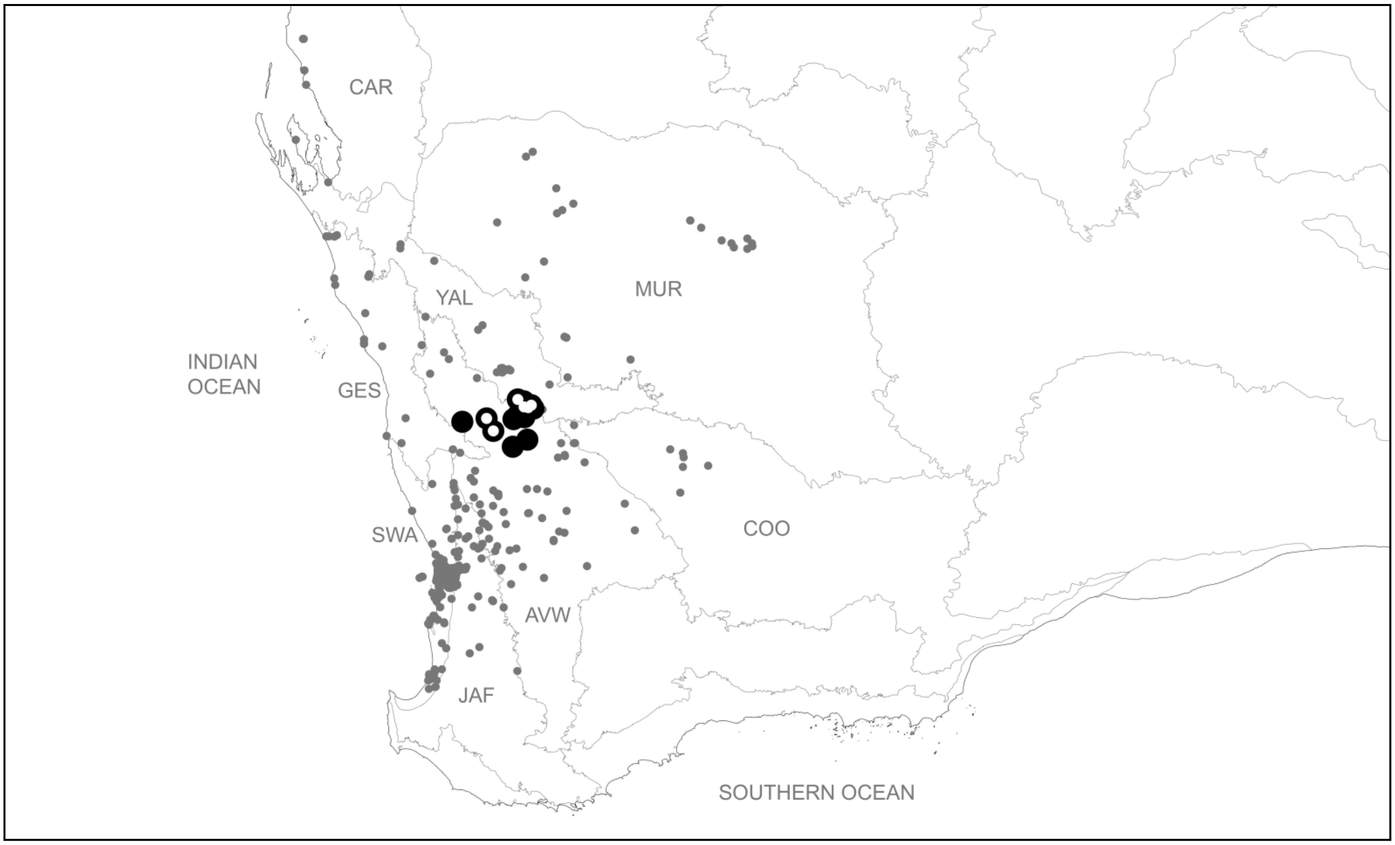

Collection records (open circles = DNA)

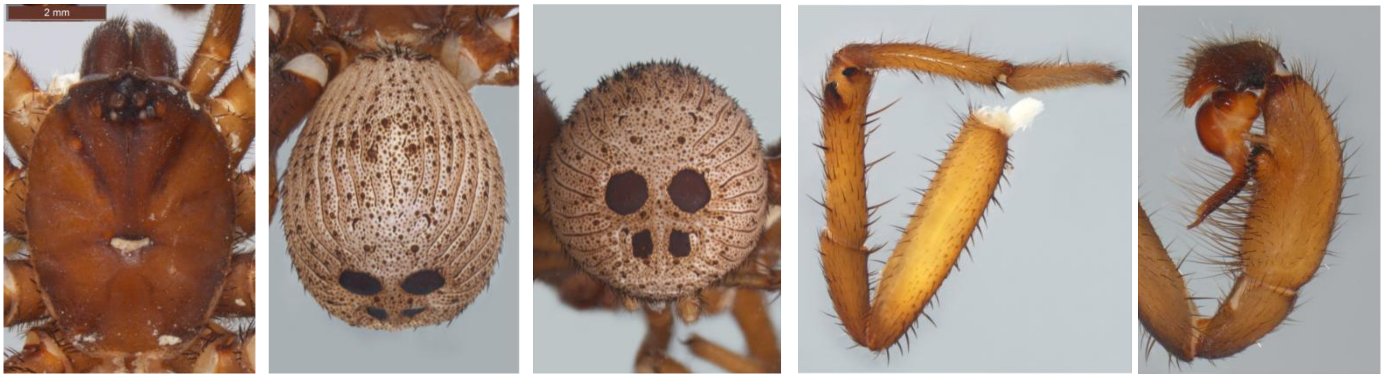

Holotype (WAM T144621) male: Snake Gully Nature Reserve, WA

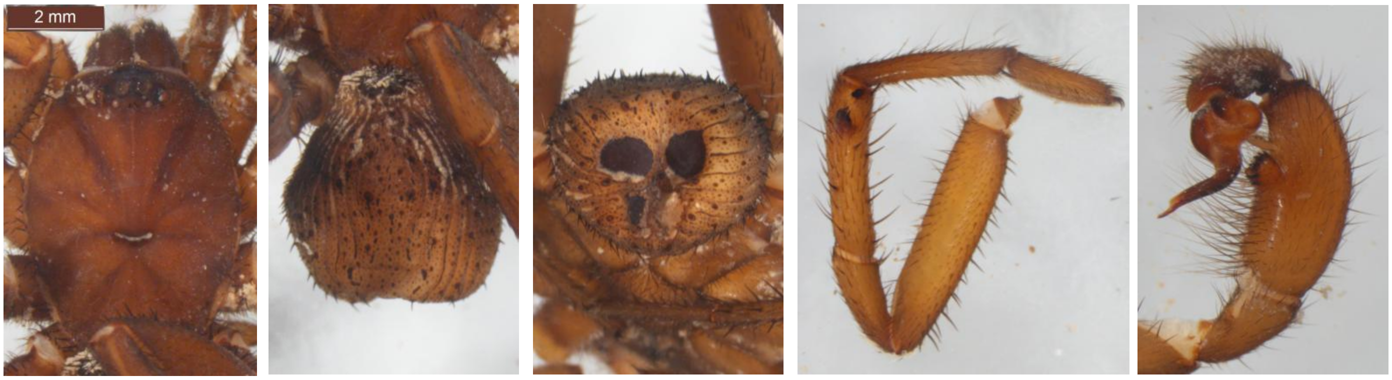

Paratype (WAM T139498) male: Snake Gully Nature Reserve, WA

Idiosoma kopejtkaorum sp. n. [MYG521] (cont.)

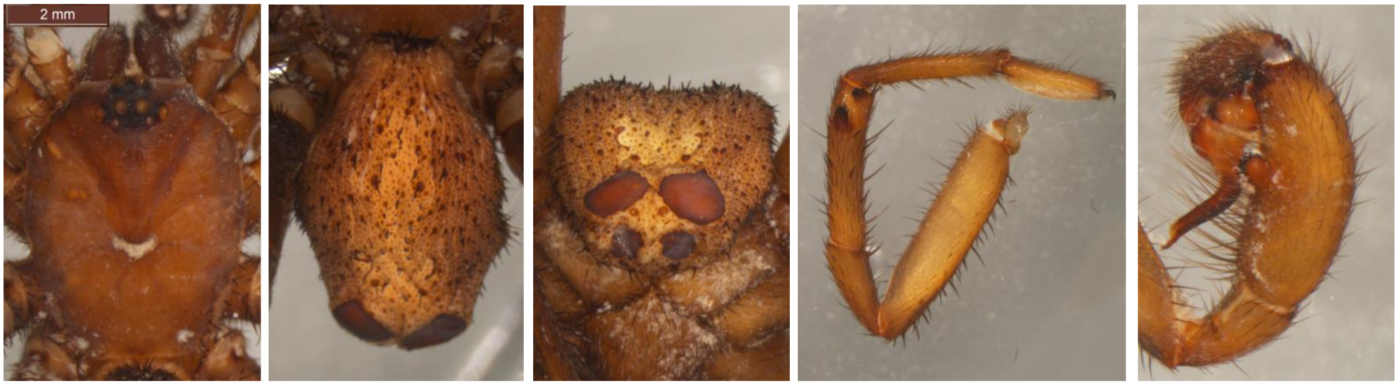

WAM T139499 male: Snake Gully Nature Reserve, WA

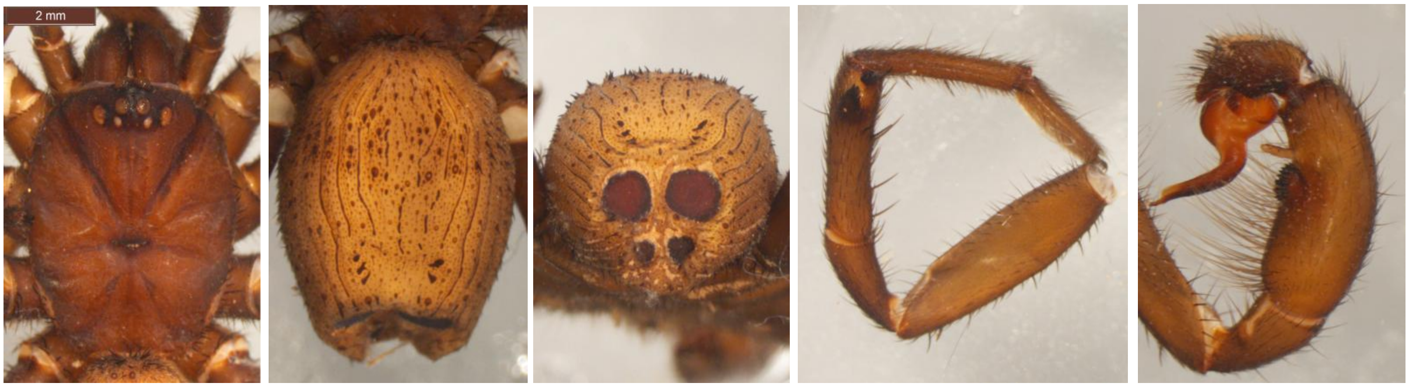

WAM T139497 male: Lake Goorly (North-west), WA

Idiosoma kwongan sp. n. [MYG472]

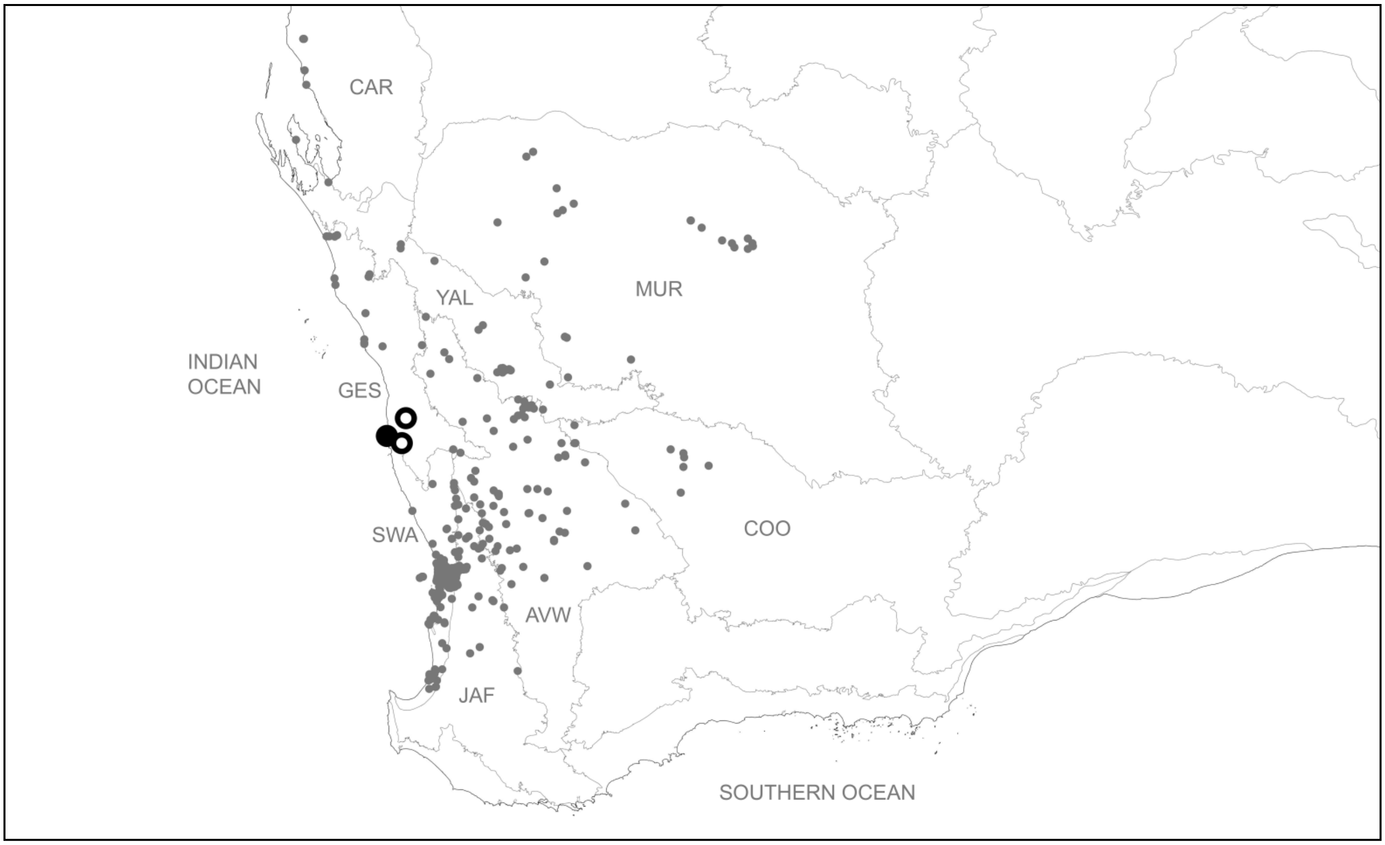

Collection records (open circles = DNA)

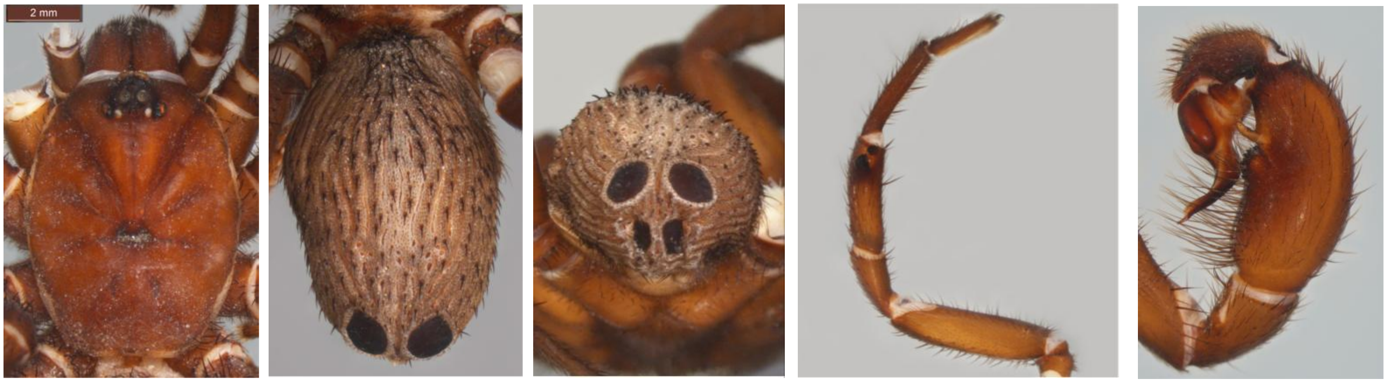

Holotype (WAM T27142) male: E. of Greenhead, WA

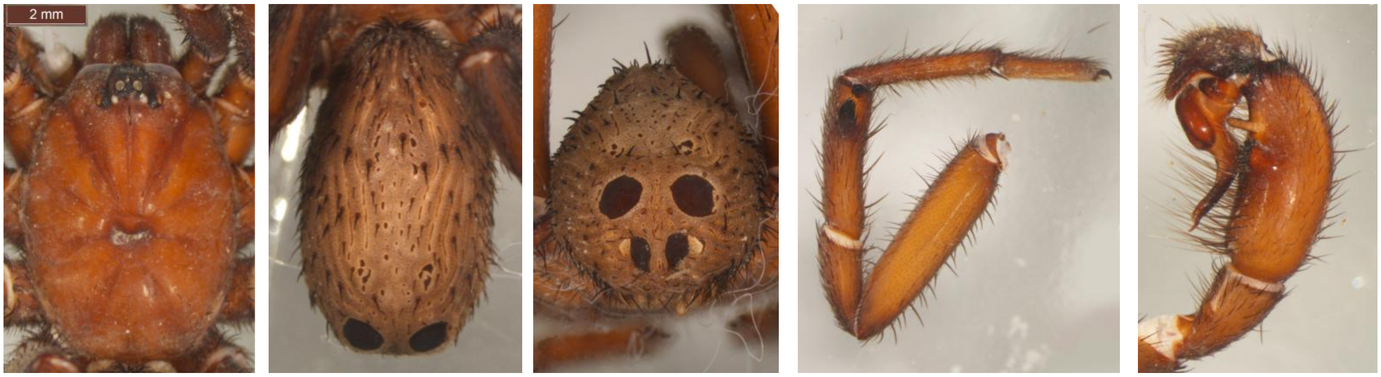

WAM T27117 male[DNA]: Eneabba, AMC Minesite, WA

Idiosoma kwongan sp. n. [MYG472] (cont.)

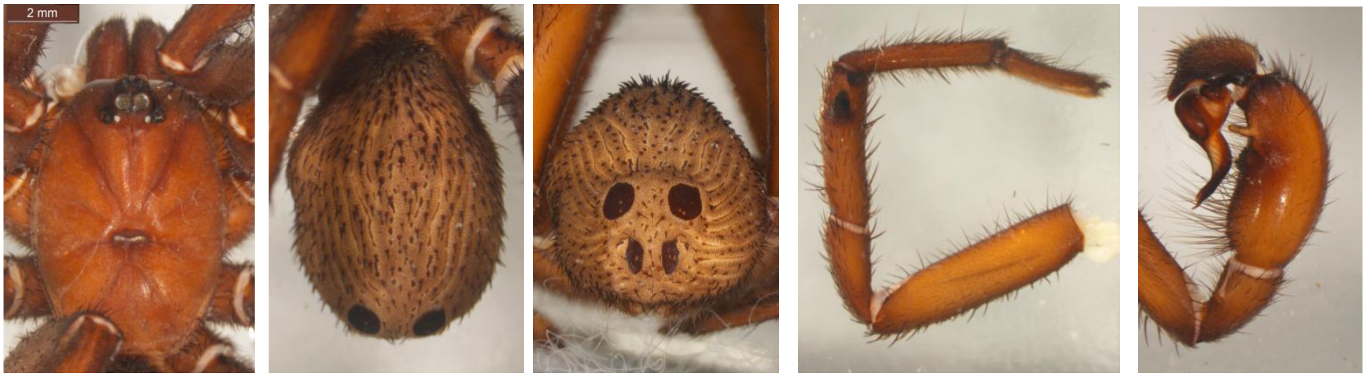

WAM T27118 male: Eneabba, AMC Minesite, WA

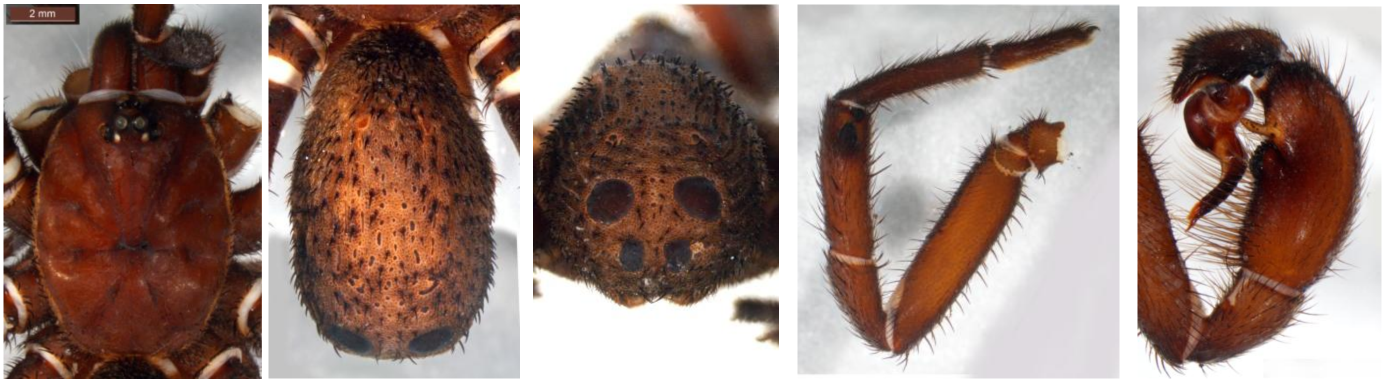

WAM T139468 male[DNA]: Lesueur National Park, WA

Idiosoma mcclementsorum sp. n. [MYG474]

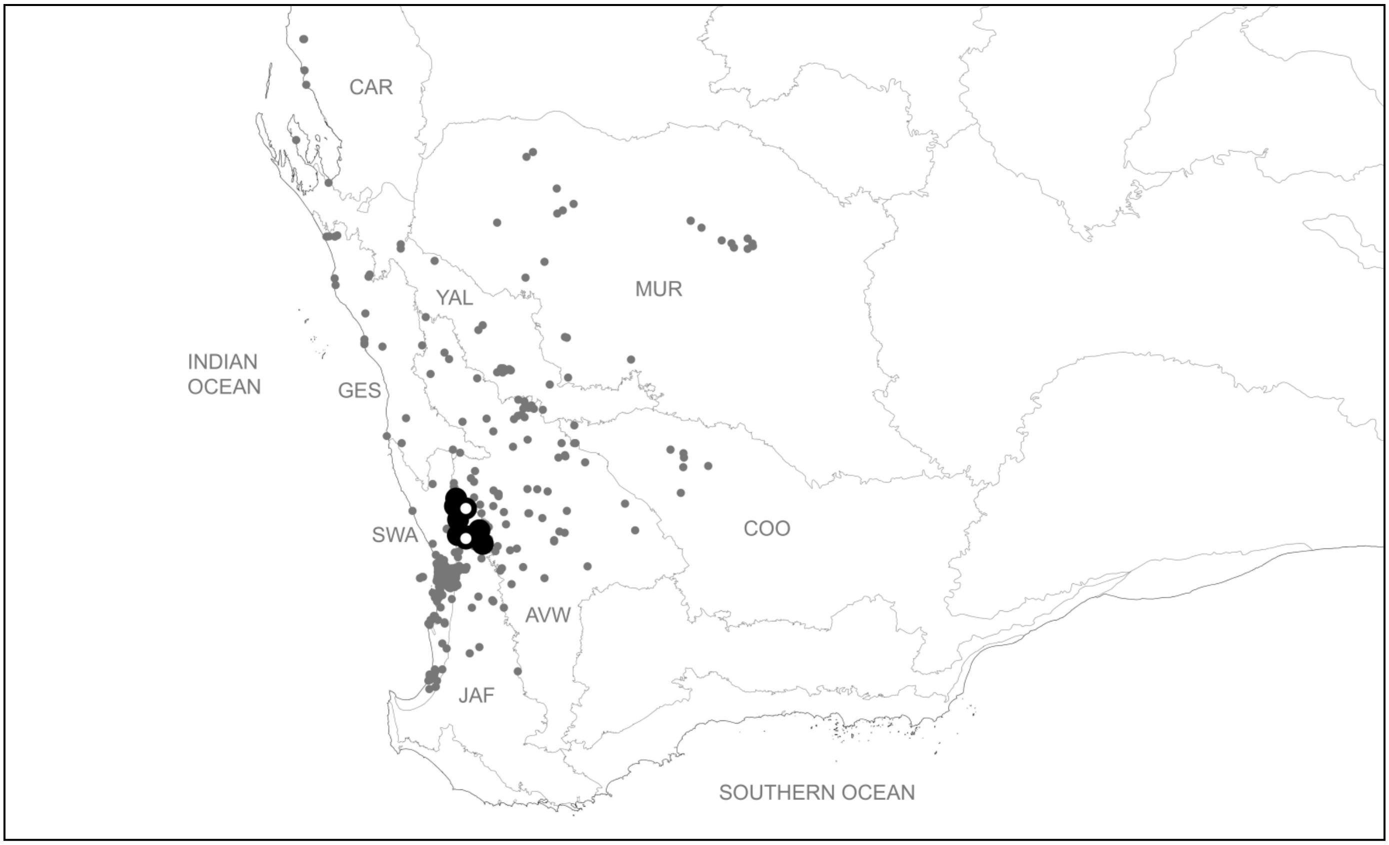

Collection records (open circles = DNA)

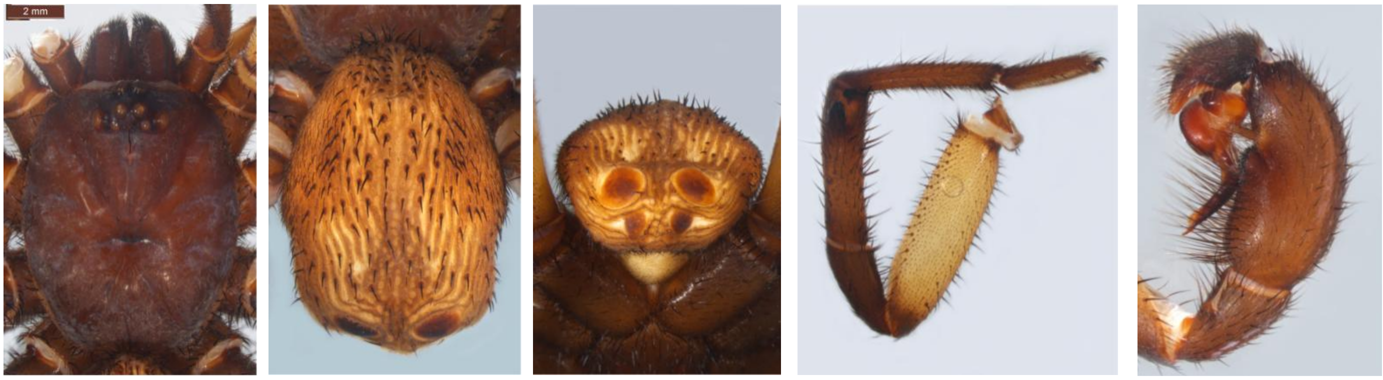

Holotype (WAM T139471) male: Julimar State Forest, WA

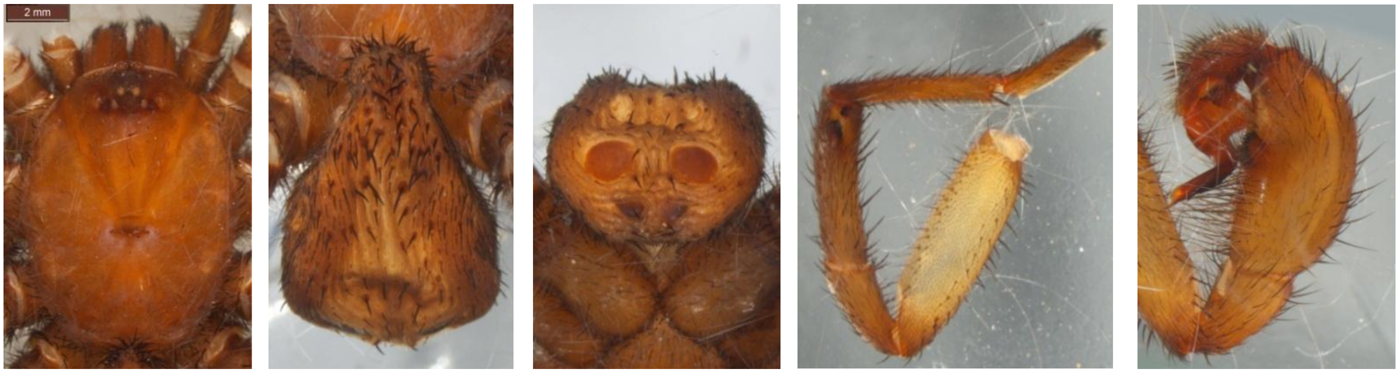

Paratype (WAM T139472) male: Julimar State Forest, WA

Idiosoma mcclementsorum sp. n. [MYG474] (cont.)

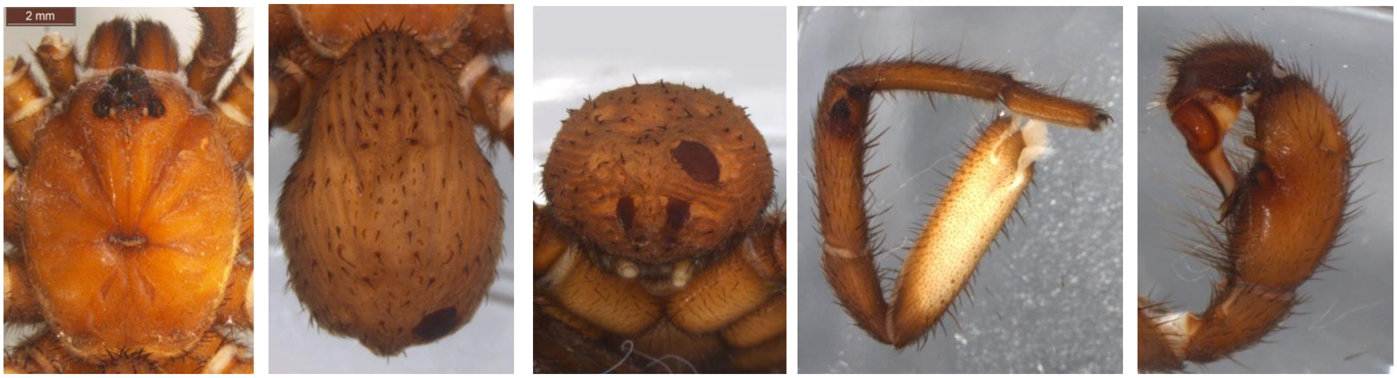

WAM T29779 male: Toodyay, Bindoon Road, WA

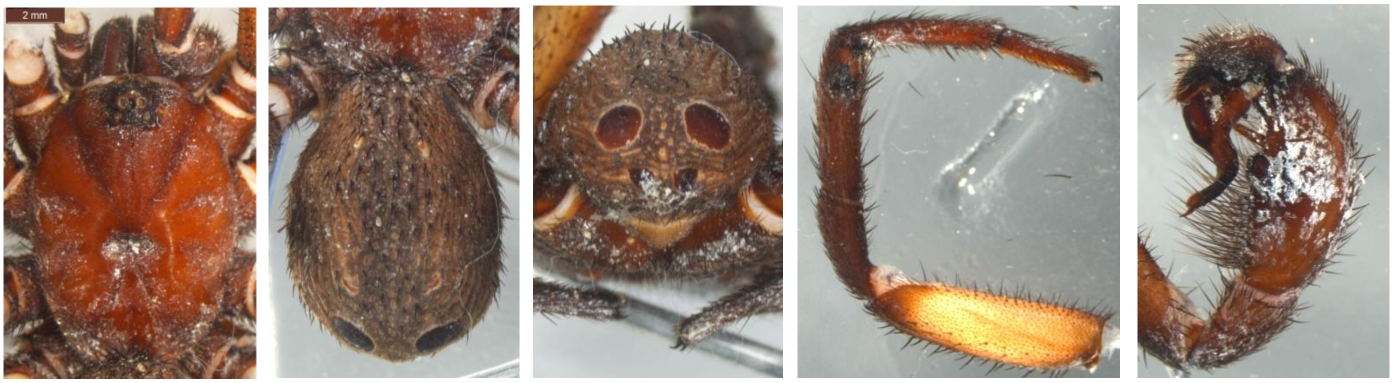

WAM T44388 male: Toodyay, WA

Idiosoma mcnamarai sp. n. [MYG520]

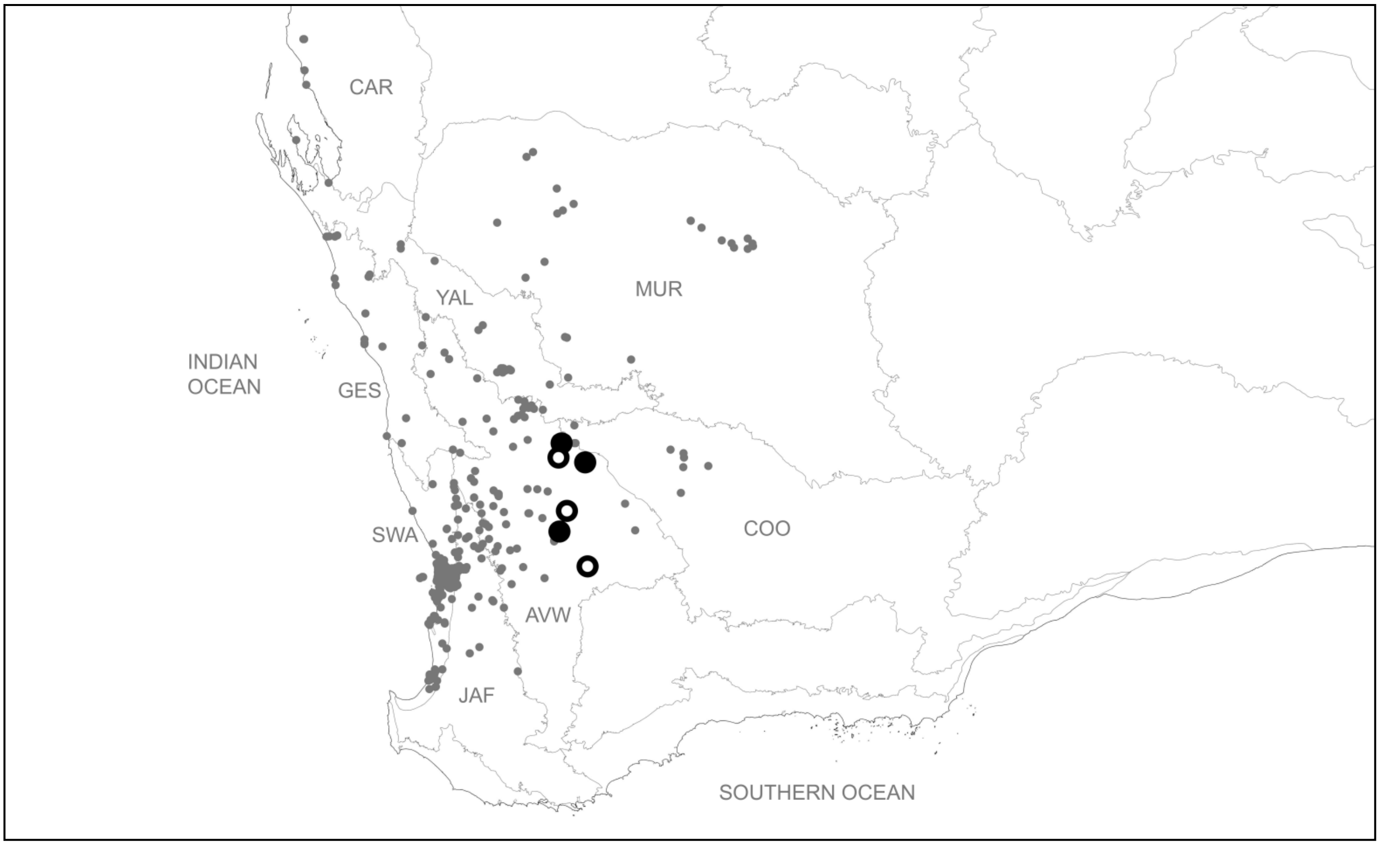

Collection records (open circles = DNA)

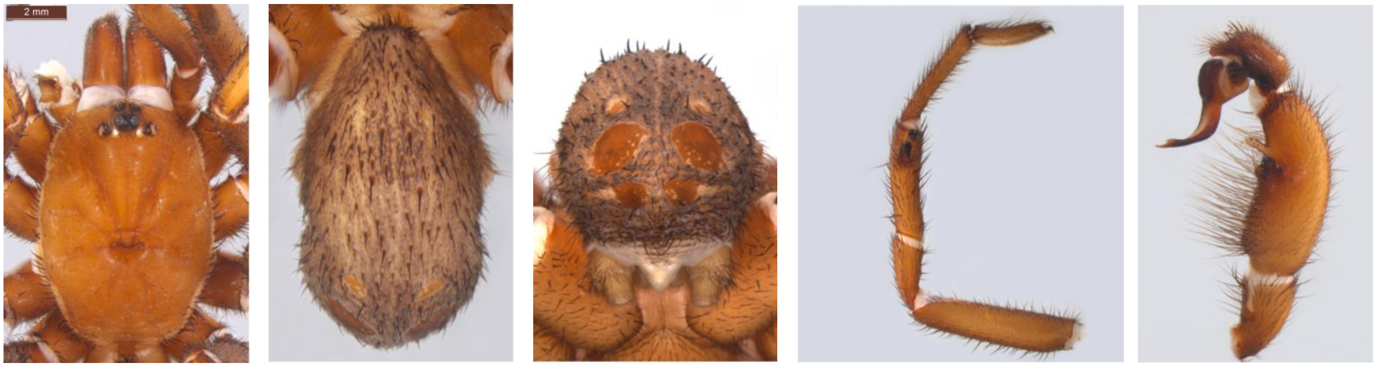

Holotype (WAM T26107) male<u>DNA</u>: Trayning, WA

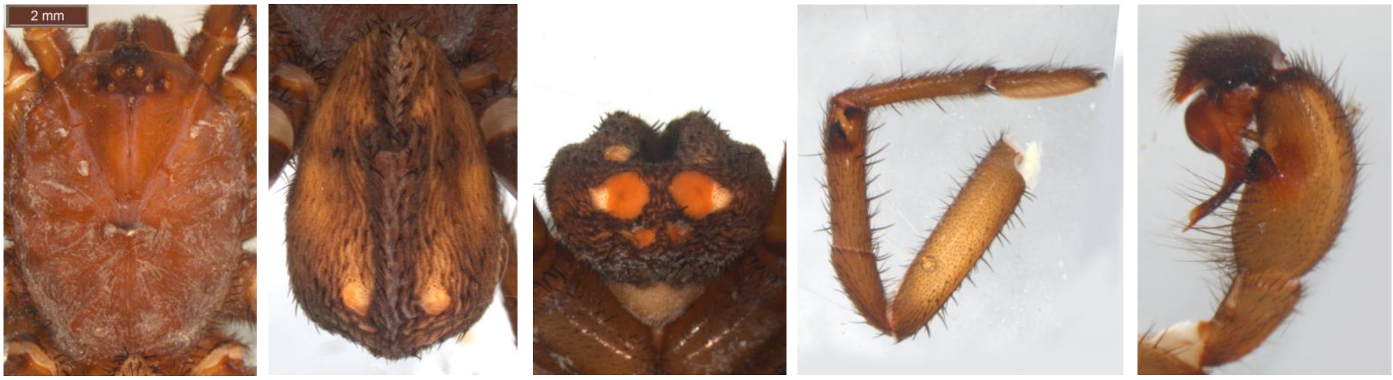

WAM T139518 male<u>DNA</u>: Bruce Rock-Doodlakine Road, WA

Idiosoma mcnamarai sp. n. [MYG520] (cont.)

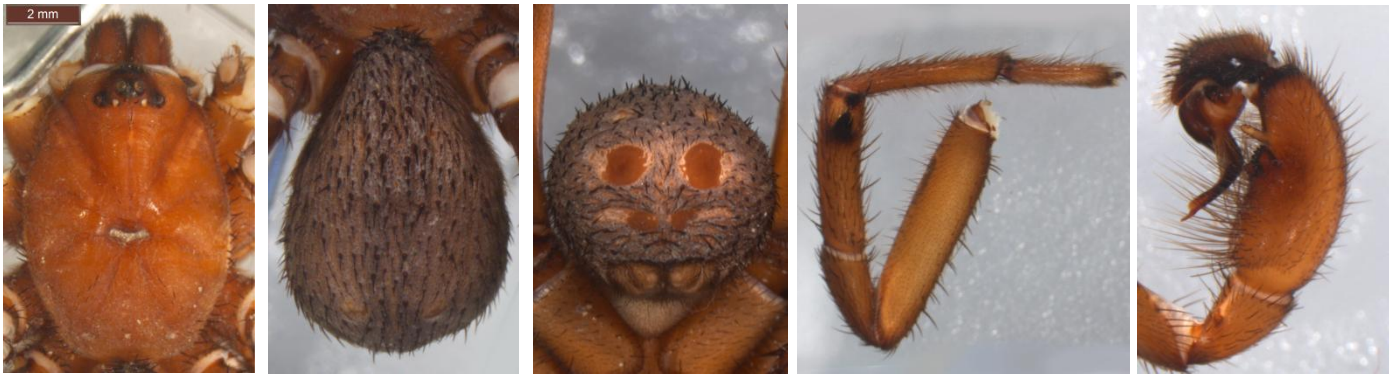

WAM T44169 male: East Yorkrakine Nature Reserve, WA

Idiosoma schoknechtorum sp. n. [MYG518]

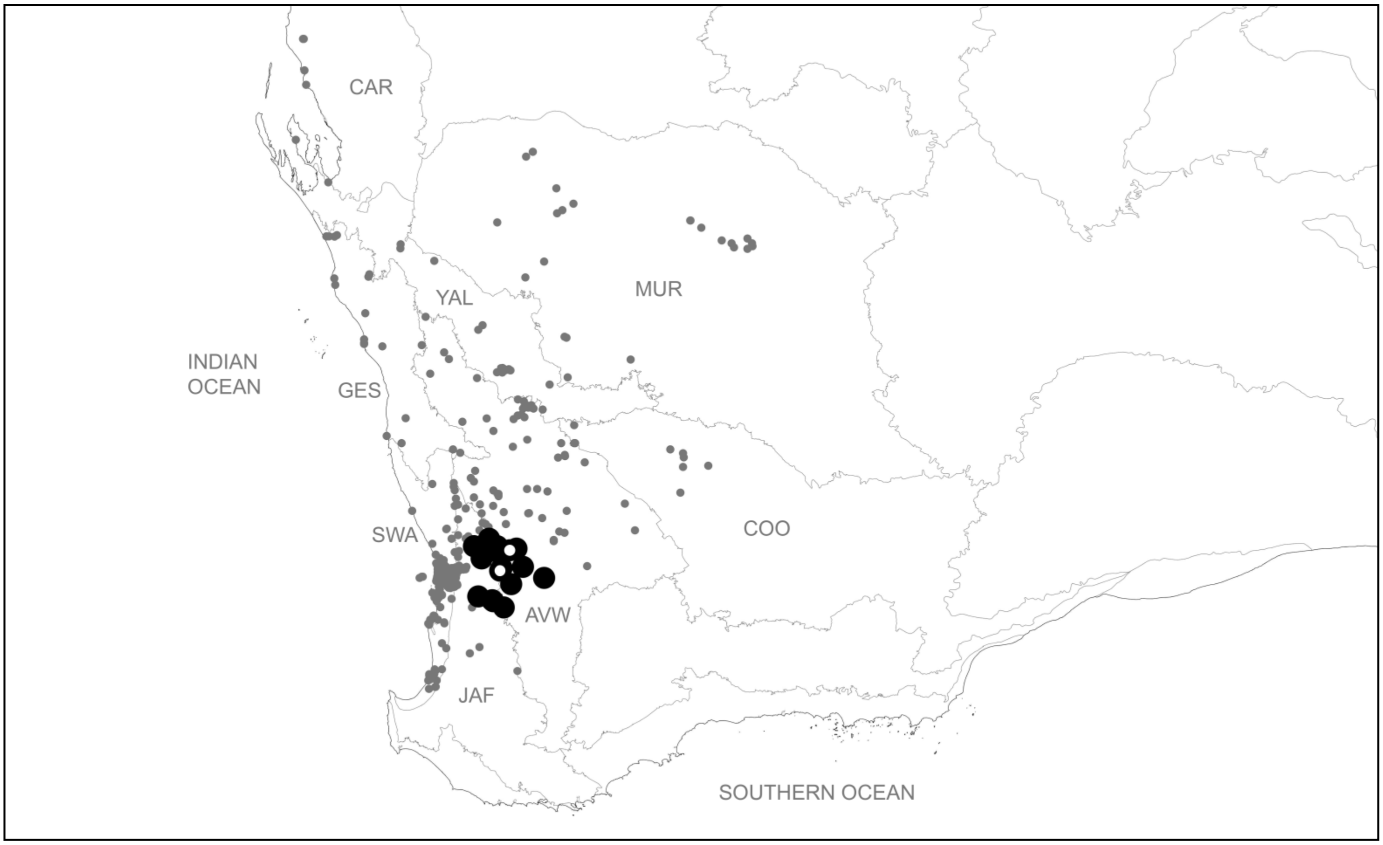

Collection records (open circles = DNA)

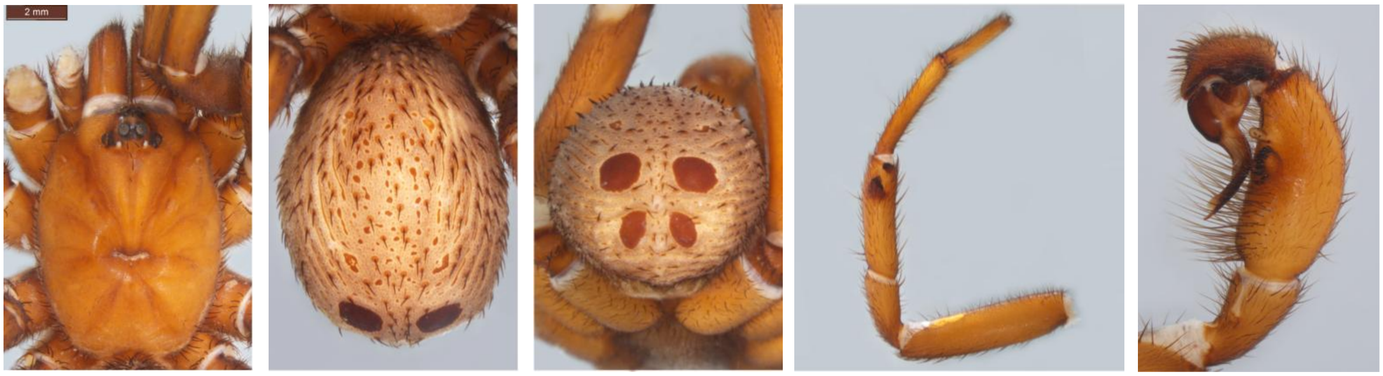

WAM T139512 male: Beverley, WA

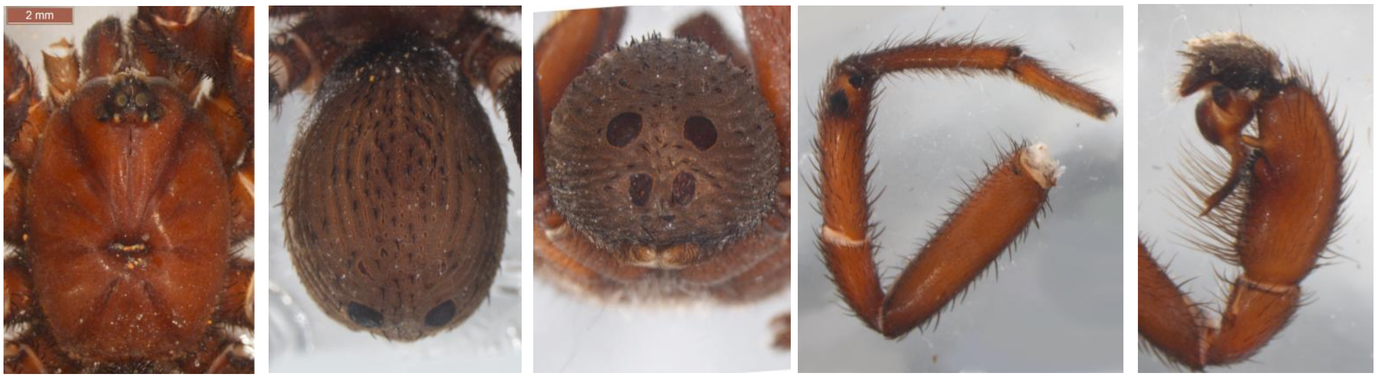

WAM T27121 male: Meckering, WA

Idiosoma schoknechtorum sp. n. [MYG518] (cont.)

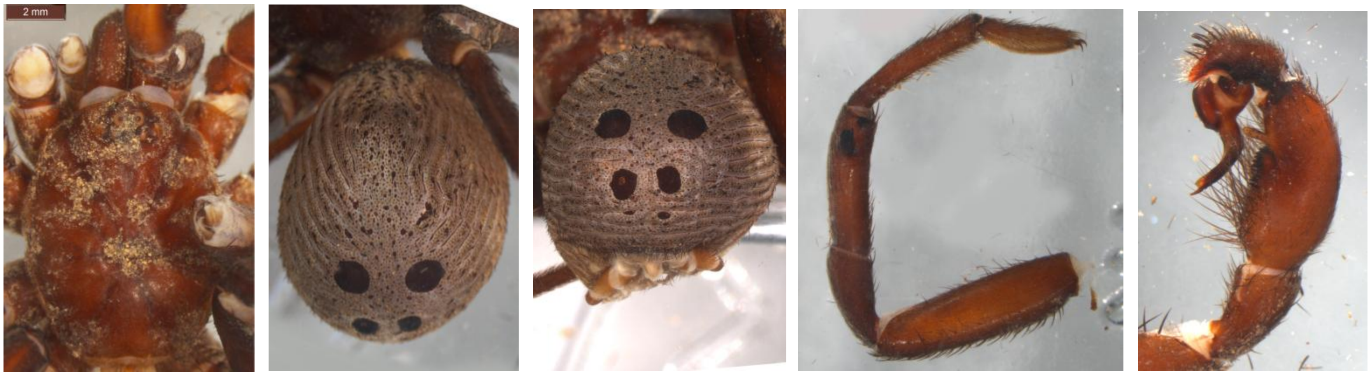

WAM T139513 male: Westdale, WA

Idiosoma sigillatum (O.P.-Cambridge, 1870)

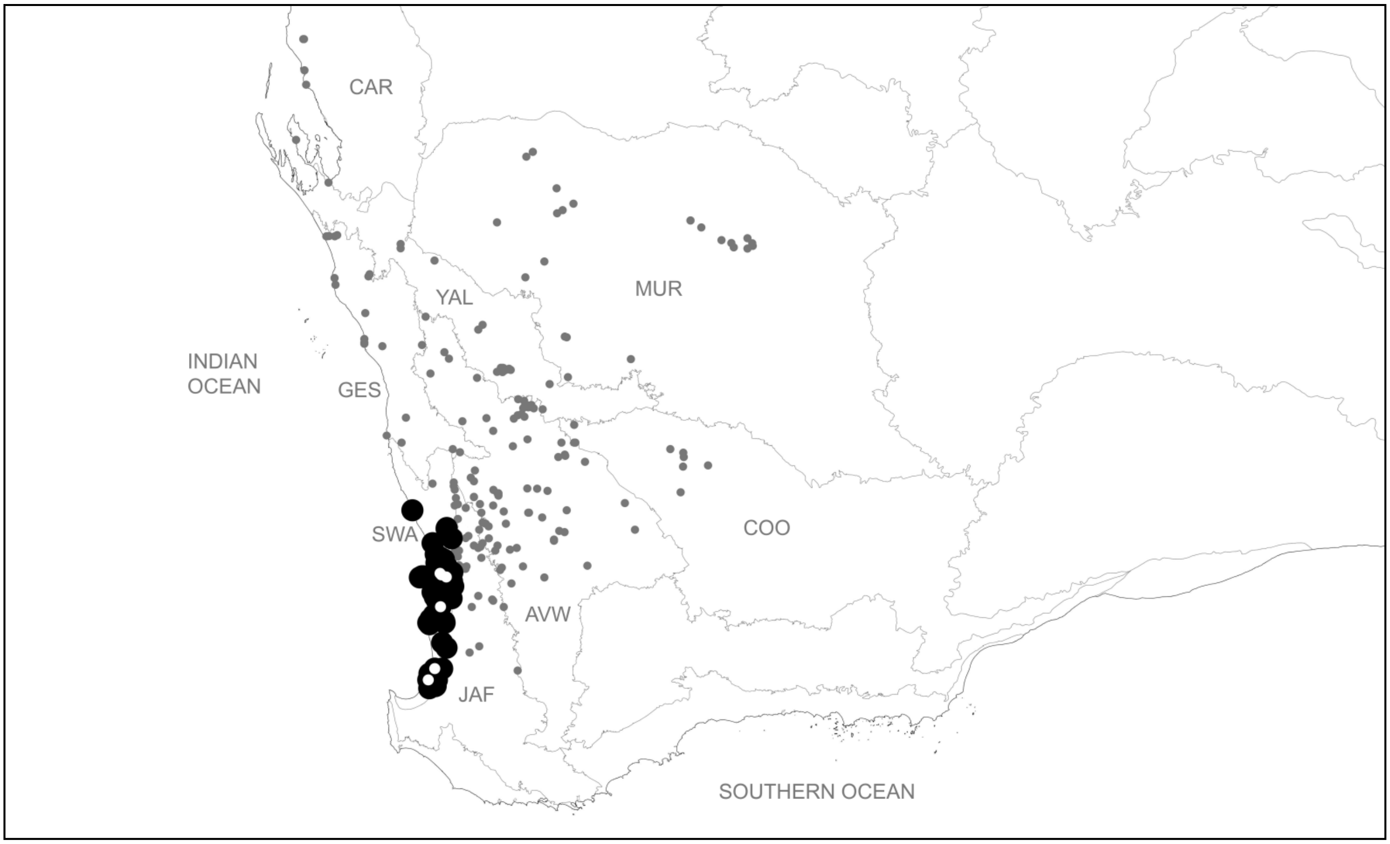

Collection records (open circles = DNA)

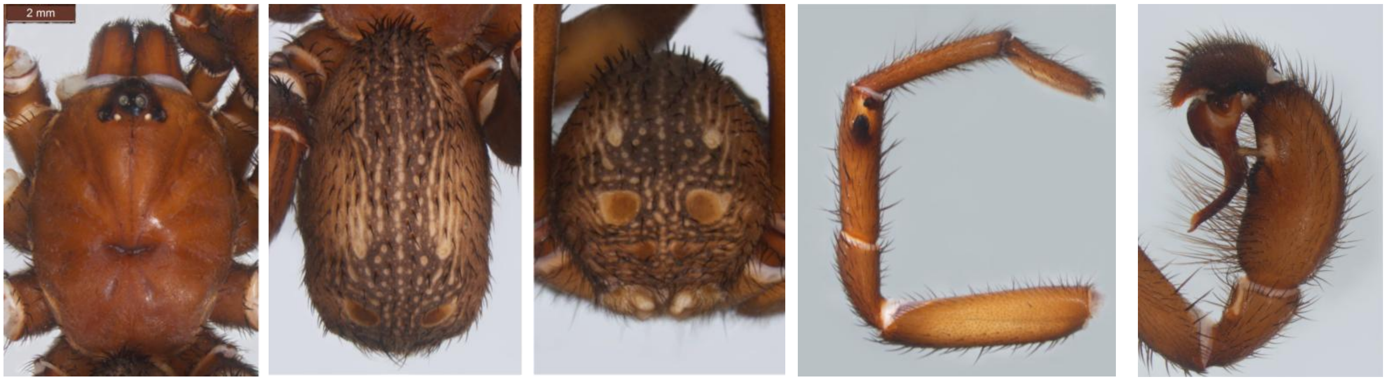

WAM T139480 male: Duncraig, Perth, WA [reference specimen]

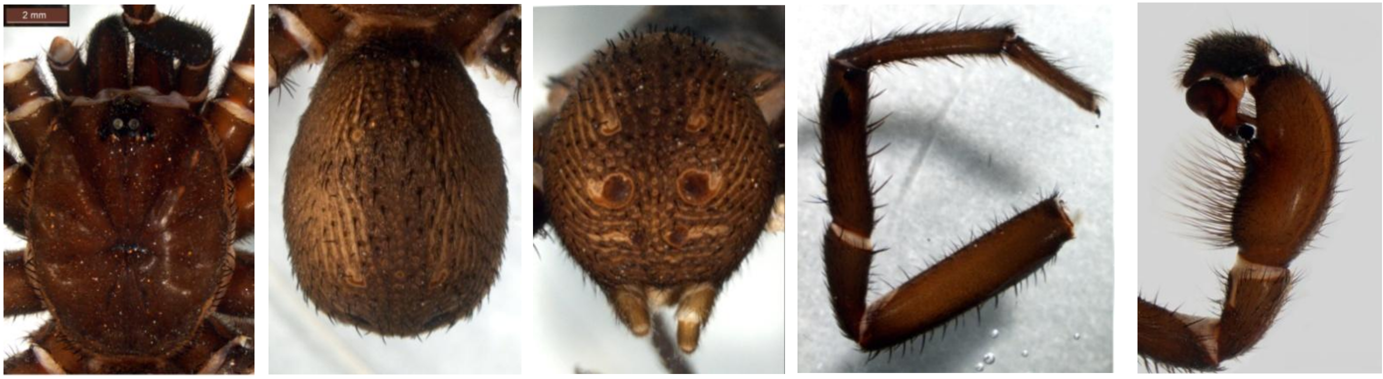

WAM T132564 male[DNA]**:** Crawley, Perth, WA

Idiosoma sigillatum (O.P.-Cambridge, 1870) (cont.)

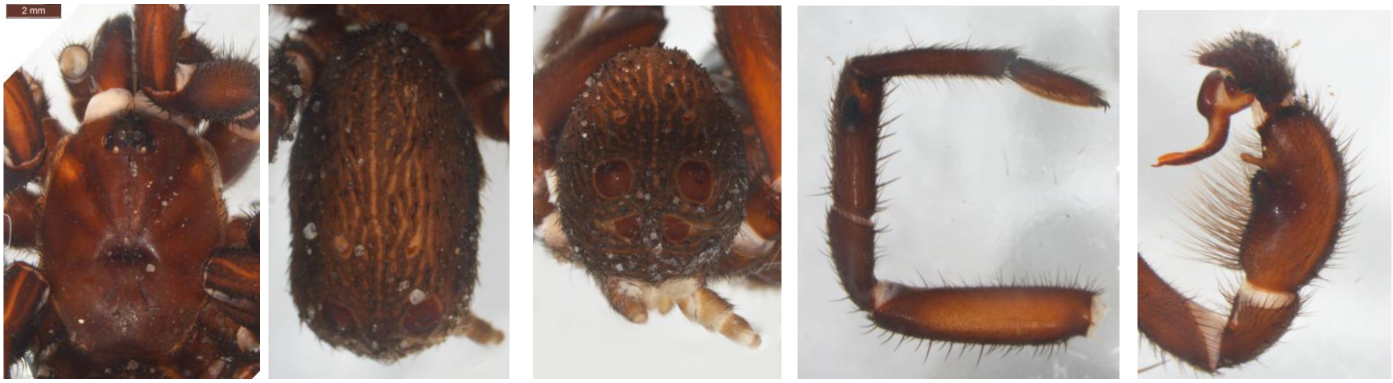

WAM T27984 male: Dardanup, WA

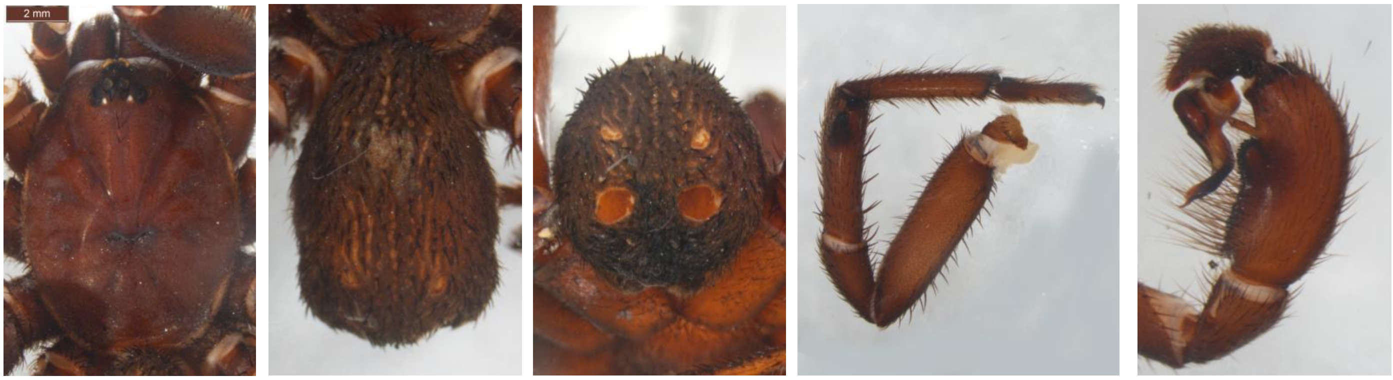

WAM T18552 male: Garden Island [sic “Naval Base”], WA

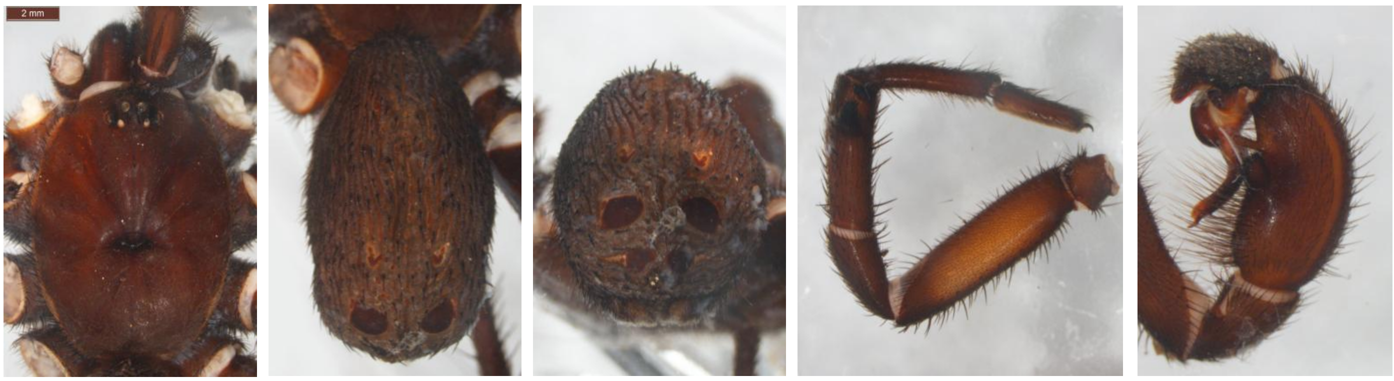

WAM T31152 male: Gelorup, WA

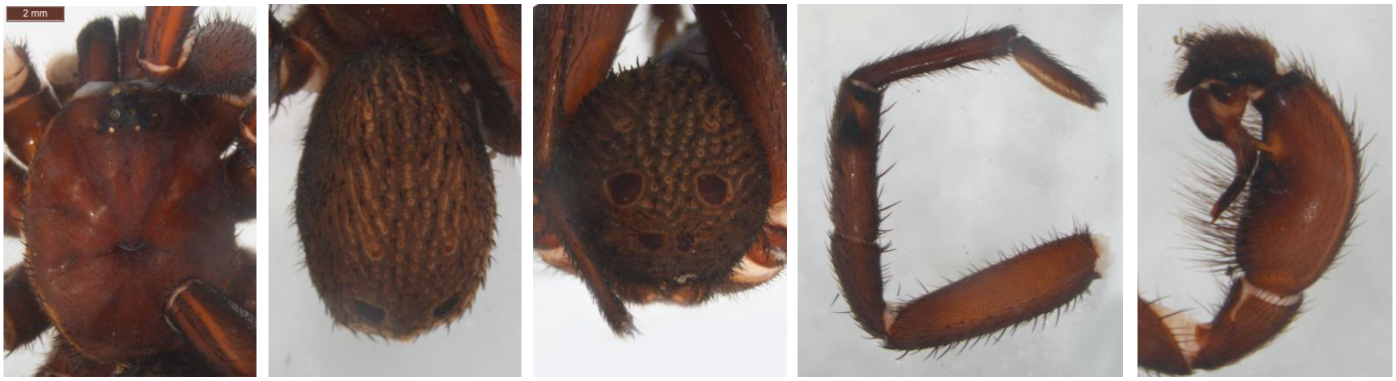

WAM T27135 male: Kings Park, Perth, WA [*NB. right palp flipped horizontal*]

Idiosoma sigillatum (O.P.-Cambridge, 1870) (cont.)

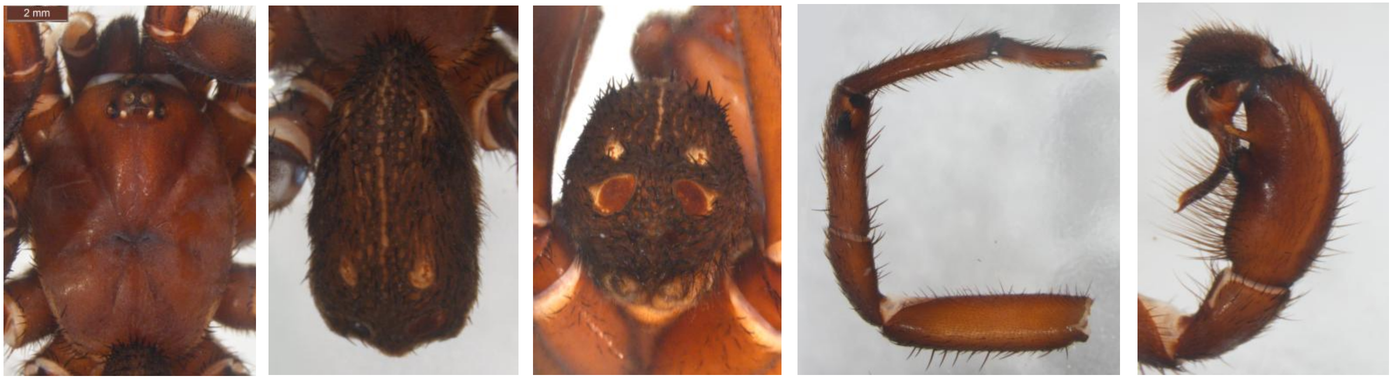

WAM T46829 male: Mandurah, WA

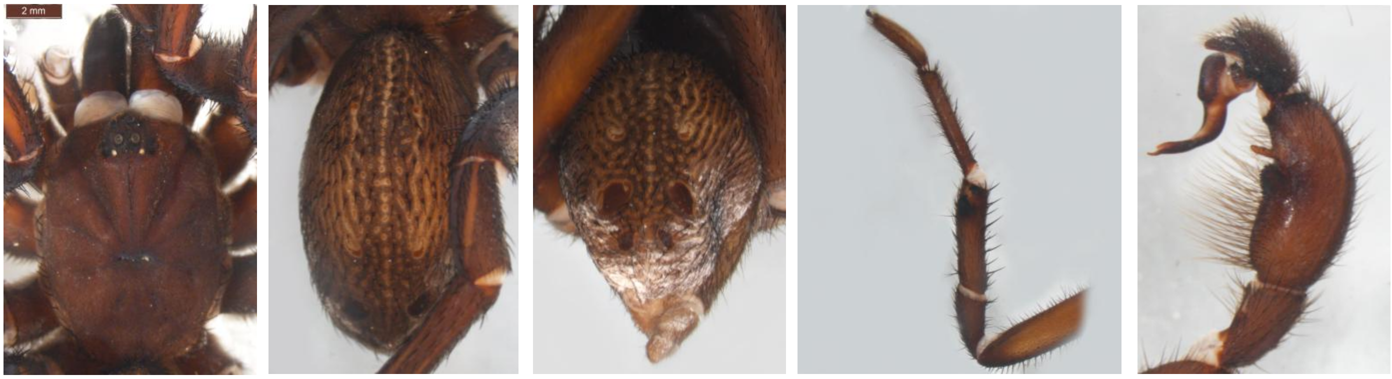

WAM T41569 male: Mundijong, WA

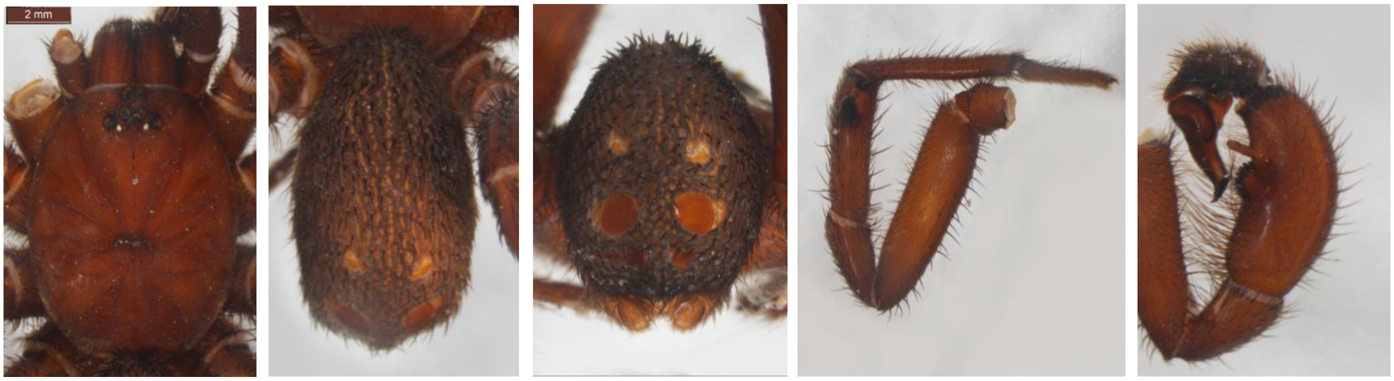

WAM T139486 male: Rottnest Island, WA

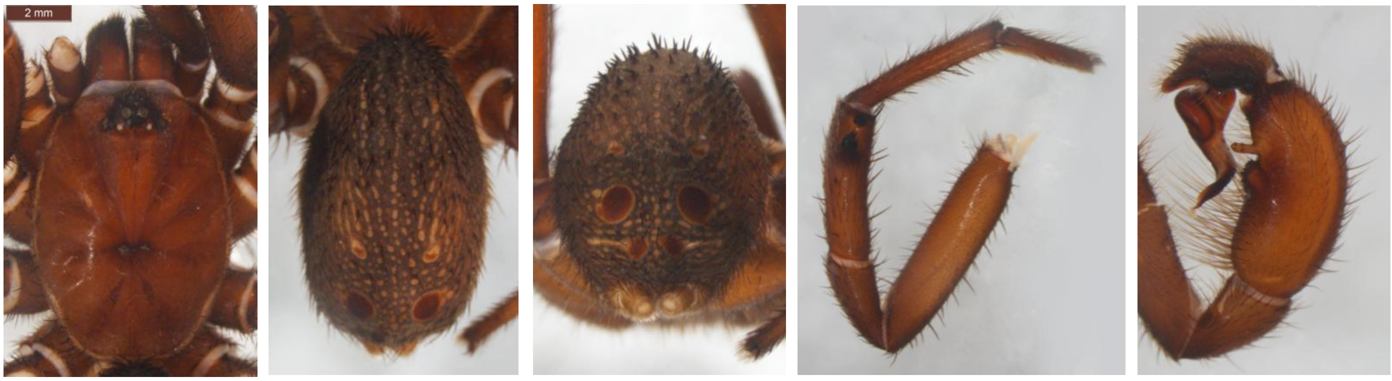

WAM T18575 male: Yanchep Park, WA
